# Accuracy and spatial properties of distributed magnetic source imaging techniques in the investigation of focal epilepsy patients

**DOI:** 10.1002/hbm.24994

**Published:** 2020-05-09

**Authors:** Giovanni Pellegrino, Tanguy Hedrich, Manuel Porras‐Bettancourt, Jean‐Marc Lina, Ümit Aydin, Jeffery Hall, Christophe Grova, Eliane Kobayashi

**Affiliations:** ^1^ Department of Neurology and Neurosurgery Montreal Neurological Institute, McGill University Montreal Quebec Canada; ^2^ IRCCS Fondazione San Camillo Hospital Venice Italy; ^3^ Department of Multimodal Functional Imaging Lab, Biomedical Engineering McGill University Montreal Quebec Canada; ^4^ Departement de Genie Electrique Ecole de Technologie Superieure Montreal Quebec Canada; ^5^ Centre de Recherches Mathematiques Montréal Quebec Canada; ^6^ Physics Department and PERFORM Centre Concordia University Montreal Quebec Canada

**Keywords:** interictal epileptiform discharges, inverse problem, magnetic source imaging, MEG, presurgical evaluation, source localization, spike

## Abstract

Source localization of interictal epileptiform discharges (IEDs) is clinically useful in the presurgical workup of epilepsy patients. We aimed to compare the performance of four different distributed magnetic source imaging (dMSI) approaches: Minimum norm estimate (MNE), dynamic statistical parametric mapping (dSPM), standardized low‐resolution electromagnetic tomography (sLORETA), and coherent maximum entropy on the mean (cMEM). We also evaluated whether a simple average of maps obtained from multiple inverse solutions (Ave) can improve localization accuracy. We analyzed dMSI of 206 IEDs derived from magnetoencephalography recordings in 28 focal epilepsy patients who had a well‐defined focus determined through intracranial EEG (iEEG), epileptogenic MRI lesions or surgical resection. dMSI accuracy and spatial properties were quantitatively estimated as: (a) distance from the epilepsy focus, (b) reproducibility, (c) spatial dispersion (SD), (d) map extension, and (e) effect of thresholding on map properties. Clinical performance was excellent for all methods (median distance from the focus MNE = 2.4 mm; sLORETA = 3.5 mm; cMEM = 3.5 mm; dSPM = 6.8 mm, Ave = 0 mm). Ave showed the lowest distance between the map maximum and epilepsy focus (Dmin lower than cMEM, MNE, and dSPM, *p* = .021, *p* = .008, *p* < .001, respectively). cMEM showed the best spatial features, with lowest SD outside the focus (SD lower than all other methods, *p* < .001 consistently) and high contrast between the generator and surrounding regions. The average map Ave provided the best localization accuracy, whereas cMEM exhibited the lowest amount of spurious distant activity. dMSI techniques have the potential to significantly improve identification of iEEG targets and to guide surgical planning, especially when multiple methods are combined.

## Graphical abstract

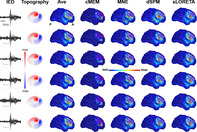

## 1 INTRODUCTION

Magnetoencephalography (MEG) is a valuable neuroimaging clinical and research tool to localize the epileptic focus in patients affected by focal epilepsy. Source localization of interictal epileptiform discharges (IEDs) detected with MEG can guide intracranial EEG (iEEG) targeting and help neurosurgical planning for cortical resection in drug‐resistant patients (Knowlton et al., 2006; Ryvlin, Cross, & Rheims, 2014; Stefan et al., 2003).

Mapping the epilepsy focus from IEDs recorded through MEG requires the solution of an ill‐posed inverse problem. This can be achieved thanks to several approaches, which largely belong to two main groups: localization techniques, consisting mainly in equivalent current dipole (ECD) source localization (Scherg & Von Cramon, 1985), and imaging techniques, including distributed magnetic source imaging using anatomical constraints (distributed magnetic source imaging [dMSI]) (Dale & Sereno, 1993; Hämäläinen & Ilmoniemi, 1994; Hillebrand, Singh, Holliday, Furlong, & Barnes, 2005; Ilmoniemi, 1993).

ECD‐based localization has been widely used and validated in clinical settings in North America as it is the unique technique approved by clinical US guidelines and Food and Drug Administration (Bagic et al., 2011; Barth, Sutherling, Engel, & Beatty, 1982; Bast et al., 2004; Knowlton et al., 1997; Stefan et al., 2003).

ECD only models a point source defined by three spatial coordinates, a magnitude and a direction. Therefore, from a theoretical point of view, ECD localization is only accurate and reliable for focal generators of limited spatial extent (Bagić, 2016; Hara et al., 2007; Ilmoniemi, 1993; Kanamori et al., 2013; Kobayashi, Yoshinaga, Ohtsuka, & Gotman, 2005; Pellegrino, Hedrich, et al., 2018; Scherg & Von Cramon, 1985; Shiraishi et al., 2005).

On the other hand, dMSI techniques are widely used in research settings, while gaining popularity as additional or even the sole method in many tertiary neuroimaging facilities for clinical applications, especially in Europe (Mouthaan et al., 2016; Mouthaan et al., 2019). An important development of dMSI relies on the usage of realistic anatomical constraints, distributing a set of dipolar sources along the cortical surface, where dipole orientation is set to be normal to the underlying cortical mesh. This was made possible through the early use of advanced segmentation techniques on individual anatomical MRI data (Dale & Sereno, 1993).

dMSI allows a better assessment of the spatial properties of the generator (Dale & Sereno, 1993; Darvas, Pantazis, Kucukaltun‐Yildirim, & Leahy, 2004), offering robustness to low signal to noise ratio data (Tanaka & Stufflebeam, 2013), and providing maps of cortical activations that are clinically relevant. In general, dMSI approaches are less operator dependent and rely on the assumption that the generators of MEG‐recorded activity are distributed over a spatial mesh and spatially extended (Tao, Baldwin, Hawes‐Ebersole, & Ebersole, 2007; von Ellenrieder, Beltrachini, Muravchik, & Gotman, 2014; von Ellenrieder, Beltrachini, Perucca, & Gotman, 2014; Pellegrino et al., 2019; Pellegrino et al., 2018; Raffin et al., 2015).

Therefore, dMSI approaches are particularly well suited to detect and analyze spatial–temporal propagations of IEDs and to recover the underlying spatial extent of their generator (Chowdhury et al., 2016; Chowdhury, Lina, Kobayashi, & Grova, 2013; Grova et al., 2016; Hämäläinen & Ilmoniemi, 1994; Sohrabpour, Lu, Worrell, & He, 2016; Tanaka et al., 2010; Tanaka & Stufflebeam, 2013; Zhu, Zhang, Dickens, & Ding, 2014; Zhu, Zhang, Dickens, King, & Ding, 2013; Giambattistelli et al., 2014). In a recent study, we investigated a large sample of MEG datasets from epilepsy patients and demonstrated that dMSI accuracy was similar to that of ECD (Pellegrino, Hedrich, et al., 2018). We focused on one dMSI method entitled the maximum entropy on the mean (MEM; Amblard, Lapalme, & Lina, 2004), which was developed and carefully evaluated in the context of presurgical study of patients with epilepsy. Its extended version, called coherent MEM (cMEM), has an unique robustness in recovering the spatial properties of IEDs generator along the cortical surface (Chowdhury et al., 2013; Chowdhury et al., 2016; Grova et al., 2016; Pellegrino, Machado, et al., 2016; Pellegrino, Hedrich, et al., 2016; Pellegrino, Hedrich, et al., 2018). Several dMSI methods have been proposed (Baillet, Mosher, & Leahy, 2001), whereas only few studies compared their performance in a systematic way with a large epilepsy dataset (de Gooijer‐van de Groep, Leijten, Ferrier, & Huiskamp, 2013; Mouthaan et al., 2019; Rampp et al., 2019; Tenney, Fujiwara, Horn, & Rose, 2014).

In the present study, we report MEG source localization performance on patients with a well‐characterized focus based on noninvasive and invasive evaluation of their epilepsy. We quantitatively compared the performance of dMSI approaches and tested whether combining multiple inverse solutions can improve localization accuracy. We compared four dMSI techniques most commonly used in presurgical evaluation and which are freely available and easy to translate into clinical settings: (a) cMEM (Chowdhury et al., 2013); (b) minimum norm estimate (MNE) (Hämäläinen & Ilmoniemi, 1994); (c) standardized low‐resolution electromagnetic tomography (sLORETA) (Pascual‐Marqui, Esslen, Kochi, & Lehmann, 2002); and dynamical statistical parametric mapping (dSPM) (Dale et al., 2000).

## 2 METHODS

### 2.1 Patients

The study was approved by the Research Ethics Board of the Montreal Neurological Institute and Hospital—McGill University Health Center. All patients signed a written informed consent prior to enrollment. All experimental procedures were performed in agreement with the Declaration of Helsinki and its later amendments. We retrospectively reviewed all focal epilepsy patients scanned over a period of 9 years between 2006 and 2015 and included those who underwent presurgical assessment leading to accurate identification of the epilepsy focus. The identification of the epileptic focus was deemed accurate and the patient included when at least one of the following was satisfied: (a) patient underwent surgery and became seizure free, (b) invasive EEG capturing interictal and/or ictal activity, and (c) epileptogenic MRI lesion concordant with scalp EEG findings. We excluded patients whose MEG had magnetization artifact, patients with extensive brain lesions that could affect the estimation of the forward model and patients affected by mesial temporal lobe epilepsy, as this condition does not require a presurgical MEG assessment (Pellegrino, Hedrich, et al., 2018). We also excluded patients having less than five spikes per study to comply with American Clinical Magnetoencephalography Society guidelines and to avoid a too low signal to noise ratio. Clinical data are reported in Table 1.

**Table 1:** Epidemiological and clinical features

		EEG		SEEG					
PA	Sex/age	IEDs	Ictal	Electrodes/side:regions	Interictal	Ictal	MRI findings	Surgery	Engel class
1	M/33	LP	LP				LP FCD		
2	M/35	LP	L FCP				L precuneus FCD		
3	F/15	Bil F	Bil F				R F gyration abnormality		
4	F/27	L FT	L FT	5/L: A, Ha, Hp, Ca, OF	LF, LT	LF, LT	Sphenoethmoidal meningoencephalocele	L FO	1
5	M/15	Bil C	Bil C	3/R: RAC, SMA, Lesion	Lesion	Lesion	R F parasagittal FCD	R F	1
6	M/16	Bil FC (L > R)	Bil F	5/R:OF, Ca, Cm, SMAa, SMAp; 5/L: OF, Ca, Cm, SMAa, SMAp	Bil F (L > R)	Bil F, max L SMA		L F	2
7	M/24	BiF F (L > R)	L F	4/R: OF, Ca, Cp, Lesion; 4/L: OF, Ca, Cp, F	Lesion, Bil F	Bil F, max lesion	L F Fa FCD, parasagittal	L F FCD	4
8	F/20	RF					R F FCD		
9	M/32	Bil F (R > L)	RF	9/R: OF, Ca, Cm, SMAa, SMAp, Ia, Ip, A; 1/L:Hc	R OF, Ia	R OF, Ia	R hemimegalencephally	R OF	4
10	M/32	R FT	RF	9/R: A, H, Ia, Ip, OF, Ca, Cm, SMAa, SMAp	OF, Ia, A, H	OF, Ia, operculum	R hemimegalencephally	R OF	4
11	F/25	R FT	RF	9/R: A, Ha, Hp, Im, OF, Ca, Cm, SMAa, SMAp	OF, F convexity, Ta neocortex	OF	R F FCD	R OF	1
12	M/20	L CP	L CP	8 × 8 GRID L FCT convexity	Rolandic m	Sensory P		L post C	4
13	M/41	R FC	R FC	9/R: A, Ha, Hp, Ip, OF, SMA, Ca, Cp, P	H, SMA, Cm, OF	T,SMA		R F	3
14	F/21	RF	RF	2/L: Ha, Hp; 7/R: Ha, Hp, A, SMAa, SMAm, SMAp, Ca, Cp	SMAa, SMAm, or SMAp	SMA, overlapping with FCD	R F FCD	RF	4
15	F/24	Bil F	Bil F (R > L)	7/R: H, OF, Fp, Ca, Cm, SMAa, SMAp; 2/L:OF, Ca	Bil F (R > L)	Bil F (R > L)	R F FCD		
16	M/39	RC	R FC	2/L: SMAa, SMAp; 6/R: H, I, Ca, Cp, SMAa, SMAp	R SMAp	R SMAp	R FC parasagittal FCD		
17	M/39	R C	R FC	2/L: SMAa, SMAp; 6/R: H, I, Ca, Cp, SMAa, SMAp	R SMAp, R CP	R SMAp	R FC parasagittal FCD		
18	M/34	Bil FC	R FC	7/R:A, H, OF, Ca, Cm, Ia, Ip	Fm	Fm	R Fm FCD	R Fm	1
19	M/38	R FT	R FT	8/R: Fa, OF, Ca, Cp, SMAa, SMAp, A, H	OF	OF	R OF FCD	R OF	1
20	F/38	L FT	L FT	5/L: A, Ha, Hp, Ca, OF	Neocortical and mesiotemporal, multifocal or widespread	Neocortical and mesiotemporal, multifocal or widespread	Cerebral herniation off the left orbitofrontal region through orbital bone, left hippocampus malrotation	L A H	4
21	M/29	L FC	L FC				R F polar FCD	R F polar	1
22	M/35	LT	L FT	2/R: Ca, Ha; 9/L: A, Ha, Hp, OF, Ia, Fa, Ca, SMAa, SMAp	L I, FO, T	FO	L OF FCD	L F	3
23	M/55	RT	RT				R T anomaly	RT	3
24	F/22	L FT	L FCT	7/L:OF,G. Rectus, Ca, Cp, LA, Ha, Hp	L Ca, T pole, Hp, A	L Ca Ta	L Ca, OF FCD	L Ta, OF	4
25	F/19	L FT	L FT				L F (opercular) FCD		
26	F/22	L P	—				L postcentral FCD		
27	F/30	L FC	L F				L F (precentral) FCD		
28	F/28	Bil F (L > R)	L F				L Ca FCD	L F	1

Abbreviations: A, amigdala; a, anterior; Bil, bilateral; C, central; C, cingulate; F, frontal; FCD, focal cortical dysplasia; H, hippocampus; I, insula; IED, interictal epileptiform discharge; L, left; m, middle; O, occipital; OF, orbito frontal; P, parietal; p, posterior; Post Quad, posterior quadrant; R, right; SMA, supplementary motor area; T, temporal.

### 2.2 Data acquisition

MEG scans were performed at the Psychology Department of University of Montreal between 2006 and 2012 and at McConnell Brain Imaging Center of the Montreal Neurological Institute from 2012 to 2015. In both centers, MEG signals were recorded with a CTF MEG system (MISL, Vancouver, Canada) equipped with 275 gradiometers. Dedicated bipolar electrodes were applied to detect eye movements and electrocardiogram. Head position in the dewar was continuously monitored using three localization coils placed on anatomical landmarks (nasion and left and right ear). At least 200 head points were digitized using a Polhemus localization system for MRI–MEG coregistration. The sampling rate was set to 600 Hz or higher. The acquisition was divided in blocks of 6 min and lasted for about 1 hr. All acquisitions were performed with the patient lying in a supine position. Patients were given the following instruction: “Clear your mind and stay relaxed.” In order to build an accurate individual head model, each patient underwent an MRI scan in a Siemens Tim Trio 3 T scanner. We acquired a high‐resolution anatomical T1W MPRAGE sequence with the following parameters: 1 mm isotropic 3D images, 192 sagittal slices, 256 × 256 matrix, TE 52.98 ms, and TR 52.3 s.

### 2.3 MEG data preprocessing

MEG data analysis was performed using Brainstorm toolbox (Tadel, Baillet, Mosher, Pantazis, & Leahy, 2011)—and MATLAB in‐house script (The MathWorks, version 2017b). Preprocessing consisted of: (a) third‐order spatial gradient noise cancelation, (b) DC offset removal, (c) 60‐Hz line frequency notch and (0.3–70 Hz) bandpass filters, and (d) resampling to 600 Hz (whenever necessary) (Pellegrino, Maran, et al., 2018; Pellegrino et al., 2019). MEG IEDs were visually identified and marked at their peak by trained neurophysiologists (G. P. and E. K.). MEG signal was then segmented into epochs of 2 s around IED peak (−1 to +1 s). IEDs were classified according to topographical distribution of the magnetic field and their morphology. IEDs occurring at the time of artifacts or EKG QRS complex were excluded. Epochs of the same type belonging to the same run were averaged. Each averaged IED group corresponded to a source imaging “study” (Heers et al., 2016; Pellegrino, Hedrich, et al., 2016; Pellegrino, Hedrich, et al., 2018).

### 2.4 Forward model

Individual MRIs were processed using the FreeSurfer toolbox (Dale, Fischl, & Sereno, 1999). Skull surface was reconstructed from individual MRI in Brainstorm. MEG‐MRI coregistration was ensured using a procedure of surface fitting with a rigid geometrical transformation (three rotations, three translations) between the head shape segmented from MRI and the head fiducials and the head points previously digitized. We selected the mesh of the “mid” layer equidistant from the white/gray matter interface and the pial matter as source space for source imaging and downsampled it to 8,000 cortical vertices. The distributed source model consisted in dipolar sources on every vertex of the mesh, oriented perpendicularly to the cortical surface. The forward model assessing the contribution of every dipolar source to MEG sensors was built with a 1‐layer boundary element method (BEM) method (Kybic, Clerc, Faugeras, Keriven, & Papadopoulo, 2006) as implemented in the OpenMEEG toolbox (Gramfort, Papadopoulo, Olivi, & Clerc, 2010). This BEM model was computed using the inner‐skull surface segmented by Brainstorm for every patient and cortical conductivity was set to 0.33 S/m.

### 2.5 dMSI methods

Source maps were estimated at the time of the peak of the average IED. Noise‐covariance was modeled using a 1 s baseline without any visually identified IEDs.

We computed the following inverse solutions applying default Brainstorm parameters:

1. *MNE* (Hämäläinen & Ilmoniemi, 1994): This is the “depth‐weighted” linear L2‐MNE current density (also known as wMNE) which imposes a constraint of minimum energy on the source map, which is equivalent to Tikhonov regularization to solve an underdetermined linear problem (i.e., MNE minimizes the L2‐norm of the current distribution).
2. *sLORETA* (Pascual‐Marqui et al., 2002) is a standardized version of MNE, which normalizes the MNE solution by its resolution matrix, allowing sLORETA to be sensitive to deeper generators and to provide no localization error in the absence of measurement noise.
3. *cMEM* (Amblard et al., 2004; Chowdhury et al., 2013): This is a nonlinear probabilistic Bayesian approach relying on maximizing relative entropy. cMEM allows “switching off” the cortical parcels that do not contribute to the solution, while preserving the ability to create a contrast of current intensities within the active parcels and is sensitive to the spatial extent of the generator (Chowdhury et al., 2013; Chowdhury et al., 2016; Grova et al., 2016; Hedrich, Pellegrino, Kobayashi, Lina, & Grova, 2017; Heers et al., 2016).
4. *dSPM* (Dale et al., 2000) is another noise‐normalized version of MNE using a source noise covariance matrix and the whitened forward solution to obtain time‐varying statistical maps of neuronal activity.

In order to assess whether the combination of multiple source imaging techniques can achieve better accuracy, we computed an *Average* of the maps (denoted Ave) resulting from the four previously described source imaging methods. Before averaging, the original maps were rescaled so that the amplitude at each cortical vertex ranged between 0 (no activity) and 1 (maximum activity).

The result of dMSI source imaging is a map (dMSI map) where each vertex of the cortical surface is characterized by an intensity indicating its contribution to the signal recorded at sensor level.

Finally, we have also performed a standard analysis with the ECD technique (Bagic et al., 2011), which is reported in the supplementary material (Supplementary Material, Dipole Analysis), as the focus of this study was on dMSI.

### 2.6 Definition of the epileptic focus

Prior to MEG data analysis, the epileptic focus was manually drawn along the cortical surface based on the available clinical information for each patient. This information is included in Table 1, and consisted of (in order of priority; not all factors were available for every patient): resected region, ictal and interictal invasive EEG findings, visible lesion in the MRI, ictal and interictal scalp EEG findings. The identification of the region was never performed on scalp EEG findings alone, and an epileptogenic MRI lesion or invasive recordings were always required. In patients who became seizure free after surgery, the epileptic region corresponded to the surgical cavity and an ad hoc analysis was performed for this small group. The location of the epileptic focus was then independently identified by two epileptologists (E. K. and G. P.) over the individual cortical surface, and a consensus was reached on its extension upon discussion, blind to source localization results, following the same methodology proposed in our previous studies (Pellegrino, Hedrich, et al., 2018; von Ellenrieder et al., 2016).

### 2.7 Assessment of performance and spatial properties of dMSI methods

The following quantitative metrics of localization accuracy and spatial properties were estimated.

Measures of distance from the focus and across methods:

1. *Dmin*: Dmin was estimated as the minimum Euclidean distance expressed in mm between the maximum of the dMSI map (vertex exhibiting the largest current amplitude) and the epileptic focus.
2. *Reproducibility of Dmin*: This measure was computed for the patients having multiple studies and corresponded to the within‐subject and within‐method interquartile range of *Dmin*. This measure is expressed in mm.
3. *Inter_dMSI distance*: For each dMSI method, we computed the average of the distances between the maximum vertex of a given dMSI map and the maxima of all corresponding maps computed with the other inverse methods. This measure provides an estimate of the spatial concordance between one method and all the others, expressed in mm.

Quantitative assessment of source maps spatial characteristics.

1. *Spatial dispersion* (*SD*): SD measures the spatial spread of a current source density map and is computed as follows:
  $$\mathrm{SD}=\sqrt{\frac{\sum_{i=1}^{p}\left(\min_{j\in \Theta }\left(D_{\mathrm{ij}}^{2}\right){\hat{j}}_{i}^{2}\right)}{\sum_{i=1}^{p}{\hat{j}}_{i}^{2}}}$$
  where Θ is the reference region (the epileptic focus in our case), and ${\hat{j}}_{i}$ is the amplitude of the current density distribution estimated for the dipolar source *i*. The amplitude of all the *p* cortical sources is weighted by their minimum distance from all the dipolar sources belonging to Θ. $\min_{j\in \Theta }\left(D_{\mathrm{ij}}\right)$ provides the minimum distance between the source *i* and the closest dipolar source of Θ. SD provides a measure in millimeters and a source localization method with either high spatial spread around Θ or high localization error will result in high SD. Further details on the computation of this quantitative measure can be found in Molins, Stufflebeam, Brown, and Hämäläinen (2008) and Pellegrino, Hedrich, et al. (2016). The effect of thresholding on SD was also estimated by thresholding the map $\hat{J}$ before estimating the resulting SD.
2. *Map size*: dMSI map size was measured as the number of active cortical vertices. This measure is sensitive to the threshold applied to dMSI maps. When no threshold is applied (denoted as 0% threshold), the entire cortical surface is active, and all cortical vertices have nonzero values. As the cortical surface was tessellated into 8,000 points, Map size is 8,000 when 0% threshold is applied. When the maximum threshold is applied (denoted as 100% threshold), all cortical vertices but the one with highest amplitude are set to zero and the Map size is 1. Map size can therefore range between 1 and 8,000, depending on the threshold and on the spatial properties of the dMSI map. This metric was proposed to investigate the spatial extent of dMSI maps as a function of the threshold.
3. *Map_Dmin*: At a given threshold, the resulting dMSI map was binarized and the minimum distance between this new binary map accounting for the extent of the generator and the epileptic focus is computed. This metric is expressed in mm. Therefore, as compared to Dmin, which only relies on the position of the map maximum, Map_Dmin accounts for the spatial extent of the generator and is highly dependent on the threshold applied. If the dMSI map maximum is outside the epileptic focus, higher threshold reduces the map size and increases Map_Dmin; conversely, lower threshold increases map size and reduces Map_Dmin. Ultimately, the differences in Map_Dmin across dMSI methods depend on the spatial properties of the dMSI maps.

Curves of SD, Map size, and Map_Dmin measures as function of map thresholding were estimated for thresholds between 0 and 100%, in steps of 10%.

A patient‐based analysis restricted to Dmin was performed in addition to the study‐based statistical analysis. To this aim, intrasubject Dmin was computed as the median Dmin across subject's studies. Finally, an additional Dmin analysis was restricted to the studies of operated patients with seizure freedom (Engel 1a outcome) at 1 year following surgery.

As the distribution of several variables was not Gaussian, statistical significance was assessed by applying nonparametric tests (Friedman test and Wilcoxon Signed‐Rank tests). For the comparison of SD, Map size and Map_Dmin curves as function of the threshold and of the method to generate the cortical map (MNE, sLORETA, cMEM, dSPM, Ave), we applied a generalized estimating equation (GEE) model, with “study” as cluster variable and SD (or Map size or Map_Dmin) as dependent variable. This is an extension of the generalized linear model particularly suitable for correlated data and repeated measures (Hanley, Negassa, Edwardes, & Forrester, 2003; Hardin & Hilbe, 2012). For the purpose of this model, only three threshold values were considered: 30, 60, and 90%. The database with SD, Map size, and Map_Dmin for all studies, inverse solutions, and thresholds (0–100% in 10% steps) is provided as supplementary material. Significance levels were set at *p* < .05. Bonferroni correction for multiple comparisons was applied when appropriate.

### 2.8 Data availability

Individual results are available as supplementary material. The original raw data supporting the findings of this study are available upon reasonable request to the corresponding authors.

## 3 RESULTS

A total of 206 studies from 28 patients (age 27.28 ± 7.59 years, 15 males) were analyzed. Clinical details are reported in Table 1. The clinical gold standard reference (epileptic focus) was based on MRI lesion + iEEG + Surgery (*N* = 12), MRI lesion + Surgery (*N* = 3), iEEG + Surgery (*N* = 3), iEEG + MRI lesion (*N* = 3), and MRI lesion alone (*N* = 7 patients).

### 3.1 Measures of distance from the focus and across methods

**Figure 1:**
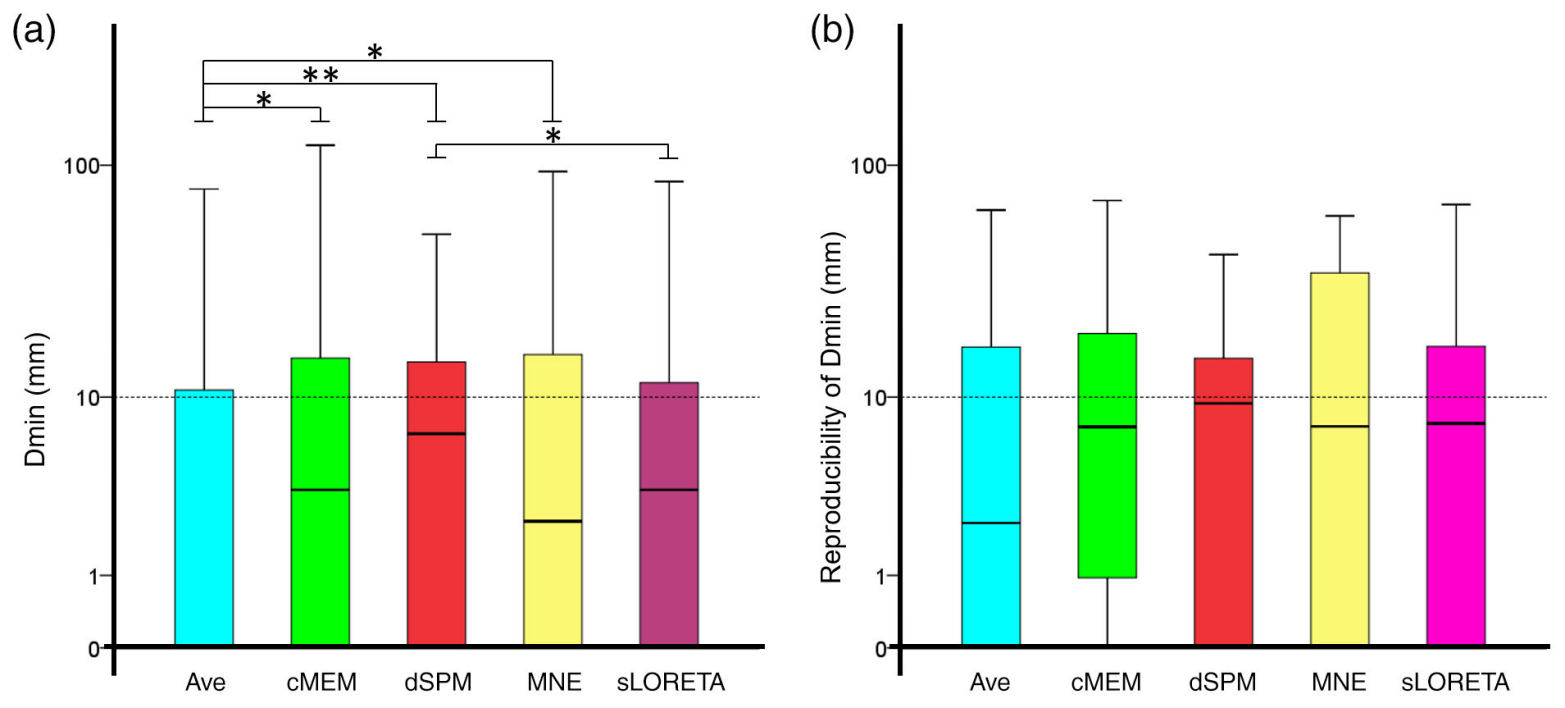
(a) Median Dmin was below 1 cm for all methods. The performance of standardized low‐resolution electromagnetic tomography (sLORETA) was slightly but significantly better than the one of dynamic statistical parametric mapping (dSPM). The performance of Ave (median = 0 mm) was significantly better than coherent maximum entropy on the mean (cMEM), dynamic statistical parametric mapping (dSPM), and minimum norm estimate (MNE). (b) Reproducibility of Dmin measured as within‐subject interquartile range was not significantly different across methods. Its median was below 1 cm for all methods. All graphs are boxplots depicting median and quartiles. The dash line is set to 1 cm. * denotes *p* < .05; ** denotes *p* < .001

Median Dmin was found below 1 cm for all methods (median: MNE = 2.359 mm; sLORETA = 3.530 mm; cMEM = 3.530 mm; dSPM = 6.753 mm, Ave = 0.000 mm), however, with significant differences across them (Friedman's test Chi‐square = 33.987, *df* = 4, *p* < .001). Post hoc analysis revealed that sLORETA Dmin was significantly better than dSPM (*p* = .034, Bonferroni corrected) and Ave Dmin was significantly lower than cMEM, MNE, and dSPM (Bonferroni corrected, *p* = .021, *p* = .008, *p* < .001, respectively) (Figure 1a). These results were confirmed on the subsample of studies (*N* = 65) of postsurgical seizure‐free patients (*N* = 7) (Friedman's test Chi‐square = 36.697, *df* = 4, *p* < .001), with *Ave* being more accurate than cMEM, MNE, and sLORETA (median: MNE = 5.110 mm; sLORETA = 4.843 mm; cMEM = 3.530 mm; dSPM = 11.425 mm, Ave = 3.530 mm).

The patient‐based analysis revealed that all methods had a median Dmin value of 0 mm, with no significant differences across methods (*p* > .100).

Reproducibility of Dmin (Patients *N* = 24) differed across methods (Friedman's test Chi‐square = 12.118, *df* = 4, *p* = .016), but post hoc comparisons did not unveil any significance after correction for multiple comparisons. To be noted, the median interquartile range was below 1 cm for all methods (Figure 1b) (median: MNE = 7.600 mm; sLORETA = 7.557 mm; cMEM = 7.352 mm; dSPM = 9.366 mm, Ave = 2.302 mm).

**Figure 2:**
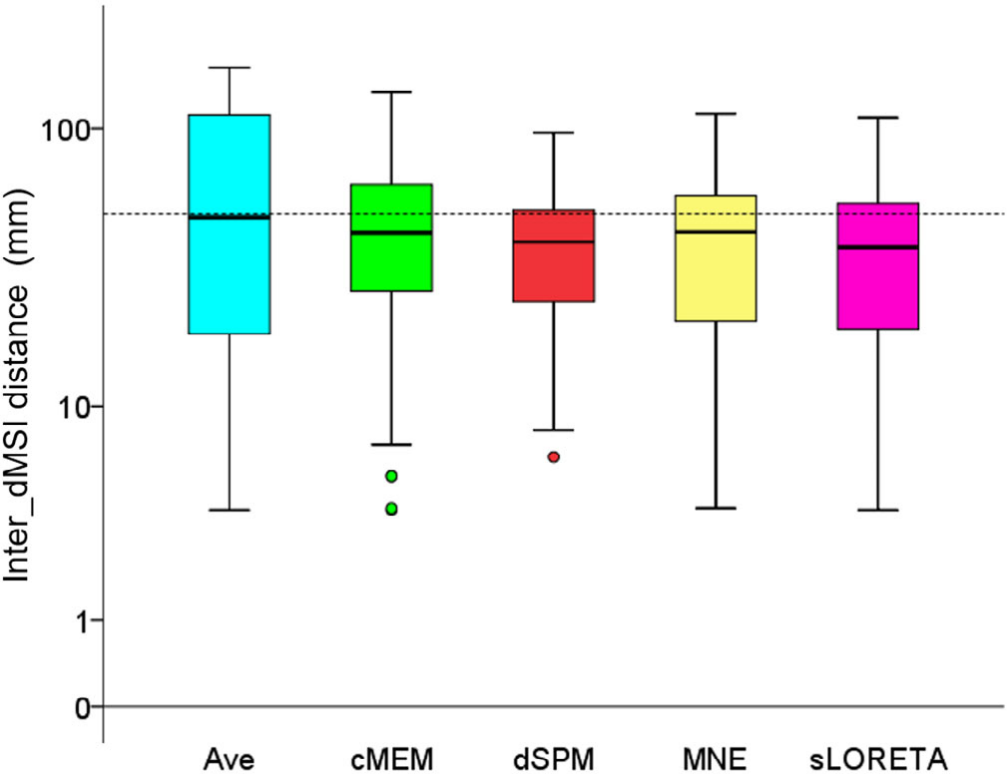
Inter_dMSI distance. This measure provides an estimate of the spatial concordance between one method and all others and is expressed in mm. Inter_dMSI distance was significantly different across inverse methods, but the median value was consistently around 5 cm. This analysis confirms that there is a remarkable variability in the position of the map maximum across inverse solution techniques and supports the attempt to combine inverse methods to achieve a common map owning higher accuracy. Boxplots depict median and quartiles. The dash line is set to 5 cm

Inter_dMSI distance was significantly different across methods (Friedman's test Chi‐square = 40.329, *df* = 4, *p* < .001; median: MNE = 26.899 mm; sLORETA = 23.473 mm; cMEM = 31.765 mm; dSPM = 25.220 mm, Ave = 60.123 mm) (Figure 2).

### 3.2 Spatial properties

**Figure 3:**
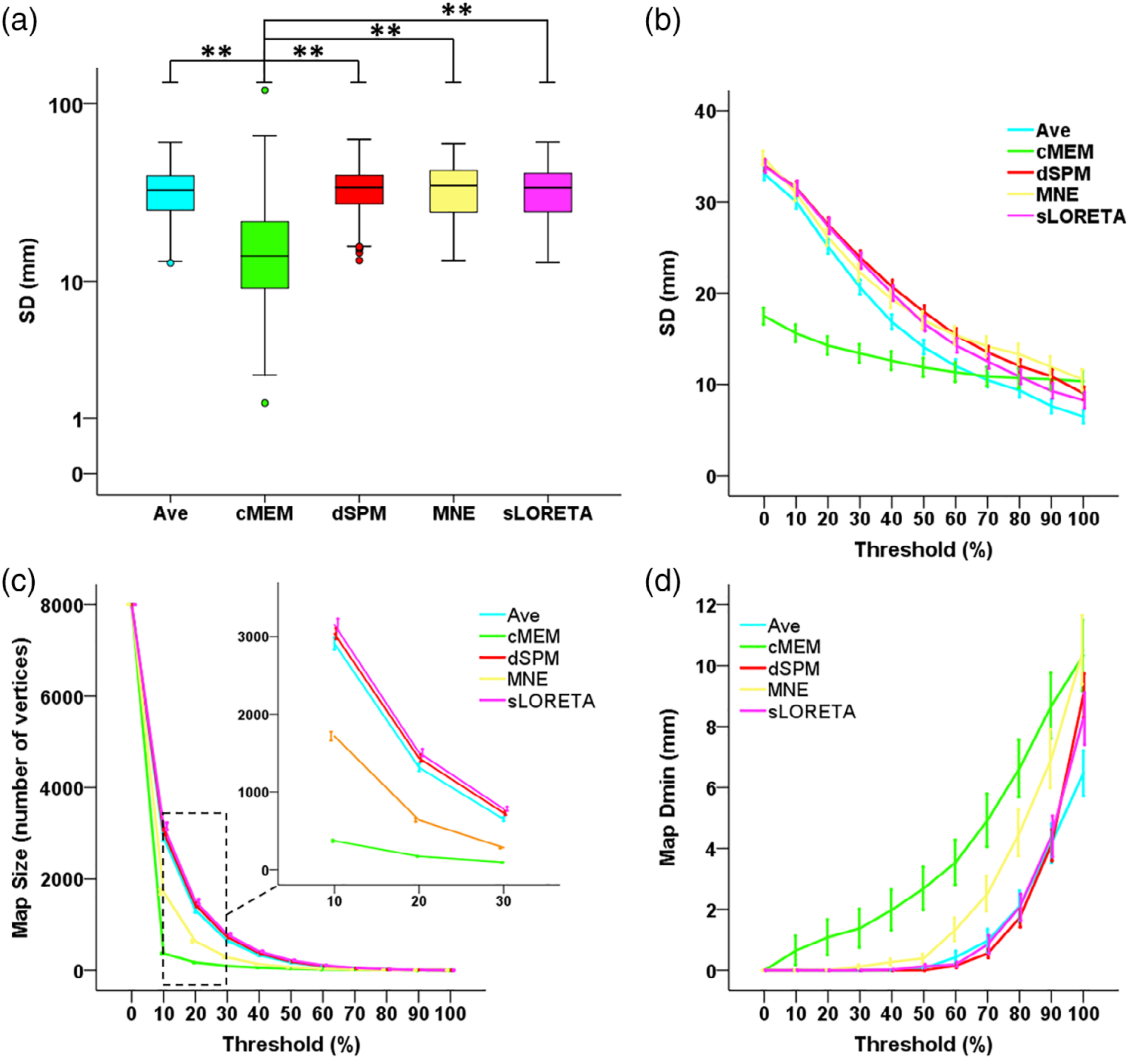
Spatial properties of distributed magnetic source imaging (dMSI) methods. (a) Spatial dispersion (SD) for all methods, computed without applying any threshold. SD was significantly lower for coherent maximum entropy on the mean (cMEM) when compared to every other method. The boxplot depicts median and quartiles. * denotes *p* < .05, ** denotes *p* < .001. (b) SD expressed as function of the threshold value. As the source maximum was typically found in the vicinity of the focus, increasing the threshold reduced the amount of spurious activity for all methods (lower SD), and especially for Minimum Norm Estimate (MNE), dynamic statistical parametric mapping (dSPM), standardized low‐resolution electromagnetic tomography (sLORETA), and Ave. The SD curve of cMEM remained rather “flat” and below other inverse solution techniques, suggesting that cMEM maps (a) result into a very high contrast between the generator and surrounding region, (b) most of the map activity is found within the borders of the focus, and (c) the amount of spurious activity is significantly lower when compared to other techniques. x axis = threshold ranging between 0 and 100%. y axis = SD expressed in mm. The curves denote average values and the variability is expressed as *SE* over “studies.” (c) Size of the source map expressed as function of the threshold value. For a given threshold, the average map size of cMEM was smaller than for other methods. With increasing thresholds, the size of cMEM maps reaches a plateau already at 10–20% threshold (see also zoomed view between 10 and 30% threshold indicated by the dashed box). The other methods exhibited a more regular decrease in extent of the map with increasing threshold, suggesting mainly the influence of the noise level rather than sensitivity to the underlying spatial extent. In other words, within a large range of threshold values, we observed very little influence on the size of the source map for cMEM. x axis = threshold ranging between 0 and 100%. y axis = size of the generator expressed as number of active vertices. y scale ranges between 1 (0% threshold—the entire cortical surface of 8,000 vertices is considered as active) and 8,000 (100% threshold, only the vertex exhibiting maximum amplitude is active). The curves denote average values and the variability are expressed as *SE* over “studies.” (d) Distance from the epilepsy focus (Map Dmin) as function of the threshold. For a given threshold, the average distances of cMEM and minimum norm estimate (MNE) from the focus were larger when compared to the other methods that were more likely to overestimate the size of the generator, especially for lower thresholds. x axis = threshold ranging between 0 and 100%. y axis = distance between the thresholded dMSI map and the focus expressed in mm. The curves denote average values and the variability is expressed as *SE* over “studies”

SD of cortical maps without threshold was significantly different across methods (Friedman's test Chi‐square = 375.386, *df* = 4, *p* < .001). More specifically, cMEM had a significantly lower (i.e., better) SD than all other methods (*p* < .001 consistently) (Figure 3a). Figure 3b illustrates the average SD (±*SE*) across studies as function of the threshold. The GEE revealed a significant difference across inverse solutions (Wald Chi‐square = 178.880, *df* = 4, *p* < .001), an expected main effect of the threshold (Wald Chi‐square = 572.868, *df* = 2, *p* < .001) and a significant interaction (Wald Chi‐square = 288.545, *df* = 8, *p* < .001). The curve profile of cMEM SD was remarkably and significantly lower than all the other inverse solutions for a wide range of threshold values: for threshold = 30% cMEM SD was significantly lower than all other methods (*p* < .001, consistently), for threshold = 60% cMEM SD remained significantly lower than dSPM and MNE (*p* = .011 and *p* = .038, respectively), whereas for threshold = 90%, there was no significant difference between cMEM and other inverse solutions (*p* > .200 consistently). The entire set of Bonferroni‐corrected pairwise comparisons is reported in Supplementary Table S1.

Figure 3c illustrates the average Map size (±*SE*) across studies as function of the threshold. The GEE revealed a significant difference across inverse solutions (Wald Chi‐square = 1,019.417; *df* = 4; *p* < .001), an expected main effect of the threshold (Wald Chi‐square = 1,108.096; *df* = 2; *p* < .001) and a significant interaction (Wald Chi‐square = 1,304.067; *df* = 8; *p* < .001). No significant difference was found between the size of dSPM and sLORETA maps regardless of the threshold value (*p* > .05, consistently). All other methods typically showed a significant difference in size, although with different degree. In particular, for a threshold value ranging between 30 and 60%, cMEM maps were significantly smaller than all other inverse solutions (*p* < .001, consistently, mean difference across methods ranging between 188 and 636 vertices for threshold = 30%).

Figure 3d illustrates the average Map_Dmin (±*SE*) across studies as function of the threshold. The GEE revealed a significant difference across inverse solutions (Wald Chi‐square = 34.320, *df* = 4, *p* < .001), an expected main effect of the threshold (Wald Chi‐square = 99.438, *df* = 2, *p* < .001) and a significant interaction (Wald Chi‐square = 61.900, *df* = 8, *p* < .001). Indeed, while no significant differences across inverse solutions techniques were found for threshold = 30% (*p* > .200, consistently), cMEM Map_Dmin was significantly higher than Ave (*p* = .002), dSPM and sLORETA (*p* < .001 consistently) for threshold = 60%.

**Figure 4:**
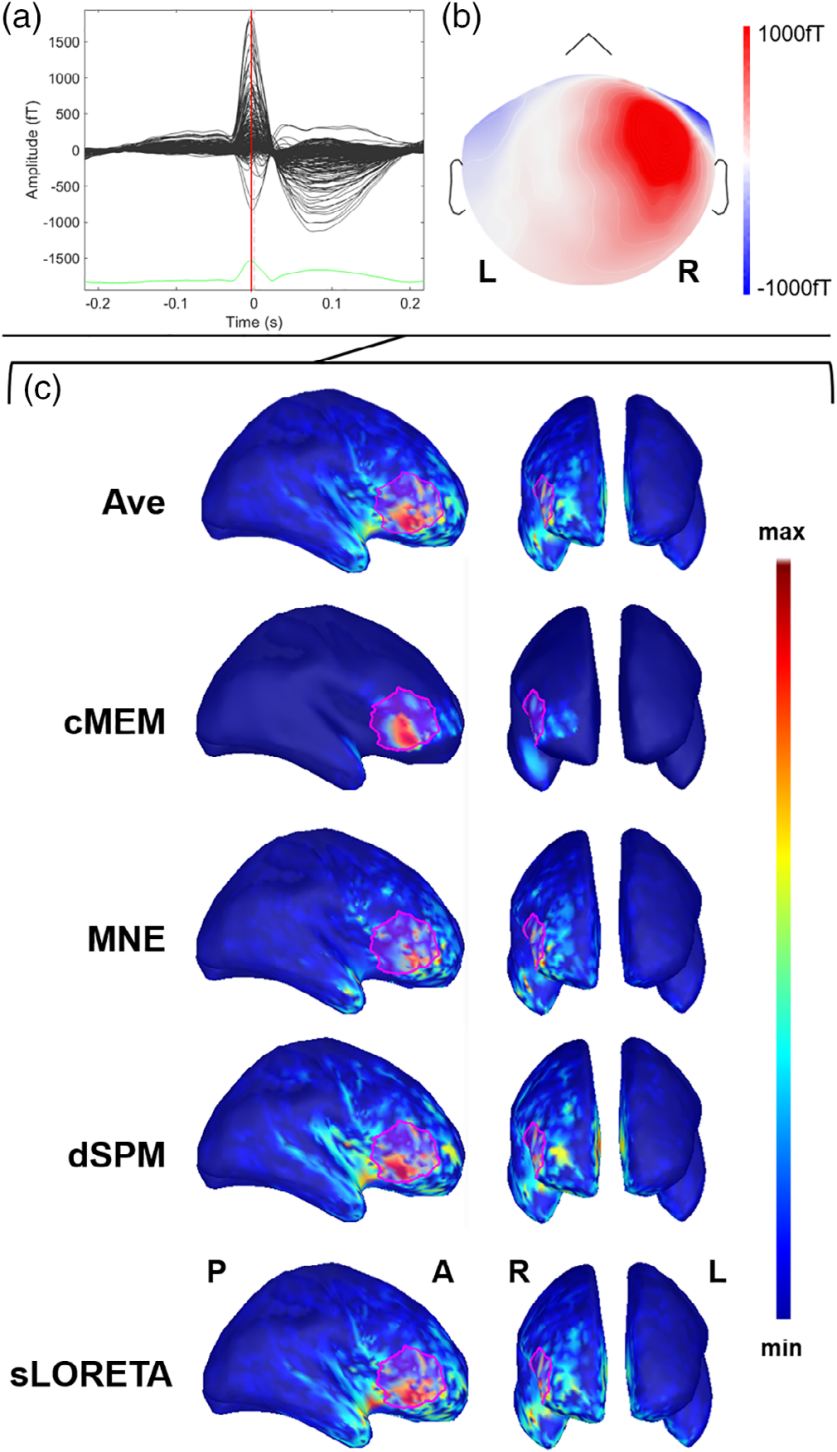
Example of patient with focal epilepsy originating from the right frontal operculum (Patient 11). (a) The average interictal epileptiform discharge (IED). Source imaging is considered at its peak marked by the vertical red line. (b) The magnetic topographical distribution at the peak of the IED. (c) The nonthresholded distributed magnetic source imaging (dMSI) maps for all methods. Cortical surface has been inflated to improve its visualization. The reconstruction of the epileptic focus based on clinical information is depicted in magenta. All the dMSI methods provide a good localization of the epilepsy focus. Coherent maximum entropy on the mean (cMEM) map shows high contrast, with the maximum centered in the right frontal operculum and very little activity spreading outside the epileptic focus. Standardized low‐resolution electromagnetic tomography (sLORETA) shows a higher amount of activity outside the epileptic focus and some strong localization in the anterior insula. Dynamic statistical parametric mapping (dSPM) map shows very similar features to sLORETA. Minimum norm estimate (MNE) retrieved a less blurred map when compared to sLORETA and dSPM, with a maximum in the right frontal operculum, but also some activity spread over the right frontal and temporal pole. Ave shows a good localization, with less spatial spreading as compared to sLORETA, dSPM, and MNE

**Figure 5:**
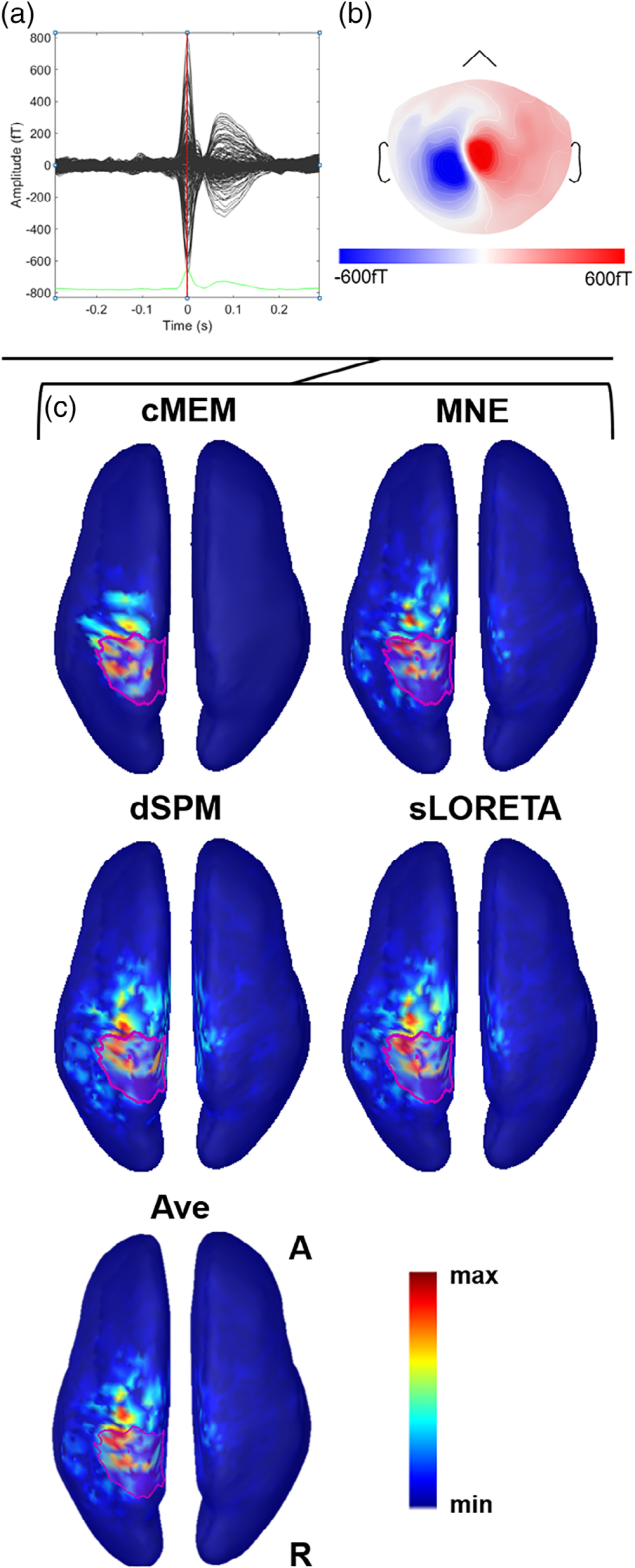
Example of patient with focal epilepsy originating from the left postcentral gyrus (Patient 26). (a) The average interictal epileptiform discharge (IED). Source imaging is considered at its peak marked by the vertical red line. (b) The magnetic topographical distribution at the peak of the IED. (c) The nonthresholded distributed magnetic source imaging (dMSI) maps. Cortical surface has been inflated to improve its visualization. The reconstruction of the epileptic focus based on clinical information is depicted in magenta. All dMSI methods provide a good localization of the epilepsy focus. Coherent maximum entropy on the mean (cMEM) map shows high contrast, with the maximal activity all included in the region of the epileptic focus. Standardized low‐resolution electromagnetic tomography (sLORETA) and dynamic statistical parametric mapping (dSPM) show a very large map, with high activity in the left fronto‐central‐parietal regions. Minimum norm estimate (MNE) retrieved a less distributed map, yet largely displayed outside the epilepsy focus. Also in this case, cMEM shows a very good performance, balancing localization accuracy, and activity spreading outside the epilepsy focus

**Figure 6:**
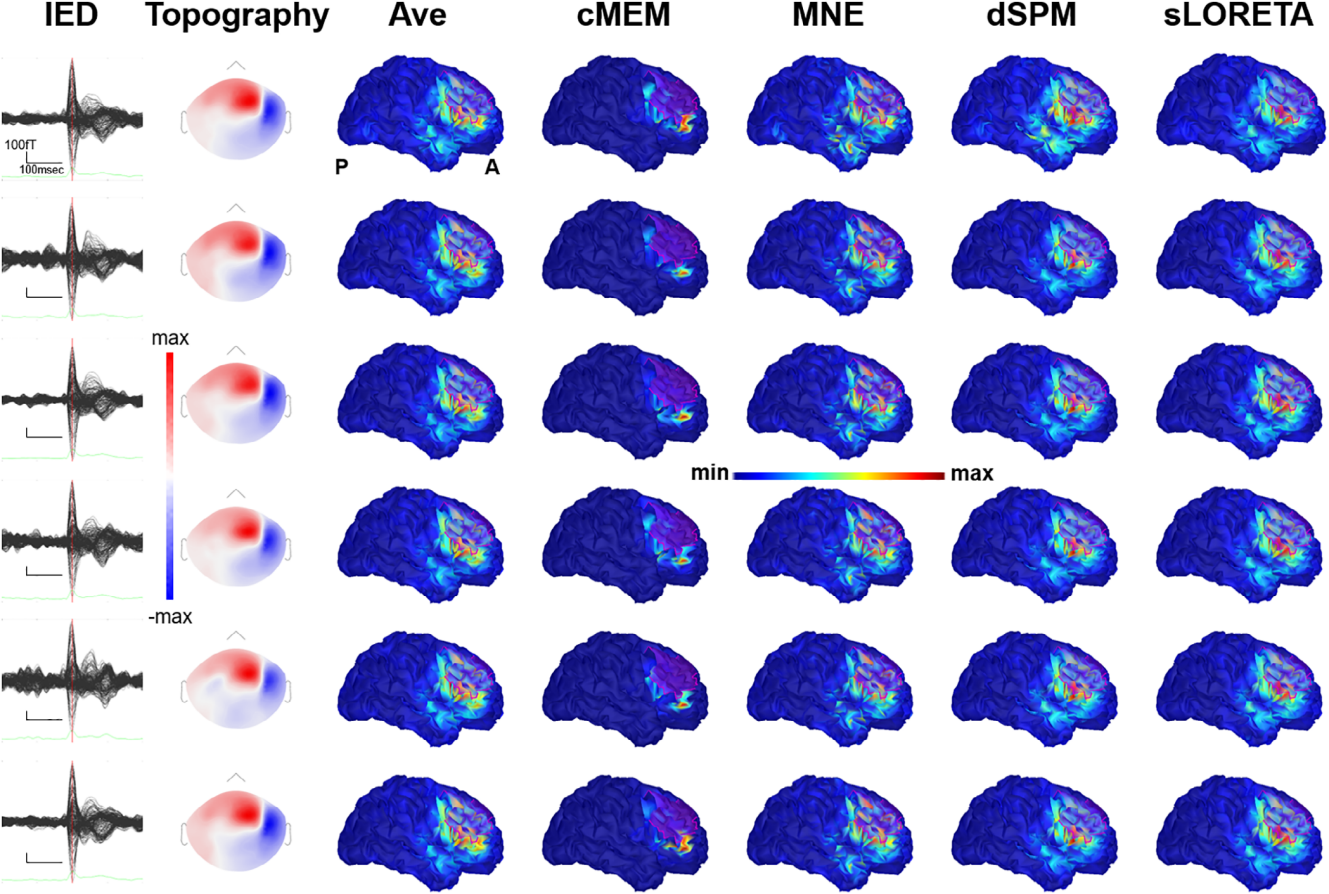
Illustrative patient with focal seizures originating from the right frontal cortex (Patient 14). Each row corresponds to a study. From left to right, interictal epileptiform discharges (IEDs) average, topography at the peak, unthresholded source localization results obtained with Ave, coherent maximum entropy on the mean (cMEM), minimum norm estimate (MNE), dynamic statistical parametric mapping (dSPM), and standardized low‐resolution electromagnetic tomography (sLORETA). Source localization was performed at the peak. The reconstruction of the presumed epileptic focus is depicted in magenta. This example shows that all distributed magnetic source imaging (dMSI) methods provide a good localization of the epilepsy focus. There is good reproducibility, but in this specific case MNE, dSPM, sLORETA perform slightly better than cMEM (see supplementary material for details on the distance from the focus and spatial dispersion (SD))

**Figure 7:**
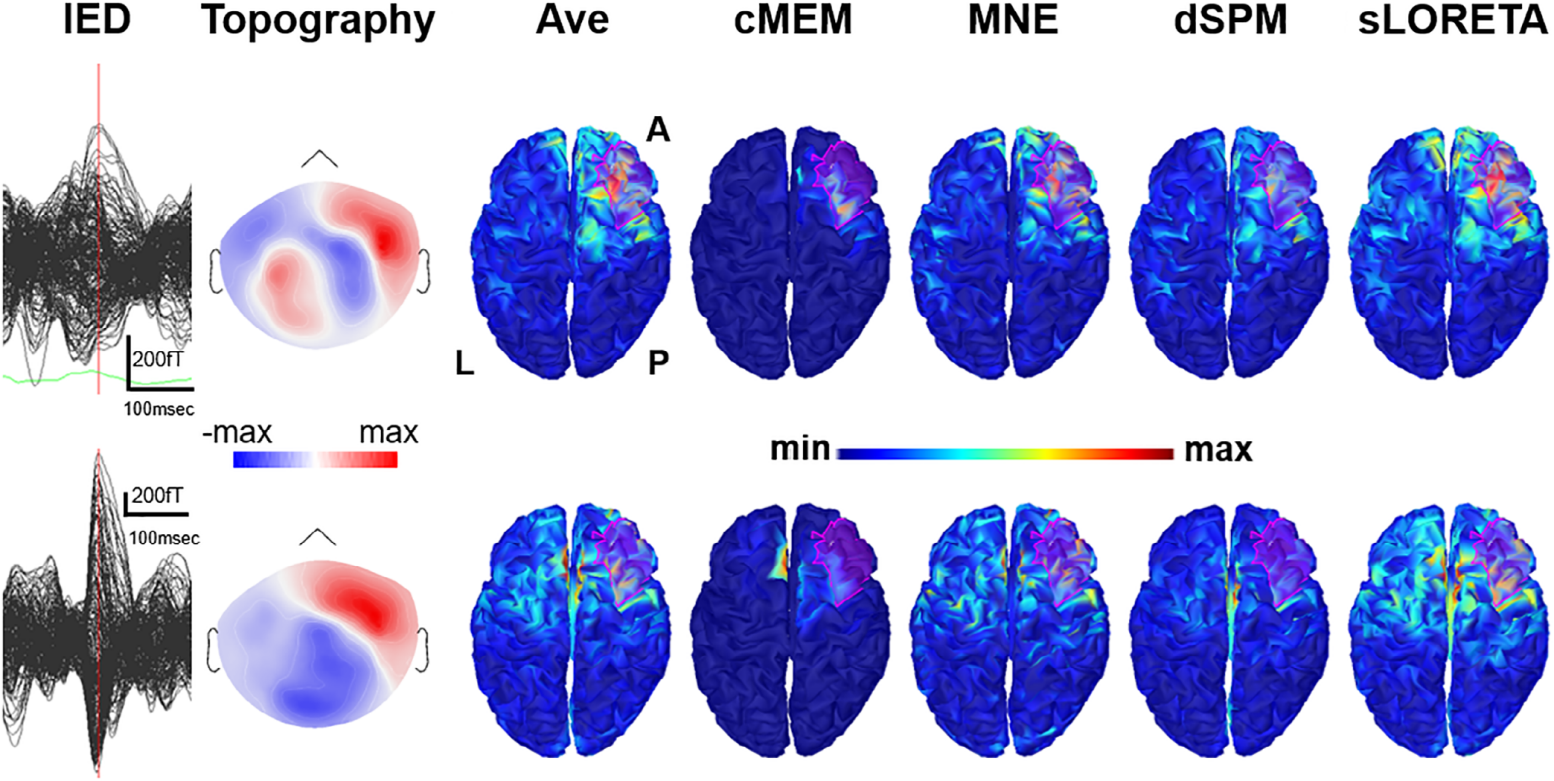
Illustrative patient with focal seizures originating from the right frontal parasagittal cortex (Patient 18). The first row corresponds to Study 1, the second row corresponds to Study 3 (see supplementary material for further details). From left to right, interictal epileptiform discharges (IEDs) average, topography at the peak, unthresholded source localization results obtained with Ave, coherent maximum entropy on the mean (cMEM), minimum norm estimate (MNE), dynamic statistical parametric mapping (dSPM), and standardized low‐resolution electromagnetic tomography (sLORETA). Source localization was performed at the peak. The example shows that distributed magnetic source imaging (dMSI) is sometimes able to reconstruct recover an accurate source also when the signal to noise ratio is not ideal and the topographical distribution is complex (first raw). Conversely, source localization might be inaccurate, at times with the maximum localized in the opposite hemisphere even when the signal to noise ratio is high and the topographical distribution is dipolar (lower row). This often occurs when the source is close to the midline

These results can be appreciated at individual patient level, as illustrated in Figures 4, 5, 6, 7.

## 4 DISCUSSION AND CONCLUSION

Several studies have demonstrated that dMSI has a very good accuracy when compared to ECD, with good overlap between iEEG activity and the reconstructed sources (Grova et al., 2016; Kanamori et al., 2013; Tanaka et al., 2009; Tanaka et al., 2018).

We now demonstrate that MNE, dSPM, cMEM, and sLORETA have excellent performance with similar distance between map maximum and the epilepsy focus as a measure of accuracy, as well as similar within‐subject reliability. The subject‐based analysis further confirmed the overall excellent performance of dMSI techniques, with a median Dmin of 0 mm for all methods suggesting that source localizations of data from the same patients were often providing maps with maximum activity localized inside the epileptic focus.

It is important to notice that in clinical settings, there is no a priori knowledge of the true location and extension of the focus. Different inverse solutions applied to the same data can provide different results. In this study we demonstrated that a simple average of maps from different inverse solutions improves significantly the localization accuracy, as compared to any other technique alone (Figure 1a). The overall effect size of such improvement was however small and in the order of only few millimeters. It is possible that several dMSI approaches localize the maximum of the generator in the vicinity of the focus, with a different degree and direction of error. The average, therefore, may tone down the localization error and take advantage of the localization performance of all the techniques. It would be in principle possible and promising to apply the same strategy to a large number of source imaging approaches, thus capturing different aspects of the same generator. In this context, it would be important to combine some of the more standard localization techniques investigated here to techniques specifically developed to be more sensitive to the underlying extent of the generators (Birot, Albera, Wendling, & Merlet, 2011; Chowdhury et al., 2016; Chowdhury et al., 2018; Sohrabpour et al., 2016). Taking benefit from several source localization methods in the context of epilepsy has been suggested in (de Gooijer‐van de Groep et al., 2013), whereas Trujillo‐Barreto et al. proposed an interesting theoretical framework to combine several source localization results through Bayesian model averaging (Trujillo‐Barreto, Aubert‐Vazquez, & Valdes‐Sosa, 2004). Averaging multiple dMSI solutions is certainly not the only possible approach. Future studies will need to address how to handle the validity and reliability of the results produced by different methods applied on the same data and how to integrate this information for the benefit of the clinical assessment.

Our study focused on the spatial properties of the maps generated with different dMSI approaches. One of the best advantages of dMSI is its ability to provide an extended generator. Not all inverse solution techniques are able to recover the spatial extent of the generator: MNE, dSPM and sLORETA may reach a good accuracy in localizing the peak of the source, but typically provide large maps, often biased, especially for deep generators. (Chowdhury et al., 2013; Chowdhury et al., 2016; Ding, 2009; Lin et al., 2006). For instance, Ding has demonstrated that the minimum norm model fails to recover the continuous cortical distribution of extended sources (Ding, 2009).

In agreement with our previous report (Heers et al., 2016), cMEM was the inverse solution with the lowest SD outside the epilepsy focus. As the source maximum was typically found in the vicinity of the focus, increasing the threshold reduced the amount of spurious activity for all methods (lower SD), and especially for MNE, dSPM, sLORETA, and Ave. The SD curve of cMEM remained rather “flat” and below other inverse solution techniques (Figure 3b), cMEM SD was remarkably and significantly lower than all the other inverse solutions for a wide range of threshold values (threshold = 30% lower than all other methods *p* < .001 consistently, threshold = 60% lower than dSPM (*p* = .011) and MNE [*p* = .038]). For higher threshold values (i.e., 90%), the map activity became highly centered around the peak value, and there were no more significant differences between cMEM and other methods. Overall, our results suggest that for cMEM: (a) there is a very high contrast between the generator and surrounding region, (b) most of the map activity is found within the borders of the focus, and (c) the amount of spurious activity is significantly lower when compared to other techniques. This finding was recently confirmed in a systematic investigation of cMEM's performance in localizing the source of somatosensory evoked potentials and in realistic simulations (Hedrich et al., 2017).

The investigation of the spatial properties also revealed that for a given threshold the average size of the generator was smaller for cMEM when compared to MNE, sLORETA, dSPM, and *Ave*. The purpose of the thresholding was not to find an objective manner of estimating the underlying spatial extent of the generator, yet we considered this approach to further clarify the spatial properties of source imaging maps. The relationship between the threshold of the generator and the average size of the source quickly reached a plateau for cMEM (at about 10–20% threshold when compared to the local maximum amplitude). In addition to the results on SD, this finding confirms that the high contrast cMEM maps are only slightly affected by thresholding: a threshold ranging between 30 to 60% of the local maximum amplitude will leave the map largely unchanged (Figures 4 and 5) and significantly smaller than any other inverse solutions (p < 0.001, consistently). Overall, the relationship between threshold and distance from the focus suggests that the map size and its distance from the epilepsy focus strongly depend on the threshold applied and therefore on the underlying noise level. Applying a method that is minimally affected by thresholding avoids an additional step that is user‐dependent and that could negatively impact the results. Whereas Ave showed a better performance in localizing the map maximum, its spatial properties were similar to those of MNE, sLORETA, and dSPM. This was because Ave was the result of the average of four maps, three of which (MNE, sLORETA, and dSPM) showing significant degree of distant spurious activity (Figures 6 and 7).

The appropriate threshold of source imaging maps strongly depends on the specific source imaging approach and an unambiguous solution to this problem has not yet been found (Becker et al., 2014; Becker et al., 2017; Chowdhury et al., 2016; Jung et al., 2013; Sohrabpour et al., 2016; Zhu et al., 2014). In the case of the cMEM algorithm, the thresholding issue is largely overcome by the inverse solution itself. cMEM is able to switch off some cortical parcels that do not contribute to the inverse solution, while preserving the ability to create a contrast of current intensities within the remaining active parcels. This allowed us in the past to propose and validate an empirical 30% threshold which allows removing most or almost all the background noise (Heers et al., 2014; Heers et al., 2016; Papadelis et al., 2016; Pellegrino, Hedrich, et al., 2018; von Ellenrieder et al., 2016) (Figures 4 and 5). These findings are in agreement with recent studies, demonstrating that, among the most common dMSI techniques, cMEM recovers the generator with the highest contrast while allowing a careful estimation of the spatial extent (Chowdhury et al., 2013; Chowdhury et al., 2015; Chowdhury et al., 2016; Hedrich et al., 2017; Heers et al., 2014).

The spatial properties described above explain the relationship between distance from the epilepsy focus and map threshold as observed in this study. Although Figure 3c illustrated a better performance profile for MNE, dSPM, sLORETA, and *Ave*, this was largely dependent on the size of the underlying map. Methods that, for the same threshold, provide more widespread maps, will more often encompass the focus. The combined evaluation of the properties of the map maximum and spatial properties of the map indicates the use of all these four methods to be very safe. cMEM outperforms the other techniques with respect to the investigation of spatial properties of the generator, whereas *Ave* slightly outperforms all other techniques with respect to the localization of the map maximum.

Our cohort included neocortical epilepsy patients, for whom MEG source localization is more likely to contribute during presurgical evaluation (Genow et al., 2004; Knowlton et al., 1997; Knowlton et al., 2006; Mamelak, Lopez, Akhtari, & Sutherling, 2002; Mu et al., 2014; Ryvlin et al., 2014; Shiraishi et al., 2001; Stefan et al., 2003; Stefan et al., 2011). In agreement with the experience of other centers, only about 64% (18/28) patients were operated and about 40% were fully seizure free at 1‐year follow‐up (Engel Class Ia) (Table 1) (Jeha et al., 2007; Kwan, 2011; Mosewich et al., 2000; Aydin et al., 2020).

By localizing the generator of IEDs, we assessed the so‐called irritative zone. This is typically performed in clinical practice, and consistently with the idea that it typically overlaps with the seizure onset zone (source of the ictal epileptiform activity) (Pellegrino, Hedrich, et al., 2016) and epileptogenic zone (theoretical concept, and refers to the cortex to resect for achieving seizure freedom) (Rosenow & Luders, 2001) (Bagic et al., 2011; Hari et al., 2018). Here the dMSI performance was tested against the “epileptic focus,” which is clinically assessed in a retrospective way, taking into consideration the available information (postsurgical cavity, invasive EEG, MRI epileptogenic lesions) (Agirre‐Arrizubieta, Huiskamp, Ferrier, van Huffelen, & Leijten, 2009; Chowdhury et al., 2018; Heers et al., 2016; Pellegrino, Hedrich, et al., 2016; Pellegrino, Hedrich, et al., 2018).

Realistic simulations are possibly the best initial approach to quantitatively characterize the properties of inverse solutions and all the methods applied here have been previously carefully evaluated using realistic simulations (Chowdhury et al., 2013; Chowdhury et al., 2016; Grova et al., 2006; Hedrich et al., 2017). Biophysical computational models continue pushing further the level of realism of simulations, proposing advanced EEG/MEG generative models (Chowdhury et al., 2016; Cosandier‐Rimélé, Merlet, Badier, Chauvel, & Wendling, 2008; Garnier, Vidal, & Benali, 2016). Simulations, however, are never truly realistic and validation in a clinical setting is necessary to progressively bring new techniques in clinical practice (Chowdhury et al., 2018; Grova et al., 2016; Heers et al., 2016).

Typically, accuracy of a source localization method is assessed considering the lobar or sublobar concordance with the presumed focus/epileptogenic zone/seizure onset zone (Agirre‐Arrizubieta et al., 2009; Heers et al., 2016; Rampp et al., 2019). Here, we performed a personalized evaluation to further validate clinical utility of the applied methods, proposing quantitative metric going beyond sublobar qualitative judgment.

All source imaging procedures applied in this study were performed with standard/default parameters, being operator‐independent and easily performed with user‐friendly freely available toolboxes (Gramfort et al., 2014; Oostenveld, Fries, Maris, & Schoffelen, 2011; Tadel et al., 2011).

This study demonstrates that all the dMSI techniques that we tested provided excellent localization performance. The average map Ave provided the best localization accuracy, whereas cMEM exhibited the lowest amount of spurious distant activity and was less sensitive to map thresholding as compared to other methods. Taking advantage of the strengths and complementarity of different inverse solution methods might be desirable in clinical setting. dMSI of IEDs remains a clinical procedure which cannot be dealt with separately from complete clinical assessment of the underlying epilepsy and from comprehension of the basis of data modeling (principles, parameters to set, and so on) applied for source imaging.

## CONFLICT OF INTEREST

The authors declare no potential conflict of interest.

## Supporting information


**Appendix** **S1.** Supporting Information.

**Appendix** **S2.** Supporting Information.

**Appendix** **S3.** Supporting Information.

**Appendix** **S4.** Supporting Information.

## Data Availability

Individual results are available as supplementary material. The original raw data supporting the findings of this study are available upon reasonable request to the corresponding authors.
