# Supplementary material for: Accuracy and spatial properties of distributed magnetic source imaging techniques in the investigation of focal epilepsy patients
Source: Hum Brain Mapp. 2020 May 9;41(11):3019–33. doi: 10.1002/hbm.24994 (PMC7336148; doi:10.1002/hbm.24994)
Supplement: Supplementary file 1 — Appendix S1. Supporting Information. [file HBM-41-3019-s001.docx]

| ID | Study | NumberOfIEDs | IED Type | Topography |
| --- | --- | --- | --- | --- |
|  |  |  |  |  |
| 1 | 1 | 17 | Spike followed by slow wave | 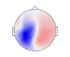 |
| 1 | 2 | 16 | Spike followed by slow wave | 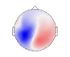 |
| 1 | 3 | 22 | Spike followed by slow wave | 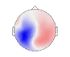 |
| 1 | 4 | 21 | Spike followed by slow wave | 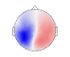 |
| 1 | 5 | 7 | Spike followed by slow wave | 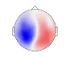 |
| 1 | 6 | 18 | Spike followed by slow wave | 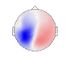 |
| 1 | 7 | 85 | Spike followed by slow wave | 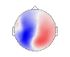 |
| 1 | 8 | 76 | Spike followed by slow wave | 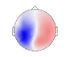 |
| 1 | 9 | 6 | Spike followed by slow wave | 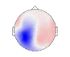 |
| 1 | 10 | 17 | Spike followed by slow wave | 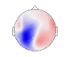 |
| 1 | 11 | 28 | Spike followed by slow wave | 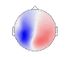 |
|  |  |  |  |  |
| 2 | 1 | 9 | Polyspike followed by slow wave | 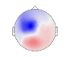 |
|  |  |  |  |  |
| 3 | 1 | 9 | Spike | 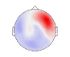 |
| 3 | 2 | 6 | Spike | 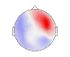 |
| 3 | 3 | 7 | Spike | 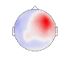 |
| 3 | 4 | 6 | Spike | 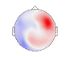 |
| 3 | 5 | 5 | Spike | 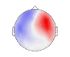 |
|  |  |  |  |  |
| 4 | 1 | 57 | Sharp wave | 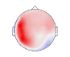 |
| 4 | 2 | 11 | Sharp wave | 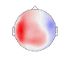 |
| 4 | 3 | 7 | Sharp wave | 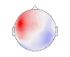 |
| 4 | 4 | 43 | Sharp wave | 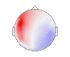 |
| 4 | 5 | 35 | Sharp wave | 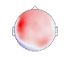 |
| 4 | 6 | 74 | Sharp wave | 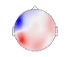 |
| 4 | 7 | 33 | Sharp wave | 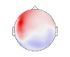 |
| 4 | 8 | 69 | Sharp wave | 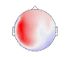 |
| 4 | 9 | 30 | Sharp wave | 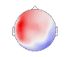 |
|  |  |  |  |  |
| 5 | 1 | 23 | Spike | 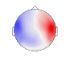 |
| 5 | 2 | 13 | Spike | 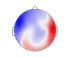 |
| 5 | 3 | 26 | Spike | 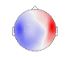 |
| 5 | 4 | 5 | Spike | 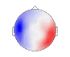 |
|  |  |  |  |  |
| 6 | 1 | 129 | Spike and Wave | 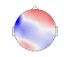 |
| 6 | 2 | 169 | Spike and Wave | 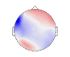 |
| 6 | 3 | 168 | Spike and Wave | 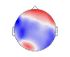 |
| 6 | 4 | 248 | Spike and Wave | 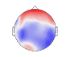 |
| 6 | 5 | 149 | Spike and Wave | 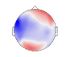 |
|  |  |  |  |  |
| 7 | 1 | 10 | Spike followed by slow wave | 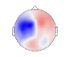 |
| 7 | 2 | 5 | Spike followed by slow wave | 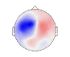 |
| 7 | 3 | 20 | Spike followed by slow wave | 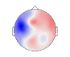 |
| 7 | 4 | 14 | Spike followed by slow wave | 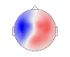 |
| 7 | 5 | 9 | Spike followed by slow wave | 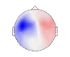 |
| 7 | 6 | 6 | Spike followed by slow wave | 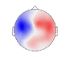 |
|  |  |  |  |  |
| 8 | 1 | 101 | Spike | 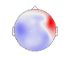 |
| 8 | 2 | 189 | Spike | 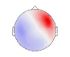 |
| 8 | 3 | 26 | Spike | 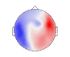 |
| 8 | 4 | 22 | Spike | 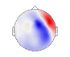 |
| 8 | 5 | 11 | Spike | 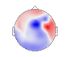 |
| 8 | 6 | 22 | Spike | 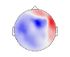 |
|  |  |  |  |  |
| 9 | 1 | 12 | Spike | 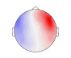 |
| 9 | 2 | 9 | Spike | 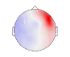 |
| 9 | 3 | 10 | Spike | 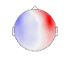 |
| 9 | 4 | 13 | Spike | 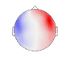 |
| 9 | 5 | 7 | Spike | 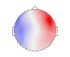 |
| 9 | 6 | 8 | Spike | 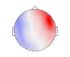 |
| 9 | 7 | 5 | Spike | 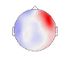 |
| 9 | 8 | 8 | Spike | 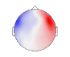 |
|  |  |  |  |  |
| 10 | 1 | 6 | Spike followed by slow wave | 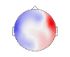 |
| 10 | 2 | 8 | Spike followed by slow wave | 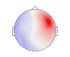 |
| 10 | 3 | 26 | Spike followed by slow wave | 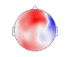 |
| 10 | 4 | 14 | Spike followed by slow wave | 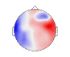 |
| 10 | 5 | 14 | Spike followed by slow wave | 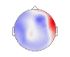 |
| 10 | 6 | 9 | Spike followed by slow wave | 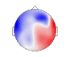 |
| 10 | 7 | 21 | Spike followed by slow wave | 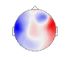 |
| 10 | 8 | 15 | Spike followed by slow wave | 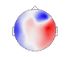 |
| 10 | 9 | 7 | Spike followed by slow wave | 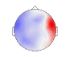 |
|  |  |  |  |  |
| 11 | 1 | 287 | Spike followed by slow wave | 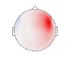 |
| 11 | 2 | 275 | Spike followed by slow wave | 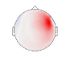 |
| 11 | 3 | 251 | Spike followed by slow wave | 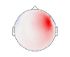 |
| 11 | 4 | 236 | Spike followed by slow wave | 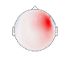 |
| 11 | 5 | 204 | Spike followed by slow wave | 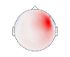 |
| 11 | 6 | 201 | Spike followed by slow wave | 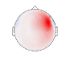 |
|  |  |  |  |  |
| 12 | 1 | 18 | Sharp wave | 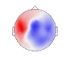 |
| 12 | 2 | 36 | Sharp wave | 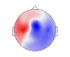 |
| 12 | 3 | 16 | Sharp wave | 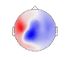 |
| 12 | 4 | 5 | Sharp wave | 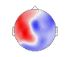 |
|  |  |  |  |  |
| 13 | 1 | 10 | Sharp wave | 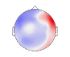 |
| 13 | 2 | 12 | Sharp wave | 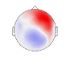 |
| 13 | 3 | 14 | Sharp wave | 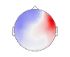 |
| 13 | 4 | 5 | Sharp wave | 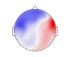 |
| 13 | 5 | 21 | Sharp wave | 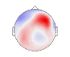 |
| 13 | 6 | 35 | Sharp wave | 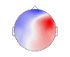 |
| 13 | 7 | 19 | Sharp wave | 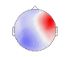 |
| 13 | 8 | 34 | Sharp wave | 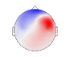 |
| 13 | 9 | 42 | Sharp wave | 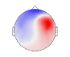 |
| 13 | 10 | 17 | Sharp wave | 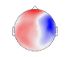 |
| 13 | 11 | 31 | Sharp wave | 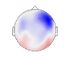 |
| 13 | 12 | 6 | Sharp wave | 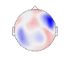 |
| 13 | 13 | 25 | Sharp wave | 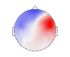 |
| 13 | 14 | 43 | Sharp wave | 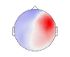 |
| 13 | 15 | 6 | Sharp wave | 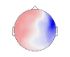 |
|  |  |  |  |  |
| 14 | 1 | 13 | Sharp wave | 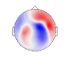 |
| 14 | 2 | 11 | Sharp wave | 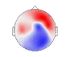 |
| 14 | 3 | 5 | Sharp wave | 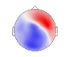 |
|  |  |  |  |  |
| 15 | 1 | 117 | Spike and Wave | 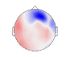 |
| 15 | 2 | 77 | Spike and Wave | 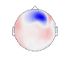 |
| 15 | 3 | 43 | Spike and Wave | 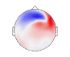 |
| 15 | 4 | 79 | Spike and Wave | 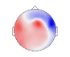 |
| 15 | 5 | 79 | Spike and Wave | 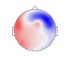 |
| 15 | 6 | 89 | Spike and Wave | 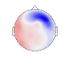 |
| 15 | 7 | 70 | Spike and Wave | 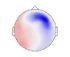 |
| 15 | 8 | 74 | Spike and Wave | 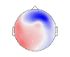 |
| 15 | 9 | 50 | Spike and Wave |  |
|  |  |  |  |  |
| 16 | 1 | 9 | Spike |  |
| 16 | 2 | 5 | Spike |  |
|  |  |  |  |  |
| 17 | 1 | 5 | Sharp Wave |  |
|  |  |  |  |  |
| 18 | 1 | 74 | Spike followed by slow wave |  |
| 18 | 2 | 15 | Spike followed by slow wave |  |
| 18 | 3 | 65 | Spike followed by slow wave |  |
| 18 | 4 | 18 | Spike followed by slow wave |  |
| 18 | 5 | 9 | Spike followed by slow wave |  |
| 18 | 6 | 37 | Spike followed by slow wave |  |
|  |  |  |  |  |
| 19 | 1 | 49 | Spike followed by slow wave |  |
| 19 | 2 | 36 | Spike followed by slow wave |  |
| 19 | 3 | 91 | Spike followed by slow wave |  |
| 19 | 4 | 28 | Spike followed by slow wave |  |
| 19 | 5 | 111 | Spike followed by slow wave |  |
| 19 | 6 | 23 | Spike followed by slow wave |  |
| 19 | 7 | 121 | Spike followed by slow wave |  |
| 19 | 8 | 87 | Spike followed by slow wave |  |
| 19 | 9 | 211 | Spike followed by slow wave |  |
| 19 | 10 | 407 | Spike followed by slow wave |  |
| 19 | 11 | 303 | Spike followed by slow wave |  |
| 19 | 12 | 267 | Spike followed by slow wave |  |
| 19 | 13 | 302 | Spike followed by slow wave |  |
| 19 | 14 | 57 | Spike followed by slow wave |  |
| 19 | 15 | 47 | Spike followed by slow wave |  |
|  |  |  |  |  |
| 20 | 1 | 6 | Sharp Wave |  |
| 20 | 2 | 40 | Sharp Wave |  |
| 20 | 3 | 17 | Sharp Wave |  |
| 20 | 4 | 31 | Sharp Wave |  |
| 20 | 5 | 89 | Sharp Wave |  |
| 20 | 6 | 37 | Sharp Wave |  |
|  |  |  |  |  |
| 21 | 1 | 33 | Spike |  |
| 21 | 2 | 60 | Spike |  |
| 21 | 3 | 33 | Spike |  |
| 21 | 4 | 32 | Spike |  |
| 21 | 5 | 54 | Spike |  |
| 21 | 6 | 26 | Spike |  |
| 21 | 7 | 63 | Spike |  |
| 21 | 8 | 38 | Spike |  |
| 21 | 9 | 36 | Spike |  |
| 21 | 10 | 19 | Spike |  |
| 21 | 11 | 13 | Spike |  |
| 21 | 12 | 55 | Spike |  |
| 21 | 13 | 31 | Spike |  |
| 21 | 14 | 44 | Spike |  |
| 21 | 15 | 35 | Spike |  |
| 21 | 16 | 36 | Spike |  |
| 21 | 17 | 38 | Spike |  |
| 21 | 18 | 44 | Spike |  |
|  |  |  |  |  |
| 22 | 1 | 5 | Sharp wave |  |
| 22 | 2 | 5 | Sharp wave |  |
| 22 | 3 | 6 | Sharp wave |  |
| 22 | 4 | 5 | Sharp wave |  |
|  |  |  |  |  |
| 23 | 1 | 13 | Sharp wave |  |
| 23 | 2 | 14 | Sharp wave |  |
| 23 | 3 | 10 | Sharp wave |  |
| 23 | 4 | 16 | Sharp wave |  |
| 23 | 5 | 5 | Sharp wave |  |
|  |  |  |  |  |
| 24 | 1 | 82 | Spike and wave |  |
| 24 | 2 | 76 | Spike and wave |  |
| 24 | 3 | 67 | Spike and wave |  |
| 24 | 4 | 56 | Spike and wave |  |
| 24 | 5 | 35 | Spike and wave |  |
| 24 | 6 | 23 | Spike and wave |  |
| 24 | 7 | 64 | Spike and wave |  |
| 24 | 8 | 71 | Spike and wave |  |
| 24 | 9 | 69 | Spike and wave |  |
| 24 | 10 | 68 | Spike and wave |  |
|  |  |  |  |  |
| 25 | 1 | 24 | Spike |  |
| 25 | 2 | 17 | Spike |  |
| 25 | 3 | 13 | Spike |  |
| 25 | 4 | 5 | Spike |  |
| 25 | 5 | 18 | Spike |  |
| 25 | 6 | 20 | Spike |  |
| 25 | 7 | 13 | Spike |  |
| 25 | 8 | 12 | Spike |  |
| 25 | 9 | 6 | Spike |  |
|  |  |  |  |  |
| 26 | 1 | 418 | Spike |  |
| 26 | 2 | 306 | Spike |  |
| 26 | 3 | 273 | Spike |  |
| 26 | 4 | 42 | Spike |  |
| 26 | 5 | 20 | Spike |  |
| 26 | 6 | 69 | Spike |  |
| 26 | 7 | 40 | Spike |  |
| 26 | 8 | 11 | Spike |  |
| 26 | 9 | 11 | Spike |  |
|  |  |  |  |  |
| 27 | 1 | 5 | Sharp Wave |  |
| 27 | 2 | 28 | Sharp Wave |  |
| 27 | 3 | 12 | Sharp Wave |  |
| 27 | 4 | 27 | Sharp Wave |  |
| 27 | 5 | 21 | Sharp Wave |  |
| 27 | 6 | 31 | Sharp Wave |  |
| 27 | 7 | 9 | Sharp Wave |  |
| 27 | 8 | 73 | Sharp Wave |  |
| 27 | 9 | 42 | Sharp Wave |  |
| 27 | 10 | 38 | Sharp Wave |  |
| 27 | 11 | 17 | Sharp Wave |  |
| 27 | 12 | 9 | Sharp Wave |  |
| 27 | 13 | 5 | Sharp Wave |  |
|  |  |  |  |  |
| 28 | 1 | 10 | Sharp Wave |  |
| 28 | 2 | 18 | Sharp Wave |  |
| 28 | 3 | 8 | Sharp Wave |  |
| 28 | 4 | 10 | Sharp Wave |  |
| 28 | 5 | 5 | Sharp Wave |  |
| 28 | 6 | 8 | Sharp Wave |  |
| 28 | 7 | 21 | Sharp Wave |  |
