# Supplementary material for: Accuracy and spatial properties of distributed magnetic source imaging techniques in the investigation of focal epilepsy patients
Source: Hum Brain Mapp. 2020 May 9;41(11):3019–33. doi: 10.1002/hbm.24994 (PMC7336148; doi:10.1002/hbm.24994)
Supplement: Supplementary file 2 — Appendix S2. Supporting Information. [file HBM-41-3019-s002.docx]

***Equivalent Current Dipole Analysis.***

The material and methods for the application of the Equivalen Current Dipole technique have been carefully described in (Pellegrino et al., 2018), in agreement with the American Clinical Magnetoencephalography Society (Bagic, Knowlton, Rose, Ebersole, & Acmegs Clinical Practice Guideline Committee, 2011). Briefly, single dipoles were fitted considering all MEG channels and without any a priori definition of the initialization point. A diagonal noise‐covariance was modeled from a 1s baseline without any visually identified IEDs. The fitting was restricted to a small time-window of 4 msec around the IED peak. The solution was not restricted to the cortical surface and the forward model was computed according to the overlapping spheres method approach. In the table below we report the results relative to each study. Quantitative analyses included: (a) the minimum Euclidean distance (Dmin, expressed in mm) between the ECD and the focus, (b) the minimum Euclidean distance between the dipole projection to the closest point of the cortical surface and the focus (Dmin_Proj); and (c) the goodness of fit (Bagic et al., 2011).

| Patient ID | Study | Dmin | Dmin_Proj | GOF |
| --- | --- | --- | --- | --- |
| 1 | 1 | 3.49 | 0.00 | 0.71 |
| 1 | 2 | 1.24 | 0.00 | 0.88 |
| 1 | 3 | 4.14 | 0.00 | 0.81 |
| 1 | 4 | 5.81 | 5.63 | 0.90 |
| 1 | 5 | 6.57 | 5.28 | 0.91 |
| 1 | 6 | 3.29 | 0.00 | 0.86 |
| 1 | 7 | 3.26 | 0.00 | 0.86 |
| 1 | 8 | 2.68 | 3.29 | 0.87 |
| 1 | 9 | 2.04 | 0.00 | 0.68 |
| 1 | 10 | 1.48 | 0.00 | 0.70 |
| 1 | 11 | 1.09 | 0.00 | 0.91 |
| 2 | 1 | 4.78 | 0.00 | 0.89 |
| 3 | 1 | 50.77 | 51.40 | 0.74 |
| 3 | 2 | 16.25 | 14.42 | 0.65 |
| 3 | 3 | 1.85 | 0.00 | 0.83 |
| 3 | 4 | 7.61 | 8.72 | 0.72 |
| 3 | 5 | 2.15 | 0.00 | 0.91 |
| 4 | 1 | 8.81 | 0.00 | 0.73 |
| 4 | 2 | 2.28 | 0.00 | 0.80 |
| 4 | 3 | 2.62 | 0.00 | 0.89 |
| 4 | 4 | 1.86 | 0.00 | 0.80 |
| 4 | 5 | 4.30 | 0.00 | 0.72 |
| 4 | 6 | 6.77 | 0.00 | 0.84 |
| 4 | 7 | 3.53 | 0.00 | 0.79 |
| 4 | 8 | 2.23 | 0.00 | 0.84 |
| 4 | 9 | 3.18 | 0.00 | 0.83 |
| 5 | 1 | 22.28 | 23.50 | 0.85 |
| 5 | 2 | 53.09 | 45.14 | 0.80 |
| 5 | 3 | 43.73 | 38.54 | 0.90 |
| 5 | 4 | 45.83 | 45.53 | 0.88 |
| 6 | 1 | 23.04 | 0.00 | 0.60 |
| 6 | 2 | 22.78 | 0.00 | 0.61 |
| 6 | 3 | 6.23 | 0.00 | 0.36 |
| 6 | 4 | 49.27 | 50.68 | 0.47 |
| 6 | 5 | 50.47 | 47.05 | 0.53 |
| 7 | 1 | 2.24 | 0.00 | 0.79 |
| 7 | 2 | 6.60 | 7.10 | 0.78 |
| 7 | 3 | 35.59 | 33.26 | 0.68 |
| 7 | 4 | 29.02 | 25.53 | 0.95 |
| 7 | 5 | 15.86 | 17.98 | 0.83 |
| 7 | 6 | 2.70 | 0.00 | 0.85 |
| 8 | 1 | 59.68 | 58.10 | 0.77 |
| 8 | 2 | 29.53 | 30.87 | 0.77 |
| 8 | 3 | 41.55 | 42.81 | 0.76 |
| 8 | 4 | 13.20 | 12.62 | 0.78 |
| 8 | 5 | 19.56 | 14.75 | 0.43 |
| 8 | 6 | 9.10 | 10.64 | 0.64 |
| 9 | 1 | 2.26 | 0.00 | 0.93 |
| 9 | 2 | 4.06 | 0.00 | 0.80 |
| 9 | 3 | 3.55 | 0.00 | 0.87 |
| 9 | 4 | 3.78 | 0.00 | 0.90 |
| 9 | 5 | 7.15 | 4.07 | 0.87 |
| 9 | 6 | 5.55 | 4.60 | 0.89 |
| 9 | 7 | 1.76 | 0.00 | 0.81 |
| 9 | 8 | 1.80 | 0.00 | 0.91 |
| 10 | 1 | 30.22 | 0.00 | 0.80 |
| 10 | 2 | 10.64 | 0.00 | 0.92 |
| 10 | 3 | 14.23 | 13.75 | 0.77 |
| 10 | 4 | 13.27 | 0.00 | 0.58 |
| 10 | 5 | 1.91 | 0.00 | 0.54 |
| 10 | 6 | 38.89 | 0.00 | 0.65 |
| 10 | 7 | 36.24 | 0.00 | 0.68 |
| 10 | 8 | 1.66 | 0.00 | 0.63 |
| 10 | 9 | 22.52 | 0.00 | 0.75 |
| 11 | 1 | 3.01 | 0.00 | 0.96 |
| 11 | 2 | 3.28 | 0.00 | 0.97 |
| 11 | 3 | 3.89 | 0.00 | 0.97 |
| 11 | 4 | 2.87 | 0.00 | 0.97 |
| 11 | 5 | 3.10 | 0.00 | 0.97 |
| 11 | 6 | 3.18 | 0.00 | 0.97 |
| 12 | 1 | 5.82 | 0.00 | 0.86 |
| 12 | 2 | 2.34 | 0.00 | 0.76 |
| 12 | 3 | 7.87 | 10.47 | 0.82 |
| 12 | 4 | 3.65 | 0.00 | 0.59 |
| 13 | 1 | 15.00 | 14.35 | 0.72 |
| 13 | 2 | 25.52 | 20.72 | 0.69 |
| 13 | 3 | 9.59 | 11.61 | 0.94 |
| 13 | 4 | 8.05 | 8.45 | 0.85 |
| 13 | 5 | 14.53 | 11.47 | 0.75 |
| 13 | 6 | 13.82 | 12.79 | 0.94 |
| 13 | 7 | 3.70 | 0.00 | 0.66 |
| 13 | 8 | 14.49 | 17.43 | 0.94 |
| 13 | 9 | 10.86 | 7.40 | 0.95 |
| 13 | 10 | 11.70 | 11.87 | 0.77 |
| 13 | 11 | 26.50 | 22.04 | 0.81 |
| 13 | 12 | 38.68 | 27.36 | 0.51 |
| 13 | 13 | 12.27 | 14.09 | 0.95 |
| 13 | 14 | 12.38 | 11.37 | 0.91 |
| 13 | 15 | 5.53 | 8.45 | 0.91 |
| 14 | 1 | 1.26 | 0.00 | 0.62 |
| 14 | 2 | 13.87 | 17.29 | 0.66 |
| 14 | 3 | 13.09 | 10.07 | 0.79 |
| 15 | 1 | 4.64 | 0.00 | 0.53 |
| 15 | 2 | 5.27 | 0.00 | 0.68 |
| 15 | 3 | 2.60 | 0.00 | 0.60 |
| 15 | 4 | 2.32 | 0.00 | 0.59 |
| 15 | 5 | 8.98 | 0.00 | 0.64 |
| 15 | 6 | 3.94 | 0.00 | 0.65 |
| 15 | 7 | 2.83 | 0.00 | 0.75 |
| 15 | 8 | 1.99 | 0.00 | 0.58 |
| 15 | 9 | 3.92 | 0.00 | 0.72 |
| 16 | 1 | 10.61 | 9.40 | 0.90 |
| 16 | 2 | 3.92 | 4.31 | 0.87 |
| 17 | 1 | 19.05 | 24.51 | 0.90 |
| 18 | 1 | 6.12 | 0.00 | 0.87 |
| 18 | 2 | 6.33 | 5.89 | 0.86 |
| 18 | 3 | 7.41 | 5.89 | 0.90 |
| 18 | 4 | 7.41 | 5.89 | 0.86 |
| 18 | 5 | 10.68 | 5.89 | 0.78 |
| 18 | 6 | 7.88 | 5.89 | 0.85 |
| 18 | 7 | 4.25 | 4.45 | 0.94 |
| 19 | 1 | 4.15 | 4.45 | 0.96 |
| 19 | 2 | 0.78 | 0.00 | 0.92 |
| 19 | 3 | 5.18 | 5.39 | 0.95 |
| 19 | 4 | 1.29 | 0.00 | 0.92 |
| 19 | 5 | 3.40 | 0.00 | 0.95 |
| 19 | 6 | 2.15 | 0.00 | 0.94 |
| 19 | 7 | 4.13 | 0.00 | 0.92 |
| 19 | 8 | 4.81 | 2.60 | 0.96 |
| 19 | 9 | 5.17 | 0.00 | 0.97 |
| 19 | 10 | 4.60 | 0.00 | 0.96 |
| 19 | 11 | 4.77 | 2.60 | 0.97 |
| 19 | 12 | 4.60 | 0.00 | 0.97 |
| 19 | 13 | 2.95 | 0.00 | 0.95 |
| 19 | 14 | 3.18 | 0.00 | 0.95 |
| 20 | 1 | 6.35 | 0.00 | 0.74 |
| 20 | 2 | 5.11 | 5.55 | 0.92 |
| 20 | 3 | 3.92 | 5.45 | 0.82 |
| 20 | 4 | 2.15 | 0.00 | 0.95 |
| 20 | 5 | 2.44 | 0.00 | 0.97 |
| 20 | 6 | 2.66 | 0.00 | 0.91 |
| 21 | 1 | 1.46 | 0.00 | 0.69 |
| 21 | 2 | 2.67 | 0.00 | 0.79 |
| 21 | 3 | 2.16 | 0.00 | 0.65 |
| 21 | 4 | 3.65 | 0.00 | 0.71 |
| 21 | 5 | 3.95 | 0.00 | 0.78 |
| 21 | 6 | 2.32 | 0.00 | 0.67 |
| 21 | 7 | 3.40 | 0.00 | 0.80 |
| 21 | 8 | 1.86 | 0.00 | 0.76 |
| 21 | 9 | 2.33 | 0.00 | 0.76 |
| 21 | 10 | 1.51 | 0.00 | 0.80 |
| 21 | 11 | 2.78 | 0.00 | 0.72 |
| 21 | 12 | 2.95 | 0.00 | 0.85 |
| 21 | 13 | 3.30 | 0.00 | 0.82 |
| 21 | 14 | 3.78 | 0.00 | 0.75 |
| 21 | 15 | 3.06 | 0.00 | 0.82 |
| 21 | 16 | 3.77 | 0.00 | 0.73 |
| 21 | 17 | 1.29 | 0.00 | 0.82 |
| 21 | 18 | 3.52 | 0.00 | 0.83 |
| 22 | 1 | 1.89 | 0.00 | 0.97 |
| 22 | 2 | 8.93 | 0.00 | 0.85 |
| 22 | 3 | 3.31 | 0.00 | 0.91 |
| 22 | 4 | 4.06 | 0.00 | 0.88 |
| 23 | 1 | 1.31 | 0.00 | 0.86 |
| 23 | 2 | 12.39 | 9.54 | 0.86 |
| 23 | 3 | 6.99 | 5.53 | 0.86 |
| 23 | 4 | 3.80 | 0.00 | 0.76 |
| 23 | 5 | 2.02 | 0.00 | 0.83 |
| 24 | 1 | 2.69 | 0.00 | 0.78 |
| 24 | 2 | 6.92 | 0.00 | 0.64 |
| 24 | 3 | 9.20 | 9.39 | 0.80 |
| 24 | 4 | 1.67 | 0.00 | 0.82 |
| 24 | 5 | 3.06 | 0.00 | 0.80 |
| 24 | 6 | 1.62 | 0.00 | 0.83 |
| 24 | 7 | 3.98 | 0.00 | 0.90 |
| 24 | 8 | 3.12 | 0.00 | 0.83 |
| 24 | 9 | 2.82 | 0.00 | 0.80 |
| 24 | 10 | 3.63 | 0.00 | 0.72 |
| 25 | 1 | 1.61 | 0.00 | 0.94 |
| 25 | 2 | 7.21 | 0.00 | 0.94 |
| 25 | 3 | 10.76 | 0.00 | 0.92 |
| 25 | 4 | 36.18 | 35.87 | 0.68 |
| 25 | 5 | 2.59 | 0.00 | 0.92 |
| 25 | 6 | 2.26 | 0.00 | 0.94 |
| 25 | 7 | 2.43 | 0.00 | 0.92 |
| 25 | 8 | 1.87 | 0.00 | 0.93 |
| 25 | 9 | 7.60 | 5.14 | 0.91 |
| 26 | 1 | 6.08 | 0.00 | 0.93 |
| 26 | 2 | 4.82 | 0.00 | 0.93 |
| 26 | 3 | 5.76 | 0.00 | 0.93 |
| 26 | 4 | 5.33 | 0.00 | 0.90 |
| 26 | 5 | 47.49 | 46.75 | 0.39 |
| 26 | 6 | 7.59 | 0.00 | 0.91 |
| 26 | 7 | 7.27 | 0.00 | 0.88 |
| 26 | 8 | 2.18 | 0.00 | 0.90 |
| 26 | 9 | 30.36 | 29.86 | 0.72 |
| 27 | 1 | 5.55 | 4.79 | 0.56 |
| 27 | 2 | 27.23 | 22.53 | 0.66 |
| 27 | 3 | 5.18 | 5.25 | 0.78 |
| 27 | 4 | 23.77 | 22.25 | 0.72 |
| 27 | 5 | 3.80 | 1.91 | 0.71 |
| 27 | 6 | 27.74 | 22.53 | 0.61 |
| 27 | 7 | 3.59 | 0.00 | 0.87 |
| 27 | 8 | 24.36 | 20.09 | 0.70 |
| 27 | 9 | 8.74 | 9.22 | 0.76 |
| 27 | 10 | 22.19 | 22.25 | 0.85 |
| 27 | 11 | 2.88 | 0.00 | 0.84 |
| 27 | 12 | 14.10 | 7.29 | 0.40 |
| 27 | 13 | 4.42 | 0.00 | 0.86 |
| 28 | 1 | 19.59 | 15.53 | 0.77 |
| 28 | 2 | 38.39 | 36.89 | 0.67 |
| 28 | 3 | 37.82 | 39.86 | 0.42 |
| 28 | 4 | 22.15 | 22.22 | 0.57 |
| 28 | 5 | 33.37 | 33.90 | 0.45 |
| 28 | 6 | 68.28 | 66.51 | 0.51 |
| 28 | 7 | 29.60 | 26.90 | 0.83 |
| **Mean** | | **10.51** | **7.15** | **0.80** |
| **Median** | | **4.70** | **0.00** | **0.82** |
| **Std. Error of Mean** | | **0.90** | **0.90** | **0.01** |
| **Minimum** | | **0.78** | **0.00** | **0.36** |
| **Maximum** | | **68.28** | **66.51** | **0.97** |
| **Range** | | **67.51** | **66.51** | **0.61** |
| **Std. Deviation** | | **12.88** | **12.95** | **0.13** |
