# Supplementary material for: Accuracy and spatial properties of distributed magnetic source imaging techniques in the investigation of focal epilepsy patients
Source: Hum Brain Mapp. 2020 May 9;41(11):3019–33. doi: 10.1002/hbm.24994 (PMC7336148; doi:10.1002/hbm.24994)
Supplement: Supplementary file 3 — Appendix S3. Supporting Information. [file HBM-41-3019-s003.docx]

Patient ID Study Inverse Threshold (%) SD Map_Dmin Size

1 1 Ave 0 32.996 0.000 8002

1 1 Ave 10 30.469 0.000 3439

1 1 Ave 20 23.871 0.000 1621

1 1 Ave 30 19.032 0.000 843

1 1 Ave 40 15.378 0.000 453

1 1 Ave 50 11.587 0.000 242

1 1 Ave 60 8.998 0.000 127

1 1 Ave 70 6.935 0.000 69

1 1 Ave 80 5.048 0.000 27

1 1 Ave 90 2.513 0.000 8

1 1 Ave 100 0.000 0.000 1

1 1 cMEM 0 9.821 0.000 8002

1 1 cMEM 10 6.822 0.000 246

1 1 cMEM 20 5.614 0.000 140

1 1 cMEM 30 5.086 0.000 79

1 1 cMEM 40 4.902 0.000 46

1 1 cMEM 50 3.158 0.000 26

1 1 cMEM 60 0.845 0.000 12

1 1 cMEM 70 0.000 0.000 6

1 1 cMEM 80 0.000 0.000 2

1 1 cMEM 90 0.000 0.000 2

1 1 cMEM 100 0.000 0.000 1

1 1 dSPM 0 31.308 0.000 8002

1 1 dSPM 10 28.209 0.000 2493

1 1 dSPM 20 24.546 0.000 1152

1 1 dSPM 30 21.849 0.000 612

1 1 dSPM 40 19.167 0.000 331

1 1 dSPM 50 17.337 0.000 169

1 1 dSPM 60 14.333 0.000 85

1 1 dSPM 70 12.228 0.000 46

1 1 dSPM 80 10.425 0.000 18

1 1 dSPM 90 7.277 0.000 7

1 1 dSPM 100 0.000 0.000 1

1 1 MNE 0 36.432 0.000 8002

1 1 MNE 10 31.895 0.000 2124

1 1 MNE 20 23.572 0.000 737

1 1 MNE 30 18.321 0.000 343

1 1 MNE 40 15.304 0.000 149

1 1 MNE 50 11.694 0.000 68

1 1 MNE 60 10.319 0.000 28

1 1 MNE 70 12.163 0.000 10

1 1 MNE 80 14.853 14.853 1

1 1 MNE 90 14.853 14.853 1

1 1 MNE 100 14.853 14.853 1

1 1 sLORETA 0 35.717 0.000 8002

1 1 sLORETA 10 33.964 0.000 3920

1 1 sLORETA 20 28.777 0.000 2039

1 1 sLORETA 30 22.867 0.000 1081

1 1 sLORETA 40 19.384 0.000 615

1 1 sLORETA 50 15.339 0.000 311

1 1 sLORETA 60 11.305 0.000 161

1 1 sLORETA 70 9.577 0.000 83

1 1 sLORETA 80 7.254 0.000 26

1 1 sLORETA 90 5.785 0.000 11

1 1 sLORETA 100 0.000 0.000 1

1 2 Ave 0 43.046 0.000 8002

1 2 Ave 10 41.379 0.000 3498

1 2 Ave 20 35.751 0.000 1486

1 2 Ave 30 23.474 0.000 622

1 2 Ave 40 15.133 0.000 271

1 2 Ave 50 11.529 0.000 143

1 2 Ave 60 5.958 0.000 78

1 2 Ave 70 4.896 0.000 39

1 2 Ave 80 3.589 0.000 16

1 2 Ave 90 0.000 0.000 6

1 2 Ave 100 0.000 0.000 1

1 2 cMEM 0 4.416 0.000 8002

1 2 cMEM 10 2.835 0.000 146

1 2 cMEM 20 1.102 0.000 63

1 2 cMEM 30 0.736 0.000 35

1 2 cMEM 40 0.000 0.000 23

1 2 cMEM 50 0.000 0.000 17

1 2 cMEM 60 0.000 0.000 11

1 2 cMEM 70 0.000 0.000 6

1 2 cMEM 80 0.000 0.000 2

1 2 cMEM 90 0.000 0.000 2

1 2 cMEM 100 0.000 0.000 1

1 2 dSPM 0 38.661 0.000 8002

1 2 dSPM 10 35.728 0.000 2680

1 2 dSPM 20 28.539 0.000 1062

1 2 dSPM 30 20.287 0.000 444

1 2 dSPM 40 16.419 0.000 201

1 2 dSPM 50 12.224 0.000 86

1 2 dSPM 60 10.031 0.000 43

1 2 dSPM 70 8.474 0.000 23

1 2 dSPM 80 5.536 0.000 9

1 2 dSPM 90 2.946 0.000 3

1 2 dSPM 100 0.000 0.000 1

1 2 MNE 0 45.457 0.000 8002

1 2 MNE 10 42.603 0.000 2070

1 2 MNE 20 34.090 0.000 625

1 2 MNE 30 23.947 0.000 251

1 2 MNE 40 17.744 0.000 129

1 2 MNE 50 17.638 0.000 59

1 2 MNE 60 14.638 0.000 30

1 2 MNE 70 11.023 0.000 16

1 2 MNE 80 9.979 0.000 7

1 2 MNE 90 10.759 0.000 4

1 2 MNE 100 14.853 14.853 1

1 2 sLORETA 0 47.335 0.000 8002

1 2 sLORETA 10 46.573 0.000 4295

1 2 sLORETA 20 44.132 0.000 2198

1 2 sLORETA 30 39.869 0.000 1125

1 2 sLORETA 40 31.572 0.000 578

1 2 sLORETA 50 18.447 0.000 249

1 2 sLORETA 60 12.849 0.000 128

1 2 sLORETA 70 7.250 0.000 64

1 2 sLORETA 80 6.628 0.000 29

1 2 sLORETA 90 5.379 0.000 8

1 2 sLORETA 100 0.000 0.000 1

1 3 Ave 0 36.595 0.000 8002

1 3 Ave 10 34.122 0.000 3424

1 3 Ave 20 27.804 0.000 1545

1 3 Ave 30 22.146 0.000 730

1 3 Ave 40 17.148 0.000 356

1 3 Ave 50 13.870 0.000 181

1 3 Ave 60 9.946 0.000 91

1 3 Ave 70 6.639 0.000 45

1 3 Ave 80 6.110 0.000 21

1 3 Ave 90 6.014 0.000 8

1 3 Ave 100 0.000 0.000 1

1 3 cMEM 0 8.114 0.000 8002

1 3 cMEM 10 5.177 0.000 210

1 3 cMEM 20 3.706 0.000 110

1 3 cMEM 30 2.810 0.000 58

1 3 cMEM 40 1.616 0.000 31

1 3 cMEM 50 0.000 0.000 17

1 3 cMEM 60 0.000 0.000 10

1 3 cMEM 70 0.000 0.000 6

1 3 cMEM 80 0.000 0.000 2

1 3 cMEM 90 0.000 0.000 2

1 3 cMEM 100 0.000 0.000 1

1 3 dSPM 0 34.295 0.000 8002

1 3 dSPM 10 31.160 0.000 2725

1 3 dSPM 20 26.731 0.000 1247

1 3 dSPM 30 22.712 0.000 622

1 3 dSPM 40 19.926 0.000 314

1 3 dSPM 50 16.272 0.000 155

1 3 dSPM 60 13.635 0.000 67

1 3 dSPM 70 9.862 0.000 30

1 3 dSPM 80 5.735 0.000 8

1 3 dSPM 90 5.911 0.000 3

1 3 dSPM 100 0.000 0.000 1

1 3 MNE 0 39.011 0.000 8002

1 3 MNE 10 35.223 0.000 2041

1 3 MNE 20 27.582 0.000 659

1 3 MNE 30 21.771 0.000 294

1 3 MNE 40 19.214 0.000 138

1 3 MNE 50 18.740 0.000 75

1 3 MNE 60 16.115 0.000 39

1 3 MNE 70 15.201 0.000 23

1 3 MNE 80 14.756 0.000 12

1 3 MNE 90 10.663 0.000 5

1 3 MNE 100 12.847 12.847 1

1 3 sLORETA 0 39.463 0.000 8002

1 3 sLORETA 10 37.847 0.000 3881

1 3 sLORETA 20 32.990 0.000 1950

1 3 sLORETA 30 27.543 0.000 995

1 3 sLORETA 40 21.679 0.000 475

1 3 sLORETA 50 17.330 0.000 238

1 3 sLORETA 60 13.568 0.000 115

1 3 sLORETA 70 10.095 0.000 44

1 3 sLORETA 80 7.909 0.000 18

1 3 sLORETA 90 12.535 11.326 4

1 3 sLORETA 100 11.326 11.326 1

1 4 Ave 0 35.787 0.000 8002

1 4 Ave 10 32.959 0.000 3065

1 4 Ave 20 26.288 0.000 1337

1 4 Ave 30 19.092 0.000 621

1 4 Ave 40 15.164 0.000 319

1 4 Ave 50 11.879 0.000 165

1 4 Ave 60 9.300 0.000 97

1 4 Ave 70 6.711 0.000 45

1 4 Ave 80 4.111 0.000 23

1 4 Ave 90 0.000 0.000 6

1 4 Ave 100 0.000 0.000 1

1 4 cMEM 0 7.421 0.000 8002

1 4 cMEM 10 5.852 0.000 249

1 4 cMEM 20 4.664 0.000 146

1 4 cMEM 30 3.617 0.000 85

1 4 cMEM 40 3.352 0.000 64

1 4 cMEM 50 3.021 0.000 45

1 4 cMEM 60 2.727 0.000 30

1 4 cMEM 70 1.625 0.000 16

1 4 cMEM 80 0.000 0.000 10

1 4 cMEM 90 0.000 0.000 4

1 4 cMEM 100 0.000 0.000 1

1 4 dSPM 0 33.536 0.000 8002

1 4 dSPM 10 30.521 0.000 2483

1 4 dSPM 20 25.008 0.000 1062

1 4 dSPM 30 22.210 0.000 524

1 4 dSPM 40 20.166 0.000 279

1 4 dSPM 50 17.982 0.000 140

1 4 dSPM 60 16.934 0.000 76

1 4 dSPM 70 15.870 0.000 44

1 4 dSPM 80 14.978 0.000 21

1 4 dSPM 90 13.814 0.000 11

1 4 dSPM 100 0.000 0.000 1

1 4 MNE 0 39.817 0.000 8002

1 4 MNE 10 36.587 0.000 2275

1 4 MNE 20 30.111 0.000 807

1 4 MNE 30 25.167 0.000 374

1 4 MNE 40 18.873 0.000 198

1 4 MNE 50 16.351 0.000 100

1 4 MNE 60 14.594 0.000 47

1 4 MNE 70 12.149 0.000 24

1 4 MNE 80 8.048 0.000 11

1 4 MNE 90 8.290 0.000 3

1 4 MNE 100 10.757 10.757 1

1 4 sLORETA 0 39.186 0.000 8002

1 4 sLORETA 10 37.614 0.000 3806

1 4 sLORETA 20 32.844 0.000 1863

1 4 sLORETA 30 27.407 0.000 979

1 4 sLORETA 40 20.348 0.000 496

1 4 sLORETA 50 16.883 0.000 256

1 4 sLORETA 60 13.015 0.000 123

1 4 sLORETA 70 11.489 0.000 63

1 4 sLORETA 80 9.114 0.000 23

1 4 sLORETA 90 7.336 0.000 7

1 4 sLORETA 100 9.715 9.715 1

1 5 Ave 0 41.487 0.000 8002

1 5 Ave 10 40.055 0.000 4089

1 5 Ave 20 34.401 0.000 1869

1 5 Ave 30 26.754 0.000 869

1 5 Ave 40 19.041 0.000 408

1 5 Ave 50 14.175 0.000 222

1 5 Ave 60 11.797 0.000 124

1 5 Ave 70 10.668 0.000 55

1 5 Ave 80 7.159 0.000 26

1 5 Ave 90 5.533 0.000 8

1 5 Ave 100 14.843 14.843 1

1 5 cMEM 0 12.482 0.000 8002

1 5 cMEM 10 9.449 0.000 330

1 5 cMEM 20 7.716 0.000 139

1 5 cMEM 30 7.052 0.000 96

1 5 cMEM 40 6.720 0.000 70

1 5 cMEM 50 6.414 0.000 50

1 5 cMEM 60 6.339 0.000 30

1 5 cMEM 70 6.396 0.000 26

1 5 cMEM 80 4.290 0.000 14

1 5 cMEM 90 0.000 0.000 4

1 5 cMEM 100 0.000 0.000 1

1 5 dSPM 0 36.712 0.000 8002

1 5 dSPM 10 34.137 0.000 3135

1 5 dSPM 20 27.583 0.000 1289

1 5 dSPM 30 23.256 0.000 621

1 5 dSPM 40 20.399 0.000 311

1 5 dSPM 50 18.831 0.000 193

1 5 dSPM 60 17.263 0.000 106

1 5 dSPM 70 16.673 0.000 67

1 5 dSPM 80 16.161 0.000 26

1 5 dSPM 90 17.325 8.958 6

1 5 dSPM 100 14.437 14.437 1

1 5 MNE 0 48.847 0.000 8002

1 5 MNE 10 47.652 0.000 3260

1 5 MNE 20 44.486 0.000 1327

1 5 MNE 30 40.900 0.000 602

1 5 MNE 40 38.178 0.000 288

1 5 MNE 50 33.118 0.000 142

1 5 MNE 60 25.877 0.000 78

1 5 MNE 70 22.360 0.000 38

1 5 MNE 80 8.627 0.000 12

1 5 MNE 90 7.210 0.000 4

1 5 MNE 100 2.455 2.455 1

1 5 sLORETA 0 43.977 0.000 8002

1 5 sLORETA 10 43.197 0.000 4727

1 5 sLORETA 20 39.860 0.000 2561

1 5 sLORETA 30 34.337 0.000 1351

1 5 sLORETA 40 27.824 0.000 678

1 5 sLORETA 50 19.578 0.000 345

1 5 sLORETA 60 15.355 0.000 183

1 5 sLORETA 70 13.279 0.000 100

1 5 sLORETA 80 11.704 0.000 45

1 5 sLORETA 90 10.407 4.745 9

1 5 sLORETA 100 8.958 8.958 1

1 6 Ave 0 31.245 0.000 8002

1 6 Ave 10 27.251 0.000 2751

1 6 Ave 20 21.035 0.000 1209

1 6 Ave 30 15.699 0.000 593

1 6 Ave 40 12.768 0.000 333

1 6 Ave 50 9.619 0.000 185

1 6 Ave 60 7.934 0.000 103

1 6 Ave 70 5.696 0.000 51

1 6 Ave 80 3.527 0.000 22

1 6 Ave 90 0.000 0.000 10

1 6 Ave 100 0.000 0.000 1

1 6 cMEM 0 6.510 0.000 8002

1 6 cMEM 10 5.050 0.000 171

1 6 cMEM 20 3.212 0.000 90

1 6 cMEM 30 1.229 0.000 40

1 6 cMEM 40 0.686 0.000 18

1 6 cMEM 50 0.478 0.000 14

1 6 cMEM 60 0.000 0.000 9

1 6 cMEM 70 0.000 0.000 6

1 6 cMEM 80 0.000 0.000 2

1 6 cMEM 90 0.000 0.000 2

1 6 cMEM 100 0.000 0.000 1

1 6 dSPM 0 29.390 0.000 8002

1 6 dSPM 10 25.461 0.000 2053

1 6 dSPM 20 21.049 0.000 901

1 6 dSPM 30 17.945 0.000 451

1 6 dSPM 40 16.120 0.000 225

1 6 dSPM 50 14.312 0.000 120

1 6 dSPM 60 13.601 0.000 63

1 6 dSPM 70 12.351 0.000 34

1 6 dSPM 80 10.258 0.000 17

1 6 dSPM 90 8.254 0.000 5

1 6 dSPM 100 0.000 0.000 1

1 6 MNE 0 33.525 0.000 8002

1 6 MNE 10 28.062 0.000 1794

1 6 MNE 20 20.208 0.000 596

1 6 MNE 30 14.628 0.000 290

1 6 MNE 40 10.911 0.000 152

1 6 MNE 50 8.543 0.000 87

1 6 MNE 60 8.876 0.000 47

1 6 MNE 70 7.364 0.000 21

1 6 MNE 80 8.415 0.000 5

1 6 MNE 90 0.000 0.000 1

1 6 MNE 100 0.000 0.000 1

1 6 sLORETA 0 33.772 0.000 8002

1 6 sLORETA 10 30.775 0.000 3129

1 6 sLORETA 20 25.122 0.000 1457

1 6 sLORETA 30 19.440 0.000 721

1 6 sLORETA 40 14.573 0.000 382

1 6 sLORETA 50 11.708 0.000 202

1 6 sLORETA 60 8.532 0.000 107

1 6 sLORETA 70 6.655 0.000 45

1 6 sLORETA 80 4.761 0.000 21

1 6 sLORETA 90 3.330 0.000 8

1 6 sLORETA 100 0.000 0.000 1

1 7 Ave 0 36.492 0.000 8002

1 7 Ave 10 34.229 0.000 3487

1 7 Ave 20 27.990 0.000 1568

1 7 Ave 30 23.657 0.000 809

1 7 Ave 40 20.449 0.000 448

1 7 Ave 50 17.768 0.000 247

1 7 Ave 60 14.471 0.000 114

1 7 Ave 70 12.673 0.000 54

1 7 Ave 80 6.752 0.000 29

1 7 Ave 90 0.000 0.000 12

1 7 Ave 100 0.000 0.000 1

1 7 cMEM 0 9.095 0.000 8002

1 7 cMEM 10 8.005 0.000 294

1 7 cMEM 20 7.034 0.000 160

1 7 cMEM 30 5.492 0.000 101

1 7 cMEM 40 4.046 0.000 60

1 7 cMEM 50 4.220 0.000 41

1 7 cMEM 60 4.274 0.000 27

1 7 cMEM 70 4.448 0.000 17

1 7 cMEM 80 4.049 0.000 6

1 7 cMEM 90 3.627 0.000 4

1 7 cMEM 100 0.000 0.000 1

1 7 dSPM 0 35.540 0.000 8002

1 7 dSPM 10 32.950 0.000 2878

1 7 dSPM 20 28.770 0.000 1344

1 7 dSPM 30 24.961 0.000 664

1 7 dSPM 40 21.228 0.000 347

1 7 dSPM 50 18.088 0.000 173

1 7 dSPM 60 16.023 0.000 85

1 7 dSPM 70 13.154 0.000 39

1 7 dSPM 80 10.545 0.000 12

1 7 dSPM 90 0.000 0.000 2

1 7 dSPM 100 0.000 0.000 1

1 7 MNE 0 38.535 0.000 8002

1 7 MNE 10 34.554 0.000 1998

1 7 MNE 20 28.794 0.000 729

1 7 MNE 30 26.062 0.000 351

1 7 MNE 40 24.548 0.000 176

1 7 MNE 50 22.429 0.000 84

1 7 MNE 60 21.097 0.000 44

1 7 MNE 70 22.047 0.000 22

1 7 MNE 80 22.046 0.000 7

1 7 MNE 90 17.121 0.000 4

1 7 MNE 100 28.378 28.378 1

1 7 sLORETA 0 39.296 0.000 8002

1 7 sLORETA 10 38.054 0.000 4214

1 7 sLORETA 20 33.723 0.000 2191

1 7 sLORETA 30 27.767 0.000 1164

1 7 sLORETA 40 24.781 0.000 666

1 7 sLORETA 50 22.727 0.000 388

1 7 sLORETA 60 20.657 0.000 220

1 7 sLORETA 70 19.523 0.000 111

1 7 sLORETA 80 18.967 0.000 48

1 7 sLORETA 90 15.392 0.000 18

1 7 sLORETA 100 7.819 7.819 1

1 8 Ave 0 33.413 0.000 8002

1 8 Ave 10 30.406 0.000 3034

1 8 Ave 20 22.840 0.000 1262

1 8 Ave 30 17.564 0.000 588

1 8 Ave 40 12.808 0.000 288

1 8 Ave 50 8.943 0.000 166

1 8 Ave 60 6.551 0.000 88

1 8 Ave 70 4.622 0.000 49

1 8 Ave 80 1.837 0.000 24

1 8 Ave 90 0.000 0.000 9

1 8 Ave 100 0.000 0.000 1

1 8 cMEM 0 7.118 0.000 8002

1 8 cMEM 10 6.334 0.000 278

1 8 cMEM 20 5.689 0.000 161

1 8 cMEM 30 5.449 0.000 108

1 8 cMEM 40 4.807 0.000 72

1 8 cMEM 50 2.261 0.000 44

1 8 cMEM 60 1.221 0.000 28

1 8 cMEM 70 1.305 0.000 23

1 8 cMEM 80 0.000 0.000 8

1 8 cMEM 90 0.000 0.000 5

1 8 cMEM 100 0.000 0.000 1

1 8 dSPM 0 32.981 0.000 8002

1 8 dSPM 10 30.055 0.000 2450

1 8 dSPM 20 25.761 0.000 1081

1 8 dSPM 30 22.726 0.000 529

1 8 dSPM 40 19.742 0.000 257

1 8 dSPM 50 17.771 0.000 132

1 8 dSPM 60 15.948 0.000 69

1 8 dSPM 70 14.147 0.000 40

1 8 dSPM 80 12.633 0.000 20

1 8 dSPM 90 9.880 0.000 7

1 8 dSPM 100 0.000 0.000 1

1 8 MNE 0 36.678 0.000 8002

1 8 MNE 10 32.637 0.000 2180

1 8 MNE 20 25.954 0.000 734

1 8 MNE 30 20.358 0.000 354

1 8 MNE 40 15.525 0.000 168

1 8 MNE 50 13.460 0.000 97

1 8 MNE 60 7.142 0.000 44

1 8 MNE 70 7.032 0.000 19

1 8 MNE 80 8.291 0.000 8

1 8 MNE 90 10.196 0.000 2

1 8 MNE 100 0.000 0.000 1

1 8 sLORETA 0 36.799 0.000 8002

1 8 sLORETA 10 34.936 0.000 3682

1 8 sLORETA 20 29.619 0.000 1810

1 8 sLORETA 30 23.791 0.000 939

1 8 sLORETA 40 18.840 0.000 467

1 8 sLORETA 50 13.665 0.000 220

1 8 sLORETA 60 10.053 0.000 115

1 8 sLORETA 70 8.475 0.000 52

1 8 sLORETA 80 6.347 0.000 25

1 8 sLORETA 90 5.095 0.000 10

1 8 sLORETA 100 0.000 0.000 1

1 9 Ave 0 39.604 0.000 8002

1 9 Ave 10 37.883 0.000 3646

1 9 Ave 20 31.152 0.000 1600

1 9 Ave 30 23.294 0.000 736

1 9 Ave 40 15.739 0.000 372

1 9 Ave 50 8.948 0.000 179

1 9 Ave 60 6.130 0.000 98

1 9 Ave 70 5.425 0.000 54

1 9 Ave 80 4.877 0.000 22

1 9 Ave 90 0.000 0.000 4

1 9 Ave 100 0.000 0.000 1

1 9 cMEM 0 10.391 0.000 8002

1 9 cMEM 10 6.413 0.000 265

1 9 cMEM 20 5.035 0.000 147

1 9 cMEM 30 4.138 0.000 99

1 9 cMEM 40 3.840 0.000 72

1 9 cMEM 50 4.033 0.000 46

1 9 cMEM 60 3.971 0.000 33

1 9 cMEM 70 4.004 0.000 23

1 9 cMEM 80 3.902 0.000 11

1 9 cMEM 90 3.429 0.000 5

1 9 cMEM 100 3.589 3.589 1

1 9 dSPM 0 37.885 0.000 8002

1 9 dSPM 10 36.055 0.000 3093

1 9 dSPM 20 31.191 0.000 1424

1 9 dSPM 30 27.462 0.000 769

1 9 dSPM 40 23.648 0.000 407

1 9 dSPM 50 18.661 0.000 208

1 9 dSPM 60 14.409 0.000 100

1 9 dSPM 70 11.846 0.000 53

1 9 dSPM 80 8.839 0.000 24

1 9 dSPM 90 6.002 0.000 7

1 9 dSPM 100 9.015 9.015 1

1 9 MNE 0 42.119 0.000 8002

1 9 MNE 10 39.171 0.000 2386

1 9 MNE 20 30.370 0.000 774

1 9 MNE 30 21.300 0.000 302

1 9 MNE 40 15.431 0.000 162

1 9 MNE 50 10.252 0.000 85

1 9 MNE 60 9.628 0.000 40

1 9 MNE 70 10.113 0.000 20

1 9 MNE 80 10.417 0.000 10

1 9 MNE 90 10.576 0.000 5

1 9 MNE 100 7.819 7.819 1

1 9 sLORETA 0 43.908 0.000 8002

1 9 sLORETA 10 43.112 0.000 4416

1 9 sLORETA 20 39.868 0.000 2385

1 9 sLORETA 30 33.535 0.000 1235

1 9 sLORETA 40 27.169 0.000 640

1 9 sLORETA 50 21.771 0.000 349

1 9 sLORETA 60 18.029 0.000 171

1 9 sLORETA 70 9.861 0.000 69

1 9 sLORETA 80 6.209 0.000 34

1 9 sLORETA 90 6.236 0.000 15

1 9 sLORETA 100 10.004 10.004 1

1 10 Ave 0 41.560 0.000 8002

1 10 Ave 10 40.283 0.000 4066

1 10 Ave 20 35.808 0.000 1884

1 10 Ave 30 28.989 0.000 896

1 10 Ave 40 22.087 0.000 492

1 10 Ave 50 14.815 0.000 254

1 10 Ave 60 10.933 0.000 157

1 10 Ave 70 9.674 0.000 88

1 10 Ave 80 7.016 0.000 40

1 10 Ave 90 6.898 0.000 14

1 10 Ave 100 0.000 0.000 1

1 10 cMEM 0 11.323 0.000 8002

1 10 cMEM 10 6.358 0.000 225

1 10 cMEM 20 5.664 0.000 149

1 10 cMEM 30 5.282 0.000 108

1 10 cMEM 40 4.373 0.000 65

1 10 cMEM 50 4.611 0.000 42

1 10 cMEM 60 4.918 0.000 20

1 10 cMEM 70 5.333 0.000 13

1 10 cMEM 80 5.390 0.000 9

1 10 cMEM 90 5.200 3.589 2

1 10 cMEM 100 3.589 3.589 1

1 10 dSPM 0 37.883 0.000 8002

1 10 dSPM 10 35.801 0.000 3178

1 10 dSPM 20 29.770 0.000 1381

1 10 dSPM 30 21.752 0.000 642

1 10 dSPM 40 18.026 0.000 323

1 10 dSPM 50 17.096 0.000 171

1 10 dSPM 60 15.584 0.000 90

1 10 dSPM 70 13.958 0.000 53

1 10 dSPM 80 11.830 0.000 26

1 10 dSPM 90 11.110 0.000 9

1 10 dSPM 100 0.000 0.000 1

1 10 MNE 0 46.727 0.000 8002

1 10 MNE 10 44.463 0.000 2285

1 10 MNE 20 39.885 0.000 761

1 10 MNE 30 35.888 0.000 338

1 10 MNE 40 32.449 0.000 167

1 10 MNE 50 31.218 0.000 70

1 10 MNE 60 27.553 0.000 29

1 10 MNE 70 16.272 7.819 15

1 10 MNE 80 16.734 11.326 8

1 10 MNE 90 18.447 12.847 3

1 10 MNE 100 14.853 14.853 1

1 10 sLORETA 0 45.814 0.000 8002

1 10 sLORETA 10 45.011 0.000 4460

1 10 sLORETA 20 42.321 0.000 2284

1 10 sLORETA 30 38.628 0.000 1230

1 10 sLORETA 40 31.607 0.000 617

1 10 sLORETA 50 23.450 0.000 315

1 10 sLORETA 60 14.379 0.000 146

1 10 sLORETA 70 11.300 0.000 81

1 10 sLORETA 80 9.889 0.000 32

1 10 sLORETA 90 9.206 0.000 11

1 10 sLORETA 100 9.526 9.526 1

1 11 Ave 0 32.661 0.000 8002

1 11 Ave 10 28.991 0.000 2529

1 11 Ave 20 22.942 0.000 1050

1 11 Ave 30 18.390 0.000 494

1 11 Ave 40 13.208 0.000 233

1 11 Ave 50 7.815 0.000 115

1 11 Ave 60 5.694 0.000 59

1 11 Ave 70 1.127 0.000 25

1 11 Ave 80 0.000 0.000 13

1 11 Ave 90 0.000 0.000 5

1 11 Ave 100 0.000 0.000 1

1 11 cMEM 0 4.556 0.000 8002

1 11 cMEM 10 3.300 0.000 164

1 11 cMEM 20 2.764 0.000 91

1 11 cMEM 30 2.423 0.000 53

1 11 cMEM 40 1.599 0.000 21

1 11 cMEM 50 0.000 0.000 13

1 11 cMEM 60 0.000 0.000 12

1 11 cMEM 70 0.000 0.000 8

1 11 cMEM 80 0.000 0.000 4

1 11 cMEM 90 0.000 0.000 2

1 11 cMEM 100 0.000 0.000 1

1 11 dSPM 0 32.137 0.000 8002

1 11 dSPM 10 29.073 0.000 2269

1 11 dSPM 20 24.852 0.000 1045

1 11 dSPM 30 21.022 0.000 485

1 11 dSPM 40 17.160 0.000 235

1 11 dSPM 50 15.851 0.000 118

1 11 dSPM 60 15.381 0.000 66

1 11 dSPM 70 13.401 0.000 35

1 11 dSPM 80 9.954 0.000 17

1 11 dSPM 90 7.848 0.000 3

1 11 dSPM 100 0.000 0.000 1

1 11 MNE 0 34.485 0.000 8002

1 11 MNE 10 29.391 0.000 1483

1 11 MNE 20 24.774 0.000 561

1 11 MNE 30 21.557 0.000 263

1 11 MNE 40 18.203 0.000 136

1 11 MNE 50 16.535 0.000 60

1 11 MNE 60 15.380 0.000 25

1 11 MNE 70 10.247 0.000 10

1 11 MNE 80 0.000 0.000 2

1 11 MNE 90 0.000 0.000 1

1 11 MNE 100 0.000 0.000 1

1 11 sLORETA 0 35.091 0.000 8002

1 11 sLORETA 10 32.927 0.000 3337

1 11 sLORETA 20 27.396 0.000 1582

1 11 sLORETA 30 23.276 0.000 828

1 11 sLORETA 40 19.838 0.000 435

1 11 sLORETA 50 15.552 0.000 232

1 11 sLORETA 60 9.300 0.000 114

1 11 sLORETA 70 5.994 0.000 58

1 11 sLORETA 80 4.001 0.000 23

1 11 sLORETA 90 0.000 0.000 7

1 11 sLORETA 100 0.000 0.000 1

2 12 Ave 0 34.436 0.000 8002

2 12 Ave 10 32.217 0.000 2988

2 12 Ave 20 25.646 0.000 1234

2 12 Ave 30 16.546 0.000 544

2 12 Ave 40 6.990 0.000 257

2 12 Ave 50 4.198 0.000 129

2 12 Ave 60 3.453 0.000 75

2 12 Ave 70 2.464 0.000 40

2 12 Ave 80 1.817 0.000 17

2 12 Ave 90 1.590 0.000 8

2 12 Ave 100 0.000 0.000 1

2 12 cMEM 0 9.390 0.000 8002

2 12 cMEM 10 6.492 0.000 381

2 12 cMEM 20 4.996 0.000 191

2 12 cMEM 30 3.646 0.000 103

2 12 cMEM 40 3.438 0.000 59

2 12 cMEM 50 3.397 0.000 36

2 12 cMEM 60 2.213 0.000 20

2 12 cMEM 70 2.092 0.000 10

2 12 cMEM 80 0.000 0.000 4

2 12 cMEM 90 0.000 0.000 2

2 12 cMEM 100 0.000 0.000 1

2 12 dSPM 0 30.674 0.000 8002

2 12 dSPM 10 27.991 0.000 2252

2 12 dSPM 20 22.964 0.000 944

2 12 dSPM 30 18.082 0.000 480

2 12 dSPM 40 14.313 0.000 250

2 12 dSPM 50 10.084 0.000 149

2 12 dSPM 60 6.689 0.000 78

2 12 dSPM 70 3.728 0.000 45

2 12 dSPM 80 3.037 0.000 19

2 12 dSPM 90 0.000 0.000 9

2 12 dSPM 100 0.000 0.000 1

2 12 MNE 0 39.369 0.000 8002

2 12 MNE 10 37.092 0.000 2547

2 12 MNE 20 30.632 0.000 894

2 12 MNE 30 21.295 0.000 307

2 12 MNE 40 14.823 0.000 151

2 12 MNE 50 9.621 0.000 85

2 12 MNE 60 5.578 0.000 37

2 12 MNE 70 4.583 0.000 24

2 12 MNE 80 1.579 0.000 11

2 12 MNE 90 1.080 0.000 6

2 12 MNE 100 0.000 0.000 1

2 12 sLORETA 0 38.584 0.000 8002

2 12 sLORETA 10 37.305 0.000 3625

2 12 sLORETA 20 34.077 0.000 1774

2 12 sLORETA 30 28.788 0.000 871

2 12 sLORETA 40 20.621 0.000 411

2 12 sLORETA 50 7.751 0.000 179

2 12 sLORETA 60 4.491 0.000 95

2 12 sLORETA 70 3.436 0.000 45

2 12 sLORETA 80 1.521 0.000 13

2 12 sLORETA 90 0.000 0.000 4

2 12 sLORETA 100 0.000 0.000 1

3 13 Ave 0 39.161 0.000 8002

3 13 Ave 10 35.608 0.000 2499

3 13 Ave 20 32.127 0.000 1184

3 13 Ave 30 26.504 0.000 562

3 13 Ave 40 20.994 0.000 288

3 13 Ave 50 16.477 0.000 165

3 13 Ave 60 13.464 0.000 86

3 13 Ave 70 14.246 0.000 39

3 13 Ave 80 18.060 0.000 12

3 13 Ave 90 16.543 14.792 3

3 13 Ave 100 14.792 14.792 1

3 13 cMEM 0 24.087 0.000 8002

3 13 cMEM 10 23.043 0.000 380

3 13 cMEM 20 22.776 0.000 156

3 13 cMEM 30 20.539 0.000 90

3 13 cMEM 40 18.714 0.000 55

3 13 cMEM 50 17.412 0.000 35

3 13 cMEM 60 17.033 0.000 28

3 13 cMEM 70 16.411 0.000 15

3 13 cMEM 80 18.214 0.000 9

3 13 cMEM 90 21.898 17.154 4

3 13 cMEM 100 24.042 24.042 1

3 13 dSPM 0 42.599 0.000 8002

3 13 dSPM 10 39.970 0.000 3119

3 13 dSPM 20 36.425 0.000 1520

3 13 dSPM 30 33.060 0.000 863

3 13 dSPM 40 28.307 0.000 467

3 13 dSPM 50 23.388 0.000 243

3 13 dSPM 60 18.337 0.000 118

3 13 dSPM 70 15.706 0.000 57

3 13 dSPM 80 13.498 0.000 26

3 13 dSPM 90 13.912 9.522 9

3 13 dSPM 100 9.522 9.522 1

3 13 MNE 0 40.164 0.000 8002

3 13 MNE 10 36.776 0.000 1897

3 13 MNE 20 32.634 0.000 783

3 13 MNE 30 28.841 0.000 361

3 13 MNE 40 23.701 0.000 205

3 13 MNE 50 19.452 0.000 122

3 13 MNE 60 15.950 0.000 74

3 13 MNE 70 16.394 0.000 38

3 13 MNE 80 10.250 0.000 16

3 13 MNE 90 11.564 5.474 2

3 13 MNE 100 14.792 14.792 1

3 13 sLORETA 0 38.623 0.000 8002

3 13 sLORETA 10 35.551 0.000 2547

3 13 sLORETA 20 32.341 0.000 1199

3 13 sLORETA 30 27.080 0.000 578

3 13 sLORETA 40 22.955 0.000 299

3 13 sLORETA 50 17.888 0.000 163

3 13 sLORETA 60 14.405 0.000 86

3 13 sLORETA 70 12.707 0.000 39

3 13 sLORETA 80 10.854 0.000 16

3 13 sLORETA 90 13.760 13.752 2

3 13 sLORETA 100 13.752 13.752 1

3 14 Ave 0 40.596 0.000 8002

3 14 Ave 10 38.159 0.000 3555

3 14 Ave 20 32.437 0.000 1755

3 14 Ave 30 27.508 0.000 895

3 14 Ave 40 24.101 0.000 483

3 14 Ave 50 22.052 0.000 269

3 14 Ave 60 18.810 0.000 132

3 14 Ave 70 17.640 0.000 56

3 14 Ave 80 13.785 0.000 19

3 14 Ave 90 11.126 0.000 3

3 14 Ave 100 13.068 13.068 1

3 14 cMEM 0 27.842 0.000 8002

3 14 cMEM 10 26.693 0.000 418

3 14 cMEM 20 26.268 0.000 157

3 14 cMEM 30 25.084 0.000 70

3 14 cMEM 40 23.147 0.000 31

3 14 cMEM 50 18.912 0.000 15

3 14 cMEM 60 17.204 13.068 5

3 14 cMEM 70 17.204 13.068 5

3 14 cMEM 80 16.304 13.068 2

3 14 cMEM 90 13.068 13.068 1

3 14 cMEM 100 13.068 13.068 1

3 14 dSPM 0 44.656 0.000 8002

3 14 dSPM 10 42.911 0.000 3900

3 14 dSPM 20 37.911 0.000 1887

3 14 dSPM 30 33.403 0.000 989

3 14 dSPM 40 30.232 0.000 521

3 14 dSPM 50 30.313 0.000 286

3 14 dSPM 60 31.060 0.000 152

3 14 dSPM 70 29.418 0.000 73

3 14 dSPM 80 33.571 0.000 31

3 14 dSPM 90 36.173 13.982 9

3 14 dSPM 100 46.602 46.602 1

3 14 MNE 0 41.013 0.000 8002

3 14 MNE 10 37.412 0.000 2103

3 14 MNE 20 30.644 0.000 825

3 14 MNE 30 25.610 0.000 389

3 14 MNE 40 22.280 0.000 188

3 14 MNE 50 21.027 0.000 83

3 14 MNE 60 20.907 0.000 39

3 14 MNE 70 19.279 0.000 23

3 14 MNE 80 20.902 0.000 7

3 14 MNE 90 31.140 31.140 1

3 14 MNE 100 31.140 31.140 1

3 14 sLORETA 0 39.170 0.000 8002

3 14 sLORETA 10 36.805 0.000 3574

3 14 sLORETA 20 32.496 0.000 1878

3 14 sLORETA 30 27.932 0.000 998

3 14 sLORETA 40 23.969 0.000 540

3 14 sLORETA 50 20.755 0.000 297

3 14 sLORETA 60 18.855 0.000 150

3 14 sLORETA 70 16.467 0.000 87

3 14 sLORETA 80 13.921 0.000 32

3 14 sLORETA 90 13.101 0.000 9

3 14 sLORETA 100 13.632 13.632 1

3 15 Ave 0 41.590 0.000 8002

3 15 Ave 10 38.532 0.000 3473

3 15 Ave 20 31.538 0.000 1541

3 15 Ave 30 25.789 0.000 734

3 15 Ave 40 20.563 0.000 338

3 15 Ave 50 19.021 0.000 173

3 15 Ave 60 16.622 0.000 73

3 15 Ave 70 20.667 0.000 23

3 15 Ave 80 27.813 0.000 4

3 15 Ave 90 28.729 28.729 1

3 15 Ave 100 28.729 28.729 1

3 15 cMEM 0 118.291 0.000 8002

3 15 cMEM 10 118.433 96.016 138

3 15 cMEM 20 119.063 102.723 77

3 15 cMEM 30 119.464 108.120 33

3 15 cMEM 40 119.984 108.120 17

3 15 cMEM 50 120.382 112.187 13

3 15 cMEM 60 120.121 112.187 10

3 15 cMEM 70 119.610 112.187 7

3 15 cMEM 80 120.215 116.782 5

3 15 cMEM 90 119.818 116.782 3

3 15 cMEM 100 121.346 121.346 1

3 15 dSPM 0 44.327 0.000 8002

3 15 dSPM 10 41.937 0.000 3697

3 15 dSPM 20 34.519 0.000 1638

3 15 dSPM 30 27.620 0.000 754

3 15 dSPM 40 22.314 0.000 368

3 15 dSPM 50 18.077 0.000 157

3 15 dSPM 60 14.817 0.000 66

3 15 dSPM 70 12.701 0.000 26

3 15 dSPM 80 9.862 0.000 11

3 15 dSPM 90 10.085 0.000 5

3 15 dSPM 100 12.721 12.721 1

3 15 MNE 0 40.089 0.000 8002

3 15 MNE 10 33.597 0.000 1221

3 15 MNE 20 26.989 0.000 376

3 15 MNE 30 24.837 0.000 148

3 15 MNE 40 24.625 0.000 55

3 15 MNE 50 27.137 0.000 22

3 15 MNE 60 31.024 28.729 5

3 15 MNE 70 30.229 28.729 3

3 15 MNE 80 30.113 28.729 2

3 15 MNE 90 30.113 28.729 2

3 15 MNE 100 28.729 28.729 1

3 15 sLORETA 0 40.347 0.000 8002

3 15 sLORETA 10 38.086 0.000 3733

3 15 sLORETA 20 33.412 0.000 1941

3 15 sLORETA 30 28.761 0.000 1048

3 15 sLORETA 40 24.681 0.000 583

3 15 sLORETA 50 20.083 0.000 298

3 15 sLORETA 60 17.960 0.000 154

3 15 sLORETA 70 18.301 0.000 77

3 15 sLORETA 80 18.794 0.000 21

3 15 sLORETA 90 30.394 27.241 5

3 15 sLORETA 100 28.729 28.729 1

3 16 Ave 0 48.676 0.000 8002

3 16 Ave 10 47.119 0.000 4383

3 16 Ave 20 41.203 0.000 2021

3 16 Ave 30 33.160 0.000 942

3 16 Ave 40 24.892 0.000 448

3 16 Ave 50 18.211 0.000 230

3 16 Ave 60 12.918 0.000 124

3 16 Ave 70 12.039 0.000 53

3 16 Ave 80 9.528 0.000 22

3 16 Ave 90 10.372 0.000 2

3 16 Ave 100 0.000 0.000 1

3 16 cMEM 0 31.156 0.000 8002

3 16 cMEM 10 23.135 0.000 273

3 16 cMEM 20 11.679 0.000 103

3 16 cMEM 30 3.342 0.000 47

3 16 cMEM 40 2.518 0.000 29

3 16 cMEM 50 0.000 0.000 18

3 16 cMEM 60 0.000 0.000 11

3 16 cMEM 70 0.000 0.000 6

3 16 cMEM 80 0.000 0.000 5

3 16 cMEM 90 0.000 0.000 3

3 16 cMEM 100 0.000 0.000 1

3 16 dSPM 0 54.783 0.000 8002

3 16 dSPM 10 53.837 0.000 4548

3 16 dSPM 20 51.005 0.000 2373

3 16 dSPM 30 47.034 0.000 1149

3 16 dSPM 40 40.680 0.000 496

3 16 dSPM 50 36.841 0.000 229

3 16 dSPM 60 32.683 0.000 99

3 16 dSPM 70 25.317 0.000 39

3 16 dSPM 80 14.777 0.000 16

3 16 dSPM 90 14.669 0.000 5

3 16 dSPM 100 12.656 12.656 1

3 16 MNE 0 46.157 0.000 8002

3 16 MNE 10 43.255 0.000 2739

3 16 MNE 20 37.448 0.000 1083

3 16 MNE 30 33.100 0.000 538

3 16 MNE 40 30.472 0.000 319

3 16 MNE 50 28.443 0.000 179

3 16 MNE 60 23.723 0.000 91

3 16 MNE 70 25.544 0.000 42

3 16 MNE 80 18.974 0.000 20

3 16 MNE 90 24.533 0.000 9

3 16 MNE 100 14.792 14.792 1

3 16 sLORETA 0 47.125 0.000 8002

3 16 sLORETA 10 45.285 0.000 4168

3 16 sLORETA 20 38.837 0.000 1905

3 16 sLORETA 30 30.896 0.000 871

3 16 sLORETA 40 24.457 0.000 405

3 16 sLORETA 50 17.375 0.000 199

3 16 sLORETA 60 14.494 0.000 104

3 16 sLORETA 70 13.139 0.000 46

3 16 sLORETA 80 13.364 5.850 19

3 16 sLORETA 90 13.200 7.225 6

3 16 sLORETA 100 13.752 13.752 1

3 17 Ave 0 40.616 0.000 8002

3 17 Ave 10 37.448 0.000 3255

3 17 Ave 20 30.038 0.000 1396

3 17 Ave 30 23.789 0.000 705

3 17 Ave 40 19.441 0.000 373

3 17 Ave 50 17.167 0.000 213

3 17 Ave 60 15.247 0.000 86

3 17 Ave 70 15.804 0.000 30

3 17 Ave 80 16.068 0.000 8

3 17 Ave 90 6.072 0.000 3

3 17 Ave 100 6.516 6.516 1

3 17 cMEM 0 27.645 0.000 8002

3 17 cMEM 10 25.890 0.000 725

3 17 cMEM 20 23.448 0.000 325

3 17 cMEM 30 21.278 0.000 167

3 17 cMEM 40 18.777 0.000 98

3 17 cMEM 50 17.764 0.000 58

3 17 cMEM 60 18.631 0.000 28

3 17 cMEM 70 20.028 0.000 17

3 17 cMEM 80 26.618 0.000 5

3 17 cMEM 90 25.546 0.000 4

3 17 cMEM 100 33.774 33.774 1

3 17 dSPM 0 45.476 0.000 8002

3 17 dSPM 10 43.280 0.000 3582

3 17 dSPM 20 37.427 0.000 1661

3 17 dSPM 30 31.383 0.000 796

3 17 dSPM 40 27.099 0.000 424

3 17 dSPM 50 26.188 0.000 201

3 17 dSPM 60 22.514 0.000 91

3 17 dSPM 70 20.349 0.000 33

3 17 dSPM 80 13.463 10.076 6

3 17 dSPM 90 11.038 11.038 1

3 17 dSPM 100 11.038 11.038 1

3 17 MNE 0 39.119 0.000 8002

3 17 MNE 10 32.969 0.000 1466

3 17 MNE 20 24.942 0.000 505

3 17 MNE 30 22.240 0.000 248

3 17 MNE 40 22.965 0.000 130

3 17 MNE 50 24.142 0.000 69

3 17 MNE 60 26.410 0.000 25

3 17 MNE 70 30.004 0.000 11

3 17 MNE 80 34.181 7.469 6

3 17 MNE 90 38.154 31.703 2

3 17 MNE 100 31.703 31.703 1

3 17 sLORETA 0 40.181 0.000 8002

3 17 sLORETA 10 37.758 0.000 3581

3 17 sLORETA 20 31.581 0.000 1701

3 17 sLORETA 30 25.893 0.000 891

3 17 sLORETA 40 21.784 0.000 527

3 17 sLORETA 50 19.357 0.000 309

3 17 sLORETA 60 17.668 0.000 170

3 17 sLORETA 70 16.068 0.000 77

3 17 sLORETA 80 14.268 0.000 31

3 17 sLORETA 90 3.602 0.000 10

3 17 sLORETA 100 0.000 0.000 1

4 18 Ave 0 26.388 0.000 8002

4 18 Ave 10 23.739 0.000 2062

4 18 Ave 20 19.043 0.000 805

4 18 Ave 30 14.703 0.000 370

4 18 Ave 40 5.306 0.000 179

4 18 Ave 50 0.916 0.000 105

4 18 Ave 60 0.793 0.000 56

4 18 Ave 70 0.000 0.000 35

4 18 Ave 80 0.000 0.000 11

4 18 Ave 90 0.000 0.000 3

4 18 Ave 100 0.000 0.000 1

4 18 cMEM 0 10.220 0.000 8002

4 18 cMEM 10 6.668 0.000 220

4 18 cMEM 20 5.372 0.000 126

4 18 cMEM 30 5.426 0.000 73

4 18 cMEM 40 5.552 0.000 46

4 18 cMEM 50 5.419 0.000 33

4 18 cMEM 60 4.170 0.000 15

4 18 cMEM 70 4.152 0.000 8

4 18 cMEM 80 3.621 0.000 4

4 18 cMEM 90 0.000 0.000 1

4 18 cMEM 100 0.000 0.000 1

4 18 dSPM 0 31.261 0.000 8002

4 18 dSPM 10 30.104 0.000 2987

4 18 dSPM 20 27.903 0.000 1486

4 18 dSPM 30 25.265 0.000 750

4 18 dSPM 40 21.907 0.000 402

4 18 dSPM 50 17.678 0.000 207

4 18 dSPM 60 13.679 0.000 109

4 18 dSPM 70 5.239 0.000 58

4 18 dSPM 80 1.489 0.000 32

4 18 dSPM 90 1.420 0.000 11

4 18 dSPM 100 0.000 0.000 1

4 18 MNE 0 23.241 0.000 8002

4 18 MNE 10 16.805 0.000 673

4 18 MNE 20 9.675 0.000 195

4 18 MNE 30 1.695 0.000 79

4 18 MNE 40 0.000 0.000 38

4 18 MNE 50 0.000 0.000 19

4 18 MNE 60 0.000 0.000 11

4 18 MNE 70 0.000 0.000 6

4 18 MNE 80 0.000 0.000 1

4 18 MNE 90 0.000 0.000 1

4 18 MNE 100 0.000 0.000 1

4 18 sLORETA 0 25.311 0.000 8002

4 18 sLORETA 10 22.970 0.000 1830

4 18 sLORETA 20 19.847 0.000 804

4 18 sLORETA 30 15.826 0.000 403

4 18 sLORETA 40 10.658 0.000 219

4 18 sLORETA 50 1.525 0.000 114

4 18 sLORETA 60 1.096 0.000 66

4 18 sLORETA 70 0.000 0.000 23

4 18 sLORETA 80 0.000 0.000 10

4 18 sLORETA 90 0.000 0.000 3

4 18 sLORETA 100 0.000 0.000 1

4 19 Ave 0 26.055 0.000 8002

4 19 Ave 10 21.024 0.000 1645

4 19 Ave 20 12.063 0.000 507

4 19 Ave 30 4.531 0.000 220

4 19 Ave 40 0.700 0.000 106

4 19 Ave 50 0.000 0.000 68

4 19 Ave 60 0.000 0.000 37

4 19 Ave 70 0.000 0.000 20

4 19 Ave 80 0.000 0.000 7

4 19 Ave 90 0.000 0.000 2

4 19 Ave 100 0.000 0.000 1

4 19 cMEM 0 8.691 0.000 8002

4 19 cMEM 10 6.002 0.000 206

4 19 cMEM 20 0.938 0.000 79

4 19 cMEM 30 0.000 0.000 41

4 19 cMEM 40 0.000 0.000 22

4 19 cMEM 50 0.000 0.000 14

4 19 cMEM 60 0.000 0.000 9

4 19 cMEM 70 0.000 0.000 5

4 19 cMEM 80 0.000 0.000 4

4 19 cMEM 90 0.000 0.000 1

4 19 cMEM 100 0.000 0.000 1

4 19 dSPM 0 30.181 0.000 8002

4 19 dSPM 10 28.585 0.000 3158

4 19 dSPM 20 23.246 0.000 1212

4 19 dSPM 30 17.949 0.000 571

4 19 dSPM 40 12.991 0.000 296

4 19 dSPM 50 9.094 0.000 160

4 19 dSPM 60 4.354 0.000 88

4 19 dSPM 70 0.704 0.000 49

4 19 dSPM 80 0.000 0.000 20

4 19 dSPM 90 0.000 0.000 9

4 19 dSPM 100 0.000 0.000 1

4 19 MNE 0 26.171 0.000 8002

4 19 MNE 10 19.323 0.000 648

4 19 MNE 20 11.999 0.000 217

4 19 MNE 30 4.991 0.000 90

4 19 MNE 40 0.647 0.000 43

4 19 MNE 50 0.000 0.000 22

4 19 MNE 60 0.000 0.000 8

4 19 MNE 70 0.000 0.000 4

4 19 MNE 80 0.000 0.000 2

4 19 MNE 90 0.000 0.000 1

4 19 MNE 100 0.000 0.000 1

4 19 sLORETA 0 24.074 0.000 8002

4 19 sLORETA 10 20.117 0.000 1858

4 19 sLORETA 20 13.102 0.000 665

4 19 sLORETA 30 6.294 0.000 343

4 19 sLORETA 40 2.168 0.000 192

4 19 sLORETA 50 0.672 0.000 110

4 19 sLORETA 60 0.000 0.000 70

4 19 sLORETA 70 0.000 0.000 42

4 19 sLORETA 80 0.000 0.000 14

4 19 sLORETA 90 0.000 0.000 6

4 19 sLORETA 100 0.000 0.000 1

4 20 Ave 0 34.273 0.000 8002

4 20 Ave 10 33.120 0.000 3462

4 20 Ave 20 29.059 0.000 1266

4 20 Ave 30 23.442 0.000 518

4 20 Ave 40 19.942 0.000 276

4 20 Ave 50 17.508 0.000 153

4 20 Ave 60 15.256 0.000 67

4 20 Ave 70 4.345 0.000 27

4 20 Ave 80 0.000 0.000 12

4 20 Ave 90 0.000 0.000 5

4 20 Ave 100 0.000 0.000 1

4 20 cMEM 0 10.781 0.000 8002

4 20 cMEM 10 4.744 0.000 154

4 20 cMEM 20 2.508 0.000 86

4 20 cMEM 30 1.955 0.000 64

4 20 cMEM 40 1.315 0.000 34

4 20 cMEM 50 0.000 0.000 19

4 20 cMEM 60 0.000 0.000 11

4 20 cMEM 70 0.000 0.000 5

4 20 cMEM 80 0.000 0.000 3

4 20 cMEM 90 0.000 0.000 2

4 20 cMEM 100 0.000 0.000 1

4 20 dSPM 0 37.176 0.000 8002

4 20 dSPM 10 36.818 0.000 4664

4 20 dSPM 20 35.316 0.000 2484

4 20 dSPM 30 32.810 0.000 1282

4 20 dSPM 40 29.642 0.000 657

4 20 dSPM 50 25.180 0.000 349

4 20 dSPM 60 21.691 0.000 197

4 20 dSPM 70 20.217 0.000 116

4 20 dSPM 80 17.703 0.000 50

4 20 dSPM 90 17.454 0.000 13

4 20 dSPM 100 0.000 0.000 1

4 20 MNE 0 37.198 0.000 8002

4 20 MNE 10 35.586 0.000 1330

4 20 MNE 20 32.246 0.000 378

4 20 MNE 30 29.812 0.000 170

4 20 MNE 40 26.184 0.000 77

4 20 MNE 50 25.643 0.000 33

4 20 MNE 60 28.768 0.000 15

4 20 MNE 70 34.468 0.000 4

4 20 MNE 80 45.466 0.000 2

4 20 MNE 90 0.000 0.000 1

4 20 MNE 100 0.000 0.000 1

4 20 sLORETA 0 31.492 0.000 8002

4 20 sLORETA 10 29.697 0.000 2908

4 20 sLORETA 20 25.062 0.000 1049

4 20 sLORETA 30 20.524 0.000 487

4 20 sLORETA 40 17.667 0.000 248

4 20 sLORETA 50 15.786 0.000 132

4 20 sLORETA 60 7.141 0.000 48

4 20 sLORETA 70 1.026 0.000 18

4 20 sLORETA 80 0.000 0.000 4

4 20 sLORETA 90 0.000 0.000 1

4 20 sLORETA 100 0.000 0.000 1

4 21 Ave 0 25.914 0.000 8002

4 21 Ave 10 23.119 0.000 2172

4 21 Ave 20 18.481 0.000 917

4 21 Ave 30 11.462 0.000 402

4 21 Ave 40 3.933 0.000 211

4 21 Ave 50 0.819 0.000 112

4 21 Ave 60 0.000 0.000 57

4 21 Ave 70 0.000 0.000 32

4 21 Ave 80 0.000 0.000 19

4 21 Ave 90 0.000 0.000 7

4 21 Ave 100 0.000 0.000 1

4 21 cMEM 0 9.918 0.000 8002

4 21 cMEM 10 2.261 0.000 121

4 21 cMEM 20 1.438 0.000 60

4 21 cMEM 30 0.991 0.000 24

4 21 cMEM 40 0.738 0.000 17

4 21 cMEM 50 0.000 0.000 13

4 21 cMEM 60 0.000 0.000 10

4 21 cMEM 70 0.000 0.000 6

4 21 cMEM 80 0.000 0.000 3

4 21 cMEM 90 0.000 0.000 2

4 21 cMEM 100 0.000 0.000 1

4 21 dSPM 0 30.001 0.000 8002

4 21 dSPM 10 28.765 0.000 3147

4 21 dSPM 20 25.525 0.000 1514

4 21 dSPM 30 21.973 0.000 804

4 21 dSPM 40 17.704 0.000 412

4 21 dSPM 50 13.692 0.000 215

4 21 dSPM 60 9.433 0.000 119

4 21 dSPM 70 1.288 0.000 46

4 21 dSPM 80 0.000 0.000 12

4 21 dSPM 90 0.000 0.000 5

4 21 dSPM 100 0.000 0.000 1

4 21 MNE 0 25.121 0.000 8002

4 21 MNE 10 20.582 0.000 963

4 21 MNE 20 15.541 0.000 328

4 21 MNE 30 7.883 0.000 125

4 21 MNE 40 3.320 0.000 58

4 21 MNE 50 0.000 0.000 32

4 21 MNE 60 0.000 0.000 15

4 21 MNE 70 0.000 0.000 6

4 21 MNE 80 0.000 0.000 2

4 21 MNE 90 0.000 0.000 1

4 21 MNE 100 0.000 0.000 1

4 21 sLORETA 0 25.098 0.000 8002

4 21 sLORETA 10 23.054 0.000 2262

4 21 sLORETA 20 20.212 0.000 1109

4 21 sLORETA 30 16.605 0.000 597

4 21 sLORETA 40 9.698 0.000 330

4 21 sLORETA 50 3.864 0.000 193

4 21 sLORETA 60 1.842 0.000 124

4 21 sLORETA 70 0.593 0.000 73

4 21 sLORETA 80 0.000 0.000 26

4 21 sLORETA 90 0.000 0.000 7

4 21 sLORETA 100 0.000 0.000 1

4 22 Ave 0 25.848 0.000 8002

4 22 Ave 10 22.582 0.000 1914

4 22 Ave 20 16.737 0.000 718

4 22 Ave 30 11.633 0.000 328

4 22 Ave 40 3.872 0.000 183

4 22 Ave 50 0.940 0.000 102

4 22 Ave 60 0.541 0.000 60

4 22 Ave 70 0.000 0.000 30

4 22 Ave 80 0.000 0.000 13

4 22 Ave 90 0.000 0.000 4

4 22 Ave 100 0.000 0.000 1

4 22 cMEM 0 4.433 0.000 8002

4 22 cMEM 10 3.684 0.000 136

4 22 cMEM 20 3.652 0.000 67

4 22 cMEM 30 3.362 0.000 43

4 22 cMEM 40 3.475 0.000 28

4 22 cMEM 50 2.933 0.000 14

4 22 cMEM 60 1.885 0.000 4

4 22 cMEM 70 0.000 0.000 2

4 22 cMEM 80 0.000 0.000 1

4 22 cMEM 90 0.000 0.000 1

4 22 cMEM 100 0.000 0.000 1

4 22 dSPM 0 30.988 0.000 8002

4 22 dSPM 10 29.801 0.000 2964

4 22 dSPM 20 26.862 0.000 1365

4 22 dSPM 30 20.958 0.000 637

4 22 dSPM 40 17.228 0.000 337

4 22 dSPM 50 14.399 0.000 209

4 22 dSPM 60 11.063 0.000 122

4 22 dSPM 70 6.995 0.000 61

4 22 dSPM 80 1.469 0.000 34

4 22 dSPM 90 1.567 0.000 8

4 22 dSPM 100 0.000 0.000 1

4 22 MNE 0 23.690 0.000 8002

4 22 MNE 10 17.154 0.000 754

4 22 MNE 20 9.516 0.000 221

4 22 MNE 30 6.127 0.000 105

4 22 MNE 40 1.513 0.000 49

4 22 MNE 50 0.000 0.000 26

4 22 MNE 60 0.000 0.000 16

4 22 MNE 70 0.000 0.000 5

4 22 MNE 80 0.000 0.000 4

4 22 MNE 90 0.000 0.000 1

4 22 MNE 100 0.000 0.000 1

4 22 sLORETA 0 23.774 0.000 8002

4 22 sLORETA 10 21.034 0.000 1782

4 22 sLORETA 20 17.599 0.000 810

4 22 sLORETA 30 13.718 0.000 424

4 22 sLORETA 40 5.309 0.000 218

4 22 sLORETA 50 1.118 0.000 137

4 22 sLORETA 60 1.040 0.000 89

4 22 sLORETA 70 0.721 0.000 37

4 22 sLORETA 80 0.000 0.000 14

4 22 sLORETA 90 0.000 0.000 5

4 22 sLORETA 100 0.000 0.000 1

4 23 Ave 0 23.201 0.000 8002

4 23 Ave 10 19.277 0.000 1866

4 23 Ave 20 11.910 0.000 674

4 23 Ave 30 4.848 0.000 286

4 23 Ave 40 1.673 0.000 154

4 23 Ave 50 0.685 0.000 96

4 23 Ave 60 0.000 0.000 56

4 23 Ave 70 0.000 0.000 31

4 23 Ave 80 0.000 0.000 19

4 23 Ave 90 0.000 0.000 10

4 23 Ave 100 0.000 0.000 1

4 23 cMEM 0 7.286 0.000 8002

4 23 cMEM 10 6.238 0.000 272

4 23 cMEM 20 5.839 0.000 171

4 23 cMEM 30 5.755 0.000 127

4 23 cMEM 40 4.940 0.000 76

4 23 cMEM 50 3.654 0.000 43

4 23 cMEM 60 0.867 0.000 27

4 23 cMEM 70 1.038 0.000 16

4 23 cMEM 80 0.000 0.000 7

4 23 cMEM 90 0.000 0.000 3

4 23 cMEM 100 0.000 0.000 1

4 23 dSPM 0 26.883 0.000 8002

4 23 dSPM 10 24.575 0.000 2760

4 23 dSPM 20 19.581 0.000 1101

4 23 dSPM 30 15.057 0.000 539

4 23 dSPM 40 10.741 0.000 264

4 23 dSPM 50 6.433 0.000 138

4 23 dSPM 60 1.034 0.000 75

4 23 dSPM 70 0.799 0.000 30

4 23 dSPM 80 0.000 0.000 15

4 23 dSPM 90 0.000 0.000 5

4 23 dSPM 100 0.000 0.000 1

4 23 MNE 0 23.703 0.000 8002

4 23 MNE 10 18.175 0.000 915

4 23 MNE 20 10.183 0.000 287

4 23 MNE 30 3.939 0.000 131

4 23 MNE 40 1.775 0.000 69

4 23 MNE 50 1.479 0.000 39

4 23 MNE 60 0.000 0.000 21

4 23 MNE 70 0.000 0.000 10

4 23 MNE 80 0.000 0.000 7

4 23 MNE 90 0.000 0.000 2

4 23 MNE 100 0.000 0.000 1

4 23 sLORETA 0 23.013 0.000 8002

4 23 sLORETA 10 19.977 0.000 2017

4 23 sLORETA 20 14.908 0.000 844

4 23 sLORETA 30 9.272 0.000 401

4 23 sLORETA 40 3.937 0.000 214

4 23 sLORETA 50 0.964 0.000 128

4 23 sLORETA 60 0.924 0.000 81

4 23 sLORETA 70 0.605 0.000 46

4 23 sLORETA 80 0.000 0.000 21

4 23 sLORETA 90 0.000 0.000 5

4 23 sLORETA 100 0.000 0.000 1

4 24 Ave 0 24.364 0.000 8002

4 24 Ave 10 21.959 0.000 2044

4 24 Ave 20 17.963 0.000 970

4 24 Ave 30 14.813 0.000 488

4 24 Ave 40 13.623 0.000 246

4 24 Ave 50 12.061 0.000 131

4 24 Ave 60 10.039 0.000 62

4 24 Ave 70 7.673 0.000 29

4 24 Ave 80 3.330 0.000 14

4 24 Ave 90 0.000 0.000 6

4 24 Ave 100 0.000 0.000 1

4 24 cMEM 0 7.013 0.000 8002

4 24 cMEM 10 6.496 0.000 158

4 24 cMEM 20 5.910 0.000 86

4 24 cMEM 30 5.401 0.000 63

4 24 cMEM 40 4.416 0.000 46

4 24 cMEM 50 3.983 0.000 37

4 24 cMEM 60 3.790 0.000 31

4 24 cMEM 70 3.419 0.000 24

4 24 cMEM 80 2.279 0.000 12

4 24 cMEM 90 1.072 0.000 8

4 24 cMEM 100 0.000 0.000 1

4 24 dSPM 0 27.350 0.000 8002

4 24 dSPM 10 26.024 0.000 2731

4 24 dSPM 20 23.164 0.000 1406

4 24 dSPM 30 20.415 0.000 819

4 24 dSPM 40 18.817 0.000 488

4 24 dSPM 50 16.321 0.000 236

4 24 dSPM 60 13.367 0.000 118

4 24 dSPM 70 8.682 0.000 53

4 24 dSPM 80 7.972 0.000 22

4 24 dSPM 90 8.500 0.000 6

4 24 dSPM 100 9.286 9.286 1

4 24 MNE 0 24.826 0.000 8002

4 24 MNE 10 22.219 0.000 1118

4 24 MNE 20 19.244 0.000 462

4 24 MNE 30 16.605 0.000 213

4 24 MNE 40 16.419 0.000 117

4 24 MNE 50 15.419 0.000 67

4 24 MNE 60 13.343 0.000 32

4 24 MNE 70 12.908 0.000 22

4 24 MNE 80 12.309 0.000 11

4 24 MNE 90 9.476 0.000 5

4 24 MNE 100 6.631 6.631 1

4 24 sLORETA 0 23.118 0.000 8002

4 24 sLORETA 10 21.171 0.000 2169

4 24 sLORETA 20 17.632 0.000 1102

4 24 sLORETA 30 14.906 0.000 650

4 24 sLORETA 40 13.548 0.000 369

4 24 sLORETA 50 11.679 0.000 189

4 24 sLORETA 60 9.070 0.000 99

4 24 sLORETA 70 6.570 0.000 52

4 24 sLORETA 80 2.556 0.000 21

4 24 sLORETA 90 0.000 0.000 9

4 24 sLORETA 100 0.000 0.000 1

4 25 Ave 0 26.756 0.000 8002

4 25 Ave 10 24.230 0.000 2112

4 25 Ave 20 20.315 0.000 945

4 25 Ave 30 14.680 0.000 454

4 25 Ave 40 2.693 0.000 179

4 25 Ave 50 1.179 0.000 93

4 25 Ave 60 0.613 0.000 41

4 25 Ave 70 0.000 0.000 16

4 25 Ave 80 0.000 0.000 9

4 25 Ave 90 0.000 0.000 3

4 25 Ave 100 0.000 0.000 1

4 25 cMEM 0 13.700 0.000 8002

4 25 cMEM 10 9.149 0.000 175

4 25 cMEM 20 0.477 0.000 63

4 25 cMEM 30 0.000 0.000 39

4 25 cMEM 40 0.000 0.000 19

4 25 cMEM 50 0.000 0.000 8

4 25 cMEM 60 0.000 0.000 4

4 25 cMEM 70 0.000 0.000 3

4 25 cMEM 80 0.000 0.000 1

4 25 cMEM 90 0.000 0.000 1

4 25 cMEM 100 0.000 0.000 1

4 25 dSPM 0 29.916 0.000 8002

4 25 dSPM 10 28.738 0.000 3277

4 25 dSPM 20 26.507 0.000 1693

4 25 dSPM 30 24.155 0.000 1008

4 25 dSPM 40 21.373 0.000 609

4 25 dSPM 50 18.399 0.000 324

4 25 dSPM 60 16.526 0.000 160

4 25 dSPM 70 9.390 0.000 66

4 25 dSPM 80 2.038 0.000 31

4 25 dSPM 90 1.797 0.000 13

4 25 dSPM 100 0.000 0.000 1

4 25 MNE 0 26.656 0.000 8002

4 25 MNE 10 22.489 0.000 1121

4 25 MNE 20 17.002 0.000 396

4 25 MNE 30 11.520 0.000 160

4 25 MNE 40 2.727 0.000 75

4 25 MNE 50 1.867 0.000 33

4 25 MNE 60 0.000 0.000 16

4 25 MNE 70 0.000 0.000 8

4 25 MNE 80 0.000 0.000 5

4 25 MNE 90 0.000 0.000 3

4 25 MNE 100 0.000 0.000 1

4 25 sLORETA 0 26.214 0.000 8002

4 25 sLORETA 10 24.664 0.000 2379

4 25 sLORETA 20 22.442 0.000 1240

4 25 sLORETA 30 19.485 0.000 733

4 25 sLORETA 40 14.588 0.000 407

4 25 sLORETA 50 7.933 0.000 212

4 25 sLORETA 60 2.770 0.000 117

4 25 sLORETA 70 1.504 0.000 63

4 25 sLORETA 80 0.843 0.000 26

4 25 sLORETA 90 0.000 0.000 13

4 25 sLORETA 100 0.000 0.000 1

4 26 Ave 0 25.880 0.000 8002

4 26 Ave 10 22.989 0.000 2052

4 26 Ave 20 17.551 0.000 804

4 26 Ave 30 12.096 0.000 374

4 26 Ave 40 4.388 0.000 163

4 26 Ave 50 1.226 0.000 103

4 26 Ave 60 0.946 0.000 71

4 26 Ave 70 0.000 0.000 30

4 26 Ave 80 0.000 0.000 15

4 26 Ave 90 0.000 0.000 3

4 26 Ave 100 0.000 0.000 1

4 26 cMEM 0 7.098 0.000 8002

4 26 cMEM 10 2.620 0.000 109

4 26 cMEM 20 1.596 0.000 57

4 26 cMEM 30 1.331 0.000 32

4 26 cMEM 40 1.239 0.000 18

4 26 cMEM 50 1.421 0.000 10

4 26 cMEM 60 0.000 0.000 5

4 26 cMEM 70 0.000 0.000 4

4 26 cMEM 80 0.000 0.000 1

4 26 cMEM 90 0.000 0.000 1

4 26 cMEM 100 0.000 0.000 1

4 26 dSPM 0 30.977 0.000 8002

4 26 dSPM 10 29.725 0.000 2816

4 26 dSPM 20 27.239 0.000 1348

4 26 dSPM 30 23.962 0.000 689

4 26 dSPM 40 20.067 0.000 346

4 26 dSPM 50 16.863 0.000 171

4 26 dSPM 60 13.243 0.000 82

4 26 dSPM 70 6.342 0.000 43

4 26 dSPM 80 1.667 0.000 16

4 26 dSPM 90 0.000 0.000 5

4 26 dSPM 100 0.000 0.000 1

4 26 MNE 0 23.178 0.000 8002

4 26 MNE 10 16.534 0.000 847

4 26 MNE 20 8.594 0.000 234

4 26 MNE 30 2.184 0.000 117

4 26 MNE 40 1.389 0.000 67

4 26 MNE 50 0.000 0.000 33

4 26 MNE 60 0.000 0.000 17

4 26 MNE 70 0.000 0.000 8

4 26 MNE 80 0.000 0.000 5

4 26 MNE 90 0.000 0.000 4

4 26 MNE 100 0.000 0.000 1

4 26 sLORETA 0 25.069 0.000 8002

4 26 sLORETA 10 22.692 0.000 2066

4 26 sLORETA 20 19.368 0.000 946

4 26 sLORETA 30 15.234 0.000 521

4 26 sLORETA 40 7.995 0.000 248

4 26 sLORETA 50 1.925 0.000 136

4 26 sLORETA 60 1.398 0.000 86

4 26 sLORETA 70 1.210 0.000 43

4 26 sLORETA 80 0.920 0.000 21

4 26 sLORETA 90 0.000 0.000 7

4 26 sLORETA 100 0.000 0.000 1

5 27 Ave 0 51.033 0.000 8002

5 27 Ave 10 51.012 0.000 4794

5 27 Ave 20 51.431 0.000 2397

5 27 Ave 30 53.537 0.000 904

5 27 Ave 40 58.124 0.000 285

5 27 Ave 50 66.853 0.000 101

5 27 Ave 60 75.694 37.788 31

5 27 Ave 70 79.410 71.655 11

5 27 Ave 80 81.585 77.494 5

5 27 Ave 90 80.101 77.494 3

5 27 Ave 100 79.419 79.419 1

5 27 cMEM 0 57.997 0.000 8002

5 27 cMEM 10 59.584 0.000 734

5 27 cMEM 20 62.100 0.000 287

5 27 cMEM 30 64.630 0.000 124

5 27 cMEM 40 67.440 12.022 66

5 27 cMEM 50 72.637 14.872 30

5 27 cMEM 60 78.095 15.772 12

5 27 cMEM 70 81.063 77.179 8

5 27 cMEM 80 81.224 77.179 7

5 27 cMEM 90 82.510 79.419 3

5 27 cMEM 100 83.290 83.290 1

5 27 dSPM 0 47.020 0.000 8002

5 27 dSPM 10 46.925 0.000 5306

5 27 dSPM 20 46.538 0.000 3235

5 27 dSPM 30 46.056 0.000 1774

5 27 dSPM 40 45.677 0.000 936

5 27 dSPM 50 44.022 0.000 450

5 27 dSPM 60 41.720 0.000 205

5 27 dSPM 70 30.537 0.000 59

5 27 dSPM 80 22.283 4.268 17

5 27 dSPM 90 15.162 9.555 5

5 27 dSPM 100 18.023 18.023 1

5 27 MNE 0 55.303 0.000 8002

5 27 MNE 10 55.709 0.000 3035

5 27 MNE 20 57.684 0.000 1115

5 27 MNE 30 61.186 0.000 391

5 27 MNE 40 66.578 0.000 137

5 27 MNE 50 71.230 0.000 65

5 27 MNE 60 76.980 33.502 30

5 27 MNE 70 79.420 37.788 11

5 27 MNE 80 81.799 72.677 6

5 27 MNE 90 82.258 77.494 2

5 27 MNE 100 77.494 77.494 1

5 27 sLORETA 0 52.005 0.000 8002

5 27 sLORETA 10 52.024 0.000 5397

5 27 sLORETA 20 52.287 0.000 3244

5 27 sLORETA 30 53.556 0.000 1600

5 27 sLORETA 40 56.247 0.000 703

5 27 sLORETA 50 60.016 0.000 301

5 27 sLORETA 60 64.831 0.000 139

5 27 sLORETA 70 67.185 37.477 73

5 27 sLORETA 80 71.788 48.365 24

5 27 sLORETA 90 81.600 77.494 4

5 27 sLORETA 100 85.393 85.393 1

5 28 Ave 0 42.015 0.000 8002

5 28 Ave 10 41.697 0.000 5181

5 28 Ave 20 40.286 0.000 2949

5 28 Ave 30 37.950 0.000 1587

5 28 Ave 40 34.422 0.000 722

5 28 Ave 50 29.900 0.000 286

5 28 Ave 60 24.267 0.000 106

5 28 Ave 70 20.393 0.000 40

5 28 Ave 80 18.401 12.374 10

5 28 Ave 90 17.354 12.374 4

5 28 Ave 100 16.200 16.200 1

5 28 cMEM 0 39.556 0.000 8002

5 28 cMEM 10 38.862 0.000 1348

5 28 cMEM 20 36.874 0.000 475

5 28 cMEM 30 35.345 0.000 194

5 28 cMEM 40 33.564 0.000 102

5 28 cMEM 50 31.819 0.000 55

5 28 cMEM 60 27.430 5.597 29

5 28 cMEM 70 13.222 5.674 14

5 28 cMEM 80 8.304 6.314 5

5 28 cMEM 90 6.445 6.314 2

5 28 cMEM 100 6.314 6.314 1

5 28 dSPM 0 40.879 0.000 8002

5 28 dSPM 10 40.573 0.000 5122

5 28 dSPM 20 39.386 0.000 3118

5 28 dSPM 30 37.761 0.000 1839

5 28 dSPM 40 36.280 0.000 1045

5 28 dSPM 50 34.829 0.000 565

5 28 dSPM 60 31.687 0.000 261

5 28 dSPM 70 28.517 0.000 103

5 28 dSPM 80 28.543 0.000 39

5 28 dSPM 90 23.588 7.218 6

5 28 dSPM 100 20.784 20.784 1

5 28 MNE 0 43.950 0.000 8002

5 28 MNE 10 43.102 0.000 2645

5 28 MNE 20 39.849 0.000 868

5 28 MNE 30 35.164 0.000 301

5 28 MNE 40 30.816 0.000 109

5 28 MNE 50 26.842 0.000 38

5 28 MNE 60 21.620 12.374 11

5 28 MNE 70 24.336 16.200 5

5 28 MNE 80 17.515 16.200 2

5 28 MNE 90 18.469 18.469 1

5 28 MNE 100 18.469 18.469 1

5 28 sLORETA 0 42.611 0.000 8002

5 28 sLORETA 10 42.288 0.000 5103

5 28 sLORETA 20 40.851 0.000 2749

5 28 sLORETA 30 38.509 0.000 1420

5 28 sLORETA 40 34.484 0.000 594

5 28 sLORETA 50 30.851 0.000 263

5 28 sLORETA 60 30.632 0.000 103

5 28 sLORETA 70 23.236 0.000 35

5 28 sLORETA 80 15.309 0.000 13

5 28 sLORETA 90 17.194 12.374 5

5 28 sLORETA 100 18.469 18.469 1

5 29 Ave 0 42.042 0.000 8002

5 29 Ave 10 41.825 0.000 5593

5 29 Ave 20 40.652 0.000 3466

5 29 Ave 30 38.415 0.000 2002

5 29 Ave 40 35.189 0.000 1024

5 29 Ave 50 31.048 0.000 464

5 29 Ave 60 26.383 0.000 186

5 29 Ave 70 20.414 0.000 59

5 29 Ave 80 14.356 0.000 17

5 29 Ave 90 15.669 0.000 5

5 29 Ave 100 0.000 0.000 1

5 29 cMEM 0 21.187 0.000 8002

5 29 cMEM 10 18.200 0.000 417

5 29 cMEM 20 15.674 0.000 219

5 29 cMEM 30 13.522 0.000 142

5 29 cMEM 40 11.734 0.000 80

5 29 cMEM 50 11.026 0.000 50

5 29 cMEM 60 10.111 0.000 26

5 29 cMEM 70 7.546 0.000 5

5 29 cMEM 80 1.608 0.000 2

5 29 cMEM 90 1.608 0.000 2

5 29 cMEM 100 2.256 2.256 1

5 29 dSPM 0 41.234 0.000 8002

5 29 dSPM 10 40.817 0.000 4968

5 29 dSPM 20 39.339 0.000 2733

5 29 dSPM 30 37.672 0.000 1427

5 29 dSPM 40 36.212 0.000 683

5 29 dSPM 50 34.216 0.000 275

5 29 dSPM 60 31.586 9.136 115

5 29 dSPM 70 33.366 9.136 44

5 29 dSPM 80 32.796 9.136 16

5 29 dSPM 90 29.297 13.182 4

5 29 dSPM 100 13.182 13.182 1

5 29 MNE 0 43.179 0.000 8002

5 29 MNE 10 42.591 0.000 3377

5 29 MNE 20 39.922 0.000 1398

5 29 MNE 30 37.262 0.000 620

5 29 MNE 40 31.770 0.000 245

5 29 MNE 50 26.307 0.000 100

5 29 MNE 60 22.115 0.000 45

5 29 MNE 70 17.400 0.000 20

5 29 MNE 80 14.224 0.000 7

5 29 MNE 90 13.019 0.000 4

5 29 MNE 100 0.000 0.000 1

5 29 sLORETA 0 43.142 0.000 8002

5 29 sLORETA 10 42.956 0.000 5698

5 29 sLORETA 20 42.052 0.000 3691

5 29 sLORETA 30 40.464 0.000 2212

5 29 sLORETA 40 38.196 0.000 1192

5 29 sLORETA 50 35.580 0.000 578

5 29 sLORETA 60 35.531 0.000 238

5 29 sLORETA 70 33.872 0.000 90

5 29 sLORETA 80 29.297 0.000 17

5 29 sLORETA 90 0.000 0.000 2

5 29 sLORETA 100 0.000 0.000 1

5 30 Ave 0 43.426 0.000 8002

5 30 Ave 10 43.022 0.000 4665

5 30 Ave 20 40.849 0.000 2113

5 30 Ave 30 36.832 0.000 785

5 30 Ave 40 33.933 0.000 362

5 30 Ave 50 32.544 0.000 160

5 30 Ave 60 31.805 0.000 73

5 30 Ave 70 31.722 15.800 26

5 30 Ave 80 32.753 28.350 13

5 30 Ave 90 33.191 29.748 6

5 30 Ave 100 36.955 36.955 1

5 30 cMEM 0 36.826 0.000 8002

5 30 cMEM 10 35.578 5.568 418

5 30 cMEM 20 34.260 14.259 137

5 30 cMEM 30 33.832 19.209 83

5 30 cMEM 40 33.800 19.209 65

5 30 cMEM 50 33.934 23.491 38

5 30 cMEM 60 34.050 23.491 22

5 30 cMEM 70 33.349 28.350 7

5 30 cMEM 80 32.893 29.708 2

5 30 cMEM 90 34.890 34.890 1

5 30 cMEM 100 34.890 34.890 1

5 30 dSPM 0 43.247 0.000 8002

5 30 dSPM 10 42.900 0.000 4945

5 30 dSPM 20 41.549 0.000 2637

5 30 dSPM 30 39.142 0.000 1221

5 30 dSPM 40 36.756 0.000 540

5 30 dSPM 50 34.448 0.000 221

5 30 dSPM 60 33.160 8.166 84

5 30 dSPM 70 33.519 9.899 37

5 30 dSPM 80 33.094 9.899 12

5 30 dSPM 90 34.202 31.540 5

5 30 dSPM 100 39.168 39.168 1

5 30 MNE 0 45.522 0.000 8002

5 30 MNE 10 45.070 0.000 2967

5 30 MNE 20 43.348 0.000 1251

5 30 MNE 30 41.597 0.000 574

5 30 MNE 40 40.173 0.000 276

5 30 MNE 50 39.891 0.000 131

5 30 MNE 60 41.009 0.000 58

5 30 MNE 70 40.734 0.000 29

5 30 MNE 80 38.475 0.000 11

5 30 MNE 90 33.491 29.748 5

5 30 MNE 100 30.693 30.693 1

5 30 sLORETA 0 43.439 0.000 8002

5 30 sLORETA 10 43.161 0.000 5099

5 30 sLORETA 20 41.702 0.000 2697

5 30 sLORETA 30 38.400 0.000 1164

5 30 sLORETA 40 35.119 0.000 522

5 30 sLORETA 50 31.090 0.000 263

5 30 sLORETA 60 28.910 0.000 125

5 30 sLORETA 70 28.650 0.000 47

5 30 sLORETA 80 30.130 11.025 16

5 30 sLORETA 90 27.842 15.800 2

5 30 sLORETA 100 35.825 35.825 1

6 31 Ave 0 34.057 0.000 8002

6 31 Ave 10 31.553 0.000 2341

6 31 Ave 20 24.272 0.000 808

6 31 Ave 30 16.399 0.000 363

6 31 Ave 40 13.626 0.000 212

6 31 Ave 50 11.886 0.000 106

6 31 Ave 60 9.048 0.000 51

6 31 Ave 70 6.560 0.000 17

6 31 Ave 80 1.643 0.000 11

6 31 Ave 90 0.000 0.000 3

6 31 Ave 100 0.000 0.000 1

6 31 cMEM 0 4.134 0.000 8002

6 31 cMEM 10 0.000 0.000 38

6 31 cMEM 20 0.000 0.000 28

6 31 cMEM 30 0.000 0.000 22

6 31 cMEM 40 0.000 0.000 16

6 31 cMEM 50 0.000 0.000 12

6 31 cMEM 60 0.000 0.000 11

6 31 cMEM 70 0.000 0.000 8

6 31 cMEM 80 0.000 0.000 7

6 31 cMEM 90 0.000 0.000 3

6 31 cMEM 100 0.000 0.000 1

6 31 dSPM 0 35.142 0.000 8002

6 31 dSPM 10 34.037 0.000 3318

6 31 dSPM 20 29.428 0.000 1517

6 31 dSPM 30 20.084 0.000 669

6 31 dSPM 40 14.005 0.000 358

6 31 dSPM 50 11.926 0.000 225

6 31 dSPM 60 7.702 0.000 105

6 31 dSPM 70 4.993 0.000 57

6 31 dSPM 80 3.934 0.000 21

6 31 dSPM 90 3.032 0.000 9

6 31 dSPM 100 3.462 3.462 1

6 31 MNE 0 36.178 0.000 8002

6 31 MNE 10 33.244 0.000 1339

6 31 MNE 20 28.167 0.000 491

6 31 MNE 30 22.961 0.000 216

6 31 MNE 40 20.356 0.000 112

6 31 MNE 50 17.756 0.000 64

6 31 MNE 60 17.518 0.000 29

6 31 MNE 70 16.085 0.000 20

6 31 MNE 80 19.563 0.000 7

6 31 MNE 90 16.436 0.000 4

6 31 MNE 100 0.000 0.000 1

6 31 sLORETA 0 33.458 0.000 8002

6 31 sLORETA 10 31.338 0.000 2320

6 31 sLORETA 20 26.127 0.000 936

6 31 sLORETA 30 18.717 0.000 474

6 31 sLORETA 40 14.652 0.000 287

6 31 sLORETA 50 13.473 0.000 157

6 31 sLORETA 60 10.746 0.000 77

6 31 sLORETA 70 6.655 0.000 36

6 31 sLORETA 80 3.333 0.000 13

6 31 sLORETA 90 3.404 0.000 6

6 31 sLORETA 100 3.462 3.462 1

6 32 Ave 0 50.140 0.000 8002

6 32 Ave 10 50.141 0.000 3954

6 32 Ave 20 50.095 0.000 2097

6 32 Ave 30 48.708 0.000 1047

6 32 Ave 40 44.996 0.000 454

6 32 Ave 50 38.303 0.000 189

6 32 Ave 60 31.302 0.000 78

6 32 Ave 70 23.835 0.000 30

6 32 Ave 80 26.075 2.224 10

6 32 Ave 90 30.292 30.292 1

6 32 Ave 100 30.292 30.292 1

6 32 cMEM 0 5.136 0.000 8002

6 32 cMEM 10 3.238 0.000 92

6 32 cMEM 20 0.000 0.000 35

6 32 cMEM 30 0.000 0.000 23

6 32 cMEM 40 0.000 0.000 22

6 32 cMEM 50 0.000 0.000 17

6 32 cMEM 60 0.000 0.000 12

6 32 cMEM 70 0.000 0.000 10

6 32 cMEM 80 0.000 0.000 7

6 32 cMEM 90 0.000 0.000 2

6 32 cMEM 100 0.000 0.000 1

6 32 dSPM 0 51.812 0.000 8002

6 32 dSPM 10 51.821 0.000 4668

6 32 dSPM 20 51.973 0.000 3041

6 32 dSPM 30 52.195 0.000 1930

6 32 dSPM 40 52.442 0.000 1190

6 32 dSPM 50 52.530 0.000 670

6 32 dSPM 60 52.088 0.000 332

6 32 dSPM 70 47.454 0.000 150

6 32 dSPM 80 30.928 0.000 43

6 32 dSPM 90 21.312 0.000 10

6 32 dSPM 100 23.825 23.825 1

6 32 MNE 0 51.564 0.000 8002

6 32 MNE 10 51.461 0.000 1827

6 32 MNE 20 50.857 0.000 692

6 32 MNE 30 49.085 0.000 274

6 32 MNE 40 46.859 0.000 107

6 32 MNE 50 43.495 0.000 39

6 32 MNE 60 34.209 0.000 16

6 32 MNE 70 26.594 19.260 6

6 32 MNE 80 29.695 28.821 2

6 32 MNE 90 30.292 30.292 1

6 32 MNE 100 30.292 30.292 1

6 32 sLORETA 0 48.197 0.000 8002

6 32 sLORETA 10 48.124 0.000 3746

6 32 sLORETA 20 47.527 0.000 1986

6 32 sLORETA 30 45.636 0.000 1046

6 32 sLORETA 40 41.811 0.000 516

6 32 sLORETA 50 37.567 0.000 253

6 32 sLORETA 60 29.756 0.000 113

6 32 sLORETA 70 25.756 0.000 53

6 32 sLORETA 80 24.634 0.000 20

6 32 sLORETA 90 22.627 2.224 8

6 32 sLORETA 100 30.292 30.292 1

6 33 Ave 0 48.650 0.000 8002

6 33 Ave 10 48.583 0.000 4038

6 33 Ave 20 48.339 0.000 2249

6 33 Ave 30 47.033 0.000 1234

6 33 Ave 40 43.852 0.000 665

6 33 Ave 50 41.228 0.000 375

6 33 Ave 60 37.705 0.000 179

6 33 Ave 70 32.379 0.000 76

6 33 Ave 80 29.484 0.000 28

6 33 Ave 90 7.993 0.000 9

6 33 Ave 100 3.662 3.662 1

6 33 cMEM 0 14.187 0.000 8002

6 33 cMEM 10 7.409 0.000 161

6 33 cMEM 20 3.004 0.000 38

6 33 cMEM 30 0.000 0.000 22

6 33 cMEM 40 0.000 0.000 21

6 33 cMEM 50 0.000 0.000 15

6 33 cMEM 60 0.000 0.000 13

6 33 cMEM 70 0.000 0.000 10

6 33 cMEM 80 0.000 0.000 8

6 33 cMEM 90 0.000 0.000 4

6 33 cMEM 100 0.000 0.000 1

6 33 dSPM 0 50.332 0.000 8002

6 33 dSPM 10 50.285 0.000 4370

6 33 dSPM 20 50.381 0.000 2707

6 33 dSPM 30 50.150 0.000 1667

6 33 dSPM 40 49.413 0.000 1001

6 33 dSPM 50 47.842 0.000 543

6 33 dSPM 60 43.880 0.000 256

6 33 dSPM 70 33.582 0.000 104

6 33 dSPM 80 15.083 0.000 31

6 33 dSPM 90 4.483 0.000 11

6 33 dSPM 100 6.114 6.114 1

6 33 MNE 0 52.282 0.000 8002

6 33 MNE 10 52.288 0.000 2226

6 33 MNE 20 52.102 0.000 987

6 33 MNE 30 52.180 0.000 448

6 33 MNE 40 52.493 0.000 228

6 33 MNE 50 54.417 0.000 87

6 33 MNE 60 55.443 0.000 30

6 33 MNE 70 60.526 0.000 11

6 33 MNE 80 67.649 28.821 4

6 33 MNE 90 65.958 30.292 2

6 33 MNE 100 30.292 30.292 1

6 33 sLORETA 0 45.139 0.000 8002

6 33 sLORETA 10 44.884 0.000 3391

6 33 sLORETA 20 43.667 0.000 1667

6 33 sLORETA 30 40.598 0.000 897

6 33 sLORETA 40 38.011 0.000 541

6 33 sLORETA 50 34.426 0.000 305

6 33 sLORETA 60 29.490 0.000 129

6 33 sLORETA 70 23.357 0.000 57

6 33 sLORETA 80 14.530 0.000 23

6 33 sLORETA 90 13.369 0.000 9

6 33 sLORETA 100 6.114 6.114 1

6 34 Ave 0 47.312 0.000 8002

6 34 Ave 10 47.225 0.000 4670

6 34 Ave 20 46.795 0.000 2801

6 34 Ave 30 45.803 0.000 1562

6 34 Ave 40 43.713 0.000 872

6 34 Ave 50 37.039 0.000 455

6 34 Ave 60 33.928 0.000 237

6 34 Ave 70 31.459 0.000 122

6 34 Ave 80 29.067 0.000 45

6 34 Ave 90 24.420 16.571 7

6 34 Ave 100 18.582 18.582 1

6 34 cMEM 0 38.720 0.000 8002

6 34 cMEM 10 38.261 0.000 425

6 34 cMEM 20 36.405 0.000 166

6 34 cMEM 30 31.133 0.000 62

6 34 cMEM 40 22.627 0.000 28

6 34 cMEM 50 10.731 0.000 17

6 34 cMEM 60 0.000 0.000 13

6 34 cMEM 70 0.000 0.000 11

6 34 cMEM 80 0.000 0.000 9

6 34 cMEM 90 0.000 0.000 4

6 34 cMEM 100 0.000 0.000 1

6 34 dSPM 0 48.993 0.000 8002

6 34 dSPM 10 48.955 0.000 4695

6 34 dSPM 20 48.869 0.000 2930

6 34 dSPM 30 48.524 0.000 1815

6 34 dSPM 40 47.953 0.000 1059

6 34 dSPM 50 45.867 0.000 584

6 34 dSPM 60 42.391 0.000 292

6 34 dSPM 70 34.398 2.939 101

6 34 dSPM 80 37.247 14.444 41

6 34 dSPM 90 34.199 16.701 11

6 34 dSPM 100 34.293 34.293 1

6 34 MNE 0 49.852 0.000 8002

6 34 MNE 10 49.772 0.000 3345

6 34 MNE 20 49.050 0.000 1589

6 34 MNE 30 48.289 0.000 867

6 34 MNE 40 47.734 0.000 456

6 34 MNE 50 47.559 0.000 223

6 34 MNE 60 45.225 0.000 109

6 34 MNE 70 48.279 0.000 41

6 34 MNE 80 53.055 0.000 14

6 34 MNE 90 36.392 0.000 2

6 34 MNE 100 0.000 0.000 1

6 34 sLORETA 0 44.335 0.000 8002

6 34 sLORETA 10 44.128 0.000 4275

6 34 sLORETA 20 43.186 0.000 2303

6 34 sLORETA 30 40.940 0.000 1200

6 34 sLORETA 40 37.539 0.000 667

6 34 sLORETA 50 33.759 0.000 395

6 34 sLORETA 60 31.683 0.000 246

6 34 sLORETA 70 31.876 0.000 146

6 34 sLORETA 80 29.822 2.939 60

6 34 sLORETA 90 25.852 16.571 11

6 34 sLORETA 100 18.582 18.582 1

6 35 Ave 0 40.525 0.000 8002

6 35 Ave 10 39.843 0.000 3369

6 35 Ave 20 37.336 0.000 1618

6 35 Ave 30 33.076 0.000 801

6 35 Ave 40 27.987 0.000 431

6 35 Ave 50 24.397 0.000 270

6 35 Ave 60 20.754 0.000 155

6 35 Ave 70 19.643 0.000 92

6 35 Ave 80 17.677 0.000 44

6 35 Ave 90 16.560 0.000 14

6 35 Ave 100 14.695 14.695 1

6 35 cMEM 0 13.678 0.000 8002

6 35 cMEM 10 9.145 0.000 186

6 35 cMEM 20 3.128 0.000 37

6 35 cMEM 30 0.000 0.000 23

6 35 cMEM 40 0.000 0.000 19

6 35 cMEM 50 0.000 0.000 15

6 35 cMEM 60 0.000 0.000 13

6 35 cMEM 70 0.000 0.000 11

6 35 cMEM 80 0.000 0.000 7

6 35 cMEM 90 0.000 0.000 2

6 35 cMEM 100 0.000 0.000 1

6 35 dSPM 0 41.784 0.000 8002

6 35 dSPM 10 41.337 0.000 3564

6 35 dSPM 20 39.503 0.000 1863

6 35 dSPM 30 36.085 0.000 934

6 35 dSPM 40 30.613 0.000 473

6 35 dSPM 50 24.878 0.000 280

6 35 dSPM 60 18.201 0.000 149

6 35 dSPM 70 16.903 0.000 97

6 35 dSPM 80 15.148 0.000 46

6 35 dSPM 90 15.819 4.897 18

6 35 dSPM 100 14.695 14.695 1

6 35 MNE 0 44.491 0.000 8002

6 35 MNE 10 43.641 0.000 2190

6 35 MNE 20 41.408 0.000 917

6 35 MNE 30 38.530 0.000 474

6 35 MNE 40 36.007 0.000 235

6 35 MNE 50 36.874 0.000 124

6 35 MNE 60 36.108 0.000 62

6 35 MNE 70 41.847 0.000 23

6 35 MNE 80 41.489 34.122 6

6 35 MNE 90 41.210 34.411 2

6 35 MNE 100 46.576 46.576 1

6 35 sLORETA 0 37.822 0.000 8002

6 35 sLORETA 10 37.016 0.000 3104

6 35 sLORETA 20 35.028 0.000 1625

6 35 sLORETA 30 31.031 0.000 868

6 35 sLORETA 40 26.958 0.000 500

6 35 sLORETA 50 22.823 0.000 308

6 35 sLORETA 60 19.504 0.000 214

6 35 sLORETA 70 18.555 0.000 126

6 35 sLORETA 80 17.052 0.000 71

6 35 sLORETA 90 13.090 0.000 25

6 35 sLORETA 100 14.695 14.695 1

7 36 Ave 0 37.257 0.000 8002

7 36 Ave 10 35.865 0.000 3690

7 36 Ave 20 30.839 0.000 1571

7 36 Ave 30 25.000 0.000 673

7 36 Ave 40 17.015 0.000 254

7 36 Ave 50 11.417 0.000 115

7 36 Ave 60 6.725 0.000 60

7 36 Ave 70 5.486 0.000 29

7 36 Ave 80 0.000 0.000 10

7 36 Ave 90 0.000 0.000 2

7 36 Ave 100 0.000 0.000 1

7 36 cMEM 0 14.161 0.000 8002

7 36 cMEM 10 11.088 0.000 458

7 36 cMEM 20 9.686 0.000 210

7 36 cMEM 30 8.072 0.000 109

7 36 cMEM 40 6.968 0.000 53

7 36 cMEM 50 6.829 0.000 26

7 36 cMEM 60 5.310 0.000 11

7 36 cMEM 70 1.642 0.000 5

7 36 cMEM 80 0.000 0.000 3

7 36 cMEM 90 0.000 0.000 2

7 36 cMEM 100 0.000 0.000 1

7 36 dSPM 0 37.436 0.000 8002

7 36 dSPM 10 36.332 0.000 3919

7 36 dSPM 20 32.662 0.000 1807

7 36 dSPM 30 28.708 0.000 826

7 36 dSPM 40 25.029 0.000 388

7 36 dSPM 50 21.856 0.000 176

7 36 dSPM 60 17.881 0.000 76

7 36 dSPM 70 16.823 0.000 33

7 36 dSPM 80 3.489 0.000 9

7 36 dSPM 90 0.000 0.000 5

7 36 dSPM 100 0.000 0.000 1

7 36 MNE 0 40.555 0.000 8002

7 36 MNE 10 38.450 0.000 1874

7 36 MNE 20 33.264 0.000 564

7 36 MNE 30 28.102 0.000 235

7 36 MNE 40 24.840 0.000 76

7 36 MNE 50 15.386 0.000 32

7 36 MNE 60 13.208 0.000 15

7 36 MNE 70 14.984 0.000 6

7 36 MNE 80 15.752 0.000 3

7 36 MNE 90 0.000 0.000 1

7 36 MNE 100 0.000 0.000 1

7 36 sLORETA 0 37.916 0.000 8002

7 36 sLORETA 10 36.882 0.000 4070

7 36 sLORETA 20 32.850 0.000 1863

7 36 sLORETA 30 27.900 0.000 857

7 36 sLORETA 40 21.968 0.000 401

7 36 sLORETA 50 16.972 0.000 173

7 36 sLORETA 60 11.959 0.000 82

7 36 sLORETA 70 7.535 0.000 40

7 36 sLORETA 80 0.000 0.000 13

7 36 sLORETA 90 0.000 0.000 3

7 36 sLORETA 100 0.000 0.000 1

7 37 Ave 0 37.586 0.000 8002

7 37 Ave 10 36.340 0.000 4040

7 37 Ave 20 31.471 0.000 1713

7 37 Ave 30 25.564 0.000 770

7 37 Ave 40 16.708 0.000 277

7 37 Ave 50 9.706 0.000 124

7 37 Ave 60 7.541 0.000 68

7 37 Ave 70 7.365 0.000 36

7 37 Ave 80 4.847 0.000 16

7 37 Ave 90 0.000 0.000 4

7 37 Ave 100 0.000 0.000 1

7 37 cMEM 0 16.996 0.000 8002

7 37 cMEM 10 15.116 0.000 368

7 37 cMEM 20 12.870 0.000 162

7 37 cMEM 30 9.861 0.000 66

7 37 cMEM 40 9.470 0.000 36

7 37 cMEM 50 8.316 0.000 15

7 37 cMEM 60 5.506 0.000 6

7 37 cMEM 70 6.863 6.103 2

7 37 cMEM 80 6.863 6.103 2

7 37 cMEM 90 6.863 6.103 2

7 37 cMEM 100 6.103 6.103 1

7 37 dSPM 0 37.788 0.000 8002

7 37 dSPM 10 36.980 0.000 4348

7 37 dSPM 20 33.933 0.000 2135

7 37 dSPM 30 30.440 0.000 1089

7 37 dSPM 40 27.413 0.000 578

7 37 dSPM 50 25.297 0.000 306

7 37 dSPM 60 21.444 0.000 130

7 37 dSPM 70 17.223 0.000 61

7 37 dSPM 80 7.709 0.000 26

7 37 dSPM 90 5.993 0.000 7

7 37 dSPM 100 7.924 7.924 1

7 37 MNE 0 40.352 0.000 8002

7 37 MNE 10 38.090 0.000 2016

7 37 MNE 20 32.002 0.000 562

7 37 MNE 30 25.775 0.000 209

7 37 MNE 40 17.839 0.000 84

7 37 MNE 50 13.541 0.000 45

7 37 MNE 60 9.319 0.000 18

7 37 MNE 70 0.000 0.000 7

7 37 MNE 80 0.000 0.000 4

7 37 MNE 90 0.000 0.000 3

7 37 MNE 100 0.000 0.000 1

7 37 sLORETA 0 38.554 0.000 8002

7 37 sLORETA 10 37.673 0.000 4422

7 37 sLORETA 20 33.982 0.000 2118

7 37 sLORETA 30 28.320 0.000 962

7 37 sLORETA 40 22.008 0.000 427

7 37 sLORETA 50 14.009 0.000 182

7 37 sLORETA 60 9.528 0.000 96

7 37 sLORETA 70 5.334 0.000 53

7 37 sLORETA 80 3.306 0.000 19

7 37 sLORETA 90 0.000 0.000 7

7 37 sLORETA 100 0.000 0.000 1

7 38 Ave 0 43.806 0.000 8002

7 38 Ave 10 43.051 0.000 3727

7 38 Ave 20 38.788 0.000 1276

7 38 Ave 30 32.774 0.000 469

7 38 Ave 40 24.388 0.000 181

7 38 Ave 50 15.762 0.000 78

7 38 Ave 60 11.481 0.000 45

7 38 Ave 70 11.490 2.264 30

7 38 Ave 80 10.815 2.264 15

7 38 Ave 90 9.526 2.264 6

7 38 Ave 100 7.323 7.323 1

7 38 cMEM 0 23.901 0.000 8002

7 38 cMEM 10 20.415 0.000 367

7 38 cMEM 20 17.181 0.000 164

7 38 cMEM 30 15.205 0.000 90

7 38 cMEM 40 14.821 0.000 50

7 38 cMEM 50 15.307 0.000 32

7 38 cMEM 60 16.242 7.323 16

7 38 cMEM 70 16.937 9.069 8

7 38 cMEM 80 17.882 13.307 5

7 38 cMEM 90 18.098 18.098 1

7 38 cMEM 100 18.098 18.098 1

7 38 dSPM 0 43.531 0.000 8002

7 38 dSPM 10 42.889 0.000 4027

7 38 dSPM 20 40.113 0.000 1776

7 38 dSPM 30 36.732 0.000 806

7 38 dSPM 40 30.394 0.000 338

7 38 dSPM 50 21.402 0.000 113

7 38 dSPM 60 11.682 0.000 37

7 38 dSPM 70 12.194 6.804 14

7 38 dSPM 80 12.069 8.992 4

7 38 dSPM 90 9.841 8.992 2

7 38 dSPM 100 8.992 8.992 1

7 38 MNE 0 46.067 0.000 8002

7 38 MNE 10 45.023 0.000 1985

7 38 MNE 20 39.127 0.000 537

7 38 MNE 30 30.837 0.000 186

7 38 MNE 40 25.796 0.000 89

7 38 MNE 50 15.822 0.000 41

7 38 MNE 60 10.320 0.000 22

7 38 MNE 70 8.981 2.264 8

7 38 MNE 80 8.510 2.264 6

7 38 MNE 90 6.513 2.264 2

7 38 MNE 100 2.264 2.264 1

7 38 sLORETA 0 46.118 0.000 8002

7 38 sLORETA 10 45.773 0.000 4311

7 38 sLORETA 20 43.895 0.000 1807

7 38 sLORETA 30 40.486 0.000 725

7 38 sLORETA 40 37.052 0.000 347

7 38 sLORETA 50 33.202 0.000 179

7 38 sLORETA 60 30.078 0.000 103

7 38 sLORETA 70 24.003 2.264 49

7 38 sLORETA 80 15.131 2.264 20

7 38 sLORETA 90 10.877 7.961 8

7 38 sLORETA 100 10.137 10.137 1

7 39 Ave 0 42.491 0.000 8002

7 39 Ave 10 42.063 0.000 4677

7 39 Ave 20 40.073 0.000 2449

7 39 Ave 30 37.228 0.000 1253

7 39 Ave 40 34.781 0.000 674

7 39 Ave 50 31.858 0.000 346

7 39 Ave 60 29.369 0.000 143

7 39 Ave 70 26.219 0.000 52

7 39 Ave 80 25.377 0.000 19

7 39 Ave 90 29.032 0.000 6

7 39 Ave 100 0.000 0.000 1

7 39 cMEM 0 32.470 0.000 8002

7 39 cMEM 10 31.716 0.000 512

7 39 cMEM 20 31.502 0.000 238

7 39 cMEM 30 31.940 0.000 117

7 39 cMEM 40 33.109 0.000 74

7 39 cMEM 50 34.589 0.000 40

7 39 cMEM 60 36.395 16.913 19

7 39 cMEM 70 40.491 16.913 8

7 39 cMEM 80 41.443 38.156 4

7 39 cMEM 90 42.196 41.153 3

7 39 cMEM 100 43.396 43.396 1

7 39 dSPM 0 42.660 0.000 8002

7 39 dSPM 10 41.931 0.000 3830

7 39 dSPM 20 39.917 0.000 1778

7 39 dSPM 30 39.098 0.000 831

7 39 dSPM 40 38.762 0.000 362

7 39 dSPM 50 38.704 0.000 181

7 39 dSPM 60 40.337 0.000 90

7 39 dSPM 70 40.425 7.711 39

7 39 dSPM 80 39.964 7.711 15

7 39 dSPM 90 38.944 29.935 6

7 39 dSPM 100 47.353 47.353 1

7 39 MNE 0 43.950 0.000 8002

7 39 MNE 10 42.941 0.000 2738

7 39 MNE 20 39.354 0.000 935

7 39 MNE 30 34.806 0.000 381

7 39 MNE 40 28.974 0.000 170

7 39 MNE 50 20.568 0.000 72

7 39 MNE 60 19.366 0.000 39

7 39 MNE 70 9.782 0.000 12

7 39 MNE 80 8.117 0.000 5

7 39 MNE 90 6.125 0.000 2

7 39 MNE 100 0.000 0.000 1

7 39 sLORETA 0 43.450 0.000 8002

7 39 sLORETA 10 43.159 0.000 4925

7 39 sLORETA 20 41.577 0.000 2697

7 39 sLORETA 30 39.298 0.000 1469

7 39 sLORETA 40 37.204 0.000 828

7 39 sLORETA 50 35.426 0.000 461

7 39 sLORETA 60 33.519 0.000 229

7 39 sLORETA 70 31.285 0.000 83

7 39 sLORETA 80 25.883 0.000 32

7 39 sLORETA 90 19.239 0.000 7

7 39 sLORETA 100 10.137 10.137 1

7 40 Ave 0 44.315 0.000 8002

7 40 Ave 10 43.959 0.000 4595

7 40 Ave 20 42.207 0.000 2411

7 40 Ave 30 39.812 0.000 1221

7 40 Ave 40 38.119 0.000 635

7 40 Ave 50 35.257 0.000 288

7 40 Ave 60 31.738 0.000 116

7 40 Ave 70 27.877 7.687 32

7 40 Ave 80 25.292 7.687 10

7 40 Ave 90 20.421 18.098 3

7 40 Ave 100 18.098 18.098 1

7 40 cMEM 0 31.241 0.000 8002

7 40 cMEM 10 28.649 0.000 667

7 40 cMEM 20 26.849 0.000 278

7 40 cMEM 30 23.945 0.000 109

7 40 cMEM 40 22.440 0.000 43

7 40 cMEM 50 20.089 6.103 16

7 40 cMEM 60 18.286 6.103 8

7 40 cMEM 70 20.666 18.919 4

7 40 cMEM 80 21.119 20.368 2

7 40 cMEM 90 20.368 20.368 1

7 40 cMEM 100 20.368 20.368 1

7 40 dSPM 0 43.361 0.000 8002

7 40 dSPM 10 42.802 0.000 4210

7 40 dSPM 20 40.700 0.000 2048

7 40 dSPM 30 38.190 0.000 970

7 40 dSPM 40 36.340 0.000 453

7 40 dSPM 50 35.181 0.000 181

7 40 dSPM 60 35.442 0.000 67

7 40 dSPM 70 34.503 0.000 31

7 40 dSPM 80 39.568 30.406 8

7 40 dSPM 90 39.193 32.091 2

7 40 dSPM 100 32.091 32.091 1

7 40 MNE 0 46.507 0.000 8002

7 40 MNE 10 45.830 0.000 2776

7 40 MNE 20 43.721 0.000 1070

7 40 MNE 30 41.363 0.000 459

7 40 MNE 40 37.648 0.000 187

7 40 MNE 50 35.938 0.000 83

7 40 MNE 60 36.313 0.000 37

7 40 MNE 70 34.187 9.010 13

7 40 MNE 80 36.329 9.010 4

7 40 MNE 90 45.682 43.476 2

7 40 MNE 100 43.476 43.476 1

7 40 sLORETA 0 46.152 0.000 8002

7 40 sLORETA 10 45.975 0.000 5148

7 40 sLORETA 20 45.084 0.000 3159

7 40 sLORETA 30 43.285 0.000 1803

7 40 sLORETA 40 41.746 0.000 954

7 40 sLORETA 50 41.353 0.000 515

7 40 sLORETA 60 41.071 0.000 263

7 40 sLORETA 70 39.530 0.000 111

7 40 sLORETA 80 33.974 7.925 30

7 40 sLORETA 90 29.674 7.925 6

7 40 sLORETA 100 7.925 7.925 1

7 41 Ave 0 39.829 0.000 8002

7 41 Ave 10 39.039 0.000 4547

7 41 Ave 20 35.634 0.000 2276

7 41 Ave 30 30.174 0.000 1000

7 41 Ave 40 24.672 0.000 421

7 41 Ave 50 18.403 0.000 190

7 41 Ave 60 13.010 0.000 81

7 41 Ave 70 1.142 0.000 34

7 41 Ave 80 0.000 0.000 20

7 41 Ave 90 0.000 0.000 9

7 41 Ave 100 0.000 0.000 1

7 41 cMEM 0 17.846 0.000 8002

7 41 cMEM 10 14.967 0.000 368

7 41 cMEM 20 10.557 0.000 123

7 41 cMEM 30 4.578 0.000 59

7 41 cMEM 40 3.201 0.000 36

7 41 cMEM 50 0.000 0.000 15

7 41 cMEM 60 0.000 0.000 6

7 41 cMEM 70 0.000 0.000 3

7 41 cMEM 80 0.000 0.000 1

7 41 cMEM 90 0.000 0.000 1

7 41 cMEM 100 0.000 0.000 1

7 41 dSPM 0 38.368 0.000 8002

7 41 dSPM 10 37.310 0.000 4142

7 41 dSPM 20 33.563 0.000 1926

7 41 dSPM 30 29.015 0.000 854

7 41 dSPM 40 24.353 0.000 363

7 41 dSPM 50 18.971 0.000 147

7 41 dSPM 60 12.457 0.000 66

7 41 dSPM 70 8.630 0.000 34

7 41 dSPM 80 8.270 0.000 18

7 41 dSPM 90 4.903 0.000 6

7 41 dSPM 100 0.000 0.000 1

7 41 MNE 0 42.811 0.000 8002

7 41 MNE 10 41.693 0.000 2855

7 41 MNE 20 37.883 0.000 989

7 41 MNE 30 33.097 0.000 395

7 41 MNE 40 29.308 0.000 178

7 41 MNE 50 23.330 0.000 71

7 41 MNE 60 14.861 0.000 31

7 41 MNE 70 11.377 0.000 12

7 41 MNE 80 0.000 0.000 6

7 41 MNE 90 0.000 0.000 4

7 41 MNE 100 0.000 0.000 1

7 41 sLORETA 0 41.036 0.000 8002

7 41 sLORETA 10 40.502 0.000 4853

7 41 sLORETA 20 37.932 0.000 2568

7 41 sLORETA 30 33.410 0.000 1243

7 41 sLORETA 40 29.113 0.000 591

7 41 sLORETA 50 25.761 0.000 284

7 41 sLORETA 60 20.216 0.000 120

7 41 sLORETA 70 9.856 0.000 44

7 41 sLORETA 80 2.148 0.000 17

7 41 sLORETA 90 0.000 0.000 6

7 41 sLORETA 100 0.000 0.000 1

8 42 Ave 0 54.785 0.000 8002

8 42 Ave 10 54.171 0.000 4625

8 42 Ave 20 52.312 0.000 2549

8 42 Ave 30 50.487 0.000 1441

8 42 Ave 40 47.705 0.000 739

8 42 Ave 50 45.550 0.000 348

8 42 Ave 60 41.297 0.000 143

8 42 Ave 70 33.362 5.526 57

8 42 Ave 80 29.467 14.427 15

8 42 Ave 90 24.036 15.488 5

8 42 Ave 100 22.249 22.249 1

8 42 cMEM 0 48.422 0.000 8002

8 42 cMEM 10 48.859 0.000 421

8 42 cMEM 20 49.700 14.645 136

8 42 cMEM 30 49.288 14.645 73

8 42 cMEM 40 49.563 16.908 48

8 42 cMEM 50 50.416 24.008 34

8 42 cMEM 60 49.517 26.739 17

8 42 cMEM 70 48.858 27.899 10

8 42 cMEM 80 50.929 27.899 7

8 42 cMEM 90 51.695 27.899 5

8 42 cMEM 100 56.276 56.276 1

8 42 dSPM 0 55.751 0.000 8002

8 42 dSPM 10 55.126 0.000 4536

8 42 dSPM 20 53.656 0.000 2470

8 42 dSPM 30 51.869 0.000 1303

8 42 dSPM 40 49.277 0.000 652

8 42 dSPM 50 44.930 0.000 301

8 42 dSPM 60 39.949 8.642 137

8 42 dSPM 70 36.877 8.642 62

8 42 dSPM 80 34.660 18.046 21

8 42 dSPM 90 20.605 18.046 3

8 42 dSPM 100 18.046 18.046 1

8 42 MNE 0 54.473 0.000 8002

8 42 MNE 10 52.976 0.000 2592

8 42 MNE 20 49.891 0.000 958

8 42 MNE 30 47.358 0.000 423

8 42 MNE 40 43.941 0.000 176

8 42 MNE 50 42.721 0.000 87

8 42 MNE 60 42.948 0.000 39

8 42 MNE 70 36.811 0.000 15

8 42 MNE 80 22.090 15.285 4

8 42 MNE 90 22.249 22.249 1

8 42 MNE 100 22.249 22.249 1

8 42 sLORETA 0 55.470 0.000 8002

8 42 sLORETA 10 54.872 0.000 4500

8 42 sLORETA 20 53.479 0.000 2477

8 42 sLORETA 30 52.323 0.000 1456

8 42 sLORETA 40 51.153 0.000 783

8 42 sLORETA 50 50.659 0.000 375

8 42 sLORETA 60 48.611 0.000 145

8 42 sLORETA 70 43.368 0.000 47

8 42 sLORETA 80 27.435 0.000 18

8 42 sLORETA 90 18.755 14.427 6

8 42 sLORETA 100 21.911 21.911 1

8 43 Ave 0 44.524 0.000 8002

8 43 Ave 10 43.015 0.000 4111

8 43 Ave 20 38.024 0.000 2043

8 43 Ave 30 33.870 0.000 1083

8 43 Ave 40 30.455 0.000 575

8 43 Ave 50 27.072 0.000 262

8 43 Ave 60 24.612 0.000 117

8 43 Ave 70 24.773 0.000 49

8 43 Ave 80 22.204 1.808 11

8 43 Ave 90 3.751 1.808 2

8 43 Ave 100 4.894 4.894 1

8 43 cMEM 0 44.501 0.000 8002

8 43 cMEM 10 44.023 0.000 799

8 43 cMEM 20 43.614 0.000 364

8 43 cMEM 30 44.530 0.000 201

8 43 cMEM 40 44.493 0.000 120

8 43 cMEM 50 47.070 0.000 57

8 43 cMEM 60 47.872 0.000 22

8 43 cMEM 70 10.458 0.000 4

8 43 cMEM 80 10.458 0.000 4

8 43 cMEM 90 0.000 0.000 1

8 43 cMEM 100 0.000 0.000 1

8 43 dSPM 0 45.573 0.000 8002

8 43 dSPM 10 43.908 0.000 3618

8 43 dSPM 20 39.506 0.000 1664

8 43 dSPM 30 36.899 0.000 898

8 43 dSPM 40 34.479 0.000 504

8 43 dSPM 50 32.949 0.000 256

8 43 dSPM 60 31.541 6.296 111

8 43 dSPM 70 28.540 8.179 38

8 43 dSPM 80 21.060 11.694 15

8 43 dSPM 90 17.993 12.518 6

8 43 dSPM 100 22.084 22.084 1

8 43 MNE 0 47.329 0.000 8002

8 43 MNE 10 43.857 0.000 2209

8 43 MNE 20 38.460 0.000 727

8 43 MNE 30 33.626 0.000 302

8 43 MNE 40 29.294 0.000 113

8 43 MNE 50 28.284 0.000 40

8 43 MNE 60 24.330 0.000 15

8 43 MNE 70 27.153 1.808 6

8 43 MNE 80 23.921 1.808 2

8 43 MNE 90 23.921 1.808 2

8 43 MNE 100 1.808 1.808 1

8 43 sLORETA 0 45.592 0.000 8002

8 43 sLORETA 10 44.582 0.000 4572

8 43 sLORETA 20 41.220 0.000 2585

8 43 sLORETA 30 36.887 0.000 1464

8 43 sLORETA 40 33.796 0.000 817

8 43 sLORETA 50 31.112 0.000 448

8 43 sLORETA 60 29.451 0.000 224

8 43 sLORETA 70 29.901 0.000 95

8 43 sLORETA 80 27.777 0.000 43

8 43 sLORETA 90 31.807 20.427 5

8 43 sLORETA 100 36.484 36.484 1

8 44 Ave 0 61.341 0.000 8002

8 44 Ave 10 61.090 0.000 4787

8 44 Ave 20 59.749 0.000 2526

8 44 Ave 30 57.000 0.000 1248

8 44 Ave 40 52.612 0.000 581

8 44 Ave 50 46.816 0.000 228

8 44 Ave 60 29.407 0.000 71

8 44 Ave 70 10.897 0.000 30

8 44 Ave 80 7.712 0.000 7

8 44 Ave 90 10.439 10.439 1

8 44 Ave 100 10.439 10.439 1

8 44 cMEM 0 43.789 0.000 8002

8 44 cMEM 10 43.310 0.000 704

8 44 cMEM 20 41.617 0.000 349

8 44 cMEM 30 38.827 0.000 189

8 44 cMEM 40 35.606 0.000 120

8 44 cMEM 50 34.060 0.000 74

8 44 cMEM 60 29.831 0.000 46

8 44 cMEM 70 31.950 3.031 21

8 44 cMEM 80 34.029 6.723 12

8 44 cMEM 90 29.636 6.723 6

8 44 cMEM 100 40.194 40.194 1

8 44 dSPM 0 63.505 0.000 8002

8 44 dSPM 10 63.355 0.000 5128

8 44 dSPM 20 62.717 0.000 3015

8 44 dSPM 30 61.786 0.000 1680

8 44 dSPM 40 61.666 0.000 839

8 44 dSPM 50 63.841 0.000 430

8 44 dSPM 60 66.578 0.000 197

8 44 dSPM 70 70.145 0.000 95

8 44 dSPM 80 76.429 0.000 34

8 44 dSPM 90 81.041 7.849 14

8 44 dSPM 100 7.849 7.849 1

8 44 MNE 0 59.231 0.000 8002

8 44 MNE 10 58.416 0.000 2847

8 44 MNE 20 55.374 0.000 1132

8 44 MNE 30 50.879 0.000 479

8 44 MNE 40 44.751 0.000 210

8 44 MNE 50 38.636 0.000 97

8 44 MNE 60 35.335 0.000 46

8 44 MNE 70 20.732 0.000 20

8 44 MNE 80 12.525 0.000 11

8 44 MNE 90 7.343 0.000 6

8 44 MNE 100 0.000 0.000 1

8 44 sLORETA 0 61.571 0.000 8002

8 44 sLORETA 10 61.272 0.000 4590

8 44 sLORETA 20 59.869 0.000 2247

8 44 sLORETA 30 56.640 0.000 1087

8 44 sLORETA 40 52.049 0.000 472

8 44 sLORETA 50 47.267 0.000 181

8 44 sLORETA 60 40.028 0.000 62

8 44 sLORETA 70 24.402 0.000 10

8 44 sLORETA 80 10.439 10.439 1

8 44 sLORETA 90 10.439 10.439 1

8 44 sLORETA 100 10.439 10.439 1

8 45 Ave 0 51.555 0.000 8002

8 45 Ave 10 50.411 0.000 4103

8 45 Ave 20 46.212 0.000 1899

8 45 Ave 30 41.352 0.000 931

8 45 Ave 40 35.620 0.000 425

8 45 Ave 50 29.188 0.000 192

8 45 Ave 60 25.780 0.000 86

8 45 Ave 70 22.673 0.000 34

8 45 Ave 80 22.976 0.000 16

8 45 Ave 90 19.074 0.000 5

8 45 Ave 100 30.250 30.250 1

8 45 cMEM 0 14.678 0.000 8002

8 45 cMEM 10 11.299 0.000 253

8 45 cMEM 20 8.015 0.000 101

8 45 cMEM 30 3.454 0.000 55

8 45 cMEM 40 2.106 0.000 43

8 45 cMEM 50 1.688 0.000 25

8 45 cMEM 60 0.000 0.000 16

8 45 cMEM 70 0.000 0.000 11

8 45 cMEM 80 0.000 0.000 7

8 45 cMEM 90 0.000 0.000 2

8 45 cMEM 100 0.000 0.000 1

8 45 dSPM 0 52.395 0.000 8002

8 45 dSPM 10 51.623 0.000 4468

8 45 dSPM 20 48.128 0.000 2260

8 45 dSPM 30 43.090 0.000 1151

8 45 dSPM 40 37.227 0.000 566

8 45 dSPM 50 33.379 0.000 283

8 45 dSPM 60 32.141 0.000 138

8 45 dSPM 70 30.819 0.000 60

8 45 dSPM 80 28.810 0.000 20

8 45 dSPM 90 28.849 23.842 8

8 45 dSPM 100 23.842 23.842 1

8 45 MNE 0 55.297 0.000 8002

8 45 MNE 10 53.768 0.000 2122

8 45 MNE 20 50.529 0.000 775

8 45 MNE 30 46.850 0.000 340

8 45 MNE 40 44.072 0.000 156

8 45 MNE 50 41.401 0.000 70

8 45 MNE 60 41.948 4.556 33

8 45 MNE 70 45.149 5.050 20

8 45 MNE 80 49.637 17.738 5

8 45 MNE 90 31.873 29.086 3

8 45 MNE 100 30.250 30.250 1

8 45 sLORETA 0 52.385 0.000 8002

8 45 sLORETA 10 51.467 0.000 4193

8 45 sLORETA 20 48.426 0.000 2081

8 45 sLORETA 30 44.963 0.000 1111

8 45 sLORETA 40 40.934 0.000 536

8 45 sLORETA 50 35.700 0.000 261

8 45 sLORETA 60 29.411 0.000 114

8 45 sLORETA 70 27.126 0.000 42

8 45 sLORETA 80 27.892 8.426 15

8 45 sLORETA 90 28.108 23.842 5

8 45 sLORETA 100 28.711 28.711 1

8 46 Ave 0 50.031 0.000 8002

8 46 Ave 10 48.835 0.000 4168

8 46 Ave 20 44.629 0.000 1909

8 46 Ave 30 39.060 0.000 883

8 46 Ave 40 31.021 0.000 451

8 46 Ave 50 20.239 0.000 199

8 46 Ave 60 16.484 0.000 94

8 46 Ave 70 13.347 0.000 42

8 46 Ave 80 9.196 0.000 23

8 46 Ave 90 8.517 0.000 8

8 46 Ave 100 8.012 8.012 1

8 46 cMEM 0 33.534 0.000 8002

8 46 cMEM 10 31.468 0.000 872

8 46 cMEM 20 29.903 0.000 430

8 46 cMEM 30 29.758 0.000 258

8 46 cMEM 40 29.734 0.000 164

8 46 cMEM 50 29.245 0.000 104

8 46 cMEM 60 28.555 0.000 61

8 46 cMEM 70 30.503 0.000 35

8 46 cMEM 80 33.331 15.170 21

8 46 cMEM 90 36.762 31.761 9

8 46 cMEM 100 34.627 34.627 1

8 46 dSPM 0 50.365 0.000 8002

8 46 dSPM 10 49.537 0.000 4701

8 46 dSPM 20 45.955 0.000 2375

8 46 dSPM 30 39.525 0.000 1166

8 46 dSPM 40 29.508 0.000 531

8 46 dSPM 50 22.216 0.000 271

8 46 dSPM 60 20.474 0.000 151

8 46 dSPM 70 17.250 0.000 61

8 46 dSPM 80 9.071 0.000 20

8 46 dSPM 90 8.454 0.000 7

8 46 dSPM 100 11.774 11.774 1

8 46 MNE 0 49.503 0.000 8002

8 46 MNE 10 47.344 0.000 2131

8 46 MNE 20 42.010 0.000 788

8 46 MNE 30 37.079 0.000 356

8 46 MNE 40 30.524 0.000 163

8 46 MNE 50 24.244 0.000 75

8 46 MNE 60 15.341 0.000 38

8 46 MNE 70 11.597 0.000 17

8 46 MNE 80 7.770 0.000 10

8 46 MNE 90 7.636 0.000 5

8 46 MNE 100 10.055 10.055 1

8 46 sLORETA 0 50.627 0.000 8002

8 46 sLORETA 10 49.266 0.000 3772

8 46 sLORETA 20 45.691 0.000 1719

8 46 sLORETA 30 41.068 0.000 792

8 46 sLORETA 40 30.711 0.000 330

8 46 sLORETA 50 17.219 0.000 133

8 46 sLORETA 60 13.043 0.000 63

8 46 sLORETA 70 11.180 0.000 32

8 46 sLORETA 80 7.834 0.000 11

8 46 sLORETA 90 2.060 0.000 3

8 46 sLORETA 100 3.408 3.408 1

8 47 Ave 0 44.227 0.000 8002

8 47 Ave 10 41.841 0.000 3401

8 47 Ave 20 34.548 0.000 1345

8 47 Ave 30 27.929 0.000 625

8 47 Ave 40 24.309 0.000 328

8 47 Ave 50 22.026 0.000 151

8 47 Ave 60 20.461 0.000 68

8 47 Ave 70 19.227 0.000 22

8 47 Ave 80 17.505 0.000 6

8 47 Ave 90 10.766 10.766 1

8 47 Ave 100 10.766 10.766 1

8 47 cMEM 0 18.281 0.000 8002

8 47 cMEM 10 16.361 0.000 550

8 47 cMEM 20 14.954 0.000 246

8 47 cMEM 30 13.959 0.000 136

8 47 cMEM 40 12.940 0.000 60

8 47 cMEM 50 12.235 0.000 27

8 47 cMEM 60 11.654 0.000 11

8 47 cMEM 70 13.538 0.000 6

8 47 cMEM 80 17.429 15.285 2

8 47 cMEM 90 15.285 15.285 1

8 47 cMEM 100 15.285 15.285 1

8 47 dSPM 0 45.669 0.000 8002

8 47 dSPM 10 44.554 0.000 4595

8 47 dSPM 20 39.694 0.000 2316

8 47 dSPM 30 33.055 0.000 1163

8 47 dSPM 40 28.132 0.000 646

8 47 dSPM 50 25.229 0.000 357

8 47 dSPM 60 23.760 0.000 205

8 47 dSPM 70 23.062 0.000 94

8 47 dSPM 80 23.013 0.000 39

8 47 dSPM 90 19.494 0.000 8

8 47 dSPM 100 25.235 25.235 1

8 47 MNE 0 45.154 0.000 8002

8 47 MNE 10 42.618 0.000 2101

8 47 MNE 20 36.099 0.000 796

8 47 MNE 30 31.165 0.000 372

8 47 MNE 40 27.282 0.000 191

8 47 MNE 50 26.034 0.000 112

8 47 MNE 60 24.782 0.000 53

8 47 MNE 70 24.524 3.764 26

8 47 MNE 80 24.802 5.842 14

8 47 MNE 90 22.895 8.012 6

8 47 MNE 100 30.250 30.250 1

8 47 sLORETA 0 45.440 0.000 8002

8 47 sLORETA 10 43.925 0.000 3761

8 47 sLORETA 20 39.251 0.000 1765

8 47 sLORETA 30 33.518 0.000 871

8 47 sLORETA 40 28.839 0.000 448

8 47 sLORETA 50 25.790 0.000 256

8 47 sLORETA 60 23.071 0.000 129

8 47 sLORETA 70 21.779 0.000 53

8 47 sLORETA 80 22.345 3.408 20

8 47 sLORETA 90 20.715 8.426 5

8 47 sLORETA 100 28.711 28.711 1

9 48 Ave 0 22.604 0.000 8002

9 48 Ave 10 21.053 0.000 3740

9 48 Ave 20 16.904 0.000 1714

9 48 Ave 30 11.945 0.000 829

9 48 Ave 40 7.262 0.000 433

9 48 Ave 50 4.971 0.000 247

9 48 Ave 60 4.258 0.000 145

9 48 Ave 70 2.830 0.000 66

9 48 Ave 80 2.667 0.000 27

9 48 Ave 90 0.000 0.000 9

9 48 Ave 100 0.000 0.000 1

9 48 cMEM 0 26.531 0.000 8002

9 48 cMEM 10 26.378 0.000 764

9 48 cMEM 20 26.196 0.000 306

9 48 cMEM 30 25.857 0.000 153

9 48 cMEM 40 26.142 0.000 99

9 48 cMEM 50 26.913 0.000 52

9 48 cMEM 60 29.177 0.000 24

9 48 cMEM 70 31.373 0.000 15

9 48 cMEM 80 36.935 33.224 5

9 48 cMEM 90 35.495 35.495 1

9 48 cMEM 100 35.495 35.495 1

9 48 dSPM 0 25.440 0.000 8002

9 48 dSPM 10 24.465 0.000 3709

9 48 dSPM 20 22.373 0.000 1936

9 48 dSPM 30 19.304 0.000 1016

9 48 dSPM 40 16.958 0.000 519

9 48 dSPM 50 14.887 0.000 274

9 48 dSPM 60 11.259 0.000 136

9 48 dSPM 70 10.319 0.000 72

9 48 dSPM 80 9.324 0.000 27

9 48 dSPM 90 9.601 0.000 11

9 48 dSPM 100 0.000 0.000 1

9 48 MNE 0 23.642 0.000 8002

9 48 MNE 10 21.464 0.000 2692

9 48 MNE 20 18.016 0.000 1167

9 48 MNE 30 14.646 0.000 581

9 48 MNE 40 12.983 0.000 308

9 48 MNE 50 11.913 0.000 162

9 48 MNE 60 11.359 0.000 79

9 48 MNE 70 0.000 0.000 33

9 48 MNE 80 0.000 0.000 11

9 48 MNE 90 0.000 0.000 4

9 48 MNE 100 0.000 0.000 1

9 48 sLORETA 0 22.498 0.000 8002

9 48 sLORETA 10 20.584 0.000 3418

9 48 sLORETA 20 16.203 0.000 1445

9 48 sLORETA 30 11.225 0.000 660

9 48 sLORETA 40 7.082 0.000 375

9 48 sLORETA 50 5.295 0.000 214

9 48 sLORETA 60 4.851 0.000 122

9 48 sLORETA 70 3.703 0.000 45

9 48 sLORETA 80 2.937 0.000 11

9 48 sLORETA 90 0.000 0.000 1

9 48 sLORETA 100 0.000 0.000 1

9 49 Ave 0 23.601 0.000 8002

9 49 Ave 10 21.583 0.000 3467

9 49 Ave 20 14.898 0.000 1416

9 49 Ave 30 9.380 0.000 718

9 49 Ave 40 5.721 0.000 365

9 49 Ave 50 5.284 0.000 158

9 49 Ave 60 4.973 0.000 72

9 49 Ave 70 4.948 0.000 31

9 49 Ave 80 5.278 0.000 10

9 49 Ave 90 6.997 0.000 2

9 49 Ave 100 0.000 0.000 1

9 49 cMEM 0 14.114 0.000 8002

9 49 cMEM 10 13.440 0.000 305

9 49 cMEM 20 13.319 0.000 135

9 49 cMEM 30 12.580 0.000 73

9 49 cMEM 40 11.512 0.000 44

9 49 cMEM 50 10.478 0.000 28

9 49 cMEM 60 10.605 0.000 17

9 49 cMEM 70 10.843 5.130 9

9 49 cMEM 80 11.010 6.396 5

9 49 cMEM 90 9.286 8.228 2

9 49 cMEM 100 10.169 10.169 1

9 49 dSPM 0 23.613 0.000 8002

9 49 dSPM 10 21.215 0.000 3113

9 49 dSPM 20 14.748 0.000 1251

9 49 dSPM 30 8.250 0.000 528

9 49 dSPM 40 7.066 0.000 232

9 49 dSPM 50 6.379 0.000 103

9 49 dSPM 60 4.982 0.000 49

9 49 dSPM 70 4.034 0.000 21

9 49 dSPM 80 4.169 0.000 6

9 49 dSPM 90 0.000 0.000 2

9 49 dSPM 100 0.000 0.000 1

9 49 MNE 0 26.934 0.000 8002

9 49 MNE 10 24.896 0.000 2468

9 49 MNE 20 20.213 0.000 984

9 49 MNE 30 15.919 0.000 468

9 49 MNE 40 11.518 0.000 207

9 49 MNE 50 8.432 0.000 93

9 49 MNE 60 3.887 0.000 44

9 49 MNE 70 3.879 0.000 20

9 49 MNE 80 3.416 0.000 11

9 49 MNE 90 0.000 0.000 3

9 49 MNE 100 0.000 0.000 1

9 49 sLORETA 0 24.015 0.000 8002

9 49 sLORETA 10 22.615 0.000 4002

9 49 sLORETA 20 17.776 0.000 1863

9 49 sLORETA 30 13.000 0.000 1033

9 49 sLORETA 40 7.862 0.000 566

9 49 sLORETA 50 5.890 0.000 297

9 49 sLORETA 60 4.150 0.000 117

9 49 sLORETA 70 3.090 0.000 44

9 49 sLORETA 80 0.000 0.000 16

9 49 sLORETA 90 0.000 0.000 4

9 49 sLORETA 100 0.000 0.000 1

9 50 Ave 0 22.124 0.000 8002

9 50 Ave 10 20.381 0.000 3625

9 50 Ave 20 16.285 0.000 1694

9 50 Ave 30 11.270 0.000 852

9 50 Ave 40 8.290 0.000 485

9 50 Ave 50 6.940 0.000 285

9 50 Ave 60 4.521 0.000 159

9 50 Ave 70 2.416 0.000 77

9 50 Ave 80 0.000 0.000 29

9 50 Ave 90 0.000 0.000 7

9 50 Ave 100 0.000 0.000 1

9 50 cMEM 0 18.348 0.000 8002

9 50 cMEM 10 18.002 0.000 552

9 50 cMEM 20 18.575 0.000 268

9 50 cMEM 30 19.787 0.000 130

9 50 cMEM 40 20.459 0.000 76

9 50 cMEM 50 20.513 0.000 54

9 50 cMEM 60 20.630 0.000 32

9 50 cMEM 70 19.157 0.000 18

9 50 cMEM 80 17.903 0.000 9

9 50 cMEM 90 23.316 20.746 2

9 50 cMEM 100 20.746 20.746 1

9 50 dSPM 0 23.075 0.000 8002

9 50 dSPM 10 21.528 0.000 3465

9 50 dSPM 20 18.245 0.000 1667

9 50 dSPM 30 13.991 0.000 846

9 50 dSPM 40 10.006 0.000 444

9 50 dSPM 50 7.780 0.000 234

9 50 dSPM 60 5.366 0.000 125

9 50 dSPM 70 5.458 0.000 63

9 50 dSPM 80 2.942 0.000 18

9 50 dSPM 90 0.000 0.000 2

9 50 dSPM 100 0.000 0.000 1

9 50 MNE 0 24.776 0.000 8002

9 50 MNE 10 21.899 0.000 2243

9 50 MNE 20 17.851 0.000 903

9 50 MNE 30 13.024 0.000 374

9 50 MNE 40 6.363 0.000 161

9 50 MNE 50 3.444 0.000 68

9 50 MNE 60 1.617 0.000 26

9 50 MNE 70 2.120 0.000 11

9 50 MNE 80 0.000 0.000 4

9 50 MNE 90 0.000 0.000 2

9 50 MNE 100 0.000 0.000 1

9 50 sLORETA 0 22.268 0.000 8002

9 50 sLORETA 10 20.323 0.000 3439

9 50 sLORETA 20 15.999 0.000 1518

9 50 sLORETA 30 11.141 0.000 735

9 50 sLORETA 40 6.082 0.000 408

9 50 sLORETA 50 3.500 0.000 237

9 50 sLORETA 60 2.402 0.000 138

9 50 sLORETA 70 0.767 0.000 59

9 50 sLORETA 80 0.000 0.000 26

9 50 sLORETA 90 0.000 0.000 3

9 50 sLORETA 100 0.000 0.000 1

9 51 Ave 0 21.815 0.000 8002

9 51 Ave 10 20.351 0.000 3968

9 51 Ave 20 15.420 0.000 1855

9 51 Ave 30 9.160 0.000 894

9 51 Ave 40 6.668 0.000 530

9 51 Ave 50 5.622 0.000 324

9 51 Ave 60 4.639 0.000 215

9 51 Ave 70 4.241 0.000 107

9 51 Ave 80 3.357 0.000 57

9 51 Ave 90 0.000 0.000 13

9 51 Ave 100 0.000 0.000 1

9 51 cMEM 0 18.570 0.000 8002

9 51 cMEM 10 17.515 0.000 563

9 51 cMEM 20 17.255 0.000 248

9 51 cMEM 30 16.480 0.000 109

9 51 cMEM 40 16.944 0.000 71

9 51 cMEM 50 18.319 0.000 39

9 51 cMEM 60 18.019 0.000 27

9 51 cMEM 70 18.609 8.228 15

9 51 cMEM 80 19.868 11.847 7

9 51 cMEM 90 21.565 17.157 3

9 51 cMEM 100 20.746 20.746 1

9 51 dSPM 0 23.821 0.000 8002

9 51 dSPM 10 22.584 0.000 3674

9 51 dSPM 20 19.918 0.000 1909

9 51 dSPM 30 15.597 0.000 951

9 51 dSPM 40 10.354 0.000 503

9 51 dSPM 50 7.437 0.000 288

9 51 dSPM 60 7.485 0.000 157

9 51 dSPM 70 7.033 0.000 83

9 51 dSPM 80 9.572 0.000 27

9 51 dSPM 90 10.972 0.000 10

9 51 dSPM 100 0.000 0.000 1

9 51 MNE 0 23.393 0.000 8002

9 51 MNE 10 20.420 0.000 2397

9 51 MNE 20 14.186 0.000 917

9 51 MNE 30 9.465 0.000 437

9 51 MNE 40 5.803 0.000 209

9 51 MNE 50 3.188 0.000 94

9 51 MNE 60 2.807 0.000 42

9 51 MNE 70 1.431 0.000 15

9 51 MNE 80 0.000 0.000 7

9 51 MNE 90 0.000 0.000 2

9 51 MNE 100 0.000 0.000 1

9 51 sLORETA 0 22.206 0.000 8002

9 51 sLORETA 10 20.277 0.000 3541

9 51 sLORETA 20 14.553 0.000 1420

9 51 sLORETA 30 9.704 0.000 673

9 51 sLORETA 40 6.820 0.000 410

9 51 sLORETA 50 5.190 0.000 252

9 51 sLORETA 60 3.997 0.000 144

9 51 sLORETA 70 3.274 0.000 54

9 51 sLORETA 80 0.000 0.000 9

9 51 sLORETA 90 0.000 0.000 2

9 51 sLORETA 100 0.000 0.000 1

9 52 Ave 0 25.708 0.000 8002

9 52 Ave 10 23.893 0.000 3484

9 52 Ave 20 18.107 0.000 1337

9 52 Ave 30 10.236 0.000 541

9 52 Ave 40 5.260 0.000 281

9 52 Ave 50 3.164 0.000 147

9 52 Ave 60 1.654 0.000 83

9 52 Ave 70 0.000 0.000 28

9 52 Ave 80 0.000 0.000 10

9 52 Ave 90 0.000 0.000 4

9 52 Ave 100 0.000 0.000 1

9 52 cMEM 0 23.859 0.000 8002

9 52 cMEM 10 21.582 0.000 485

9 52 cMEM 20 18.973 0.000 156

9 52 cMEM 30 15.680 0.000 64

9 52 cMEM 40 9.231 0.000 25

9 52 cMEM 50 0.000 0.000 20

9 52 cMEM 60 0.000 0.000 14

9 52 cMEM 70 0.000 0.000 9

9 52 cMEM 80 0.000 0.000 5

9 52 cMEM 90 0.000 0.000 2

9 52 cMEM 100 0.000 0.000 1

9 52 dSPM 0 27.630 0.000 8002

9 52 dSPM 10 26.720 0.000 3765

9 52 dSPM 20 24.576 0.000 1941

9 52 dSPM 30 22.167 0.000 1037

9 52 dSPM 40 18.918 0.000 522

9 52 dSPM 50 15.761 0.000 286

9 52 dSPM 60 9.877 0.000 136

9 52 dSPM 70 7.207 0.000 73

9 52 dSPM 80 5.992 0.000 36

9 52 dSPM 90 1.226 0.000 13

9 52 dSPM 100 0.000 0.000 1

9 52 MNE 0 27.413 0.000 8002

9 52 MNE 10 25.018 0.000 2501

9 52 MNE 20 19.672 0.000 911

9 52 MNE 30 15.291 0.000 393

9 52 MNE 40 11.570 0.000 187

9 52 MNE 50 10.707 0.000 89

9 52 MNE 60 8.934 0.000 35

9 52 MNE 70 1.229 0.000 12

9 52 MNE 80 0.000 0.000 5

9 52 MNE 90 0.000 0.000 3

9 52 MNE 100 0.000 0.000 1

9 52 sLORETA 0 25.435 0.000 8002

9 52 sLORETA 10 24.182 0.000 3924

9 52 sLORETA 20 20.106 0.000 1730

9 52 sLORETA 30 15.564 0.000 818

9 52 sLORETA 40 11.306 0.000 444

9 52 sLORETA 50 7.708 0.000 277

9 52 sLORETA 60 5.504 0.000 167

9 52 sLORETA 70 3.008 0.000 96

9 52 sLORETA 80 1.663 0.000 42

9 52 sLORETA 90 0.000 0.000 15

9 52 sLORETA 100 0.000 0.000 1

9 53 Ave 0 24.926 0.000 8002

9 53 Ave 10 23.476 0.000 4013

9 53 Ave 20 17.517 0.000 1734

9 53 Ave 30 10.081 0.000 762

9 53 Ave 40 5.554 0.000 405

9 53 Ave 50 4.340 0.000 220

9 53 Ave 60 3.768 0.000 120

9 53 Ave 70 1.772 0.000 39

9 53 Ave 80 0.000 0.000 16

9 53 Ave 90 0.000 0.000 4

9 53 Ave 100 0.000 0.000 1

9 53 cMEM 0 23.240 0.000 8002

9 53 cMEM 10 21.990 0.000 762

9 53 cMEM 20 20.780 0.000 249

9 53 cMEM 30 19.869 0.000 133

9 53 cMEM 40 18.821 0.000 93

9 53 cMEM 50 17.904 0.000 67

9 53 cMEM 60 16.596 0.000 38

9 53 cMEM 70 17.160 0.000 23

9 53 cMEM 80 18.521 8.228 10

9 53 cMEM 90 17.463 8.228 3

9 53 cMEM 100 10.169 10.169 1

9 53 dSPM 0 26.096 0.000 8002

9 53 dSPM 10 24.790 0.000 3743

9 53 dSPM 20 21.225 0.000 1718

9 53 dSPM 30 17.280 0.000 848

9 53 dSPM 40 14.195 0.000 433

9 53 dSPM 50 10.437 0.000 211

9 53 dSPM 60 8.030 0.000 97

9 53 dSPM 70 7.348 0.000 44

9 53 dSPM 80 9.027 0.000 14

9 53 dSPM 90 0.000 0.000 1

9 53 dSPM 100 0.000 0.000 1

9 53 MNE 0 28.734 0.000 8002

9 53 MNE 10 27.139 0.000 2945

9 53 MNE 20 23.538 0.000 1225

9 53 MNE 30 18.135 0.000 568

9 53 MNE 40 12.203 0.000 256

9 53 MNE 50 5.031 0.000 119

9 53 MNE 60 2.901 0.000 51

9 53 MNE 70 2.021 0.000 22

9 53 MNE 80 2.523 0.000 9

9 53 MNE 90 0.000 0.000 4

9 53 MNE 100 0.000 0.000 1

9 53 sLORETA 0 24.240 0.000 8002

9 53 sLORETA 10 22.824 0.000 4101

9 53 sLORETA 20 16.402 0.000 1691

9 53 sLORETA 30 11.077 0.000 788

9 53 sLORETA 40 5.963 0.000 417

9 53 sLORETA 50 4.808 0.000 269

9 53 sLORETA 60 3.958 0.000 151

9 53 sLORETA 70 2.618 0.000 72

9 53 sLORETA 80 0.000 0.000 21

9 53 sLORETA 90 0.000 0.000 3

9 53 sLORETA 100 0.000 0.000 1

9 54 Ave 0 28.556 0.000 8002

9 54 Ave 10 28.074 0.000 5069

9 54 Ave 20 25.803 0.000 2920

9 54 Ave 30 22.494 0.000 1676

9 54 Ave 40 18.700 0.000 986

9 54 Ave 50 14.704 0.000 547

9 54 Ave 60 10.966 0.000 290

9 54 Ave 70 8.539 0.000 124

9 54 Ave 80 8.968 0.000 46

9 54 Ave 90 7.437 0.000 14

9 54 Ave 100 4.929 4.929 1

9 54 cMEM 0 18.554 0.000 8002

9 54 cMEM 10 17.809 0.000 282

9 54 cMEM 20 18.330 0.000 141

9 54 cMEM 30 19.064 0.000 87

9 54 cMEM 40 19.692 0.000 60

9 54 cMEM 50 19.710 4.801 44

9 54 cMEM 60 20.540 8.228 28

9 54 cMEM 70 20.569 8.228 19

9 54 cMEM 80 21.990 14.781 9

9 54 cMEM 90 23.084 17.157 4

9 54 cMEM 100 20.746 20.746 1

9 54 dSPM 0 28.185 0.000 8002

9 54 dSPM 10 27.248 0.000 4296

9 54 dSPM 20 24.011 0.000 2164

9 54 dSPM 30 19.531 0.000 1053

9 54 dSPM 40 14.890 0.000 481

9 54 dSPM 50 10.624 0.000 214

9 54 dSPM 60 7.262 0.000 93

9 54 dSPM 70 7.992 0.000 33

9 54 dSPM 80 8.803 0.000 8

9 54 dSPM 90 7.768 0.000 2

9 54 dSPM 100 10.754 10.754 1

9 54 MNE 0 32.683 0.000 8002

9 54 MNE 10 31.855 0.000 3258

9 54 MNE 20 29.457 0.000 1433

9 54 MNE 30 27.693 0.000 663

9 54 MNE 40 25.483 0.000 289

9 54 MNE 50 25.503 0.000 127

9 54 MNE 60 22.681 0.000 54

9 54 MNE 70 23.936 0.000 22

9 54 MNE 80 20.518 0.000 7

9 54 MNE 90 6.573 6.573 1

9 54 MNE 100 6.573 6.573 1

9 54 sLORETA 0 28.207 0.000 8002

9 54 sLORETA 10 27.490 0.000 4671

9 54 sLORETA 20 24.754 0.000 2494

9 54 sLORETA 30 20.752 0.000 1359

9 54 sLORETA 40 16.874 0.000 717

9 54 sLORETA 50 12.207 0.000 352

9 54 sLORETA 60 8.291 0.000 156

9 54 sLORETA 70 6.604 0.000 59

9 54 sLORETA 80 5.458 0.000 12

9 54 sLORETA 90 0.000 0.000 3

9 54 sLORETA 100 0.000 0.000 1

9 55 Ave 0 24.919 0.000 8002

9 55 Ave 10 23.399 0.000 3826

9 55 Ave 20 19.695 0.000 1781

9 55 Ave 30 15.341 0.000 806

9 55 Ave 40 11.425 0.000 424

9 55 Ave 50 6.497 0.000 213

9 55 Ave 60 3.858 0.000 96

9 55 Ave 70 3.782 0.000 45

9 55 Ave 80 4.722 0.000 11

9 55 Ave 90 6.659 0.000 3

9 55 Ave 100 7.777 7.777 1

9 55 cMEM 0 13.856 0.000 8002

9 55 cMEM 10 9.014 0.000 88

9 55 cMEM 20 7.526 0.000 38

9 55 cMEM 30 7.201 0.000 14

9 55 cMEM 40 7.571 6.141 4

9 55 cMEM 50 7.571 6.141 4

9 55 cMEM 60 7.218 6.141 3

9 55 cMEM 70 6.904 6.141 2

9 55 cMEM 80 6.904 6.141 2

9 55 cMEM 90 6.141 6.141 1

9 55 cMEM 100 6.141 6.141 1

9 55 dSPM 0 25.519 0.000 8002

9 55 dSPM 10 24.385 0.000 3835

9 55 dSPM 20 22.041 0.000 1996

9 55 dSPM 30 19.513 0.000 1049

9 55 dSPM 40 15.784 0.000 535

9 55 dSPM 50 11.382 0.000 263

9 55 dSPM 60 5.186 0.000 118

9 55 dSPM 70 4.405 0.000 50

9 55 dSPM 80 4.274 0.000 21

9 55 dSPM 90 5.960 0.000 3

9 55 dSPM 100 0.000 0.000 1

9 55 MNE 0 26.117 0.000 8002

9 55 MNE 10 23.149 0.000 2077

9 55 MNE 20 19.569 0.000 767

9 55 MNE 30 18.104 0.000 328

9 55 MNE 40 18.423 0.000 142

9 55 MNE 50 15.988 0.000 68

9 55 MNE 60 10.666 0.000 29

9 55 MNE 70 4.961 0.000 8

9 55 MNE 80 5.980 0.000 3

9 55 MNE 90 7.777 7.777 1

9 55 MNE 100 7.777 7.777 1

9 55 sLORETA 0 24.707 0.000 8002

9 55 sLORETA 10 23.203 0.000 3830

9 55 sLORETA 20 19.586 0.000 1739

9 55 sLORETA 30 16.067 0.000 818

9 55 sLORETA 40 11.831 0.000 418

9 55 sLORETA 50 6.426 0.000 227

9 55 sLORETA 60 4.031 0.000 123

9 55 sLORETA 70 2.329 0.000 64

9 55 sLORETA 80 2.301 0.000 35

9 55 sLORETA 90 1.950 0.000 12

9 55 sLORETA 100 0.000 0.000 1

10 56 Ave 0 38.065 0.000 8002

10 56 Ave 10 36.735 0.000 3542

10 56 Ave 20 33.550 0.000 1691

10 56 Ave 30 29.825 0.000 818

10 56 Ave 40 26.207 0.000 391

10 56 Ave 50 23.328 0.000 179

10 56 Ave 60 19.819 0.000 97

10 56 Ave 70 16.902 0.000 48

10 56 Ave 80 15.866 0.000 26

10 56 Ave 90 15.279 0.000 14

10 56 Ave 100 13.289 13.289 1

10 56 cMEM 0 17.458 0.000 8002

10 56 cMEM 10 14.849 0.000 625

10 56 cMEM 20 11.163 0.000 289

10 56 cMEM 30 9.758 0.000 133

10 56 cMEM 40 7.427 0.000 69

10 56 cMEM 50 2.989 0.000 38

10 56 cMEM 60 1.404 0.000 23

10 56 cMEM 70 1.226 0.000 11

10 56 cMEM 80 0.000 0.000 5

10 56 cMEM 90 0.000 0.000 5

10 56 cMEM 100 0.000 0.000 1

10 56 dSPM 0 40.810 0.000 8002

10 56 dSPM 10 40.138 0.000 3983

10 56 dSPM 20 38.570 0.000 2244

10 56 dSPM 30 36.900 0.000 1296

10 56 dSPM 40 32.756 0.000 653

10 56 dSPM 50 26.140 0.000 330

10 56 dSPM 60 19.609 0.000 152

10 56 dSPM 70 18.041 0.000 85

10 56 dSPM 80 15.999 0.000 36

10 56 dSPM 90 14.972 0.000 16

10 56 dSPM 100 13.289 13.289 1

10 56 MNE 0 39.373 0.000 8002

10 56 MNE 10 37.244 0.000 2274

10 56 MNE 20 34.923 0.000 962

10 56 MNE 30 34.993 0.000 440

10 56 MNE 40 35.673 0.000 212

10 56 MNE 50 38.587 0.000 107

10 56 MNE 60 37.859 0.000 57

10 56 MNE 70 37.987 0.000 26

10 56 MNE 80 37.624 0.000 16

10 56 MNE 90 47.516 0.000 5

10 56 MNE 100 66.465 66.465 1

10 56 sLORETA 0 37.199 0.000 8002

10 56 sLORETA 10 35.393 0.000 2874

10 56 sLORETA 20 31.727 0.000 1202

10 56 sLORETA 30 29.609 0.000 546

10 56 sLORETA 40 25.967 0.000 241

10 56 sLORETA 50 20.361 0.000 127

10 56 sLORETA 60 19.206 0.000 79

10 56 sLORETA 70 18.352 0.000 41

10 56 sLORETA 80 17.757 5.179 22

10 56 sLORETA 90 14.791 12.540 6

10 56 sLORETA 100 12.540 12.540 1

10 57 Ave 0 32.397 0.000 8002

10 57 Ave 10 29.657 0.000 3129

10 57 Ave 20 23.974 0.000 1320

10 57 Ave 30 18.322 0.000 620

10 57 Ave 40 13.243 0.000 319

10 57 Ave 50 9.663 0.000 187

10 57 Ave 60 6.665 0.000 94

10 57 Ave 70 3.922 0.000 40

10 57 Ave 80 2.355 0.000 12

10 57 Ave 90 0.000 0.000 5

10 57 Ave 100 0.000 0.000 1

10 57 cMEM 0 18.838 0.000 8002

10 57 cMEM 10 17.938 0.000 453

10 57 cMEM 20 16.743 0.000 256

10 57 cMEM 30 14.549 0.000 142

10 57 cMEM 40 12.769 0.000 84

10 57 cMEM 50 9.226 0.000 50

10 57 cMEM 60 10.364 0.000 24

10 57 cMEM 70 12.531 4.394 10

10 57 cMEM 80 13.063 11.268 5

10 57 cMEM 90 13.432 12.052 3

10 57 cMEM 100 12.052 12.052 1

10 57 dSPM 0 36.564 0.000 8002

10 57 dSPM 10 35.278 0.000 3761

10 57 dSPM 20 32.420 0.000 1937

10 57 dSPM 30 28.789 0.000 1069

10 57 dSPM 40 24.957 0.000 538

10 57 dSPM 50 19.676 0.000 287

10 57 dSPM 60 15.943 0.000 144

10 57 dSPM 70 12.503 0.000 72

10 57 dSPM 80 9.842 0.000 29

10 57 dSPM 90 6.299 0.000 3

10 57 dSPM 100 0.000 0.000 1

10 57 MNE 0 31.858 0.000 8002

10 57 MNE 10 25.946 0.000 1228

10 57 MNE 20 21.846 0.000 415

10 57 MNE 30 17.695 0.000 165

10 57 MNE 40 16.459 0.000 66

10 57 MNE 50 8.603 0.000 33

10 57 MNE 60 0.000 0.000 15

10 57 MNE 70 0.000 0.000 5

10 57 MNE 80 0.000 0.000 1

10 57 MNE 90 0.000 0.000 1

10 57 MNE 100 0.000 0.000 1

10 57 sLORETA 0 33.039 0.000 8002

10 57 sLORETA 10 31.131 0.000 3585

10 57 sLORETA 20 26.546 0.000 1581

10 57 sLORETA 30 23.241 0.000 850

10 57 sLORETA 40 19.425 0.000 486

10 57 sLORETA 50 14.603 0.000 270

10 57 sLORETA 60 11.811 0.000 155

10 57 sLORETA 70 9.976 0.000 90

10 57 sLORETA 80 7.996 0.000 35

10 57 sLORETA 90 5.558 0.000 8

10 57 sLORETA 100 4.049 4.049 1

10 58 Ave 0 41.438 0.000 8002

10 58 Ave 10 40.359 0.000 4212

10 58 Ave 20 36.240 0.000 1893

10 58 Ave 30 31.102 0.000 846

10 58 Ave 40 27.330 0.000 379

10 58 Ave 50 25.631 0.000 164

10 58 Ave 60 22.952 0.000 70

10 58 Ave 70 22.991 7.149 38

10 58 Ave 80 21.624 10.462 12

10 58 Ave 90 19.706 13.418 6

10 58 Ave 100 13.418 13.418 1

10 58 cMEM 0 15.262 0.000 8002

10 58 cMEM 10 13.099 0.000 617

10 58 cMEM 20 9.914 0.000 275

10 58 cMEM 30 6.176 0.000 148

10 58 cMEM 40 4.600 0.000 90

10 58 cMEM 50 3.546 0.000 50

10 58 cMEM 60 0.000 0.000 26

10 58 cMEM 70 0.000 0.000 14

10 58 cMEM 80 0.000 0.000 9

10 58 cMEM 90 0.000 0.000 3

10 58 cMEM 100 0.000 0.000 1

10 58 dSPM 0 43.993 0.000 8002

10 58 dSPM 10 43.570 0.000 5069

10 58 dSPM 20 41.885 0.000 2916

10 58 dSPM 30 39.315 0.000 1655

10 58 dSPM 40 36.666 0.000 912

10 58 dSPM 50 32.950 0.000 471

10 58 dSPM 60 29.577 0.000 211

10 58 dSPM 70 24.778 0.000 75

10 58 dSPM 80 17.476 7.149 28

10 58 dSPM 90 16.841 10.462 11

10 58 dSPM 100 10.462 10.462 1

10 58 MNE 0 40.783 0.000 8002

10 58 MNE 10 36.952 0.000 1500

10 58 MNE 20 30.724 0.000 370

10 58 MNE 30 27.492 0.000 119

10 58 MNE 40 25.556 0.000 60

10 58 MNE 50 26.754 0.000 34

10 58 MNE 60 26.590 0.000 18

10 58 MNE 70 25.499 0.000 9

10 58 MNE 80 30.086 16.755 5

10 58 MNE 90 24.282 24.282 1

10 58 MNE 100 24.282 24.282 1

10 58 sLORETA 0 39.329 0.000 8002

10 58 sLORETA 10 38.083 0.000 3905

10 58 sLORETA 20 34.011 0.000 1694

10 58 sLORETA 30 30.586 0.000 861

10 58 sLORETA 40 28.817 0.000 447

10 58 sLORETA 50 27.315 0.000 226

10 58 sLORETA 60 25.617 0.000 107

10 58 sLORETA 70 22.649 7.149 49

10 58 sLORETA 80 21.702 7.149 22

10 58 sLORETA 90 19.460 13.418 7

10 58 sLORETA 100 21.485 21.485 1

10 59 Ave 0 38.868 0.000 8002

10 59 Ave 10 37.424 0.000 3431

10 59 Ave 20 33.537 0.000 1426

10 59 Ave 30 30.602 0.000 692

10 59 Ave 40 27.115 0.000 349

10 59 Ave 50 22.020 0.000 168

10 59 Ave 60 22.523 0.000 73

10 59 Ave 70 19.820 0.000 34

10 59 Ave 80 19.871 13.418 7

10 59 Ave 90 21.888 16.221 4

10 59 Ave 100 16.221 16.221 1

10 59 cMEM 0 16.796 0.000 8002

10 59 cMEM 10 13.456 0.000 513

10 59 cMEM 20 10.481 0.000 229

10 59 cMEM 30 8.062 0.000 127

10 59 cMEM 40 5.589 0.000 74

10 59 cMEM 50 5.199 0.000 46

10 59 cMEM 60 4.939 0.000 28

10 59 cMEM 70 3.486 0.000 17

10 59 cMEM 80 1.624 0.000 7

10 59 cMEM 90 0.000 0.000 4

10 59 cMEM 100 0.000 0.000 1

10 59 dSPM 0 40.948 0.000 8002

10 59 dSPM 10 40.194 0.000 4094

10 59 dSPM 20 38.137 0.000 2211

10 59 dSPM 30 35.581 0.000 1173

10 59 dSPM 40 31.777 0.000 620

10 59 dSPM 50 25.379 0.000 301

10 59 dSPM 60 20.209 0.000 135

10 59 dSPM 70 19.583 0.000 59

10 59 dSPM 80 20.466 10.462 20

10 59 dSPM 90 15.907 12.385 4

10 59 dSPM 100 16.221 16.221 1

10 59 MNE 0 38.002 0.000 8002

10 59 MNE 10 34.747 0.000 1190

10 59 MNE 20 32.712 0.000 394

10 59 MNE 30 32.150 0.000 169

10 59 MNE 40 30.286 0.000 80

10 59 MNE 50 28.983 0.000 32

10 59 MNE 60 25.850 13.418 16

10 59 MNE 70 24.247 13.418 9

10 59 MNE 80 24.676 13.418 7

10 59 MNE 90 29.338 16.221 3

10 59 MNE 100 25.669 25.669 1

10 59 sLORETA 0 37.859 0.000 8002

10 59 sLORETA 10 36.121 0.000 3039

10 59 sLORETA 20 32.957 0.000 1224

10 59 sLORETA 30 31.243 0.000 642

10 59 sLORETA 40 28.520 0.000 347

10 59 sLORETA 50 23.520 0.000 177

10 59 sLORETA 60 22.407 0.000 91

10 59 sLORETA 70 19.965 5.835 44

10 59 sLORETA 80 19.806 12.608 17

10 59 sLORETA 90 17.438 16.208 3

10 59 sLORETA 100 16.221 16.221 1

10 60 Ave 0 34.537 0.000 8002

10 60 Ave 10 33.186 0.000 3469

10 60 Ave 20 30.582 0.000 1906

10 60 Ave 30 27.968 0.000 1153

10 60 Ave 40 26.932 0.000 677

10 60 Ave 50 26.769 0.000 347

10 60 Ave 60 25.653 0.000 155

10 60 Ave 70 25.442 0.000 78

10 60 Ave 80 26.982 10.056 27

10 60 Ave 90 29.302 13.802 7

10 60 Ave 100 38.776 38.776 1

10 60 cMEM 0 20.498 0.000 8002

10 60 cMEM 10 18.818 0.000 597

10 60 cMEM 20 18.171 0.000 306

10 60 cMEM 30 18.099 0.000 193

10 60 cMEM 40 18.548 0.000 112

10 60 cMEM 50 18.787 0.000 74

10 60 cMEM 60 19.572 0.000 46

10 60 cMEM 70 19.702 4.001 26

10 60 cMEM 80 21.547 12.048 10

10 60 cMEM 90 24.654 12.048 5

10 60 cMEM 100 18.938 18.938 1

10 60 dSPM 0 36.293 0.000 8002

10 60 dSPM 10 35.335 0.000 3891

10 60 dSPM 20 33.146 0.000 2176

10 60 dSPM 30 30.941 0.000 1354

10 60 dSPM 40 28.275 0.000 814

10 60 dSPM 50 26.461 0.000 477

10 60 dSPM 60 25.787 0.000 244

10 60 dSPM 70 23.741 0.000 114

10 60 dSPM 80 21.297 0.000 43

10 60 dSPM 90 21.636 11.197 10

10 60 dSPM 100 23.651 23.651 1

10 60 MNE 0 36.609 0.000 8002

10 60 MNE 10 33.696 0.000 1507

10 60 MNE 20 32.264 0.000 554

10 60 MNE 30 31.902 0.000 230

10 60 MNE 40 32.535 0.000 91

10 60 MNE 50 35.534 0.000 37

10 60 MNE 60 34.030 0.000 17

10 60 MNE 70 32.440 0.000 6

10 60 MNE 80 35.593 0.000 4

10 60 MNE 90 35.593 0.000 4

10 60 MNE 100 40.530 40.530 1

10 60 sLORETA 0 33.535 0.000 8002

10 60 sLORETA 10 31.946 0.000 2962

10 60 sLORETA 20 29.607 0.000 1557

10 60 sLORETA 30 28.191 0.000 929

10 60 sLORETA 40 27.353 0.000 549

10 60 sLORETA 50 27.120 0.000 293

10 60 sLORETA 60 26.458 0.000 154

10 60 sLORETA 70 24.966 0.000 78

10 60 sLORETA 80 24.017 5.179 33

10 60 sLORETA 90 19.371 11.197 11

10 60 sLORETA 100 21.624 21.624 1

10 61 Ave 0 40.008 0.000 8002

10 61 Ave 10 39.109 0.000 3985

10 61 Ave 20 36.869 0.000 2058

10 61 Ave 30 32.346 0.000 965

10 61 Ave 40 26.728 0.000 442

10 61 Ave 50 21.200 0.000 221

10 61 Ave 60 18.025 0.000 123

10 61 Ave 70 18.309 0.000 63

10 61 Ave 80 16.899 0.000 24

10 61 Ave 90 18.946 12.608 9

10 61 Ave 100 12.608 12.608 1

10 61 cMEM 0 22.165 0.000 8002

10 61 cMEM 10 16.635 0.000 417

10 61 cMEM 20 11.864 0.000 172

10 61 cMEM 30 12.076 0.000 73

10 61 cMEM 40 10.101 0.000 26

10 61 cMEM 50 8.482 0.000 10

10 61 cMEM 60 2.174 0.000 6

10 61 cMEM 70 2.174 0.000 6

10 61 cMEM 80 0.000 0.000 5

10 61 cMEM 90 0.000 0.000 1

10 61 cMEM 100 0.000 0.000 1

10 61 dSPM 0 42.263 0.000 8002

10 61 dSPM 10 41.832 0.000 4539

10 61 dSPM 20 40.800 0.000 2728

10 61 dSPM 30 39.470 0.000 1683

10 61 dSPM 40 37.251 0.000 975

10 61 dSPM 50 34.250 0.000 568

10 61 dSPM 60 29.952 0.000 289

10 61 dSPM 70 25.186 0.000 149

10 61 dSPM 80 19.888 0.000 81

10 61 dSPM 90 17.039 0.000 30

10 61 dSPM 100 22.298 22.298 1

10 61 MNE 0 39.797 0.000 8002

10 61 MNE 10 37.168 0.000 1696

10 61 MNE 20 34.081 0.000 578

10 61 MNE 30 31.104 0.000 233

10 61 MNE 40 29.951 0.000 99

10 61 MNE 50 26.268 0.000 42

10 61 MNE 60 21.324 0.000 22

10 61 MNE 70 15.751 0.000 5

10 61 MNE 80 0.000 0.000 3

10 61 MNE 90 0.000 0.000 2

10 61 MNE 100 0.000 0.000 1

10 61 sLORETA 0 37.579 0.000 8002

10 61 sLORETA 10 36.282 0.000 3497

10 61 sLORETA 20 32.379 0.000 1473

10 61 sLORETA 30 27.830 0.000 668

10 61 sLORETA 40 24.590 0.000 348

10 61 sLORETA 50 21.277 0.000 202

10 61 sLORETA 60 20.021 0.000 104

10 61 sLORETA 70 19.958 0.000 57

10 61 sLORETA 80 18.861 0.000 29

10 61 sLORETA 90 18.643 12.540 10

10 61 sLORETA 100 12.540 12.540 1

10 62 Ave 0 38.038 0.000 8002

10 62 Ave 10 37.007 0.000 3735

10 62 Ave 20 34.306 0.000 1828

10 62 Ave 30 30.079 0.000 858

10 62 Ave 40 26.860 0.000 423

10 62 Ave 50 24.799 0.000 244

10 62 Ave 60 24.478 0.000 141

10 62 Ave 70 23.482 0.000 73

10 62 Ave 80 21.922 0.000 30

10 62 Ave 90 17.605 4.837 8

10 62 Ave 100 4.837 4.837 1

10 62 cMEM 0 17.135 0.000 8002

10 62 cMEM 10 13.568 0.000 552

10 62 cMEM 20 10.792 0.000 259

10 62 cMEM 30 10.326 0.000 137

10 62 cMEM 40 5.251 0.000 77

10 62 cMEM 50 3.134 0.000 39

10 62 cMEM 60 3.645 0.000 13

10 62 cMEM 70 4.154 0.000 8

10 62 cMEM 80 4.419 0.000 6

10 62 cMEM 90 3.975 0.000 4

10 62 cMEM 100 4.837 4.837 1

10 62 dSPM 0 40.212 0.000 8002

10 62 dSPM 10 39.574 0.000 4116

10 62 dSPM 20 37.988 0.000 2337

10 62 dSPM 30 35.534 0.000 1306

10 62 dSPM 40 30.737 0.000 641

10 62 dSPM 50 26.513 0.000 313

10 62 dSPM 60 24.396 0.000 159

10 62 dSPM 70 24.189 0.000 78

10 62 dSPM 80 24.429 3.872 33

10 62 dSPM 90 23.826 13.180 14

10 62 dSPM 100 22.298 22.298 1

10 62 MNE 0 38.088 0.000 8002

10 62 MNE 10 35.910 0.000 1684

10 62 MNE 20 32.940 0.000 601

10 62 MNE 30 32.512 0.000 279

10 62 MNE 40 32.703 0.000 149

10 62 MNE 50 30.750 0.000 69

10 62 MNE 60 26.880 0.000 25

10 62 MNE 70 25.656 0.000 13

10 62 MNE 80 26.399 16.221 6

10 62 MNE 90 33.193 25.669 2

10 62 MNE 100 25.669 25.669 1

10 62 sLORETA 0 37.421 0.000 8002

10 62 sLORETA 10 36.076 0.000 3129

10 62 sLORETA 20 32.949 0.000 1348

10 62 sLORETA 30 29.997 0.000 633

10 62 sLORETA 40 27.104 0.000 326

10 62 sLORETA 50 26.549 0.000 177

10 62 sLORETA 60 24.766 0.000 81

10 62 sLORETA 70 24.825 5.179 51

10 62 sLORETA 80 21.723 12.540 21

10 62 sLORETA 90 18.766 12.540 5

10 62 sLORETA 100 21.624 21.624 1

10 63 Ave 0 31.493 0.000 8002

10 63 Ave 10 28.607 0.000 2728

10 63 Ave 20 23.209 0.000 1054

10 63 Ave 30 18.180 0.000 471

10 63 Ave 40 16.165 0.000 259

10 63 Ave 50 14.347 0.000 134

10 63 Ave 60 13.703 0.000 61

10 63 Ave 70 14.347 0.000 30

10 63 Ave 80 13.860 0.000 17

10 63 Ave 90 18.126 0.000 6

10 63 Ave 100 19.805 19.805 1

10 63 cMEM 0 11.006 0.000 8002

10 63 cMEM 10 8.156 0.000 316

10 63 cMEM 20 7.719 0.000 130

10 63 cMEM 30 6.190 0.000 72

10 63 cMEM 40 0.907 0.000 43

10 63 cMEM 50 0.535 0.000 27

10 63 cMEM 60 0.000 0.000 19

10 63 cMEM 70 0.000 0.000 11

10 63 cMEM 80 0.000 0.000 7

10 63 cMEM 90 0.000 0.000 2

10 63 cMEM 100 0.000 0.000 1

10 63 dSPM 0 34.846 0.000 8002

10 63 dSPM 10 33.382 0.000 3519

10 63 dSPM 20 30.141 0.000 1767

10 63 dSPM 30 24.842 0.000 863

10 63 dSPM 40 19.376 0.000 406

10 63 dSPM 50 15.805 0.000 204

10 63 dSPM 60 12.997 0.000 94

10 63 dSPM 70 13.956 0.000 39

10 63 dSPM 80 12.222 0.000 14

10 63 dSPM 90 17.504 12.385 4

10 63 dSPM 100 19.805 19.805 1

10 63 MNE 0 29.375 0.000 8002

10 63 MNE 10 24.574 0.000 945

10 63 MNE 20 22.004 0.000 333

10 63 MNE 30 19.047 0.000 161

10 63 MNE 40 18.172 0.000 79

10 63 MNE 50 17.484 0.000 43

10 63 MNE 60 17.849 0.000 20

10 63 MNE 70 16.918 0.000 11

10 63 MNE 80 18.732 0.000 5

10 63 MNE 90 12.422 0.000 2

10 63 MNE 100 0.000 0.000 1

10 63 sLORETA 0 31.275 0.000 8002

10 63 sLORETA 10 28.400 0.000 2577

10 63 sLORETA 20 24.660 0.000 1074

10 63 sLORETA 30 20.760 0.000 545

10 63 sLORETA 40 18.495 0.000 300

10 63 sLORETA 50 17.214 0.000 164

10 63 sLORETA 60 16.661 0.000 77

10 63 sLORETA 70 17.344 0.000 30

10 63 sLORETA 80 18.381 0.000 8

10 63 sLORETA 90 19.674 16.208 4

10 63 sLORETA 100 19.805 19.805 1

10 64 Ave 0 36.294 0.000 8002

10 64 Ave 10 35.098 0.000 3913

10 64 Ave 20 31.946 0.000 1918

10 64 Ave 30 29.083 0.000 1056

10 64 Ave 40 26.617 0.000 560

10 64 Ave 50 23.982 0.000 291

10 64 Ave 60 22.298 0.000 157

10 64 Ave 70 20.507 0.000 77

10 64 Ave 80 17.692 0.000 36

10 64 Ave 90 14.675 0.000 11

10 64 Ave 100 11.197 11.197 1

10 64 cMEM 0 13.675 0.000 8002

10 64 cMEM 10 7.708 0.000 316

10 64 cMEM 20 2.271 0.000 143

10 64 cMEM 30 1.303 0.000 65

10 64 cMEM 40 0.613 0.000 28

10 64 cMEM 50 0.000 0.000 18

10 64 cMEM 60 0.000 0.000 9

10 64 cMEM 70 0.000 0.000 7

10 64 cMEM 80 0.000 0.000 2

10 64 cMEM 90 0.000 0.000 1

10 64 cMEM 100 0.000 0.000 1

10 64 dSPM 0 38.898 0.000 8002

10 64 dSPM 10 38.104 0.000 4124

10 64 dSPM 20 36.262 0.000 2374

10 64 dSPM 30 33.686 0.000 1368

10 64 dSPM 40 30.038 0.000 735

10 64 dSPM 50 26.032 0.000 377

10 64 dSPM 60 22.595 0.000 181

10 64 dSPM 70 19.368 0.000 80

10 64 dSPM 80 16.476 0.000 35

10 64 dSPM 90 15.209 3.872 11

10 64 dSPM 100 10.056 10.056 1

10 64 MNE 0 36.516 0.000 8002

10 64 MNE 10 33.541 0.000 1753

10 64 MNE 20 32.092 0.000 675

10 64 MNE 30 32.382 0.000 313

10 64 MNE 40 31.966 0.000 145

10 64 MNE 50 30.244 0.000 75

10 64 MNE 60 29.043 0.000 34

10 64 MNE 70 31.007 0.000 9

10 64 MNE 80 36.337 17.969 4

10 64 MNE 90 35.635 17.969 3

10 64 MNE 100 42.192 42.192 1

10 64 sLORETA 0 35.367 0.000 8002

10 64 sLORETA 10 33.588 0.000 3205

10 64 sLORETA 20 30.593 0.000 1438

10 64 sLORETA 30 28.624 0.000 759

10 64 sLORETA 40 26.056 0.000 365

10 64 sLORETA 50 24.032 0.000 188

10 64 sLORETA 60 21.764 0.000 98

10 64 sLORETA 70 17.638 0.000 45

10 64 sLORETA 80 17.061 3.872 24

10 64 sLORETA 90 9.782 5.179 5

10 64 sLORETA 100 12.540 12.540 1

11 65 Ave 0 27.247 0.000 8002

11 65 Ave 10 24.468 0.000 1970

11 65 Ave 20 20.695 0.000 930

11 65 Ave 30 15.916 0.000 448

11 65 Ave 40 11.472 0.000 210

11 65 Ave 50 7.005 0.000 105

11 65 Ave 60 3.534 0.000 56

11 65 Ave 70 2.738 0.000 32

11 65 Ave 80 3.494 0.000 16

11 65 Ave 90 0.000 0.000 5

11 65 Ave 100 0.000 0.000 1

11 65 cMEM 0 9.008 0.000 8002

11 65 cMEM 10 8.068 0.000 255

11 65 cMEM 20 7.398 0.000 152

11 65 cMEM 30 5.964 0.000 84

11 65 cMEM 40 1.578 0.000 41

11 65 cMEM 50 1.386 0.000 17

11 65 cMEM 60 1.316 0.000 7

11 65 cMEM 70 0.929 0.000 6

11 65 cMEM 80 1.199 0.000 3

11 65 cMEM 90 0.000 0.000 1

11 65 cMEM 100 0.000 0.000 1

11 65 dSPM 0 30.065 0.000 8002

11 65 dSPM 10 28.192 0.000 2194

11 65 dSPM 20 25.688 0.000 1165

11 65 dSPM 30 21.742 0.000 575

11 65 dSPM 40 17.781 0.000 275

11 65 dSPM 50 15.812 0.000 136

11 65 dSPM 60 15.469 0.000 64

11 65 dSPM 70 12.997 0.000 27

11 65 dSPM 80 13.398 0.000 13

11 65 dSPM 90 0.000 0.000 4

11 65 dSPM 100 0.000 0.000 1

11 65 MNE 0 24.575 0.000 8002

11 65 MNE 10 19.985 0.000 1088

11 65 MNE 20 14.929 0.000 388

11 65 MNE 30 10.727 0.000 185

11 65 MNE 40 7.671 0.000 92

11 65 MNE 50 6.190 0.000 49

11 65 MNE 60 4.579 0.000 30

11 65 MNE 70 4.336 0.000 16

11 65 MNE 80 5.268 0.000 8

11 65 MNE 90 5.574 0.000 4

11 65 MNE 100 0.000 0.000 1

11 65 sLORETA 0 28.397 0.000 8002

11 65 sLORETA 10 25.882 0.000 1972

11 65 sLORETA 20 23.086 0.000 994

11 65 sLORETA 30 19.920 0.000 540

11 65 sLORETA 40 15.343 0.000 255

11 65 sLORETA 50 9.370 0.000 119

11 65 sLORETA 60 4.064 0.000 58

11 65 sLORETA 70 3.033 0.000 32

11 65 sLORETA 80 1.223 0.000 14

11 65 sLORETA 90 0.000 0.000 7

11 65 sLORETA 100 0.000 0.000 1

11 66 Ave 0 27.223 0.000 8002

11 66 Ave 10 24.213 0.000 1821

11 66 Ave 20 20.258 0.000 820

11 66 Ave 30 15.548 0.000 372

11 66 Ave 40 10.401 0.000 161

11 66 Ave 50 5.996 0.000 81

11 66 Ave 60 3.592 0.000 48

11 66 Ave 70 3.275 0.000 24

11 66 Ave 80 3.561 0.000 9

11 66 Ave 90 0.000 0.000 2

11 66 Ave 100 0.000 0.000 1

11 66 cMEM 0 8.551 0.000 8002

11 66 cMEM 10 7.037 0.000 310

11 66 cMEM 20 6.036 0.000 164

11 66 cMEM 30 4.914 0.000 105

11 66 cMEM 40 2.403 0.000 59

11 66 cMEM 50 2.020 0.000 40

11 66 cMEM 60 2.259 0.000 24

11 66 cMEM 70 2.161 0.000 15

11 66 cMEM 80 2.436 0.000 7

11 66 cMEM 90 3.039 0.000 3

11 66 cMEM 100 2.163 2.163 1

11 66 dSPM 0 29.958 0.000 8002

11 66 dSPM 10 28.077 0.000 2112

11 66 dSPM 20 25.619 0.000 1108

11 66 dSPM 30 21.546 0.000 524

11 66 dSPM 40 17.466 0.000 247

11 66 dSPM 50 15.524 0.000 125

11 66 dSPM 60 13.397 0.000 59

11 66 dSPM 70 11.949 0.000 26

11 66 dSPM 80 8.491 0.000 12

11 66 dSPM 90 0.000 0.000 5

11 66 dSPM 100 0.000 0.000 1

11 66 MNE 0 24.219 0.000 8002

11 66 MNE 10 19.500 0.000 999

11 66 MNE 20 14.504 0.000 343

11 66 MNE 30 11.018 0.000 159

11 66 MNE 40 8.281 0.000 80

11 66 MNE 50 6.144 0.000 41

11 66 MNE 60 6.003 0.000 27

11 66 MNE 70 4.544 0.000 12

11 66 MNE 80 5.201 0.000 5

11 66 MNE 90 6.924 0.000 2

11 66 MNE 100 0.000 0.000 1

11 66 sLORETA 0 28.880 0.000 8002

11 66 sLORETA 10 26.440 0.000 1974

11 66 sLORETA 20 23.878 0.000 996

11 66 sLORETA 30 20.800 0.000 548

11 66 sLORETA 40 16.531 0.000 253

11 66 sLORETA 50 10.836 0.000 116

11 66 sLORETA 60 5.102 0.000 56

11 66 sLORETA 70 3.872 0.000 33

11 66 sLORETA 80 2.843 0.000 14

11 66 sLORETA 90 0.000 0.000 7

11 66 sLORETA 100 0.000 0.000 1

11 67 Ave 0 26.952 0.000 8002

11 67 Ave 10 23.446 0.000 1784

11 67 Ave 20 19.433 0.000 789

11 67 Ave 30 14.285 0.000 355

11 67 Ave 40 7.805 0.000 142

11 67 Ave 50 4.458 0.000 76

11 67 Ave 60 2.828 0.000 42

11 67 Ave 70 2.799 0.000 23

11 67 Ave 80 2.616 0.000 13

11 67 Ave 90 0.000 0.000 5

11 67 Ave 100 0.000 0.000 1

11 67 cMEM 0 5.471 0.000 8002

11 67 cMEM 10 4.786 0.000 215

11 67 cMEM 20 4.076 0.000 126

11 67 cMEM 30 2.270 0.000 71

11 67 cMEM 40 0.523 0.000 49

11 67 cMEM 50 0.000 0.000 37

11 67 cMEM 60 0.000 0.000 33

11 67 cMEM 70 0.000 0.000 27

11 67 cMEM 80 0.000 0.000 15

11 67 cMEM 90 0.000 0.000 6

11 67 cMEM 100 0.000 0.000 1

11 67 dSPM 0 29.899 0.000 8002

11 67 dSPM 10 27.843 0.000 2104

11 67 dSPM 20 25.233 0.000 1104

11 67 dSPM 30 21.009 0.000 500

11 67 dSPM 40 16.797 0.000 235

11 67 dSPM 50 15.064 0.000 116

11 67 dSPM 60 13.474 0.000 55

11 67 dSPM 70 11.310 0.000 29

11 67 dSPM 80 7.017 0.000 10

11 67 dSPM 90 0.000 0.000 5

11 67 dSPM 100 0.000 0.000 1

11 67 MNE 0 24.314 0.000 8002

11 67 MNE 10 19.488 0.000 1103

11 67 MNE 20 14.370 0.000 383

11 67 MNE 30 10.934 0.000 178

11 67 MNE 40 8.654 0.000 94

11 67 MNE 50 5.846 0.000 45

11 67 MNE 60 4.657 0.000 29

11 67 MNE 70 4.301 0.000 14

11 67 MNE 80 5.318 0.000 8

11 67 MNE 90 5.849 0.000 3

11 67 MNE 100 0.000 0.000 1

11 67 sLORETA 0 29.200 0.000 8002

11 67 sLORETA 10 26.360 0.000 1999

11 67 sLORETA 20 23.580 0.000 987

11 67 sLORETA 30 20.508 0.000 539

11 67 sLORETA 40 15.678 0.000 246

11 67 sLORETA 50 9.146 0.000 102

11 67 sLORETA 60 4.005 0.000 49

11 67 sLORETA 70 3.187 0.000 28

11 67 sLORETA 80 2.790 0.000 14

11 67 sLORETA 90 0.000 0.000 7

11 67 sLORETA 100 0.000 0.000 1

11 68 Ave 0 26.971 0.000 8002

11 68 Ave 10 24.086 0.000 1893

11 68 Ave 20 20.570 0.000 891

11 68 Ave 30 15.893 0.000 426

11 68 Ave 40 10.670 0.000 180

11 68 Ave 50 6.206 0.000 94

11 68 Ave 60 2.832 0.000 51

11 68 Ave 70 2.745 0.000 27

11 68 Ave 80 2.780 0.000 12

11 68 Ave 90 0.000 0.000 4

11 68 Ave 100 0.000 0.000 1

11 68 cMEM 0 11.168 0.000 8002

11 68 cMEM 10 8.122 0.000 218

11 68 cMEM 20 7.286 0.000 129

11 68 cMEM 30 6.053 0.000 70

11 68 cMEM 40 0.950 0.000 35

11 68 cMEM 50 0.782 0.000 23

11 68 cMEM 60 0.640 0.000 13

11 68 cMEM 70 0.793 0.000 7

11 68 cMEM 80 0.000 0.000 2

11 68 cMEM 90 0.000 0.000 2

11 68 cMEM 100 0.000 0.000 1

11 68 dSPM 0 29.317 0.000 8002

11 68 dSPM 10 27.408 0.000 2033

11 68 dSPM 20 24.564 0.000 1025

11 68 dSPM 30 20.122 0.000 449

11 68 dSPM 40 16.406 0.000 217

11 68 dSPM 50 15.457 0.000 105

11 68 dSPM 60 13.874 0.000 49

11 68 dSPM 70 11.361 0.000 29

11 68 dSPM 80 7.280 0.000 9

11 68 dSPM 90 0.000 0.000 5

11 68 dSPM 100 0.000 0.000 1

11 68 MNE 0 23.736 0.000 8002

11 68 MNE 10 19.167 0.000 1085

11 68 MNE 20 14.335 0.000 387

11 68 MNE 30 10.763 0.000 184

11 68 MNE 40 8.146 0.000 97

11 68 MNE 50 5.837 0.000 51

11 68 MNE 60 3.651 0.000 30

11 68 MNE 70 3.958 0.000 16

11 68 MNE 80 4.277 0.000 7

11 68 MNE 90 2.824 0.000 3

11 68 MNE 100 0.000 0.000 1

11 68 sLORETA 0 28.521 0.000 8002

11 68 sLORETA 10 25.957 0.000 1942

11 68 sLORETA 20 23.401 0.000 971

11 68 sLORETA 30 19.982 0.000 525

11 68 sLORETA 40 14.950 0.000 230

11 68 sLORETA 50 8.170 0.000 90

11 68 sLORETA 60 4.678 0.000 49

11 68 sLORETA 70 3.094 0.000 27

11 68 sLORETA 80 1.252 0.000 13

11 68 sLORETA 90 0.000 0.000 8

11 68 sLORETA 100 0.000 0.000 1

11 69 Ave 0 27.001 0.000 8002

11 69 Ave 10 23.973 0.000 1862

11 69 Ave 20 20.406 0.000 857

11 69 Ave 30 15.660 0.000 399

11 69 Ave 40 9.648 0.000 160

11 69 Ave 50 5.853 0.000 83

11 69 Ave 60 2.730 0.000 45

11 69 Ave 70 2.825 0.000 23

11 69 Ave 80 2.475 0.000 15

11 69 Ave 90 0.000 0.000 5

11 69 Ave 100 0.000 0.000 1

11 69 cMEM 0 7.132 0.000 8002

11 69 cMEM 10 6.349 0.000 256

11 69 cMEM 20 5.630 0.000 151

11 69 cMEM 30 3.631 0.000 92

11 69 cMEM 40 0.541 0.000 54

11 69 cMEM 50 0.000 0.000 37

11 69 cMEM 60 0.000 0.000 33

11 69 cMEM 70 0.000 0.000 28

11 69 cMEM 80 0.000 0.000 16

11 69 cMEM 90 0.000 0.000 6

11 69 cMEM 100 0.000 0.000 1

11 69 dSPM 0 29.867 0.000 8002

11 69 dSPM 10 27.970 0.000 2078

11 69 dSPM 20 25.433 0.000 1063

11 69 dSPM 30 21.035 0.000 471

11 69 dSPM 40 16.815 0.000 214

11 69 dSPM 50 15.562 0.000 107

11 69 dSPM 60 13.688 0.000 47

11 69 dSPM 70 12.264 0.000 24

11 69 dSPM 80 7.635 0.000 8

11 69 dSPM 90 0.000 0.000 5

11 69 dSPM 100 0.000 0.000 1

11 69 MNE 0 23.892 0.000 8002

11 69 MNE 10 19.507 0.000 1127

11 69 MNE 20 15.067 0.000 410

11 69 MNE 30 11.292 0.000 194

11 69 MNE 40 8.907 0.000 97

11 69 MNE 50 5.969 0.000 51

11 69 MNE 60 4.225 0.000 31

11 69 MNE 70 4.041 0.000 16

11 69 MNE 80 4.416 0.000 7

11 69 MNE 90 5.594 0.000 4

11 69 MNE 100 0.000 0.000 1

11 69 sLORETA 0 28.972 0.000 8002

11 69 sLORETA 10 26.381 0.000 2000

11 69 sLORETA 20 24.066 0.000 1015

11 69 sLORETA 30 21.017 0.000 549

11 69 sLORETA 40 16.445 0.000 242

11 69 sLORETA 50 9.337 0.000 97

11 69 sLORETA 60 4.808 0.000 47

11 69 sLORETA 70 2.706 0.000 26

11 69 sLORETA 80 1.358 0.000 11

11 69 sLORETA 90 0.000 0.000 7

11 69 sLORETA 100 0.000 0.000 1

11 70 Ave 0 27.923 0.000 8002

11 70 Ave 10 24.845 0.000 2003

11 70 Ave 20 21.141 0.000 929

11 70 Ave 30 16.491 0.000 440

11 70 Ave 40 10.633 0.000 183

11 70 Ave 50 6.880 0.000 93

11 70 Ave 60 4.572 0.000 50

11 70 Ave 70 2.898 0.000 24

11 70 Ave 80 2.816 0.000 12

11 70 Ave 90 0.000 0.000 5

11 70 Ave 100 0.000 0.000 1

11 70 cMEM 0 13.151 0.000 8002

11 70 cMEM 10 11.581 0.000 296

11 70 cMEM 20 8.999 0.000 207

11 70 cMEM 30 8.343 0.000 148

11 70 cMEM 40 7.878 0.000 107

11 70 cMEM 50 6.397 0.000 71

11 70 cMEM 60 4.336 0.000 38

11 70 cMEM 70 1.418 0.000 28

11 70 cMEM 80 0.706 0.000 18

11 70 cMEM 90 0.000 0.000 9

11 70 cMEM 100 0.000 0.000 1

11 70 dSPM 0 30.613 0.000 8002

11 70 dSPM 10 28.597 0.000 2087

11 70 dSPM 20 25.937 0.000 1052

11 70 dSPM 30 21.340 0.000 459

11 70 dSPM 40 17.167 0.000 202

11 70 dSPM 50 16.069 0.000 102

11 70 dSPM 60 14.633 0.000 44

11 70 dSPM 70 11.968 0.000 23

11 70 dSPM 80 7.683 0.000 8

11 70 dSPM 90 0.000 0.000 4

11 70 dSPM 100 0.000 0.000 1

11 70 MNE 0 24.836 0.000 8002

11 70 MNE 10 20.358 0.000 1232

11 70 MNE 20 15.524 0.000 452

11 70 MNE 30 11.446 0.000 212

11 70 MNE 40 8.792 0.000 109

11 70 MNE 50 5.965 0.000 58

11 70 MNE 60 5.508 0.000 33

11 70 MNE 70 3.985 0.000 17

11 70 MNE 80 4.405 0.000 7

11 70 MNE 90 5.585 0.000 4

11 70 MNE 100 0.000 0.000 1

11 70 sLORETA 0 29.767 0.000 8002

11 70 sLORETA 10 27.093 0.000 2081

11 70 sLORETA 20 24.445 0.000 1038

11 70 sLORETA 30 21.141 0.000 546

11 70 sLORETA 40 16.521 0.000 247

11 70 sLORETA 50 9.502 0.000 95

11 70 sLORETA 60 5.264 0.000 50

11 70 sLORETA 70 2.385 0.000 22

11 70 sLORETA 80 1.290 0.000 12

11 70 sLORETA 90 0.000 0.000 6

11 70 sLORETA 100 0.000 0.000 1

12 71 Ave 0 38.867 0.000 8002

12 71 Ave 10 35.302 0.000 2319

12 71 Ave 20 29.564 0.000 717

12 71 Ave 30 21.417 0.000 253

12 71 Ave 40 15.036 0.000 121

12 71 Ave 50 7.641 0.000 68

12 71 Ave 60 2.999 0.000 45

12 71 Ave 70 2.609 0.000 30

12 71 Ave 80 2.320 0.000 18

12 71 Ave 90 2.987 0.000 10

12 71 Ave 100 0.000 0.000 1

12 71 cMEM 0 32.230 0.000 8002

12 71 cMEM 10 31.861 0.000 217

12 71 cMEM 20 31.807 0.000 140

12 71 cMEM 30 31.564 0.000 91

12 71 cMEM 40 30.668 0.000 64

12 71 cMEM 50 30.865 0.000 46

12 71 cMEM 60 28.348 0.000 31

12 71 cMEM 70 14.136 0.000 21

12 71 cMEM 80 3.262 0.000 14

12 71 cMEM 90 0.000 0.000 6

12 71 cMEM 100 0.000 0.000 1

12 71 dSPM 0 37.183 0.000 8002

12 71 dSPM 10 33.667 0.000 2281

12 71 dSPM 20 28.364 0.000 812

12 71 dSPM 30 24.361 0.000 341

12 71 dSPM 40 18.574 0.000 145

12 71 dSPM 50 10.399 0.000 56

12 71 dSPM 60 3.275 0.000 29

12 71 dSPM 70 1.695 0.000 21

12 71 dSPM 80 1.278 0.000 10

12 71 dSPM 90 0.000 0.000 6

12 71 dSPM 100 0.000 0.000 1

12 71 MNE 0 41.896 0.000 8002

12 71 MNE 10 37.382 0.000 1351

12 71 MNE 20 28.138 0.000 384

12 71 MNE 30 20.169 0.000 145

12 71 MNE 40 8.884 0.000 59

12 71 MNE 50 7.728 0.000 30

12 71 MNE 60 5.474 0.000 20

12 71 MNE 70 4.322 0.000 9

12 71 MNE 80 5.340 4.214 2

12 71 MNE 90 5.340 4.214 2

12 71 MNE 100 4.214 4.214 1

12 71 sLORETA 0 41.976 0.000 8002

12 71 sLORETA 10 40.415 0.000 3392

12 71 sLORETA 20 36.827 0.000 1453

12 71 sLORETA 30 33.421 0.000 660

12 71 sLORETA 40 28.233 0.000 307

12 71 sLORETA 50 11.867 0.000 134

12 71 sLORETA 60 5.743 0.000 73

12 71 sLORETA 70 3.897 0.000 47

12 71 sLORETA 80 3.626 0.000 32

12 71 sLORETA 90 2.162 0.000 10

12 71 sLORETA 100 0.000 0.000 1

12 72 Ave 0 39.692 0.000 8002

12 72 Ave 10 37.629 0.000 2931

12 72 Ave 20 33.200 0.000 1204

12 72 Ave 30 26.277 0.000 463

12 72 Ave 40 18.160 0.000 188

12 72 Ave 50 5.628 0.000 90

12 72 Ave 60 4.567 0.000 63

12 72 Ave 70 3.442 0.000 40

12 72 Ave 80 2.480 0.000 16

12 72 Ave 90 3.201 0.000 6

12 72 Ave 100 0.000 0.000 1

12 72 cMEM 0 16.935 0.000 8002

12 72 cMEM 10 14.524 0.000 214

12 72 cMEM 20 12.983 0.000 141

12 72 cMEM 30 12.218 0.000 111

12 72 cMEM 40 9.770 0.000 83

12 72 cMEM 50 3.784 0.000 59

12 72 cMEM 60 3.962 0.000 41

12 72 cMEM 70 0.832 0.000 24

12 72 cMEM 80 0.000 0.000 10

12 72 cMEM 90 0.000 0.000 5

12 72 cMEM 100 0.000 0.000 1

12 72 dSPM 0 39.509 0.000 8002

12 72 dSPM 10 37.662 0.000 2715

12 72 dSPM 20 34.510 0.000 1098

12 72 dSPM 30 31.310 0.000 503

12 72 dSPM 40 29.881 0.000 254

12 72 dSPM 50 25.985 0.000 106

12 72 dSPM 60 19.575 0.000 45

12 72 dSPM 70 3.575 0.000 20

12 72 dSPM 80 4.262 0.000 11

12 72 dSPM 90 0.000 0.000 3

12 72 dSPM 100 0.000 0.000 1

12 72 MNE 0 40.857 0.000 8002

12 72 MNE 10 36.472 0.000 1633

12 72 MNE 20 30.092 0.000 442

12 72 MNE 30 22.564 0.000 167

12 72 MNE 40 15.949 0.000 69

12 72 MNE 50 8.458 0.000 30

12 72 MNE 60 7.512 0.000 17

12 72 MNE 70 5.859 0.000 4

12 72 MNE 80 5.283 4.214 2

12 72 MNE 90 4.214 4.214 1

12 72 MNE 100 4.214 4.214 1

12 72 sLORETA 0 43.922 0.000 8002

12 72 sLORETA 10 43.066 0.000 3763

12 72 sLORETA 20 40.952 0.000 1939

12 72 sLORETA 30 37.929 0.000 1007

12 72 sLORETA 40 34.075 0.000 474

12 72 sLORETA 50 28.919 0.000 229

12 72 sLORETA 60 22.984 0.000 107

12 72 sLORETA 70 8.489 0.000 49

12 72 sLORETA 80 4.824 0.000 23

12 72 sLORETA 90 3.740 0.000 7

12 72 sLORETA 100 0.000 0.000 1

12 73 Ave 0 36.867 0.000 8002

12 73 Ave 10 33.641 0.000 2217

12 73 Ave 20 26.361 0.000 826

12 73 Ave 30 17.166 0.000 313

12 73 Ave 40 13.955 0.000 157

12 73 Ave 50 10.551 0.000 72

12 73 Ave 60 7.597 0.000 39

12 73 Ave 70 5.704 0.000 17

12 73 Ave 80 4.150 0.000 8

12 73 Ave 90 2.848 0.000 3

12 73 Ave 100 0.000 0.000 1

12 73 cMEM 0 16.026 0.000 8002

12 73 cMEM 10 12.708 0.000 232

12 73 cMEM 20 6.350 0.000 90

12 73 cMEM 30 4.845 0.000 35

12 73 cMEM 40 4.867 0.000 25

12 73 cMEM 50 5.134 0.000 18

12 73 cMEM 60 5.163 0.000 15

12 73 cMEM 70 4.831 0.000 10

12 73 cMEM 80 4.915 2.609 7

12 73 cMEM 90 5.401 4.740 2

12 73 cMEM 100 5.969 5.969 1

12 73 dSPM 0 35.543 0.000 8002

12 73 dSPM 10 32.790 0.000 2366

12 73 dSPM 20 27.611 0.000 984

12 73 dSPM 30 22.034 0.000 452

12 73 dSPM 40 18.009 0.000 196

12 73 dSPM 50 15.887 0.000 104

12 73 dSPM 60 14.479 0.000 60

12 73 dSPM 70 11.982 0.000 34

12 73 dSPM 80 10.343 0.000 19

12 73 dSPM 90 5.986 0.000 5

12 73 dSPM 100 0.000 0.000 1

12 73 MNE 0 38.898 0.000 8002

12 73 MNE 10 33.427 0.000 1146

12 73 MNE 20 25.083 0.000 374

12 73 MNE 30 17.987 0.000 127

12 73 MNE 40 16.042 0.000 58

12 73 MNE 50 9.334 0.000 25

12 73 MNE 60 9.691 0.000 14

12 73 MNE 70 9.579 0.000 5

12 73 MNE 80 4.214 4.214 1

12 73 MNE 90 4.214 4.214 1

12 73 MNE 100 4.214 4.214 1

12 73 sLORETA 0 40.859 0.000 8002

12 73 sLORETA 10 39.310 0.000 2959

12 73 sLORETA 20 35.569 0.000 1337

12 73 sLORETA 30 29.612 0.000 641

12 73 sLORETA 40 21.195 0.000 294

12 73 sLORETA 50 14.118 0.000 122

12 73 sLORETA 60 11.047 0.000 58

12 73 sLORETA 70 7.952 0.000 26

12 73 sLORETA 80 7.049 0.000 11

12 73 sLORETA 90 0.000 0.000 1

12 73 sLORETA 100 0.000 0.000 1

12 74 Ave 0 55.076 0.000 8002

12 74 Ave 10 54.904 0.000 4422

12 74 Ave 20 54.143 0.000 2199

12 74 Ave 30 52.625 0.000 1087

12 74 Ave 40 50.541 0.000 527

12 74 Ave 50 47.554 0.000 192

12 74 Ave 60 49.562 0.000 80

12 74 Ave 70 54.016 0.000 28

12 74 Ave 80 56.380 53.237 9

12 74 Ave 90 58.887 58.350 2

12 74 Ave 100 59.330 59.330 1

12 74 cMEM 0 23.408 0.000 8002

12 74 cMEM 10 21.517 0.000 284

12 74 cMEM 20 18.672 0.000 156

12 74 cMEM 30 18.578 0.000 110

12 74 cMEM 40 15.814 0.000 62

12 74 cMEM 50 10.399 0.000 37

12 74 cMEM 60 0.000 0.000 23

12 74 cMEM 70 0.000 0.000 13

12 74 cMEM 80 0.000 0.000 7

12 74 cMEM 90 0.000 0.000 2

12 74 cMEM 100 0.000 0.000 1

12 74 dSPM 0 49.880 0.000 8002

12 74 dSPM 10 49.456 0.000 4326

12 74 dSPM 20 47.905 0.000 2153

12 74 dSPM 30 44.708 0.000 1029

12 74 dSPM 40 40.385 0.000 530

12 74 dSPM 50 36.717 0.000 290

12 74 dSPM 60 35.432 0.000 163

12 74 dSPM 70 34.455 0.000 77

12 74 dSPM 80 32.947 0.000 28

12 74 dSPM 90 19.791 0.000 6

12 74 dSPM 100 0.000 0.000 1

12 74 MNE 0 60.411 0.000 8002

12 74 MNE 10 60.490 0.000 2604

12 74 MNE 20 60.713 0.000 980

12 74 MNE 30 61.259 0.000 394

12 74 MNE 40 62.769 0.000 160

12 74 MNE 50 63.549 0.000 57

12 74 MNE 60 62.107 9.233 27

12 74 MNE 70 58.201 53.237 9

12 74 MNE 80 57.676 55.455 5

12 74 MNE 90 57.125 55.455 3

12 74 MNE 100 59.330 59.330 1

12 74 sLORETA 0 56.790 0.000 8002

12 74 sLORETA 10 56.691 0.000 4455

12 74 sLORETA 20 56.384 0.000 2366

12 74 sLORETA 30 55.992 0.000 1261

12 74 sLORETA 40 55.634 0.000 626

12 74 sLORETA 50 55.121 0.000 244

12 74 sLORETA 60 55.401 0.000 100

12 74 sLORETA 70 56.619 40.976 43

12 74 sLORETA 80 54.942 45.723 23

12 74 sLORETA 90 57.750 53.261 4

12 74 sLORETA 100 59.330 59.330 1

13 75 Ave 0 24.575 0.000 8002

13 75 Ave 10 21.207 0.000 2308

13 75 Ave 20 16.570 0.000 977

13 75 Ave 30 14.485 0.000 405

13 75 Ave 40 13.499 0.000 183

13 75 Ave 50 12.989 0.000 88

13 75 Ave 60 13.225 0.000 52

13 75 Ave 70 13.760 4.027 27

13 75 Ave 80 14.133 4.027 17

13 75 Ave 90 16.446 11.563 4

13 75 Ave 100 16.020 16.020 1

13 75 cMEM 0 14.075 0.000 8002

13 75 cMEM 10 13.790 0.000 268

13 75 cMEM 20 13.210 0.000 151

13 75 cMEM 30 13.194 0.000 84

13 75 cMEM 40 12.408 0.000 37

13 75 cMEM 50 11.459 4.077 15

13 75 cMEM 60 11.519 7.403 5

13 75 cMEM 70 11.827 9.481 3

13 75 cMEM 80 11.827 9.481 3

13 75 cMEM 90 9.481 9.481 1

13 75 cMEM 100 9.481 9.481 1

13 75 dSPM 0 26.941 0.000 8002

13 75 dSPM 10 25.365 0.000 3561

13 75 dSPM 20 20.913 0.000 1640

13 75 dSPM 30 16.640 0.000 815

13 75 dSPM 40 14.820 0.000 428

13 75 dSPM 50 13.241 0.000 220

13 75 dSPM 60 11.779 0.000 104

13 75 dSPM 70 11.072 0.000 54

13 75 dSPM 80 9.798 0.000 24

13 75 dSPM 90 11.061 6.139 9

13 75 dSPM 100 8.452 8.452 1

13 75 MNE 0 24.301 0.000 8002

13 75 MNE 10 19.287 0.000 848

13 75 MNE 20 15.710 0.000 234

13 75 MNE 30 14.308 0.000 86

13 75 MNE 40 14.247 0.000 41

13 75 MNE 50 14.929 0.000 25

13 75 MNE 60 16.358 0.000 10

13 75 MNE 70 16.519 4.027 4

13 75 MNE 80 17.101 4.027 2

13 75 MNE 90 21.683 21.683 1

13 75 MNE 100 21.683 21.683 1

13 75 sLORETA 0 22.938 0.000 8002

13 75 sLORETA 10 19.862 0.000 2139

13 75 sLORETA 20 16.241 0.000 980

13 75 sLORETA 30 14.683 0.000 428

13 75 sLORETA 40 13.872 0.000 212

13 75 sLORETA 50 12.819 0.000 109

13 75 sLORETA 60 13.389 0.000 58

13 75 sLORETA 70 13.706 0.000 30

13 75 sLORETA 80 14.433 4.027 13

13 75 sLORETA 90 16.337 11.563 5

13 75 sLORETA 100 16.020 16.020 1

13 76 Ave 0 28.580 0.000 8002

13 76 Ave 10 26.209 0.000 3011

13 76 Ave 20 22.367 0.000 1207

13 76 Ave 30 16.749 0.000 455

13 76 Ave 40 9.678 0.000 170

13 76 Ave 50 6.320 0.000 49

13 76 Ave 60 6.054 0.000 19

13 76 Ave 70 5.272 0.000 9

13 76 Ave 80 5.213 0.000 3

13 76 Ave 90 0.000 0.000 1

13 76 Ave 100 0.000 0.000 1

13 76 cMEM 0 12.944 0.000 8002

13 76 cMEM 10 11.752 0.000 804

13 76 cMEM 20 10.264 0.000 358

13 76 cMEM 30 8.965 0.000 175

13 76 cMEM 40 6.584 0.000 82

13 76 cMEM 50 5.515 0.000 44

13 76 cMEM 60 4.496 0.000 19

13 76 cMEM 70 1.092 0.000 10

13 76 cMEM 80 0.000 0.000 3

13 76 cMEM 90 0.000 0.000 2

13 76 cMEM 100 0.000 0.000 1

13 76 dSPM 0 30.736 0.000 8002

13 76 dSPM 10 29.823 0.000 4303

13 76 dSPM 20 27.353 0.000 2196

13 76 dSPM 30 25.223 0.000 1177

13 76 dSPM 40 21.628 0.000 583

13 76 dSPM 50 15.607 0.000 276

13 76 dSPM 60 7.608 0.000 119

13 76 dSPM 70 6.461 0.000 50

13 76 dSPM 80 6.094 0.000 23

13 76 dSPM 90 5.448 0.000 7

13 76 dSPM 100 0.000 0.000 1

13 76 MNE 0 30.073 0.000 8002

13 76 MNE 10 27.134 0.000 1670

13 76 MNE 20 23.327 0.000 513

13 76 MNE 30 20.366 0.000 190

13 76 MNE 40 15.719 0.000 77

13 76 MNE 50 13.341 0.000 37

13 76 MNE 60 8.211 0.000 19

13 76 MNE 70 7.613 0.000 9

13 76 MNE 80 6.884 0.000 4

13 76 MNE 90 6.752 0.000 2

13 76 MNE 100 0.000 0.000 1

13 76 sLORETA 0 29.381 0.000 8002

13 76 sLORETA 10 28.123 0.000 3598

13 76 sLORETA 20 25.986 0.000 1814

13 76 sLORETA 30 23.522 0.000 923

13 76 sLORETA 40 19.058 0.000 408

13 76 sLORETA 50 9.939 0.000 168

13 76 sLORETA 60 7.256 0.000 66

13 76 sLORETA 70 6.187 0.000 34

13 76 sLORETA 80 4.799 0.000 16

13 76 sLORETA 90 3.877 0.000 7

13 76 sLORETA 100 9.810 9.810 1

13 77 Ave 0 23.593 0.000 8002

13 77 Ave 10 20.285 0.000 1848

13 77 Ave 20 17.094 0.000 573

13 77 Ave 30 15.661 0.000 235

13 77 Ave 40 14.897 0.000 145

13 77 Ave 50 14.960 0.000 90

13 77 Ave 60 15.233 0.000 63

13 77 Ave 70 14.482 0.000 38

13 77 Ave 80 14.295 6.139 27

13 77 Ave 90 12.504 8.406 10

13 77 Ave 100 15.024 15.024 1

13 77 cMEM 0 14.392 0.000 8002

13 77 cMEM 10 14.338 0.000 166

13 77 cMEM 20 14.185 0.000 94

13 77 cMEM 30 14.204 0.000 62

13 77 cMEM 40 14.006 0.000 43

13 77 cMEM 50 14.429 4.077 29

13 77 cMEM 60 15.290 4.077 17

13 77 cMEM 70 15.695 6.272 8

13 77 cMEM 80 16.085 12.023 5

13 77 cMEM 90 17.545 16.239 2

13 77 cMEM 100 16.239 16.239 1

13 77 dSPM 0 26.029 0.000 8002

13 77 dSPM 10 23.619 0.000 2556

13 77 dSPM 20 19.006 0.000 947

13 77 dSPM 30 15.727 0.000 387

13 77 dSPM 40 14.262 0.000 183

13 77 dSPM 50 12.832 0.000 100

13 77 dSPM 60 11.561 0.000 56

13 77 dSPM 70 10.732 0.000 30

13 77 dSPM 80 9.713 0.000 12

13 77 dSPM 90 8.466 0.000 7

13 77 dSPM 100 0.000 0.000 1

13 77 MNE 0 23.934 0.000 8002

13 77 MNE 10 20.660 0.000 694

13 77 MNE 20 19.149 0.000 203

13 77 MNE 30 17.980 5.390 107

13 77 MNE 40 17.154 6.139 67

13 77 MNE 50 17.222 7.301 49

13 77 MNE 60 17.972 9.481 30

13 77 MNE 70 18.055 11.611 9

13 77 MNE 80 19.093 13.864 7

13 77 MNE 90 21.468 21.208 2

13 77 MNE 100 21.683 21.683 1

13 77 sLORETA 0 23.015 0.000 8002

13 77 sLORETA 10 20.276 0.000 1982

13 77 sLORETA 20 17.378 0.000 700

13 77 sLORETA 30 15.882 0.000 294

13 77 sLORETA 40 15.386 0.000 168

13 77 sLORETA 50 15.147 0.000 112

13 77 sLORETA 60 15.042 0.000 75

13 77 sLORETA 70 14.511 0.000 44

13 77 sLORETA 80 13.761 0.000 28

13 77 sLORETA 90 13.163 6.139 11

13 77 sLORETA 100 15.024 15.024 1

13 78 Ave 0 29.708 0.000 8002

13 78 Ave 10 28.263 0.000 3301

13 78 Ave 20 25.351 0.000 1396

13 78 Ave 30 22.202 0.000 636

13 78 Ave 40 17.937 0.000 305

13 78 Ave 50 16.326 0.000 159

13 78 Ave 60 15.929 0.000 95

13 78 Ave 70 15.892 0.000 55

13 78 Ave 80 17.369 0.000 26

13 78 Ave 90 17.981 11.840 9

13 78 Ave 100 19.799 19.799 1

13 78 cMEM 0 18.457 0.000 8002

13 78 cMEM 10 18.098 0.000 264

13 78 cMEM 20 18.608 0.000 137

13 78 cMEM 30 19.555 0.000 70

13 78 cMEM 40 20.366 0.000 38

13 78 cMEM 50 21.362 6.272 17

13 78 cMEM 60 22.392 6.272 10

13 78 cMEM 70 23.930 19.799 4

13 78 cMEM 80 24.158 19.799 3

13 78 cMEM 90 23.649 19.799 2

13 78 cMEM 100 19.799 19.799 1

13 78 dSPM 0 31.944 0.000 8002

13 78 dSPM 10 31.020 0.000 3964

13 78 dSPM 20 28.415 0.000 1896

13 78 dSPM 30 24.782 0.000 877

13 78 dSPM 40 21.260 0.000 412

13 78 dSPM 50 17.166 0.000 193

13 78 dSPM 60 13.865 0.000 73

13 78 dSPM 70 12.124 0.000 30

13 78 dSPM 80 13.736 0.000 10

13 78 dSPM 90 13.797 13.357 2

13 78 dSPM 100 14.182 14.182 1

13 78 MNE 0 30.479 0.000 8002

13 78 MNE 10 28.402 0.000 1530

13 78 MNE 20 25.745 0.000 539

13 78 MNE 30 22.326 0.000 239

13 78 MNE 40 17.143 0.000 120

13 78 MNE 50 17.789 0.000 55

13 78 MNE 60 18.010 0.000 37

13 78 MNE 70 17.747 0.000 15

13 78 MNE 80 18.718 14.093 7

13 78 MNE 90 16.832 14.093 3

13 78 MNE 100 17.426 17.426 1

13 78 sLORETA 0 28.781 0.000 8002

13 78 sLORETA 10 27.326 0.000 3122

13 78 sLORETA 20 24.524 0.000 1397

13 78 sLORETA 30 21.245 0.000 662

13 78 sLORETA 40 17.915 0.000 354

13 78 sLORETA 50 15.120 0.000 171

13 78 sLORETA 60 14.430 0.000 93

13 78 sLORETA 70 14.330 0.000 49

13 78 sLORETA 80 16.271 8.292 15

13 78 sLORETA 90 20.721 20.721 1

13 78 sLORETA 100 20.721 20.721 1

13 79 Ave 0 22.621 0.000 8002

13 79 Ave 10 19.494 0.000 2302

13 79 Ave 20 15.591 0.000 796

13 79 Ave 30 13.294 0.000 371

13 79 Ave 40 12.461 0.000 198

13 79 Ave 50 12.785 0.000 130

13 79 Ave 60 13.096 0.000 76

13 79 Ave 70 11.710 0.000 37

13 79 Ave 80 9.527 0.000 18

13 79 Ave 90 6.652 4.027 3

13 79 Ave 100 8.912 8.912 1

13 79 cMEM 0 14.057 0.000 8002

13 79 cMEM 10 13.435 0.000 393

13 79 cMEM 20 12.712 0.000 173

13 79 cMEM 30 11.691 0.000 71

13 79 cMEM 40 11.055 0.000 35

13 79 cMEM 50 11.125 4.935 17

13 79 cMEM 60 9.260 4.935 10

13 79 cMEM 70 7.421 4.935 5

13 79 cMEM 80 5.336 4.935 2

13 79 cMEM 90 4.935 4.935 1

13 79 cMEM 100 4.935 4.935 1

13 79 dSPM 0 24.454 0.000 8002

13 79 dSPM 10 22.418 0.000 3106

13 79 dSPM 20 18.568 0.000 1340

13 79 dSPM 30 14.030 0.000 567

13 79 dSPM 40 11.721 0.000 294

13 79 dSPM 50 10.800 0.000 184

13 79 dSPM 60 11.271 0.000 101

13 79 dSPM 70 9.604 0.000 48

13 79 dSPM 80 9.788 0.000 29

13 79 dSPM 90 5.160 0.000 7

13 79 dSPM 100 0.000 0.000 1

13 79 MNE 0 22.482 0.000 8002

13 79 MNE 10 18.952 0.000 874

13 79 MNE 20 16.211 0.000 305

13 79 MNE 30 15.305 0.000 141

13 79 MNE 40 15.040 0.000 77

13 79 MNE 50 14.480 0.000 45

13 79 MNE 60 13.816 0.000 26

13 79 MNE 70 11.889 0.000 9

13 79 MNE 80 14.676 4.027 4

13 79 MNE 90 4.027 4.027 1

13 79 MNE 100 4.027 4.027 1

13 79 sLORETA 0 21.259 0.000 8002

13 79 sLORETA 10 18.242 0.000 2130

13 79 sLORETA 20 15.064 0.000 811

13 79 sLORETA 30 13.203 0.000 405

13 79 sLORETA 40 12.556 0.000 231

13 79 sLORETA 50 12.048 0.000 143

13 79 sLORETA 60 12.204 0.000 86

13 79 sLORETA 70 12.432 0.000 49

13 79 sLORETA 80 7.524 0.000 23

13 79 sLORETA 90 9.455 5.860 5

13 79 sLORETA 100 11.867 11.867 1

13 80 Ave 0 19.644 0.000 8002

13 80 Ave 10 14.554 0.000 1063

13 80 Ave 20 13.215 0.000 438

13 80 Ave 30 12.958 0.000 258

13 80 Ave 40 13.033 0.000 164

13 80 Ave 50 13.117 0.000 108

13 80 Ave 60 13.123 0.000 67

13 80 Ave 70 13.341 0.000 35

13 80 Ave 80 13.430 0.000 15

13 80 Ave 90 12.947 5.860 5

13 80 Ave 100 11.870 11.870 1

13 80 cMEM 0 13.108 0.000 8002

13 80 cMEM 10 13.094 0.000 208

13 80 cMEM 20 13.083 0.000 133

13 80 cMEM 30 12.853 0.000 92

13 80 cMEM 40 12.730 0.000 67

13 80 cMEM 50 12.507 0.000 40

13 80 cMEM 60 11.476 0.000 23

13 80 cMEM 70 10.889 0.000 10

13 80 cMEM 80 12.364 0.000 5

13 80 cMEM 90 10.385 0.000 2

13 80 cMEM 100 0.000 0.000 1

13 80 dSPM 0 22.599 0.000 8002

13 80 dSPM 10 18.530 0.000 1893

13 80 dSPM 20 12.728 0.000 630

13 80 dSPM 30 11.804 0.000 343

13 80 dSPM 40 11.555 0.000 211

13 80 dSPM 50 11.492 0.000 127

13 80 dSPM 60 11.592 0.000 65

13 80 dSPM 70 10.737 0.000 33

13 80 dSPM 80 10.153 0.000 16

13 80 dSPM 90 10.147 8.406 5

13 80 dSPM 100 8.991 8.991 1

13 80 MNE 0 20.495 0.000 8002

13 80 MNE 10 16.264 0.000 481

13 80 MNE 20 15.599 0.000 216

13 80 MNE 30 15.329 0.000 111

13 80 MNE 40 15.799 0.000 61

13 80 MNE 50 16.338 0.000 33

13 80 MNE 60 16.578 0.000 17

13 80 MNE 70 17.458 8.990 7

13 80 MNE 80 18.414 14.093 4

13 80 MNE 90 21.683 21.683 1

13 80 MNE 100 21.683 21.683 1

13 80 sLORETA 0 19.520 0.000 8002

13 80 sLORETA 10 15.322 0.000 1248

13 80 sLORETA 20 13.514 0.000 523

13 80 sLORETA 30 13.275 0.000 313

13 80 sLORETA 40 13.256 0.000 200

13 80 sLORETA 50 13.361 0.000 125

13 80 sLORETA 60 13.682 0.000 73

13 80 sLORETA 70 13.836 0.000 48

13 80 sLORETA 80 14.446 5.860 28

13 80 sLORETA 90 15.085 9.314 8

13 80 sLORETA 100 11.870 11.870 1

13 81 Ave 0 24.650 0.000 8002

13 81 Ave 10 20.905 0.000 2193

13 81 Ave 20 14.963 0.000 609

13 81 Ave 30 14.001 0.000 223

13 81 Ave 40 14.033 0.000 122

13 81 Ave 50 14.286 0.000 74

13 81 Ave 60 14.506 4.027 48

13 81 Ave 70 14.355 6.139 32

13 81 Ave 80 13.385 6.139 14

13 81 Ave 90 15.273 8.406 4

13 81 Ave 100 15.024 15.024 1

13 81 cMEM 0 13.896 0.000 8002

13 81 cMEM 10 12.711 0.000 273

13 81 cMEM 20 12.816 0.000 131

13 81 cMEM 30 13.135 0.000 74

13 81 cMEM 40 13.210 0.000 37

13 81 cMEM 50 13.039 4.824 21

13 81 cMEM 60 12.735 4.824 12

13 81 cMEM 70 10.054 4.824 6

13 81 cMEM 80 10.786 6.272 4

13 81 cMEM 90 6.272 6.272 1

13 81 cMEM 100 6.272 6.272 1

13 81 dSPM 0 27.417 0.000 8002

13 81 dSPM 10 25.145 0.000 3140

13 81 dSPM 20 20.461 0.000 1248

13 81 dSPM 30 15.357 0.000 469

13 81 dSPM 40 13.586 0.000 191

13 81 dSPM 50 12.797 0.000 79

13 81 dSPM 60 12.603 0.000 47

13 81 dSPM 70 11.703 0.000 22

13 81 dSPM 80 10.753 6.139 7

13 81 dSPM 90 7.306 6.139 2

13 81 dSPM 100 6.139 6.139 1

13 81 MNE 0 24.153 0.000 8002

13 81 MNE 10 19.173 0.000 558

13 81 MNE 20 16.732 0.000 178

13 81 MNE 30 16.391 0.000 94

13 81 MNE 40 16.542 0.000 51

13 81 MNE 50 17.066 4.027 23

13 81 MNE 60 17.370 4.027 10

13 81 MNE 70 18.631 15.024 3

13 81 MNE 80 21.683 21.683 1

13 81 MNE 90 21.683 21.683 1

13 81 MNE 100 21.683 21.683 1

13 81 sLORETA 0 23.937 0.000 8002

13 81 sLORETA 10 20.876 0.000 2323

13 81 sLORETA 20 15.456 0.000 728

13 81 sLORETA 30 14.150 0.000 274

13 81 sLORETA 40 14.057 0.000 156

13 81 sLORETA 50 14.385 0.000 87

13 81 sLORETA 60 14.550 4.027 57

13 81 sLORETA 70 15.711 6.139 30

13 81 sLORETA 80 15.486 6.139 18

13 81 sLORETA 90 15.362 11.467 5

13 81 sLORETA 100 15.024 15.024 1

13 82 Ave 0 16.318 0.000 8002

13 82 Ave 10 13.066 0.000 884

13 82 Ave 20 12.140 0.000 387

13 82 Ave 30 11.809 0.000 179

13 82 Ave 40 11.308 0.000 85

13 82 Ave 50 10.414 0.000 41

13 82 Ave 60 9.942 0.000 21

13 82 Ave 70 9.577 4.027 13

13 82 Ave 80 8.919 5.860 6

13 82 Ave 90 10.246 8.912 3

13 82 Ave 100 11.870 11.870 1

13 82 cMEM 0 10.558 0.000 8002

13 82 cMEM 10 10.381 0.000 103

13 82 cMEM 20 9.920 0.000 53

13 82 cMEM 30 9.659 0.000 33

13 82 cMEM 40 9.541 0.000 22

13 82 cMEM 50 9.856 4.572 18

13 82 cMEM 60 9.290 4.572 15

13 82 cMEM 70 8.810 4.572 9

13 82 cMEM 80 9.181 6.272 3

13 82 cMEM 90 9.288 6.272 2

13 82 cMEM 100 6.272 6.272 1

13 82 dSPM 0 17.467 0.000 8002

13 82 dSPM 10 13.877 0.000 1529

13 82 dSPM 20 12.245 0.000 681

13 82 dSPM 30 11.307 0.000 380

13 82 dSPM 40 10.403 0.000 207

13 82 dSPM 50 9.394 0.000 104

13 82 dSPM 60 8.280 0.000 56

13 82 dSPM 70 8.044 0.000 26

13 82 dSPM 80 8.269 0.000 16

13 82 dSPM 90 6.504 0.000 4

13 82 dSPM 100 0.000 0.000 1

13 82 MNE 0 17.565 0.000 8002

13 82 MNE 10 14.889 0.000 673

13 82 MNE 20 14.489 0.000 292

13 82 MNE 30 14.287 0.000 162

13 82 MNE 40 14.301 0.000 81

13 82 MNE 50 14.451 0.000 47

13 82 MNE 60 14.163 0.000 28

13 82 MNE 70 12.948 0.000 15

13 82 MNE 80 13.125 4.027 10

13 82 MNE 90 10.473 4.027 4

13 82 MNE 100 11.870 11.870 1

13 82 sLORETA 0 16.214 0.000 8002

13 82 sLORETA 10 13.736 0.000 1234

13 82 sLORETA 20 12.831 0.000 590

13 82 sLORETA 30 12.312 0.000 331

13 82 sLORETA 40 12.176 0.000 188

13 82 sLORETA 50 11.312 0.000 104

13 82 sLORETA 60 10.584 0.000 63

13 82 sLORETA 70 9.974 0.000 31

13 82 sLORETA 80 10.329 4.027 18

13 82 sLORETA 90 9.615 5.860 6

13 82 sLORETA 100 11.870 11.870 1

13 83 Ave 0 17.175 0.000 8002

13 83 Ave 10 13.707 0.000 938

13 83 Ave 20 12.271 0.000 431

13 83 Ave 30 11.838 0.000 215

13 83 Ave 40 10.959 0.000 102

13 83 Ave 50 10.127 0.000 62

13 83 Ave 60 9.663 0.000 22

13 83 Ave 70 9.551 0.000 15

13 83 Ave 80 8.984 5.860 5

13 83 Ave 90 10.767 9.314 2

13 83 Ave 100 11.870 11.870 1

13 83 cMEM 0 13.049 0.000 8002

13 83 cMEM 10 13.032 0.000 195

13 83 cMEM 20 13.121 0.000 135

13 83 cMEM 30 13.241 0.000 80

13 83 cMEM 40 12.935 0.000 41

13 83 cMEM 50 13.138 0.000 19

13 83 cMEM 60 12.900 0.000 16

13 83 cMEM 70 13.185 0.000 11

13 83 cMEM 80 15.188 6.272 4

13 83 cMEM 90 11.579 6.272 2

13 83 cMEM 100 6.272 6.272 1

13 83 dSPM 0 18.989 0.000 8002

13 83 dSPM 10 14.937 0.000 1609

13 83 dSPM 20 12.208 0.000 677

13 83 dSPM 30 11.005 0.000 391

13 83 dSPM 40 10.032 0.000 205

13 83 dSPM 50 8.411 0.000 101

13 83 dSPM 60 7.119 0.000 51

13 83 dSPM 70 7.611 0.000 28

13 83 dSPM 80 6.538 0.000 13

13 83 dSPM 90 5.714 0.000 5

13 83 dSPM 100 0.000 0.000 1

13 83 MNE 0 18.370 0.000 8002

13 83 MNE 10 16.165 0.000 725

13 83 MNE 20 15.187 0.000 328

13 83 MNE 30 14.088 0.000 169

13 83 MNE 40 14.300 0.000 85

13 83 MNE 50 14.116 0.000 54

13 83 MNE 60 13.687 0.000 28

13 83 MNE 70 11.970 0.000 15

13 83 MNE 80 12.667 0.000 10

13 83 MNE 90 10.403 4.027 4

13 83 MNE 100 11.870 11.870 1

13 83 sLORETA 0 17.043 0.000 8002

13 83 sLORETA 10 14.228 0.000 1254

13 83 sLORETA 20 12.961 0.000 638

13 83 sLORETA 30 12.229 0.000 367

13 83 sLORETA 40 11.783 0.000 202

13 83 sLORETA 50 10.950 0.000 109

13 83 sLORETA 60 9.646 0.000 66

13 83 sLORETA 70 8.663 0.000 37

13 83 sLORETA 80 9.240 0.000 23

13 83 sLORETA 90 9.498 5.860 7

13 83 sLORETA 100 5.860 5.860 1

13 84 Ave 0 27.021 0.000 8002

13 84 Ave 10 23.621 0.000 2157

13 84 Ave 20 18.622 0.000 586

13 84 Ave 30 15.734 0.000 297

13 84 Ave 40 15.082 0.000 157

13 84 Ave 50 14.525 0.000 78

13 84 Ave 60 13.928 0.000 46

13 84 Ave 70 13.296 4.027 26

13 84 Ave 80 12.406 6.139 14

13 84 Ave 90 11.949 6.272 7

13 84 Ave 100 11.611 11.611 1

13 84 cMEM 0 15.134 0.000 8002

13 84 cMEM 10 14.954 0.000 147

13 84 cMEM 20 14.700 0.000 102

13 84 cMEM 30 14.821 0.000 72

13 84 cMEM 40 14.701 3.933 45

13 84 cMEM 50 14.078 4.824 27

13 84 cMEM 60 13.543 4.935 14

13 84 cMEM 70 14.225 6.272 9

13 84 cMEM 80 13.368 6.272 4

13 84 cMEM 90 6.272 6.272 1

13 84 cMEM 100 6.272 6.272 1

13 84 dSPM 0 29.390 0.000 8002

13 84 dSPM 10 27.053 0.000 3066

13 84 dSPM 20 21.532 0.000 964

13 84 dSPM 30 16.807 0.000 370

13 84 dSPM 40 14.275 0.000 203

13 84 dSPM 50 13.535 0.000 103

13 84 dSPM 60 12.212 0.000 49

13 84 dSPM 70 10.054 0.000 16

13 84 dSPM 80 8.322 6.139 5

13 84 dSPM 90 9.020 6.139 2

13 84 dSPM 100 6.139 6.139 1

13 84 MNE 0 26.898 0.000 8002

13 84 MNE 10 22.360 0.000 755

13 84 MNE 20 18.917 0.000 235

13 84 MNE 30 17.123 0.000 101

13 84 MNE 40 16.738 0.000 56

13 84 MNE 50 16.614 4.027 32

13 84 MNE 60 15.066 4.027 13

13 84 MNE 70 15.254 4.027 10

13 84 MNE 80 18.389 15.513 2

13 84 MNE 90 15.513 15.513 1

13 84 MNE 100 15.513 15.513 1

13 84 sLORETA 0 26.175 0.000 8002

13 84 sLORETA 10 23.252 0.000 2102

13 84 sLORETA 20 18.601 0.000 630

13 84 sLORETA 30 16.032 0.000 323

13 84 sLORETA 40 15.582 0.000 196

13 84 sLORETA 50 14.712 0.000 102

13 84 sLORETA 60 14.431 0.000 53

13 84 sLORETA 70 14.215 4.027 26

13 84 sLORETA 80 12.143 6.139 13

13 84 sLORETA 90 12.736 11.467 3

13 84 sLORETA 100 11.611 11.611 1

13 85 Ave 0 26.187 0.000 8002

13 85 Ave 10 22.914 0.000 2737

13 85 Ave 20 16.896 0.000 988

13 85 Ave 30 14.142 0.000 398

13 85 Ave 40 12.723 0.000 191

13 85 Ave 50 12.918 0.000 91

13 85 Ave 60 13.367 0.000 62

13 85 Ave 70 14.084 0.000 28

13 85 Ave 80 13.298 0.000 11

13 85 Ave 90 12.723 10.774 4

13 85 Ave 100 15.513 15.513 1

13 85 cMEM 0 17.301 0.000 8002

13 85 cMEM 10 16.894 0.000 265

13 85 cMEM 20 16.805 0.000 129

13 85 cMEM 30 16.981 0.000 76

13 85 cMEM 40 17.202 4.935 46

13 85 cMEM 50 16.939 5.581 33

13 85 cMEM 60 16.651 6.219 15

13 85 cMEM 70 16.504 10.774 8

13 85 cMEM 80 14.911 10.774 5

13 85 cMEM 90 14.042 10.774 4

13 85 cMEM 100 12.582 12.582 1

13 85 dSPM 0 28.249 0.000 8002

13 85 dSPM 10 25.942 0.000 3373

13 85 dSPM 20 18.869 0.000 1250

13 85 dSPM 30 14.895 0.000 487

13 85 dSPM 40 11.915 0.000 201

13 85 dSPM 50 11.302 0.000 106

13 85 dSPM 60 11.240 0.000 54

13 85 dSPM 70 10.751 0.000 29

13 85 dSPM 80 5.540 0.000 7

13 85 dSPM 90 0.000 0.000 2

13 85 dSPM 100 0.000 0.000 1

13 85 MNE 0 27.535 0.000 8002

13 85 MNE 10 23.142 0.000 1032

13 85 MNE 20 18.407 0.000 280

13 85 MNE 30 16.347 0.000 109

13 85 MNE 40 15.484 0.000 47

13 85 MNE 50 15.909 0.000 25

13 85 MNE 60 16.196 4.027 13

13 85 MNE 70 16.198 10.774 5

13 85 MNE 80 19.514 15.513 2

13 85 MNE 90 15.513 15.513 1

13 85 MNE 100 15.513 15.513 1

13 85 sLORETA 0 23.911 0.000 8002

13 85 sLORETA 10 20.837 0.000 2582

13 85 sLORETA 20 16.328 0.000 1125

13 85 sLORETA 30 14.100 0.000 518

13 85 sLORETA 40 12.444 0.000 246

13 85 sLORETA 50 12.187 0.000 136

13 85 sLORETA 60 13.055 0.000 78

13 85 sLORETA 70 12.969 0.000 46

13 85 sLORETA 80 12.949 0.000 24

13 85 sLORETA 90 11.626 6.139 8

13 85 sLORETA 100 11.611 11.611 1

13 86 Ave 0 28.052 0.000 8002

13 86 Ave 10 24.270 0.000 2130

13 86 Ave 20 19.828 0.000 602

13 86 Ave 30 16.901 0.000 207

13 86 Ave 40 16.344 0.000 97

13 86 Ave 50 16.234 3.786 53

13 86 Ave 60 15.974 4.027 39

13 86 Ave 70 16.129 6.139 21

13 86 Ave 80 16.824 8.406 11

13 86 Ave 90 17.185 11.611 6

13 86 Ave 100 18.267 18.267 1

13 86 cMEM 0 15.112 0.000 8002

13 86 cMEM 10 14.094 0.000 293

13 86 cMEM 20 13.646 0.000 160

13 86 cMEM 30 13.592 0.000 93

13 86 cMEM 40 13.766 0.000 49

13 86 cMEM 50 13.852 5.770 26

13 86 cMEM 60 14.606 7.251 13

13 86 cMEM 70 16.842 7.945 4

13 86 cMEM 80 16.469 7.945 3

13 86 cMEM 90 12.455 7.945 2

13 86 cMEM 100 15.372 15.372 1

13 86 dSPM 0 30.096 0.000 8002

13 86 dSPM 10 28.112 0.000 3293

13 86 dSPM 20 22.673 0.000 1162

13 86 dSPM 30 18.100 0.000 383

13 86 dSPM 40 15.722 0.000 161

13 86 dSPM 50 14.618 0.000 84

13 86 dSPM 60 15.042 0.000 51

13 86 dSPM 70 14.954 6.139 29

13 86 dSPM 80 13.256 6.139 8

13 86 dSPM 90 9.074 6.139 3

13 86 dSPM 100 11.611 11.611 1

13 86 MNE 0 27.206 0.000 8002

13 86 MNE 10 23.273 0.000 663

13 86 MNE 20 20.135 0.000 184

13 86 MNE 30 18.803 0.000 79

13 86 MNE 40 18.647 4.027 47

13 86 MNE 50 17.623 4.027 27

13 86 MNE 60 17.001 4.027 17

13 86 MNE 70 17.605 11.611 7

13 86 MNE 80 18.823 15.513 3

13 86 MNE 90 18.823 15.513 3

13 86 MNE 100 21.683 21.683 1

13 86 sLORETA 0 26.484 0.000 8002

13 86 sLORETA 10 22.986 0.000 1936

13 86 sLORETA 20 19.080 0.000 590

13 86 sLORETA 30 16.408 0.000 211

13 86 sLORETA 40 16.028 0.000 104

13 86 sLORETA 50 15.984 3.786 59

13 86 sLORETA 60 16.077 4.027 41

13 86 sLORETA 70 16.912 6.139 25

13 86 sLORETA 80 16.934 8.406 11

13 86 sLORETA 90 17.629 11.611 5

13 86 sLORETA 100 19.263 19.263 1

13 87 Ave 0 19.742 0.000 8002

13 87 Ave 10 15.452 0.000 1446

13 87 Ave 20 12.325 0.000 484

13 87 Ave 30 12.238 0.000 270

13 87 Ave 40 12.326 0.000 148

13 87 Ave 50 12.361 0.000 86

13 87 Ave 60 11.890 0.000 49

13 87 Ave 70 11.350 0.000 21

13 87 Ave 80 11.467 6.272 9

13 87 Ave 90 10.667 9.314 2

13 87 Ave 100 9.314 9.314 1

13 87 cMEM 0 14.049 0.000 8002

13 87 cMEM 10 14.116 0.000 194

13 87 cMEM 20 14.463 0.000 96

13 87 cMEM 30 14.406 0.000 50

13 87 cMEM 40 14.791 0.000 26

13 87 cMEM 50 14.238 0.000 16

13 87 cMEM 60 14.769 0.000 14

13 87 cMEM 70 16.871 6.272 6

13 87 cMEM 80 16.768 6.272 3

13 87 cMEM 90 19.554 15.372 2

13 87 cMEM 100 15.372 15.372 1

13 87 dSPM 0 21.624 0.000 8002

13 87 dSPM 10 17.874 0.000 2135

13 87 dSPM 20 12.978 0.000 760

13 87 dSPM 30 11.278 0.000 367

13 87 dSPM 40 10.378 0.000 221

13 87 dSPM 50 9.532 0.000 110

13 87 dSPM 60 8.254 0.000 52

13 87 dSPM 70 7.958 0.000 23

13 87 dSPM 80 7.633 0.000 8

13 87 dSPM 90 6.503 0.000 3

13 87 dSPM 100 8.991 8.991 1

13 87 MNE 0 20.800 0.000 8002

13 87 MNE 10 16.912 0.000 789

13 87 MNE 20 14.992 0.000 276

13 87 MNE 30 14.645 0.000 154

13 87 MNE 40 14.873 0.000 87

13 87 MNE 50 14.924 0.000 53

13 87 MNE 60 15.575 0.000 33

13 87 MNE 70 15.617 4.027 14

13 87 MNE 80 16.119 11.611 7

13 87 MNE 90 18.565 14.093 2

13 87 MNE 100 21.683 21.683 1

13 87 sLORETA 0 19.549 0.000 8002

13 87 sLORETA 10 16.380 0.000 1812

13 87 sLORETA 20 12.793 0.000 681

13 87 sLORETA 30 12.178 0.000 365

13 87 sLORETA 40 12.066 0.000 237

13 87 sLORETA 50 12.267 0.000 151

13 87 sLORETA 60 12.205 0.000 89

13 87 sLORETA 70 11.775 0.000 53

13 87 sLORETA 80 11.406 0.000 24

13 87 sLORETA 90 10.504 6.139 8

13 87 sLORETA 100 9.314 9.314 1

13 88 Ave 0 19.870 0.000 8002

13 88 Ave 10 15.670 0.000 1610

13 88 Ave 20 12.764 0.000 615

13 88 Ave 30 11.761 0.000 298

13 88 Ave 40 10.457 0.000 167

13 88 Ave 50 10.374 0.000 112

13 88 Ave 60 9.303 0.000 57

13 88 Ave 70 9.026 0.000 33

13 88 Ave 80 8.030 0.000 19

13 88 Ave 90 6.105 0.000 6

13 88 Ave 100 4.572 4.572 1

13 88 cMEM 0 10.081 0.000 8002

13 88 cMEM 10 9.165 0.000 318

13 88 cMEM 20 8.850 0.000 163

13 88 cMEM 30 8.306 0.000 110

13 88 cMEM 40 7.337 0.000 68

13 88 cMEM 50 5.916 0.000 42

13 88 cMEM 60 3.932 0.000 27

13 88 cMEM 70 2.666 0.000 12

13 88 cMEM 80 2.703 0.000 8

13 88 cMEM 90 2.611 0.000 3

13 88 cMEM 100 0.000 0.000 1

13 88 dSPM 0 22.517 0.000 8002

13 88 dSPM 10 18.935 0.000 2389

13 88 dSPM 20 13.612 0.000 867

13 88 dSPM 30 10.721 0.000 399

13 88 dSPM 40 9.088 0.000 211

13 88 dSPM 50 7.450 0.000 104

13 88 dSPM 60 6.608 0.000 60

13 88 dSPM 70 5.704 0.000 29

13 88 dSPM 80 5.418 0.000 10

13 88 dSPM 90 5.719 0.000 6

13 88 dSPM 100 0.000 0.000 1

13 88 MNE 0 22.360 0.000 8002

13 88 MNE 10 19.220 0.000 1101

13 88 MNE 20 17.220 0.000 410

13 88 MNE 30 16.267 0.000 216

13 88 MNE 40 16.202 0.000 125

13 88 MNE 50 16.080 0.000 72

13 88 MNE 60 14.126 0.000 41

13 88 MNE 70 14.136 0.000 32

13 88 MNE 80 13.567 0.000 16

13 88 MNE 90 14.306 0.000 8

13 88 MNE 100 15.513 15.513 1

13 88 sLORETA 0 19.513 0.000 8002

13 88 sLORETA 10 16.234 0.000 1879

13 88 sLORETA 20 13.281 0.000 763

13 88 sLORETA 30 11.916 0.000 384

13 88 sLORETA 40 10.676 0.000 221

13 88 sLORETA 50 10.159 0.000 136

13 88 sLORETA 60 9.470 0.000 77

13 88 sLORETA 70 8.831 0.000 44

13 88 sLORETA 80 7.928 0.000 22

13 88 sLORETA 90 7.324 0.000 8

13 88 sLORETA 100 9.314 9.314 1

13 89 Ave 0 29.919 0.000 8002

13 89 Ave 10 28.665 0.000 4009

13 89 Ave 20 24.096 0.000 1735

13 89 Ave 30 19.129 0.000 772

13 89 Ave 40 17.555 0.000 390

13 89 Ave 50 16.481 0.000 186

13 89 Ave 60 16.165 0.000 92

13 89 Ave 70 15.382 0.000 41

13 89 Ave 80 14.017 0.000 14

13 89 Ave 90 9.990 9.154 3

13 89 Ave 100 11.090 11.090 1

13 89 cMEM 0 16.134 0.000 8002

13 89 cMEM 10 15.848 0.000 326

13 89 cMEM 20 16.002 0.000 160

13 89 cMEM 30 16.058 0.000 73

13 89 cMEM 40 16.689 0.000 41

13 89 cMEM 50 16.775 4.077 28

13 89 cMEM 60 17.567 4.077 18

13 89 cMEM 70 19.065 4.572 6

13 89 cMEM 80 18.914 6.272 4

13 89 cMEM 90 23.557 19.799 2

13 89 cMEM 100 19.799 19.799 1

13 89 dSPM 0 32.366 0.000 8002

13 89 dSPM 10 31.565 0.000 4329

13 89 dSPM 20 28.923 0.000 2219

13 89 dSPM 30 25.124 0.000 1089

13 89 dSPM 40 20.854 0.000 486

13 89 dSPM 50 17.315 0.000 223

13 89 dSPM 60 12.890 0.000 101

13 89 dSPM 70 10.442 0.000 34

13 89 dSPM 80 6.402 0.000 8

13 89 dSPM 90 0.000 0.000 2

13 89 dSPM 100 0.000 0.000 1

13 89 MNE 0 29.735 0.000 8002

13 89 MNE 10 27.048 0.000 1820

13 89 MNE 20 22.726 0.000 654

13 89 MNE 30 18.551 0.000 254

13 89 MNE 40 17.361 0.000 111

13 89 MNE 50 17.534 0.000 65

13 89 MNE 60 18.291 4.348 32

13 89 MNE 70 18.480 9.481 19

13 89 MNE 80 21.500 18.987 5

13 89 MNE 90 21.466 21.208 2

13 89 MNE 100 21.683 21.683 1

13 89 sLORETA 0 28.447 0.000 8002

13 89 sLORETA 10 27.216 0.000 3989

13 89 sLORETA 20 23.335 0.000 1805

13 89 sLORETA 30 19.447 0.000 918

13 89 sLORETA 40 18.058 0.000 496

13 89 sLORETA 50 17.438 0.000 263

13 89 sLORETA 60 16.677 0.000 145

13 89 sLORETA 70 15.267 0.000 71

13 89 sLORETA 80 13.375 0.000 31

13 89 sLORETA 90 14.235 0.000 9

13 89 sLORETA 100 0.000 0.000 1

14 90 Ave 0 42.619 0.000 8002

14 90 Ave 10 40.785 0.000 3582

14 90 Ave 20 33.771 0.000 1459

14 90 Ave 30 24.008 0.000 625

14 90 Ave 40 11.474 0.000 267

14 90 Ave 50 7.652 0.000 125

14 90 Ave 60 3.489 0.000 61

14 90 Ave 70 1.647 0.000 28

14 90 Ave 80 0.000 0.000 13

14 90 Ave 90 0.000 0.000 6

14 90 Ave 100 0.000 0.000 1

14 90 cMEM 0 8.480 0.000 8002

14 90 cMEM 10 7.517 0.000 317

14 90 cMEM 20 6.381 0.000 159

14 90 cMEM 30 6.019 0.000 101

14 90 cMEM 40 5.347 0.000 60

14 90 cMEM 50 4.760 0.000 47

14 90 cMEM 60 4.037 0.000 26

14 90 cMEM 70 3.759 0.000 16

14 90 cMEM 80 2.529 0.000 5

14 90 cMEM 90 0.000 0.000 3

14 90 cMEM 100 0.000 0.000 1

14 90 dSPM 0 43.812 0.000 8002

14 90 dSPM 10 42.250 0.000 3710

14 90 dSPM 20 36.884 0.000 1653

14 90 dSPM 30 30.702 0.000 726

14 90 dSPM 40 24.952 0.000 335

14 90 dSPM 50 18.072 0.000 148

14 90 dSPM 60 9.600 0.000 55

14 90 dSPM 70 9.213 0.000 24

14 90 dSPM 80 7.655 0.000 9

14 90 dSPM 90 7.859 4.574 2

14 90 dSPM 100 4.574 4.574 1

14 90 MNE 0 42.819 0.000 8002

14 90 MNE 10 40.242 0.000 2203

14 90 MNE 20 32.039 0.000 790

14 90 MNE 30 22.383 0.000 327

14 90 MNE 40 14.513 0.000 160

14 90 MNE 50 8.124 0.000 74

14 90 MNE 60 5.898 0.000 37

14 90 MNE 70 4.409 0.000 19

14 90 MNE 80 2.580 0.000 10

14 90 MNE 90 0.000 0.000 4

14 90 MNE 100 0.000 0.000 1

14 90 sLORETA 0 45.335 0.000 8002

14 90 sLORETA 10 44.221 0.000 4004

14 90 sLORETA 20 39.887 0.000 1896

14 90 sLORETA 30 33.259 0.000 897

14 90 sLORETA 40 26.124 0.000 415

14 90 sLORETA 50 12.730 0.000 169

14 90 sLORETA 60 7.736 0.000 76

14 90 sLORETA 70 2.527 0.000 34

14 90 sLORETA 80 0.000 0.000 20

14 90 sLORETA 90 0.000 0.000 6

14 90 sLORETA 100 0.000 0.000 1

14 91 Ave 0 49.270 0.000 8002

14 91 Ave 10 48.368 0.000 4033

14 91 Ave 20 45.216 0.000 1851

14 91 Ave 30 38.631 0.000 779

14 91 Ave 40 31.449 0.000 329

14 91 Ave 50 19.136 0.000 129

14 91 Ave 60 9.771 0.000 58

14 91 Ave 70 1.560 0.000 24

14 91 Ave 80 0.000 0.000 7

14 91 Ave 90 0.000 0.000 3

14 91 Ave 100 0.000 0.000 1

14 91 cMEM 0 17.301 0.000 8002

14 91 cMEM 10 8.097 0.000 237

14 91 cMEM 20 4.360 0.000 102

14 91 cMEM 30 2.918 0.000 65

14 91 cMEM 40 2.076 0.000 47

14 91 cMEM 50 0.000 0.000 29

14 91 cMEM 60 0.000 0.000 18

14 91 cMEM 70 0.000 0.000 8

14 91 cMEM 80 0.000 0.000 3

14 91 cMEM 90 0.000 0.000 3

14 91 cMEM 100 0.000 0.000 1

14 91 dSPM 0 49.465 0.000 8002

14 91 dSPM 10 48.596 0.000 4078

14 91 dSPM 20 46.144 0.000 2032

14 91 dSPM 30 39.867 0.000 878

14 91 dSPM 40 29.668 0.000 330

14 91 dSPM 50 17.656 0.000 121

14 91 dSPM 60 10.563 0.000 39

14 91 dSPM 70 8.483 0.000 18

14 91 dSPM 80 8.085 0.000 9

14 91 dSPM 90 6.428 0.000 5

14 91 dSPM 100 4.574 4.574 1

14 91 MNE 0 51.721 0.000 8002

14 91 MNE 10 50.797 0.000 2628

14 91 MNE 20 48.356 0.000 1019

14 91 MNE 30 45.790 0.000 505

14 91 MNE 40 42.186 0.000 256

14 91 MNE 50 40.818 0.000 113

14 91 MNE 60 39.383 0.000 57

14 91 MNE 70 40.612 0.000 24

14 91 MNE 80 41.586 0.000 11

14 91 MNE 90 5.535 0.000 3

14 91 MNE 100 8.412 8.412 1

14 91 sLORETA 0 51.041 0.000 8002

14 91 sLORETA 10 50.590 0.000 4749

14 91 sLORETA 20 49.145 0.000 2691

14 91 sLORETA 30 46.319 0.000 1474

14 91 sLORETA 40 42.890 0.000 779

14 91 sLORETA 50 37.108 0.000 370

14 91 sLORETA 60 28.733 0.000 154

14 91 sLORETA 70 14.049 0.000 62

14 91 sLORETA 80 3.388 0.000 21

14 91 sLORETA 90 0.000 0.000 3

14 91 sLORETA 100 0.000 0.000 1

14 92 Ave 0 46.540 0.000 8002

14 92 Ave 10 45.302 0.000 4226

14 92 Ave 20 39.301 0.000 1847

14 92 Ave 30 30.872 0.000 773

14 92 Ave 40 20.856 0.000 309

14 92 Ave 50 16.566 0.000 139

14 92 Ave 60 17.688 0.000 67

14 92 Ave 70 18.635 0.000 34

14 92 Ave 80 19.687 8.052 13

14 92 Ave 90 22.037 18.366 4

14 92 Ave 100 18.366 18.366 1

14 92 cMEM 0 19.230 0.000 8002

14 92 cMEM 10 18.237 0.000 219

14 92 cMEM 20 17.981 0.000 130

14 92 cMEM 30 18.715 0.000 71

14 92 cMEM 40 19.994 0.000 37

14 92 cMEM 50 20.419 0.000 26

14 92 cMEM 60 21.153 7.208 19

14 92 cMEM 70 21.537 18.366 12

14 92 cMEM 80 21.821 18.366 10

14 92 cMEM 90 23.075 21.505 4

14 92 cMEM 100 21.505 21.505 1

14 92 dSPM 0 48.314 0.000 8002

14 92 dSPM 10 46.678 0.000 3371

14 92 dSPM 20 42.955 0.000 1323

14 92 dSPM 30 38.441 0.000 550

14 92 dSPM 40 29.416 0.000 248

14 92 dSPM 50 23.005 0.000 104

14 92 dSPM 60 22.341 0.000 45

14 92 dSPM 70 21.722 6.484 28

14 92 dSPM 80 22.722 15.768 14

14 92 dSPM 90 21.016 15.768 5

14 92 dSPM 100 20.946 20.946 1

14 92 MNE 0 49.585 0.000 8002

14 92 MNE 10 47.292 0.000 2744

14 92 MNE 20 41.389 0.000 882

14 92 MNE 30 32.079 0.000 301

14 92 MNE 40 25.666 0.000 123

14 92 MNE 50 14.662 0.000 42

14 92 MNE 60 8.650 0.000 15

14 92 MNE 70 8.612 0.000 8

14 92 MNE 80 12.006 8.145 3

14 92 MNE 90 8.412 8.412 1

14 92 MNE 100 8.412 8.412 1

14 92 sLORETA 0 47.420 0.000 8002

14 92 sLORETA 10 46.827 0.000 5009

14 92 sLORETA 20 43.546 0.000 2766

14 92 sLORETA 30 38.182 0.000 1432

14 92 sLORETA 40 34.602 0.000 786

14 92 sLORETA 50 28.622 0.000 379

14 92 sLORETA 60 19.992 0.000 176

14 92 sLORETA 70 17.151 0.000 79

14 92 sLORETA 80 16.455 0.000 30

14 92 sLORETA 90 14.292 0.000 10

14 92 sLORETA 100 6.484 6.484 1

15 93 Ave 0 31.464 0.000 8003

15 93 Ave 10 29.731 0.000 2569

15 93 Ave 20 22.709 0.000 941

15 93 Ave 30 9.650 0.000 324

15 93 Ave 40 3.434 0.000 115

15 93 Ave 50 1.326 0.000 50

15 93 Ave 60 0.000 0.000 23

15 93 Ave 70 0.000 0.000 14

15 93 Ave 80 0.000 0.000 6

15 93 Ave 90 0.000 0.000 2

15 93 Ave 100 0.000 0.000 1

15 93 cMEM 0 4.483 0.000 8003

15 93 cMEM 10 2.525 0.000 244

15 93 cMEM 20 0.000 0.000 110

15 93 cMEM 30 0.000 0.000 53

15 93 cMEM 40 0.000 0.000 36

15 93 cMEM 50 0.000 0.000 25

15 93 cMEM 60 0.000 0.000 18

15 93 cMEM 70 0.000 0.000 12

15 93 cMEM 80 0.000 0.000 8

15 93 cMEM 90 0.000 0.000 4

15 93 cMEM 100 0.000 0.000 1

15 93 dSPM 0 33.471 0.000 8003

15 93 dSPM 10 32.701 0.000 3371

15 93 dSPM 20 29.901 0.000 1649

15 93 dSPM 30 22.874 0.000 725

15 93 dSPM 40 10.699 0.000 289

15 93 dSPM 50 8.759 0.000 152

15 93 dSPM 60 5.798 0.000 66

15 93 dSPM 70 3.826 0.000 23

15 93 dSPM 80 3.581 0.000 10

15 93 dSPM 90 0.000 0.000 3

15 93 dSPM 100 0.000 0.000 1

15 93 MNE 0 26.553 0.000 8003

15 93 MNE 10 20.902 0.000 594

15 93 MNE 20 11.858 0.000 156

15 93 MNE 30 4.156 0.000 54

15 93 MNE 40 0.000 0.000 26

15 93 MNE 50 0.000 0.000 18

15 93 MNE 60 0.000 0.000 11

15 93 MNE 70 0.000 0.000 8

15 93 MNE 80 0.000 0.000 3

15 93 MNE 90 0.000 0.000 2

15 93 MNE 100 0.000 0.000 1

15 93 sLORETA 0 31.380 0.000 8003

15 93 sLORETA 10 29.941 0.000 2762

15 93 sLORETA 20 25.132 0.000 1154

15 93 sLORETA 30 14.909 0.000 488

15 93 sLORETA 40 7.503 0.000 177

15 93 sLORETA 50 2.024 0.000 76

15 93 sLORETA 60 0.000 0.000 35

15 93 sLORETA 70 0.000 0.000 21

15 93 sLORETA 80 0.000 0.000 9

15 93 sLORETA 90 0.000 0.000 4

15 93 sLORETA 100 0.000 0.000 1

15 94 Ave 0 25.310 0.000 8003

15 94 Ave 10 20.556 0.000 1665

15 94 Ave 20 8.241 0.000 501

15 94 Ave 30 3.431 0.000 183

15 94 Ave 40 0.505 0.000 79

15 94 Ave 50 0.000 0.000 40

15 94 Ave 60 0.000 0.000 23

15 94 Ave 70 0.000 0.000 13

15 94 Ave 80 0.000 0.000 5

15 94 Ave 90 0.000 0.000 3

15 94 Ave 100 0.000 0.000 1

15 94 cMEM 0 2.427 0.000 8003

15 94 cMEM 10 0.000 0.000 165

15 94 cMEM 20 0.000 0.000 74

15 94 cMEM 30 0.000 0.000 48

15 94 cMEM 40 0.000 0.000 31

15 94 cMEM 50 0.000 0.000 18

15 94 cMEM 60 0.000 0.000 13

15 94 cMEM 70 0.000 0.000 7

15 94 cMEM 80 0.000 0.000 2

15 94 cMEM 90 0.000 0.000 1

15 94 cMEM 100 0.000 0.000 1

15 94 dSPM 0 27.341 0.000 8003

15 94 dSPM 10 24.596 0.000 2243

15 94 dSPM 20 14.436 0.000 724

15 94 dSPM 30 7.488 0.000 351

15 94 dSPM 40 4.853 0.000 153

15 94 dSPM 50 2.596 0.000 57

15 94 dSPM 60 0.758 0.000 23

15 94 dSPM 70 0.000 0.000 13

15 94 dSPM 80 0.000 0.000 7

15 94 dSPM 90 0.000 0.000 2

15 94 dSPM 100 0.000 0.000 1

15 94 MNE 0 21.030 0.000 8003

15 94 MNE 10 12.829 0.000 422

15 94 MNE 20 4.256 0.000 112

15 94 MNE 30 0.000 0.000 50

15 94 MNE 40 0.000 0.000 27

15 94 MNE 50 0.000 0.000 18

15 94 MNE 60 0.000 0.000 13

15 94 MNE 70 0.000 0.000 6

15 94 MNE 80 0.000 0.000 3

15 94 MNE 90 0.000 0.000 2

15 94 MNE 100 0.000 0.000 1

15 94 sLORETA 0 25.415 0.000 8003

15 94 sLORETA 10 21.881 0.000 1892

15 94 sLORETA 20 10.292 0.000 621

15 94 sLORETA 30 5.862 0.000 256

15 94 sLORETA 40 2.124 0.000 111

15 94 sLORETA 50 0.445 0.000 59

15 94 sLORETA 60 0.000 0.000 33

15 94 sLORETA 70 0.000 0.000 17

15 94 sLORETA 80 0.000 0.000 12

15 94 sLORETA 90 0.000 0.000 5

15 94 sLORETA 100 0.000 0.000 1

15 95 Ave 0 27.139 0.000 8003

15 95 Ave 10 25.300 0.000 3053

15 95 Ave 20 20.730 0.000 1262

15 95 Ave 30 12.358 0.000 533

15 95 Ave 40 5.179 0.000 264

15 95 Ave 50 3.124 0.000 126

15 95 Ave 60 0.000 0.000 60

15 95 Ave 70 0.000 0.000 40

15 95 Ave 80 0.000 0.000 15

15 95 Ave 90 0.000 0.000 6

15 95 Ave 100 0.000 0.000 1

15 95 cMEM 0 1.410 0.000 8003

15 95 cMEM 10 1.011 0.000 306

15 95 cMEM 20 0.000 0.000 150

15 95 cMEM 30 0.000 0.000 83

15 95 cMEM 40 0.000 0.000 42

15 95 cMEM 50 0.000 0.000 26

15 95 cMEM 60 0.000 0.000 9

15 95 cMEM 70 0.000 0.000 5

15 95 cMEM 80 0.000 0.000 2

15 95 cMEM 90 0.000 0.000 2

15 95 cMEM 100 0.000 0.000 1

15 95 dSPM 0 29.216 0.000 8003

15 95 dSPM 10 28.122 0.000 3532

15 95 dSPM 20 25.439 0.000 1779

15 95 dSPM 30 20.261 0.000 845

15 95 dSPM 40 12.863 0.000 400

15 95 dSPM 50 9.106 0.000 216

15 95 dSPM 60 5.982 0.000 125

15 95 dSPM 70 5.089 0.000 69

15 95 dSPM 80 3.442 0.000 36

15 95 dSPM 90 4.208 0.000 13

15 95 dSPM 100 0.000 0.000 1

15 95 MNE 0 24.492 0.000 8003

15 95 MNE 10 20.261 0.000 945

15 95 MNE 20 14.428 0.000 267

15 95 MNE 30 7.887 0.000 109

15 95 MNE 40 0.000 0.000 55

15 95 MNE 50 0.000 0.000 32

15 95 MNE 60 0.000 0.000 20

15 95 MNE 70 0.000 0.000 12

15 95 MNE 80 0.000 0.000 11

15 95 MNE 90 0.000 0.000 4

15 95 MNE 100 0.000 0.000 1

15 95 sLORETA 0 28.615 0.000 8003

15 95 sLORETA 10 27.178 0.000 3207

15 95 sLORETA 20 24.144 0.000 1482

15 95 sLORETA 30 19.122 0.000 762

15 95 sLORETA 40 11.793 0.000 348

15 95 sLORETA 50 7.538 0.000 175

15 95 sLORETA 60 4.807 0.000 85

15 95 sLORETA 70 0.000 0.000 37

15 95 sLORETA 80 0.000 0.000 20

15 95 sLORETA 90 0.000 0.000 8

15 95 sLORETA 100 0.000 0.000 1

15 96 Ave 0 38.446 0.000 8003

15 96 Ave 10 38.208 0.000 4645

15 96 Ave 20 37.573 0.000 2938

15 96 Ave 30 36.356 0.000 1886

15 96 Ave 40 34.613 0.000 1150

15 96 Ave 50 31.331 0.000 656

15 96 Ave 60 25.838 0.000 329

15 96 Ave 70 19.565 0.000 129

15 96 Ave 80 17.302 0.000 62

15 96 Ave 90 13.199 0.000 14

15 96 Ave 100 11.653 11.653 1

15 96 cMEM 0 21.479 0.000 8003

15 96 cMEM 10 20.481 0.000 631

15 96 cMEM 20 20.954 0.000 259

15 96 cMEM 30 21.494 0.000 140

15 96 cMEM 40 22.371 0.000 84

15 96 cMEM 50 23.233 0.000 45

15 96 cMEM 60 23.130 0.000 38

15 96 cMEM 70 23.478 0.000 26

15 96 cMEM 80 22.406 0.000 13

15 96 cMEM 90 19.558 0.000 4

15 96 cMEM 100 0.000 0.000 1

15 96 dSPM 0 39.686 0.000 8003

15 96 dSPM 10 39.429 0.000 3987

15 96 dSPM 20 38.737 0.000 2299

15 96 dSPM 30 37.413 0.000 1293

15 96 dSPM 40 35.021 0.000 659

15 96 dSPM 50 30.589 0.000 278

15 96 dSPM 60 21.644 0.000 112

15 96 dSPM 70 12.145 0.000 35

15 96 dSPM 80 9.196 0.000 12

15 96 dSPM 90 9.159 0.000 4

15 96 dSPM 100 0.000 0.000 1

15 96 MNE 0 38.749 0.000 8003

15 96 MNE 10 37.670 0.000 2227

15 96 MNE 20 37.550 0.000 863

15 96 MNE 30 37.402 0.000 333

15 96 MNE 40 36.694 0.000 137

15 96 MNE 50 36.557 0.000 68

15 96 MNE 60 30.795 0.000 22

15 96 MNE 70 16.721 0.000 14

15 96 MNE 80 0.000 0.000 7

15 96 MNE 90 0.000 0.000 3

15 96 MNE 100 0.000 0.000 1

15 96 sLORETA 0 38.171 0.000 8003

15 96 sLORETA 10 37.834 0.000 4252

15 96 sLORETA 20 37.048 0.000 2582

15 96 sLORETA 30 35.679 0.000 1538

15 96 sLORETA 40 32.680 0.000 848

15 96 sLORETA 50 26.793 0.000 430

15 96 sLORETA 60 22.768 0.000 199

15 96 sLORETA 70 20.220 0.000 87

15 96 sLORETA 80 20.363 0.000 38

15 96 sLORETA 90 23.337 0.000 8

15 96 sLORETA 100 3.813 3.813 1

15 97 Ave 0 39.333 0.000 8003

15 97 Ave 10 39.006 0.000 4073

15 97 Ave 20 38.329 0.000 2240

15 97 Ave 30 36.141 0.000 1149

15 97 Ave 40 31.828 0.000 481

15 97 Ave 50 21.021 0.000 166

15 97 Ave 60 3.930 0.000 49

15 97 Ave 70 0.000 0.000 15

15 97 Ave 80 0.000 0.000 6

15 97 Ave 90 0.000 0.000 2

15 97 Ave 100 0.000 0.000 1

15 97 cMEM 0 11.364 0.000 8003

15 97 cMEM 10 9.509 0.000 609

15 97 cMEM 20 8.234 0.000 288

15 97 cMEM 30 5.554 0.000 124

15 97 cMEM 40 1.827 0.000 63

15 97 cMEM 50 0.000 0.000 36

15 97 cMEM 60 0.000 0.000 23

15 97 cMEM 70 0.000 0.000 19

15 97 cMEM 80 0.000 0.000 10

15 97 cMEM 90 0.000 0.000 6

15 97 cMEM 100 0.000 0.000 1

15 97 dSPM 0 41.516 0.000 8003

15 97 dSPM 10 41.357 0.000 4111

15 97 dSPM 20 41.099 0.000 2405

15 97 dSPM 30 40.292 0.000 1219

15 97 dSPM 40 39.245 0.000 578

15 97 dSPM 50 34.455 0.000 227

15 97 dSPM 60 20.829 0.000 84

15 97 dSPM 70 12.593 0.000 33

15 97 dSPM 80 0.000 0.000 9

15 97 dSPM 90 0.000 0.000 1

15 97 dSPM 100 0.000 0.000 1

15 97 MNE 0 37.040 0.000 8003

15 97 MNE 10 35.993 0.000 2166

15 97 MNE 20 34.799 0.000 825

15 97 MNE 30 31.271 0.000 343

15 97 MNE 40 27.932 0.000 142

15 97 MNE 50 24.123 0.000 69

15 97 MNE 60 15.298 0.000 30

15 97 MNE 70 8.046 0.000 13

15 97 MNE 80 0.000 0.000 6

15 97 MNE 90 0.000 0.000 3

15 97 MNE 100 0.000 0.000 1

15 97 sLORETA 0 39.688 0.000 8003

15 97 sLORETA 10 39.400 0.000 4183

15 97 sLORETA 20 38.823 0.000 2409

15 97 sLORETA 30 37.587 0.000 1326

15 97 sLORETA 40 33.876 0.000 655

15 97 sLORETA 50 29.058 0.000 264

15 97 sLORETA 60 20.258 0.000 89

15 97 sLORETA 70 13.006 0.000 25

15 97 sLORETA 80 2.338 0.000 10

15 97 sLORETA 90 0.000 0.000 4

15 97 sLORETA 100 0.000 0.000 1

15 98 Ave 0 37.668 0.000 8003

15 98 Ave 10 37.184 0.000 3273

15 98 Ave 20 35.249 0.000 1447

15 98 Ave 30 29.410 0.000 609

15 98 Ave 40 17.574 0.000 252

15 98 Ave 50 0.753 0.000 109

15 98 Ave 60 0.000 0.000 38

15 98 Ave 70 0.000 0.000 20

15 98 Ave 80 0.000 0.000 11

15 98 Ave 90 0.000 0.000 6

15 98 Ave 100 0.000 0.000 1

15 98 cMEM 0 13.960 0.000 8003

15 98 cMEM 10 12.732 0.000 641

15 98 cMEM 20 12.243 0.000 313

15 98 cMEM 30 12.718 0.000 156

15 98 cMEM 40 12.414 0.000 62

15 98 cMEM 50 12.722 0.000 31

15 98 cMEM 60 11.481 0.000 19

15 98 cMEM 70 7.591 0.000 9

15 98 cMEM 80 0.000 0.000 4

15 98 cMEM 90 0.000 0.000 3

15 98 cMEM 100 0.000 0.000 1

15 98 dSPM 0 40.310 0.000 8003

15 98 dSPM 10 40.123 0.000 3774

15 98 dSPM 20 39.742 0.000 1987

15 98 dSPM 30 38.062 0.000 1017

15 98 dSPM 40 33.755 0.000 499

15 98 dSPM 50 27.660 0.000 247

15 98 dSPM 60 17.722 0.000 120

15 98 dSPM 70 3.901 0.000 61

15 98 dSPM 80 3.519 0.000 25

15 98 dSPM 90 1.762 0.000 8

15 98 dSPM 100 0.000 0.000 1

15 98 MNE 0 35.941 0.000 8003

15 98 MNE 10 34.469 0.000 1221

15 98 MNE 20 30.147 0.000 378

15 98 MNE 30 25.220 0.000 148

15 98 MNE 40 17.733 0.000 63

15 98 MNE 50 0.000 0.000 35

15 98 MNE 60 0.000 0.000 18

15 98 MNE 70 0.000 0.000 15

15 98 MNE 80 0.000 0.000 6

15 98 MNE 90 0.000 0.000 3

15 98 MNE 100 0.000 0.000 1

15 98 sLORETA 0 38.845 0.000 8003

15 98 sLORETA 10 38.401 0.000 3227

15 98 sLORETA 20 36.935 0.000 1459

15 98 sLORETA 30 31.914 0.000 594

15 98 sLORETA 40 20.202 0.000 223

15 98 sLORETA 50 1.494 0.000 87

15 98 sLORETA 60 1.211 0.000 34

15 98 sLORETA 70 0.000 0.000 19

15 98 sLORETA 80 0.000 0.000 12

15 98 sLORETA 90 0.000 0.000 6

15 98 sLORETA 100 0.000 0.000 1

15 99 Ave 0 37.125 0.000 8003

15 99 Ave 10 35.671 0.000 2045

15 99 Ave 20 26.720 0.000 523

15 99 Ave 30 9.811 0.000 189

15 99 Ave 40 0.000 0.000 76

15 99 Ave 50 0.000 0.000 39

15 99 Ave 60 0.000 0.000 26

15 99 Ave 70 0.000 0.000 15

15 99 Ave 80 0.000 0.000 10

15 99 Ave 90 0.000 0.000 2

15 99 Ave 100 0.000 0.000 1

15 99 cMEM 0 7.107 0.000 8003

15 99 cMEM 10 3.514 0.000 143

15 99 cMEM 20 0.000 0.000 79

15 99 cMEM 30 0.000 0.000 59

15 99 cMEM 40 0.000 0.000 43

15 99 cMEM 50 0.000 0.000 29

15 99 cMEM 60 0.000 0.000 14

15 99 cMEM 70 0.000 0.000 7

15 99 cMEM 80 0.000 0.000 4

15 99 cMEM 90 0.000 0.000 1

15 99 cMEM 100 0.000 0.000 1

15 99 dSPM 0 42.201 0.000 8003

15 99 dSPM 10 42.020 0.000 3210

15 99 dSPM 20 41.118 0.000 1391

15 99 dSPM 30 36.846 0.000 582

15 99 dSPM 40 30.357 0.000 260

15 99 dSPM 50 16.676 0.000 116

15 99 dSPM 60 0.644 0.000 41

15 99 dSPM 70 0.000 0.000 13

15 99 dSPM 80 0.000 0.000 8

15 99 dSPM 90 0.000 0.000 4

15 99 dSPM 100 0.000 0.000 1

15 99 MNE 0 32.015 0.000 8003

15 99 MNE 10 27.277 0.000 743

15 99 MNE 20 17.512 0.000 221

15 99 MNE 30 6.590 0.000 82

15 99 MNE 40 0.000 0.000 41

15 99 MNE 50 0.000 0.000 25

15 99 MNE 60 0.000 0.000 18

15 99 MNE 70 0.000 0.000 9

15 99 MNE 80 0.000 0.000 6

15 99 MNE 90 0.000 0.000 3

15 99 MNE 100 0.000 0.000 1

15 99 sLORETA 0 38.637 0.000 8003

15 99 sLORETA 10 37.917 0.000 2765

15 99 sLORETA 20 34.988 0.000 1071

15 99 sLORETA 30 23.897 0.000 364

15 99 sLORETA 40 11.204 0.000 166

15 99 sLORETA 50 1.062 0.000 78

15 99 sLORETA 60 0.334 0.000 39

15 99 sLORETA 70 0.000 0.000 24

15 99 sLORETA 80 0.000 0.000 14

15 99 sLORETA 90 0.000 0.000 6

15 99 sLORETA 100 0.000 0.000 1

15 100 Ave 0 43.775 0.000 8003

15 100 Ave 10 43.655 0.000 4251

15 100 Ave 20 43.606 0.000 2442

15 100 Ave 30 42.904 0.000 1424

15 100 Ave 40 41.073 0.000 715

15 100 Ave 50 37.645 0.000 320

15 100 Ave 60 23.969 0.000 120

15 100 Ave 70 11.861 0.000 42

15 100 Ave 80 0.000 0.000 17

15 100 Ave 90 0.000 0.000 5

15 100 Ave 100 0.000 0.000 1

15 100 cMEM 0 14.416 0.000 8003

15 100 cMEM 10 9.623 0.000 496

15 100 cMEM 20 8.866 0.000 168

15 100 cMEM 30 6.348 0.000 64

15 100 cMEM 40 0.000 0.000 36

15 100 cMEM 50 0.000 0.000 21

15 100 cMEM 60 0.000 0.000 7

15 100 cMEM 70 0.000 0.000 3

15 100 cMEM 80 0.000 0.000 2

15 100 cMEM 90 0.000 0.000 1

15 100 cMEM 100 0.000 0.000 1

15 100 dSPM 0 45.795 0.000 8003

15 100 dSPM 10 45.810 0.000 3885

15 100 dSPM 20 46.089 0.000 2200

15 100 dSPM 30 46.366 0.000 1124

15 100 dSPM 40 45.184 0.000 600

15 100 dSPM 50 43.433 0.000 276

15 100 dSPM 60 37.516 0.000 102

15 100 dSPM 70 13.290 0.000 42

15 100 dSPM 80 4.998 0.000 15

15 100 dSPM 90 6.297 0.000 5

15 100 dSPM 100 0.000 0.000 1

15 100 MNE 0 40.048 0.000 8003

15 100 MNE 10 38.993 0.000 1615

15 100 MNE 20 36.206 0.000 525

15 100 MNE 30 32.272 0.000 208

15 100 MNE 40 25.082 0.000 81

15 100 MNE 50 16.336 0.000 29

15 100 MNE 60 19.540 0.000 15

15 100 MNE 70 0.000 0.000 8

15 100 MNE 80 0.000 0.000 4

15 100 MNE 90 0.000 0.000 3

15 100 MNE 100 0.000 0.000 1

15 100 sLORETA 0 44.566 0.000 8003

15 100 sLORETA 10 44.484 0.000 4612

15 100 sLORETA 20 44.484 0.000 2773

15 100 sLORETA 30 44.501 0.000 1748

15 100 sLORETA 40 43.764 0.000 1030

15 100 sLORETA 50 42.202 0.000 526

15 100 sLORETA 60 38.863 0.000 249

15 100 sLORETA 70 35.989 0.000 86

15 100 sLORETA 80 22.987 0.000 29

15 100 sLORETA 90 0.000 0.000 8

15 100 sLORETA 100 0.000 0.000 1

15 101 Ave 0 33.894 0.000 8003

15 101 Ave 10 32.801 0.000 3030

15 101 Ave 20 28.370 0.000 1164

15 101 Ave 30 16.790 0.000 403

15 101 Ave 40 3.393 0.000 186

15 101 Ave 50 1.310 0.000 79

15 101 Ave 60 0.316 0.000 46

15 101 Ave 70 0.000 0.000 28

15 101 Ave 80 0.000 0.000 14

15 101 Ave 90 0.000 0.000 6

15 101 Ave 100 0.000 0.000 1

15 101 cMEM 0 20.593 0.000 8003

15 101 cMEM 10 20.712 0.000 473

15 101 cMEM 20 21.572 0.000 236

15 101 cMEM 30 22.504 0.000 144

15 101 cMEM 40 23.536 0.000 90

15 101 cMEM 50 25.309 0.000 55

15 101 cMEM 60 26.846 0.000 26

15 101 cMEM 70 31.922 0.000 13

15 101 cMEM 80 35.500 31.201 6

15 101 cMEM 90 34.497 34.111 2

15 101 cMEM 100 34.821 34.821 1

15 101 dSPM 0 38.249 0.000 8003

15 101 dSPM 10 37.821 0.000 3417

15 101 dSPM 20 36.439 0.000 1677

15 101 dSPM 30 32.766 0.000 709

15 101 dSPM 40 29.115 0.000 358

15 101 dSPM 50 19.248 0.000 152

15 101 dSPM 60 2.976 0.000 55

15 101 dSPM 70 1.456 0.000 20

15 101 dSPM 80 0.000 0.000 4

15 101 dSPM 90 0.000 0.000 2

15 101 dSPM 100 0.000 0.000 1

15 101 MNE 0 29.126 0.000 8003

15 101 MNE 10 25.027 0.000 889

15 101 MNE 20 17.781 0.000 267

15 101 MNE 30 8.551 0.000 106

15 101 MNE 40 0.000 0.000 54

15 101 MNE 50 0.000 0.000 31

15 101 MNE 60 0.000 0.000 18

15 101 MNE 70 0.000 0.000 9

15 101 MNE 80 0.000 0.000 6

15 101 MNE 90 0.000 0.000 3

15 101 MNE 100 0.000 0.000 1

15 101 sLORETA 0 34.246 0.000 8003

15 101 sLORETA 10 33.188 0.000 3080

15 101 sLORETA 20 29.603 0.000 1235

15 101 sLORETA 30 18.500 0.000 431

15 101 sLORETA 40 3.410 0.000 207

15 101 sLORETA 50 2.539 0.000 105

15 101 sLORETA 60 1.755 0.000 56

15 101 sLORETA 70 1.032 0.000 33

15 101 sLORETA 80 0.644 0.000 15

15 101 sLORETA 90 0.000 0.000 5

15 101 sLORETA 100 0.000 0.000 1

16 102 Ave 0 46.674 0.000 8002

16 102 Ave 10 46.142 0.000 4209

16 102 Ave 20 43.312 0.000 1826

16 102 Ave 30 37.572 0.000 785

16 102 Ave 40 30.163 0.000 331

16 102 Ave 50 23.285 0.000 172

16 102 Ave 60 15.744 0.000 109

16 102 Ave 70 15.459 0.000 71

16 102 Ave 80 16.020 6.162 35

16 102 Ave 90 16.485 9.400 10

16 102 Ave 100 16.880 16.880 1

16 102 cMEM 0 31.333 0.000 8002

16 102 cMEM 10 30.525 0.000 389

16 102 cMEM 20 29.487 0.000 254

16 102 cMEM 30 29.648 0.000 183

16 102 cMEM 40 29.087 0.000 136

16 102 cMEM 50 27.316 0.000 84

16 102 cMEM 60 25.867 4.325 53

16 102 cMEM 70 13.392 4.325 19

16 102 cMEM 80 12.337 7.916 7

16 102 cMEM 90 9.780 8.683 2

16 102 cMEM 100 8.683 8.683 1

16 102 dSPM 0 38.998 0.000 8002

16 102 dSPM 10 35.861 0.000 2365

16 102 dSPM 20 28.923 0.000 802

16 102 dSPM 30 22.076 0.000 295

16 102 dSPM 40 19.955 0.000 136

16 102 dSPM 50 19.145 0.000 67

16 102 dSPM 60 18.737 0.000 37

16 102 dSPM 70 18.361 9.400 19

16 102 dSPM 80 16.408 9.400 10

16 102 dSPM 90 17.506 9.400 3

16 102 dSPM 100 9.400 9.400 1

16 102 MNE 0 54.929 0.000 8002

16 102 MNE 10 54.871 0.000 3846

16 102 MNE 20 54.548 0.000 1680

16 102 MNE 30 52.962 0.000 770

16 102 MNE 40 51.484 0.000 380

16 102 MNE 50 51.312 0.000 173

16 102 MNE 60 49.689 0.000 79

16 102 MNE 70 51.162 0.000 49

16 102 MNE 80 49.913 0.000 19

16 102 MNE 90 11.574 0.000 3

16 102 MNE 100 6.162 6.162 1

16 102 sLORETA 0 49.971 0.000 8002

16 102 sLORETA 10 49.758 0.000 4883

16 102 sLORETA 20 48.557 0.000 2537

16 102 sLORETA 30 45.965 0.000 1304

16 102 sLORETA 40 42.486 0.000 644

16 102 sLORETA 50 39.709 0.000 310

16 102 sLORETA 60 36.872 0.000 160

16 102 sLORETA 70 33.707 0.000 72

16 102 sLORETA 80 25.626 0.000 24

16 102 sLORETA 90 16.058 9.400 7

16 102 sLORETA 100 9.400 9.400 1

16 103 Ave 0 50.757 0.000 8002

16 103 Ave 10 50.465 0.000 4301

16 103 Ave 20 48.853 0.000 2018

16 103 Ave 30 44.972 0.000 895

16 103 Ave 40 39.672 0.000 363

16 103 Ave 50 32.312 0.000 168

16 103 Ave 60 17.939 0.000 75

16 103 Ave 70 12.179 0.000 35

16 103 Ave 80 11.489 2.925 21

16 103 Ave 90 10.908 6.162 8

16 103 Ave 100 11.453 11.453 1

16 103 cMEM 0 14.357 0.000 8002

16 103 cMEM 10 12.548 0.000 199

16 103 cMEM 20 11.637 0.000 106

16 103 cMEM 30 11.205 0.000 67

16 103 cMEM 40 10.042 0.000 40

16 103 cMEM 50 9.034 2.561 22

16 103 cMEM 60 8.852 2.925 15

16 103 cMEM 70 8.363 3.422 9

16 103 cMEM 80 8.057 6.002 5

16 103 cMEM 90 8.014 6.162 2

16 103 cMEM 100 6.162 6.162 1

16 103 dSPM 0 42.147 0.000 8002

16 103 dSPM 10 40.632 0.000 2983

16 103 dSPM 20 35.668 0.000 1185

16 103 dSPM 30 27.674 0.000 474

16 103 dSPM 40 20.565 0.000 222

16 103 dSPM 50 17.972 0.000 120

16 103 dSPM 60 16.189 0.000 59

16 103 dSPM 70 15.476 0.000 32

16 103 dSPM 80 16.567 7.853 16

16 103 dSPM 90 15.356 9.400 7

16 103 dSPM 100 17.689 17.689 1

16 103 MNE 0 58.454 0.000 8002

16 103 MNE 10 58.555 0.000 4056

16 103 MNE 20 59.058 0.000 1826

16 103 MNE 30 59.237 0.000 802

16 103 MNE 40 58.803 0.000 409

16 103 MNE 50 57.827 0.000 215

16 103 MNE 60 58.920 0.000 109

16 103 MNE 70 62.215 0.000 58

16 103 MNE 80 67.876 0.000 23

16 103 MNE 90 75.286 2.925 10

16 103 MNE 100 94.080 94.080 1

16 103 sLORETA 0 53.089 0.000 8002

16 103 sLORETA 10 53.026 0.000 5108

16 103 sLORETA 20 52.619 0.000 2952

16 103 sLORETA 30 51.402 0.000 1718

16 103 sLORETA 40 49.866 0.000 968

16 103 sLORETA 50 47.479 0.000 504

16 103 sLORETA 60 45.451 0.000 248

16 103 sLORETA 70 44.046 0.000 112

16 103 sLORETA 80 42.292 0.000 47

16 103 sLORETA 90 37.320 0.000 12

16 103 sLORETA 100 9.977 9.977 1

17 104 Ave 0 43.757 0.000 8002

17 104 Ave 10 42.428 0.000 3390

17 104 Ave 20 36.755 0.000 1303

17 104 Ave 30 28.993 0.000 540

17 104 Ave 40 13.925 0.000 176

17 104 Ave 50 7.896 0.000 80

17 104 Ave 60 5.046 0.000 47

17 104 Ave 70 1.987 0.000 20

17 104 Ave 80 0.000 0.000 10

17 104 Ave 90 0.000 0.000 5

17 104 Ave 100 0.000 0.000 1

17 104 cMEM 0 11.430 0.000 8002

17 104 cMEM 10 9.942 0.000 239

17 104 cMEM 20 6.076 0.000 100

17 104 cMEM 30 6.101 0.000 76

17 104 cMEM 40 5.933 0.000 60

17 104 cMEM 50 6.066 0.000 47

17 104 cMEM 60 5.920 0.000 38

17 104 cMEM 70 5.814 0.000 27

17 104 cMEM 80 5.941 0.000 16

17 104 cMEM 90 5.103 0.000 9

17 104 cMEM 100 0.000 0.000 1

17 104 dSPM 0 40.825 0.000 8002

17 104 dSPM 10 39.636 0.000 3583

17 104 dSPM 20 35.263 0.000 1545

17 104 dSPM 30 30.516 0.000 761

17 104 dSPM 40 25.814 0.000 373

17 104 dSPM 50 22.906 0.000 201

17 104 dSPM 60 18.283 0.000 111

17 104 dSPM 70 13.927 0.000 51

17 104 dSPM 80 10.684 0.000 19

17 104 dSPM 90 7.002 0.000 4

17 104 dSPM 100 0.000 0.000 1

17 104 MNE 0 49.363 0.000 8002

17 104 MNE 10 48.547 0.000 2751

17 104 MNE 20 45.216 0.000 963

17 104 MNE 30 41.192 0.000 406

17 104 MNE 40 36.091 0.000 189

17 104 MNE 50 31.701 0.000 87

17 104 MNE 60 20.638 0.000 33

17 104 MNE 70 22.588 0.000 15

17 104 MNE 80 0.000 0.000 7

17 104 MNE 90 0.000 0.000 3

17 104 MNE 100 0.000 0.000 1

17 104 sLORETA 0 46.979 0.000 8002

17 104 sLORETA 10 46.515 0.000 4398

17 104 sLORETA 20 44.097 0.000 2093

17 104 sLORETA 30 40.557 0.000 1058

17 104 sLORETA 40 36.464 0.000 545

17 104 sLORETA 50 30.981 0.000 221

17 104 sLORETA 60 12.356 0.000 71

17 104 sLORETA 70 2.219 0.000 36

17 104 sLORETA 80 0.000 0.000 14

17 104 sLORETA 90 0.000 0.000 6

17 104 sLORETA 100 0.000 0.000 1

18 105 Ave 0 30.026 0.000 8002

18 105 Ave 10 22.393 0.000 1538

18 105 Ave 20 14.603 0.000 505

18 105 Ave 30 12.800 0.000 262

18 105 Ave 40 10.047 0.000 143

18 105 Ave 50 7.147 0.000 77

18 105 Ave 60 5.631 0.000 36

18 105 Ave 70 5.857 0.000 19

18 105 Ave 80 5.149 0.000 8

18 105 Ave 90 5.239 4.041 2

18 105 Ave 100 4.041 4.041 1

18 105 cMEM 0 7.853 0.000 8002

18 105 cMEM 10 7.092 0.000 203

18 105 cMEM 20 6.877 0.000 127

18 105 cMEM 30 6.848 0.000 94

18 105 cMEM 40 6.992 0.000 47

18 105 cMEM 50 7.177 0.000 35

18 105 cMEM 60 7.204 0.000 16

18 105 cMEM 70 7.633 0.000 12

18 105 cMEM 80 8.104 4.545 6

18 105 cMEM 90 8.503 5.543 2

18 105 cMEM 100 10.640 10.640 1

18 105 dSPM 0 34.872 0.000 8002

18 105 dSPM 10 31.280 0.000 2782

18 105 dSPM 20 24.004 0.000 1089

18 105 dSPM 30 17.968 0.000 513

18 105 dSPM 40 15.592 0.000 277

18 105 dSPM 50 13.248 0.000 149

18 105 dSPM 60 10.103 0.000 72

18 105 dSPM 70 8.168 0.000 38

18 105 dSPM 80 7.509 0.000 17

18 105 dSPM 90 6.856 0.000 7

18 105 dSPM 100 0.000 0.000 1

18 105 MNE 0 30.957 0.000 8002

18 105 MNE 10 25.575 0.000 1264

18 105 MNE 20 19.243 0.000 473

18 105 MNE 30 16.145 0.000 252

18 105 MNE 40 15.641 0.000 145

18 105 MNE 50 14.670 0.000 88

18 105 MNE 60 13.624 0.000 48

18 105 MNE 70 12.334 0.000 29

18 105 MNE 80 12.435 0.000 12

18 105 MNE 90 19.655 0.000 4

18 105 MNE 100 4.332 4.332 1

18 105 sLORETA 0 30.119 0.000 8002

18 105 sLORETA 10 21.712 0.000 1338

18 105 sLORETA 20 14.537 0.000 457

18 105 sLORETA 30 12.211 0.000 230

18 105 sLORETA 40 7.331 0.000 100

18 105 sLORETA 50 5.945 0.000 41

18 105 sLORETA 60 4.938 0.000 22

18 105 sLORETA 70 4.775 0.000 11

18 105 sLORETA 80 3.948 0.000 5

18 105 sLORETA 90 0.000 0.000 2

18 105 sLORETA 100 0.000 0.000 1

18 106 Ave 0 23.470 0.000 8002

18 106 Ave 10 15.741 0.000 1089

18 106 Ave 20 11.364 0.000 436

18 106 Ave 30 9.366 0.000 247

18 106 Ave 40 7.369 0.000 147

18 106 Ave 50 5.992 0.000 81

18 106 Ave 60 5.470 0.000 43

18 106 Ave 70 5.864 0.000 21

18 106 Ave 80 5.052 0.000 9

18 106 Ave 90 5.287 4.041 2

18 106 Ave 100 6.290 6.290 1

18 106 cMEM 0 7.761 0.000 8002

18 106 cMEM 10 7.259 0.000 128

18 106 cMEM 20 7.241 0.000 76

18 106 cMEM 30 7.338 0.000 48

18 106 cMEM 40 7.219 0.000 30

18 106 cMEM 50 7.605 1.334 19

18 106 cMEM 60 8.089 3.445 11

18 106 cMEM 70 8.448 4.828 5

18 106 cMEM 80 9.429 7.904 2

18 106 cMEM 90 9.429 7.904 2

18 106 cMEM 100 10.640 10.640 1

18 106 dSPM 0 26.667 0.000 8002

18 106 dSPM 10 20.119 0.000 1463

18 106 dSPM 20 14.609 0.000 567

18 106 dSPM 30 11.211 0.000 262

18 106 dSPM 40 8.743 0.000 140

18 106 dSPM 50 8.646 0.000 72

18 106 dSPM 60 8.711 0.000 35

18 106 dSPM 70 8.597 0.000 16

18 106 dSPM 80 9.035 0.000 11

18 106 dSPM 90 9.917 6.290 5

18 106 dSPM 100 12.435 12.435 1

18 106 MNE 0 23.347 0.000 8002

18 106 MNE 10 16.938 0.000 798

18 106 MNE 20 12.644 0.000 357

18 106 MNE 30 10.901 0.000 211

18 106 MNE 40 9.613 0.000 121

18 106 MNE 50 7.431 0.000 74

18 106 MNE 60 7.594 0.000 36

18 106 MNE 70 6.406 0.000 24

18 106 MNE 80 3.802 0.000 13

18 106 MNE 90 3.496 0.000 5

18 106 MNE 100 0.000 0.000 1

18 106 sLORETA 0 24.534 0.000 8002

18 106 sLORETA 10 17.429 0.000 1182

18 106 sLORETA 20 11.657 0.000 445

18 106 sLORETA 30 9.195 0.000 256

18 106 sLORETA 40 6.737 0.000 138

18 106 sLORETA 50 5.830 0.000 68

18 106 sLORETA 60 5.306 0.000 29

18 106 sLORETA 70 4.797 0.000 16

18 106 sLORETA 80 3.322 0.000 8

18 106 sLORETA 90 0.000 0.000 2

18 106 sLORETA 100 0.000 0.000 1

18 107 Ave 0 21.792 0.000 8002

18 107 Ave 10 14.243 0.000 925

18 107 Ave 20 11.410 0.000 421

18 107 Ave 30 9.254 0.000 240

18 107 Ave 40 6.888 0.000 127

18 107 Ave 50 6.546 0.000 72

18 107 Ave 60 6.046 0.000 36

18 107 Ave 70 6.199 0.000 19

18 107 Ave 80 5.991 0.000 9

18 107 Ave 90 6.184 4.041 3

18 107 Ave 100 6.290 6.290 1

18 107 cMEM 0 8.775 0.000 8002

18 107 cMEM 10 8.180 0.000 148

18 107 cMEM 20 8.266 0.000 63

18 107 cMEM 30 8.323 1.334 40

18 107 cMEM 40 8.282 1.334 29

18 107 cMEM 50 8.345 3.445 19

18 107 cMEM 60 8.445 3.445 12

18 107 cMEM 70 9.486 7.756 6

18 107 cMEM 80 9.450 7.904 2

18 107 cMEM 90 9.450 7.904 2

18 107 cMEM 100 10.640 10.640 1

18 107 dSPM 0 25.138 0.000 8002

18 107 dSPM 10 18.404 0.000 1324

18 107 dSPM 20 14.298 0.000 560

18 107 dSPM 30 11.672 0.000 281

18 107 dSPM 40 8.854 0.000 139

18 107 dSPM 50 8.931 0.000 81

18 107 dSPM 60 9.526 0.000 37

18 107 dSPM 70 8.845 0.000 20

18 107 dSPM 80 9.480 0.000 12

18 107 dSPM 90 9.597 6.290 6

18 107 dSPM 100 12.435 12.435 1

18 107 MNE 0 21.589 0.000 8002

18 107 MNE 10 15.365 0.000 711

18 107 MNE 20 12.478 0.000 343

18 107 MNE 30 11.266 0.000 201

18 107 MNE 40 9.811 0.000 119

18 107 MNE 50 8.700 0.000 66

18 107 MNE 60 7.851 0.000 33

18 107 MNE 70 3.520 0.000 20

18 107 MNE 80 3.888 0.000 11

18 107 MNE 90 3.942 0.000 4

18 107 MNE 100 5.180 5.180 1

18 107 sLORETA 0 22.998 0.000 8002

18 107 sLORETA 10 16.176 0.000 1072

18 107 sLORETA 20 12.051 0.000 460

18 107 sLORETA 30 9.720 0.000 268

18 107 sLORETA 40 6.969 0.000 137

18 107 sLORETA 50 6.326 0.000 66

18 107 sLORETA 60 5.259 0.000 31

18 107 sLORETA 70 5.310 0.000 19

18 107 sLORETA 80 4.853 0.000 9

18 107 sLORETA 90 3.451 0.000 4

18 107 sLORETA 100 0.000 0.000 1

18 108 Ave 0 21.371 0.000 8002

18 108 Ave 10 13.693 0.000 895

18 108 Ave 20 10.547 0.000 428

18 108 Ave 30 8.044 0.000 228

18 108 Ave 40 6.585 0.000 132

18 108 Ave 50 6.372 0.000 74

18 108 Ave 60 5.901 0.000 37

18 108 Ave 70 6.131 0.000 20

18 108 Ave 80 5.473 3.445 8

18 108 Ave 90 5.003 4.041 3

18 108 Ave 100 4.041 4.041 1

18 108 cMEM 0 7.878 0.000 8002

18 108 cMEM 10 7.424 0.000 225

18 108 cMEM 20 7.308 0.000 116

18 108 cMEM 30 7.376 0.000 79

18 108 cMEM 40 7.373 0.000 43

18 108 cMEM 50 7.827 0.000 27

18 108 cMEM 60 7.676 3.445 16

18 108 cMEM 70 7.706 3.445 14

18 108 cMEM 80 9.140 7.756 4

18 108 cMEM 90 8.850 7.756 3

18 108 cMEM 100 7.756 7.756 1

18 108 dSPM 0 24.962 0.000 8002

18 108 dSPM 10 18.031 0.000 1245

18 108 dSPM 20 13.932 0.000 567

18 108 dSPM 30 10.731 0.000 298

18 108 dSPM 40 8.402 0.000 143

18 108 dSPM 50 8.057 0.000 80

18 108 dSPM 60 8.580 0.000 39

18 108 dSPM 70 8.638 0.000 18

18 108 dSPM 80 8.764 0.000 12

18 108 dSPM 90 9.483 6.290 5

18 108 dSPM 100 12.435 12.435 1

18 108 MNE 0 21.603 0.000 8002

18 108 MNE 10 15.626 0.000 772

18 108 MNE 20 11.883 0.000 355

18 108 MNE 30 10.080 0.000 202

18 108 MNE 40 8.981 0.000 116

18 108 MNE 50 7.223 0.000 68

18 108 MNE 60 7.535 0.000 35

18 108 MNE 70 3.412 0.000 17

18 108 MNE 80 3.638 0.000 12

18 108 MNE 90 2.440 0.000 3

18 108 MNE 100 0.000 0.000 1

18 108 sLORETA 0 22.821 0.000 8002

18 108 sLORETA 10 15.707 0.000 996

18 108 sLORETA 20 11.497 0.000 466

18 108 sLORETA 30 8.894 0.000 254

18 108 sLORETA 40 6.593 0.000 147

18 108 sLORETA 50 5.752 0.000 59

18 108 sLORETA 60 4.931 0.000 28

18 108 sLORETA 70 4.665 0.000 18

18 108 sLORETA 80 3.755 0.000 8

18 108 sLORETA 90 3.252 0.000 3

18 108 sLORETA 100 0.000 0.000 1

18 109 Ave 0 24.528 0.000 8002

18 109 Ave 10 15.895 0.000 1033

18 109 Ave 20 11.181 0.000 450

18 109 Ave 30 8.804 0.000 241

18 109 Ave 40 6.785 0.000 144

18 109 Ave 50 5.976 0.000 76

18 109 Ave 60 5.476 0.000 41

18 109 Ave 70 5.415 0.000 20

18 109 Ave 80 5.716 0.000 10

18 109 Ave 90 5.294 4.041 2

18 109 Ave 100 6.290 6.290 1

18 109 cMEM 0 7.758 0.000 8002

18 109 cMEM 10 7.293 0.000 125

18 109 cMEM 20 7.188 0.000 69

18 109 cMEM 30 7.136 0.000 53

18 109 cMEM 40 7.059 0.000 36

18 109 cMEM 50 7.349 0.000 20

18 109 cMEM 60 7.402 1.334 13

18 109 cMEM 70 7.841 3.445 7

18 109 cMEM 80 7.674 3.445 4

18 109 cMEM 90 9.389 7.904 2

18 109 cMEM 100 10.640 10.640 1

18 109 dSPM 0 27.630 0.000 8002

18 109 dSPM 10 20.389 0.000 1579

18 109 dSPM 20 14.446 0.000 615

18 109 dSPM 30 11.674 0.000 330

18 109 dSPM 40 8.138 0.000 158

18 109 dSPM 50 7.486 0.000 93

18 109 dSPM 60 8.173 0.000 43

18 109 dSPM 70 7.982 0.000 22

18 109 dSPM 80 8.556 0.000 12

18 109 dSPM 90 8.925 0.000 7

18 109 dSPM 100 12.435 12.435 1

18 109 MNE 0 24.985 0.000 8002

18 109 MNE 10 17.406 0.000 780

18 109 MNE 20 12.334 0.000 327

18 109 MNE 30 10.628 0.000 189

18 109 MNE 40 8.426 0.000 109

18 109 MNE 50 7.128 0.000 58

18 109 MNE 60 3.111 0.000 28

18 109 MNE 70 3.301 0.000 17

18 109 MNE 80 2.904 0.000 7

18 109 MNE 90 0.000 0.000 1

18 109 MNE 100 0.000 0.000 1

18 109 sLORETA 0 25.654 0.000 8002

18 109 sLORETA 10 18.191 0.000 1146

18 109 sLORETA 20 11.616 0.000 447

18 109 sLORETA 30 9.154 0.000 243

18 109 sLORETA 40 6.283 0.000 133

18 109 sLORETA 50 4.842 0.000 58

18 109 sLORETA 60 4.743 0.000 25

18 109 sLORETA 70 4.464 0.000 14

18 109 sLORETA 80 3.222 0.000 8

18 109 sLORETA 90 0.000 0.000 2

18 109 sLORETA 100 0.000 0.000 1

18 110 Ave 0 22.304 0.000 8002

18 110 Ave 10 14.911 0.000 1048

18 110 Ave 20 10.505 0.000 468

18 110 Ave 30 8.431 0.000 255

18 110 Ave 40 6.976 0.000 152

18 110 Ave 50 6.584 0.000 93

18 110 Ave 60 6.637 0.000 56

18 110 Ave 70 5.953 0.000 29

18 110 Ave 80 6.288 0.000 10

18 110 Ave 90 6.171 4.041 3

18 110 Ave 100 4.041 4.041 1

18 110 cMEM 0 9.064 0.000 8002

18 110 cMEM 10 8.575 0.000 229

18 110 cMEM 20 8.342 0.000 106

18 110 cMEM 30 8.090 0.000 57

18 110 cMEM 40 7.764 0.000 41

18 110 cMEM 50 7.956 0.000 27

18 110 cMEM 60 7.977 0.000 18

18 110 cMEM 70 8.653 0.000 10

18 110 cMEM 80 8.776 0.000 8

18 110 cMEM 90 12.289 10.640 2

18 110 cMEM 100 10.640 10.640 1

18 110 dSPM 0 26.360 0.000 8002

18 110 dSPM 10 19.692 0.000 1408

18 110 dSPM 20 13.975 0.000 565

18 110 dSPM 30 10.640 0.000 274

18 110 dSPM 40 8.669 0.000 145

18 110 dSPM 50 8.816 0.000 73

18 110 dSPM 60 8.763 0.000 38

18 110 dSPM 70 8.672 0.000 18

18 110 dSPM 80 9.026 0.000 11

18 110 dSPM 90 9.839 6.290 4

18 110 dSPM 100 12.435 12.435 1

18 110 MNE 0 22.241 0.000 8002

18 110 MNE 10 15.592 0.000 802

18 110 MNE 20 11.312 0.000 360

18 110 MNE 30 9.792 0.000 215

18 110 MNE 40 7.996 0.000 127

18 110 MNE 50 6.718 0.000 72

18 110 MNE 60 5.603 0.000 38

18 110 MNE 70 3.399 0.000 21

18 110 MNE 80 3.671 0.000 14

18 110 MNE 90 3.478 0.000 5

18 110 MNE 100 0.000 0.000 1

18 110 sLORETA 0 23.995 0.000 8002

18 110 sLORETA 10 17.460 0.000 1159

18 110 sLORETA 20 10.933 0.000 467

18 110 sLORETA 30 8.321 0.000 259

18 110 sLORETA 40 6.535 0.000 145

18 110 sLORETA 50 5.813 0.000 67

18 110 sLORETA 60 5.251 0.000 29

18 110 sLORETA 70 4.684 0.000 18

18 110 sLORETA 80 3.544 0.000 9

18 110 sLORETA 90 0.000 0.000 2

18 110 sLORETA 100 0.000 0.000 1

19 111 Ave 0 32.809 0.000 8002

19 111 Ave 10 27.045 0.000 1767

19 111 Ave 20 23.321 0.000 673

19 111 Ave 30 20.221 0.000 321

19 111 Ave 40 17.731 0.000 163

19 111 Ave 50 16.440 0.000 85

19 111 Ave 60 12.790 0.000 46

19 111 Ave 70 11.150 0.000 25

19 111 Ave 80 9.551 0.000 7

19 111 Ave 90 5.646 5.646 1

19 111 Ave 100 5.646 5.646 1

19 111 cMEM 0 24.630 0.000 8002

19 111 cMEM 10 24.119 0.000 195

19 111 cMEM 20 23.726 0.000 102

19 111 cMEM 30 23.687 0.000 71

19 111 cMEM 40 24.055 0.000 53

19 111 cMEM 50 23.631 4.210 37

19 111 cMEM 60 22.961 5.646 26

19 111 cMEM 70 23.691 10.812 19

19 111 cMEM 80 19.821 10.812 9

19 111 cMEM 90 21.077 19.009 3

19 111 cMEM 100 19.009 19.009 1

19 111 dSPM 0 34.639 0.000 8002

19 111 dSPM 10 31.185 0.000 2142

19 111 dSPM 20 27.575 0.000 888

19 111 dSPM 30 24.994 0.000 487

19 111 dSPM 40 22.137 0.000 263

19 111 dSPM 50 19.647 0.000 151

19 111 dSPM 60 17.934 0.000 92

19 111 dSPM 70 16.732 0.000 53

19 111 dSPM 80 16.937 0.000 26

19 111 dSPM 90 16.976 0.000 12

19 111 dSPM 100 25.414 25.414 1

19 111 MNE 0 32.853 0.000 8002

19 111 MNE 10 25.909 0.000 1146

19 111 MNE 20 21.870 0.000 406

19 111 MNE 30 19.385 0.000 203

19 111 MNE 40 16.220 0.000 90

19 111 MNE 50 14.124 0.000 43

19 111 MNE 60 9.480 0.000 18

19 111 MNE 70 6.714 0.000 6

19 111 MNE 80 4.855 0.000 4

19 111 MNE 90 4.011 0.000 2

19 111 MNE 100 5.646 5.646 1

19 111 sLORETA 0 34.484 0.000 8002

19 111 sLORETA 10 30.039 0.000 2455

19 111 sLORETA 20 25.411 0.000 1023

19 111 sLORETA 30 22.265 0.000 475

19 111 sLORETA 40 18.299 0.000 249

19 111 sLORETA 50 15.762 0.000 128

19 111 sLORETA 60 12.632 0.000 60

19 111 sLORETA 70 9.908 0.000 34

19 111 sLORETA 80 8.226 0.000 17

19 111 sLORETA 90 6.867 0.000 5

19 111 sLORETA 100 5.646 5.646 1

19 112 Ave 0 32.541 0.000 8002

19 112 Ave 10 27.099 0.000 1823

19 112 Ave 20 23.300 0.000 699

19 112 Ave 30 20.236 0.000 337

19 112 Ave 40 18.160 0.000 182

19 112 Ave 50 17.362 0.000 106

19 112 Ave 60 14.884 0.000 50

19 112 Ave 70 12.809 0.000 27

19 112 Ave 80 9.436 0.000 9

19 112 Ave 90 5.646 5.646 1

19 112 Ave 100 5.646 5.646 1

19 112 cMEM 0 27.886 0.000 8002

19 112 cMEM 10 27.142 0.000 307

19 112 cMEM 20 26.973 0.000 158

19 112 cMEM 30 27.478 0.000 90

19 112 cMEM 40 28.250 3.530 62

19 112 cMEM 50 29.584 5.646 40

19 112 cMEM 60 32.226 10.812 18

19 112 cMEM 70 36.071 32.917 9

19 112 cMEM 80 36.424 32.917 6

19 112 cMEM 90 35.602 32.917 4

19 112 cMEM 100 35.891 35.891 1

19 112 dSPM 0 34.300 0.000 8002

19 112 dSPM 10 30.777 0.000 2161

19 112 dSPM 20 27.278 0.000 880

19 112 dSPM 30 24.672 0.000 477

19 112 dSPM 40 22.293 0.000 271

19 112 dSPM 50 19.842 0.000 159

19 112 dSPM 60 18.007 0.000 95

19 112 dSPM 70 16.478 0.000 53

19 112 dSPM 80 16.496 0.000 27

19 112 dSPM 90 16.982 0.000 12

19 112 dSPM 100 26.834 26.834 1

19 112 MNE 0 32.773 0.000 8002

19 112 MNE 10 25.497 0.000 1145

19 112 MNE 20 21.209 0.000 396

19 112 MNE 30 18.327 0.000 195

19 112 MNE 40 15.354 0.000 90

19 112 MNE 50 12.752 0.000 43

19 112 MNE 60 9.809 0.000 19

19 112 MNE 70 7.794 0.000 7

19 112 MNE 80 4.883 0.000 4

19 112 MNE 90 4.014 0.000 2

19 112 MNE 100 5.646 5.646 1

19 112 sLORETA 0 33.919 0.000 8002

19 112 sLORETA 10 29.421 0.000 2444

19 112 sLORETA 20 24.908 0.000 1013

19 112 sLORETA 30 21.187 0.000 454

19 112 sLORETA 40 17.822 0.000 244

19 112 sLORETA 50 15.817 0.000 132

19 112 sLORETA 60 13.202 0.000 61

19 112 sLORETA 70 10.394 0.000 38

19 112 sLORETA 80 8.169 0.000 16

19 112 sLORETA 90 6.916 0.000 5

19 112 sLORETA 100 5.646 5.646 1

19 113 Ave 0 35.294 0.000 8002

19 113 Ave 10 30.435 0.000 2098

19 113 Ave 20 25.763 0.000 916

19 113 Ave 30 21.141 0.000 400

19 113 Ave 40 14.957 0.000 178

19 113 Ave 50 12.473 0.000 92

19 113 Ave 60 10.770 0.000 46

19 113 Ave 70 9.514 0.000 25

19 113 Ave 80 8.037 2.968 9

19 113 Ave 90 4.191 2.968 3

19 113 Ave 100 3.530 3.530 1

19 113 cMEM 0 33.441 0.000 8002

19 113 cMEM 10 32.176 0.000 785

19 113 cMEM 20 31.271 0.000 402

19 113 cMEM 30 30.311 0.000 231

19 113 cMEM 40 29.048 0.000 149

19 113 cMEM 50 27.621 0.000 90

19 113 cMEM 60 25.430 0.000 59

19 113 cMEM 70 23.894 0.000 34

19 113 cMEM 80 16.356 2.601 12

19 113 cMEM 90 17.344 3.530 3

19 113 cMEM 100 3.530 3.530 1

19 113 dSPM 0 37.461 0.000 8002

19 113 dSPM 10 34.602 0.000 2335

19 113 dSPM 20 30.946 0.000 1069

19 113 dSPM 30 27.273 0.000 529

19 113 dSPM 40 23.853 0.000 275

19 113 dSPM 50 21.457 0.000 151

19 113 dSPM 60 18.848 0.000 82

19 113 dSPM 70 17.386 0.000 42

19 113 dSPM 80 16.301 0.000 24

19 113 dSPM 90 15.354 0.000 10

19 113 dSPM 100 11.425 11.425 1

19 113 MNE 0 35.352 0.000 8002

19 113 MNE 10 29.018 0.000 1581

19 113 MNE 20 23.674 0.000 618

19 113 MNE 30 18.770 0.000 253

19 113 MNE 40 13.399 0.000 114

19 113 MNE 50 11.070 0.000 64

19 113 MNE 60 10.202 0.000 33

19 113 MNE 70 9.895 0.000 13

19 113 MNE 80 4.978 0.000 4

19 113 MNE 90 4.069 0.000 2

19 113 MNE 100 5.646 5.646 1

19 113 sLORETA 0 37.506 0.000 8002

19 113 sLORETA 10 34.488 0.000 3075

19 113 sLORETA 20 28.649 0.000 1329

19 113 sLORETA 30 25.103 0.000 720

19 113 sLORETA 40 19.575 0.000 344

19 113 sLORETA 50 15.024 0.000 171

19 113 sLORETA 60 11.075 0.000 78

19 113 sLORETA 70 9.598 0.000 44

19 113 sLORETA 80 10.213 0.000 17

19 113 sLORETA 90 7.230 0.000 9

19 113 sLORETA 100 11.425 11.425 1

19 114 Ave 0 34.580 0.000 8002

19 114 Ave 10 30.083 0.000 2378

19 114 Ave 20 24.555 0.000 942

19 114 Ave 30 20.287 0.000 418

19 114 Ave 40 17.872 0.000 238

19 114 Ave 50 17.001 0.000 139

19 114 Ave 60 15.421 0.000 74

19 114 Ave 70 9.228 0.000 28

19 114 Ave 80 8.278 0.000 12

19 114 Ave 90 4.258 2.968 3

19 114 Ave 100 3.530 3.530 1

19 114 cMEM 0 27.127 0.000 8002

19 114 cMEM 10 26.028 0.000 385

19 114 cMEM 20 25.778 0.000 202

19 114 cMEM 30 25.881 0.000 143

19 114 cMEM 40 26.708 0.000 88

19 114 cMEM 50 27.346 3.530 61

19 114 cMEM 60 27.129 6.790 40

19 114 cMEM 70 27.096 10.812 23

19 114 cMEM 80 25.402 14.176 9

19 114 cMEM 90 21.024 19.009 3

19 114 cMEM 100 19.009 19.009 1

19 114 dSPM 0 36.557 0.000 8002

19 114 dSPM 10 33.554 0.000 2442

19 114 dSPM 20 29.090 0.000 1006

19 114 dSPM 30 25.353 0.000 486

19 114 dSPM 40 22.702 0.000 268

19 114 dSPM 50 20.155 0.000 144

19 114 dSPM 60 18.485 0.000 79

19 114 dSPM 70 17.191 0.000 40

19 114 dSPM 80 16.980 0.000 24

19 114 dSPM 90 17.318 0.000 11

19 114 dSPM 100 11.425 11.425 1

19 114 MNE 0 34.724 0.000 8002

19 114 MNE 10 28.361 0.000 1553

19 114 MNE 20 22.755 0.000 545

19 114 MNE 30 18.514 0.000 233

19 114 MNE 40 13.940 0.000 110

19 114 MNE 50 11.688 0.000 59

19 114 MNE 60 10.534 0.000 31

19 114 MNE 70 9.972 0.000 10

19 114 MNE 80 5.019 0.000 4

19 114 MNE 90 5.071 0.000 3

19 114 MNE 100 5.646 5.646 1

19 114 sLORETA 0 37.134 0.000 8002

19 114 sLORETA 10 34.222 0.000 3173

19 114 sLORETA 20 28.513 0.000 1381

19 114 sLORETA 30 24.579 0.000 714

19 114 sLORETA 40 20.056 0.000 348

19 114 sLORETA 50 13.892 0.000 161

19 114 sLORETA 60 11.047 0.000 80

19 114 sLORETA 70 9.060 0.000 44

19 114 sLORETA 80 9.905 0.000 20

19 114 sLORETA 90 6.708 0.000 10

19 114 sLORETA 100 3.530 3.530 1

19 115 Ave 0 35.152 0.000 8002

19 115 Ave 10 31.234 0.000 2336

19 115 Ave 20 25.680 0.000 1045

19 115 Ave 30 20.809 0.000 524

19 115 Ave 40 16.532 0.000 264

19 115 Ave 50 12.830 0.000 146

19 115 Ave 60 11.102 0.000 78

19 115 Ave 70 9.881 0.000 39

19 115 Ave 80 7.485 0.000 15

19 115 Ave 90 4.242 2.968 3

19 115 Ave 100 3.530 3.530 1

19 115 cMEM 0 22.965 0.000 8002

19 115 cMEM 10 21.879 0.000 492

19 115 cMEM 20 20.684 0.000 232

19 115 cMEM 30 20.095 0.000 131

19 115 cMEM 40 20.132 0.000 80

19 115 cMEM 50 20.303 0.000 55

19 115 cMEM 60 21.537 10.812 30

19 115 cMEM 70 22.759 10.812 18

19 115 cMEM 80 27.261 22.745 4

19 115 cMEM 90 28.830 28.830 1

19 115 cMEM 100 28.830 28.830 1

19 115 dSPM 0 38.044 0.000 8002

19 115 dSPM 10 35.563 0.000 2235

19 115 dSPM 20 32.178 0.000 1052

19 115 dSPM 30 28.629 0.000 555

19 115 dSPM 40 25.265 0.000 289

19 115 dSPM 50 22.013 0.000 150

19 115 dSPM 60 19.333 0.000 80

19 115 dSPM 70 17.394 0.000 43

19 115 dSPM 80 16.976 0.000 24

19 115 dSPM 90 16.934 0.000 8

19 115 dSPM 100 2.578 2.578 1

19 115 MNE 0 34.924 0.000 8002

19 115 MNE 10 28.462 0.000 1536

19 115 MNE 20 22.842 0.000 604

19 115 MNE 30 18.680 0.000 264

19 115 MNE 40 14.547 0.000 126

19 115 MNE 50 12.083 0.000 78

19 115 MNE 60 10.308 0.000 38

19 115 MNE 70 9.706 0.000 13

19 115 MNE 80 5.143 0.000 5

19 115 MNE 90 4.086 0.000 2

19 115 MNE 100 5.646 5.646 1

19 115 sLORETA 0 37.198 0.000 8002

19 115 sLORETA 10 34.164 0.000 2920

19 115 sLORETA 20 28.018 0.000 1321

19 115 sLORETA 30 23.690 0.000 685

19 115 sLORETA 40 18.288 0.000 336

19 115 sLORETA 50 13.514 0.000 167

19 115 sLORETA 60 10.448 0.000 83

19 115 sLORETA 70 9.364 0.000 48

19 115 sLORETA 80 9.837 0.000 21

19 115 sLORETA 90 6.377 0.000 8

19 115 sLORETA 100 11.425 11.425 1

19 116 Ave 0 34.440 0.000 8002

19 116 Ave 10 28.929 0.000 2066

19 116 Ave 20 21.707 0.000 750

19 116 Ave 30 16.366 0.000 325

19 116 Ave 40 13.311 0.000 175

19 116 Ave 50 9.400 0.000 79

19 116 Ave 60 8.433 0.000 44

19 116 Ave 70 7.853 0.000 22

19 116 Ave 80 6.995 0.000 12

19 116 Ave 90 4.207 2.968 3

19 116 Ave 100 3.530 3.530 1

19 116 cMEM 0 16.707 0.000 8002

19 116 cMEM 10 15.513 0.000 397

19 116 cMEM 20 14.959 0.000 225

19 116 cMEM 30 14.069 0.000 131

19 116 cMEM 40 12.029 0.000 83

19 116 cMEM 50 10.197 0.000 53

19 116 cMEM 60 9.067 0.000 33

19 116 cMEM 70 9.425 0.000 23

19 116 cMEM 80 8.194 0.000 16

19 116 cMEM 90 7.246 3.530 4

19 116 cMEM 100 3.530 3.530 1

19 116 dSPM 0 37.921 0.000 8002

19 116 dSPM 10 35.257 0.000 2434

19 116 dSPM 20 31.541 0.000 1060

19 116 dSPM 30 26.882 0.000 538

19 116 dSPM 40 23.502 0.000 278

19 116 dSPM 50 20.216 0.000 147

19 116 dSPM 60 17.866 0.000 81

19 116 dSPM 70 16.752 0.000 47

19 116 dSPM 80 16.398 0.000 26

19 116 dSPM 90 17.275 0.000 10

19 116 dSPM 100 11.425 11.425 1

19 116 MNE 0 35.983 0.000 8002

19 116 MNE 10 29.412 0.000 1568

19 116 MNE 20 22.033 0.000 555

19 116 MNE 30 17.961 0.000 235

19 116 MNE 40 14.197 0.000 115

19 116 MNE 50 11.785 0.000 68

19 116 MNE 60 9.489 0.000 31

19 116 MNE 70 10.423 0.000 8

19 116 MNE 80 4.858 0.000 4

19 116 MNE 90 4.002 0.000 2

19 116 MNE 100 5.646 5.646 1

19 116 sLORETA 0 37.528 0.000 8002

19 116 sLORETA 10 34.596 0.000 3131

19 116 sLORETA 20 27.711 0.000 1344

19 116 sLORETA 30 23.424 0.000 678

19 116 sLORETA 40 18.505 0.000 334

19 116 sLORETA 50 13.496 0.000 159

19 116 sLORETA 60 10.001 0.000 80

19 116 sLORETA 70 9.061 0.000 47

19 116 sLORETA 80 8.914 0.000 23

19 116 sLORETA 90 5.458 0.000 8

19 116 sLORETA 100 3.530 3.530 1

19 117 Ave 0 33.592 0.000 8002

19 117 Ave 10 28.963 0.000 2292

19 117 Ave 20 23.834 0.000 974

19 117 Ave 30 19.810 0.000 473

19 117 Ave 40 16.115 0.000 254

19 117 Ave 50 14.020 0.000 141

19 117 Ave 60 10.493 0.000 73

19 117 Ave 70 8.246 0.000 39

19 117 Ave 80 6.801 0.000 16

19 117 Ave 90 7.679 2.968 4

19 117 Ave 100 3.530 3.530 1

19 117 cMEM 0 22.974 0.000 8002

19 117 cMEM 10 22.186 0.000 534

19 117 cMEM 20 22.152 0.000 311

19 117 cMEM 30 21.949 0.000 222

19 117 cMEM 40 22.032 0.000 155

19 117 cMEM 50 22.076 0.000 112

19 117 cMEM 60 21.818 0.000 71

19 117 cMEM 70 23.677 0.000 31

19 117 cMEM 80 20.890 0.000 12

19 117 cMEM 90 28.830 28.830 1

19 117 cMEM 100 28.830 28.830 1

19 117 dSPM 0 36.507 0.000 8002

19 117 dSPM 10 33.695 0.000 2391

19 117 dSPM 20 29.670 0.000 1036

19 117 dSPM 30 26.145 0.000 532

19 117 dSPM 40 22.839 0.000 278

19 117 dSPM 50 20.446 0.000 149

19 117 dSPM 60 18.768 0.000 83

19 117 dSPM 70 16.959 0.000 46

19 117 dSPM 80 16.526 0.000 26

19 117 dSPM 90 17.108 0.000 13

19 117 dSPM 100 11.425 11.425 1

19 117 MNE 0 34.495 0.000 8002

19 117 MNE 10 27.696 0.000 1574

19 117 MNE 20 21.978 0.000 580

19 117 MNE 30 18.328 0.000 250

19 117 MNE 40 12.962 0.000 113

19 117 MNE 50 10.722 0.000 61

19 117 MNE 60 8.392 0.000 36

19 117 MNE 70 6.217 0.000 11

19 117 MNE 80 5.063 0.000 5

19 117 MNE 90 4.024 0.000 2

19 117 MNE 100 5.646 5.646 1

19 117 sLORETA 0 36.337 0.000 8002

19 117 sLORETA 10 33.095 0.000 3049

19 117 sLORETA 20 27.668 0.000 1371

19 117 sLORETA 30 23.746 0.000 701

19 117 sLORETA 40 19.403 0.000 359

19 117 sLORETA 50 13.308 0.000 170

19 117 sLORETA 60 9.695 0.000 81

19 117 sLORETA 70 9.028 0.000 44

19 117 sLORETA 80 8.885 0.000 21

19 117 sLORETA 90 6.255 0.000 9

19 117 sLORETA 100 3.530 3.530 1

19 118 Ave 0 33.432 0.000 8002

19 118 Ave 10 28.175 0.000 2049

19 118 Ave 20 22.597 0.000 801

19 118 Ave 30 17.102 0.000 337

19 118 Ave 40 12.715 0.000 163

19 118 Ave 50 10.049 0.000 82

19 118 Ave 60 8.380 0.000 44

19 118 Ave 70 7.942 0.000 23

19 118 Ave 80 6.976 0.000 12

19 118 Ave 90 4.184 2.968 3

19 118 Ave 100 3.530 3.530 1

19 118 cMEM 0 20.790 0.000 8002

19 118 cMEM 10 18.992 0.000 503

19 118 cMEM 20 17.077 0.000 214

19 118 cMEM 30 16.529 0.000 124

19 118 cMEM 40 15.575 0.000 87

19 118 cMEM 50 13.352 0.000 53

19 118 cMEM 60 10.950 0.000 35

19 118 cMEM 70 8.892 0.000 24

19 118 cMEM 80 7.973 0.000 14

19 118 cMEM 90 7.667 3.530 3

19 118 cMEM 100 3.530 3.530 1

19 118 dSPM 0 36.044 0.000 8002

19 118 dSPM 10 33.277 0.000 2398

19 118 dSPM 20 29.534 0.000 1036

19 118 dSPM 30 26.222 0.000 532

19 118 dSPM 40 22.622 0.000 272

19 118 dSPM 50 20.803 0.000 150

19 118 dSPM 60 18.252 0.000 79

19 118 dSPM 70 16.771 0.000 46

19 118 dSPM 80 17.317 0.000 26

19 118 dSPM 90 17.192 0.000 13

19 118 dSPM 100 11.425 11.425 1

19 118 MNE 0 33.881 0.000 8002

19 118 MNE 10 27.603 0.000 1599

19 118 MNE 20 22.560 0.000 590

19 118 MNE 30 17.875 0.000 245

19 118 MNE 40 13.917 0.000 116

19 118 MNE 50 11.568 0.000 64

19 118 MNE 60 10.181 0.000 32

19 118 MNE 70 5.894 0.000 8

19 118 MNE 80 4.873 0.000 4

19 118 MNE 90 4.017 0.000 2

19 118 MNE 100 5.646 5.646 1

19 118 sLORETA 0 36.254 0.000 8002

19 118 sLORETA 10 33.097 0.000 3015

19 118 sLORETA 20 27.946 0.000 1384

19 118 sLORETA 30 23.112 0.000 683

19 118 sLORETA 40 18.822 0.000 334

19 118 sLORETA 50 13.064 0.000 157

19 118 sLORETA 60 9.748 0.000 79

19 118 sLORETA 70 9.155 0.000 42

19 118 sLORETA 80 9.129 0.000 20

19 118 sLORETA 90 6.271 0.000 9

19 118 sLORETA 100 3.530 3.530 1

19 119 Ave 0 33.582 0.000 8002

19 119 Ave 10 28.998 0.000 2224

19 119 Ave 20 23.940 0.000 941

19 119 Ave 30 19.287 0.000 414

19 119 Ave 40 15.250 0.000 203

19 119 Ave 50 13.417 0.000 109

19 119 Ave 60 12.955 0.000 63

19 119 Ave 70 12.596 0.000 34

19 119 Ave 80 12.406 2.968 12

19 119 Ave 90 3.287 2.968 2

19 119 Ave 100 3.530 3.530 1

19 119 cMEM 0 30.651 0.000 8002

19 119 cMEM 10 28.957 0.000 418

19 119 cMEM 20 27.273 0.000 210

19 119 cMEM 30 25.822 0.000 114

19 119 cMEM 40 24.126 0.000 54

19 119 cMEM 50 24.824 3.530 26

19 119 cMEM 60 25.041 19.401 16

19 119 cMEM 70 25.041 19.401 16

19 119 cMEM 80 24.694 19.558 10

19 119 cMEM 90 25.455 22.624 5

19 119 cMEM 100 28.830 28.830 1

19 119 dSPM 0 35.173 0.000 8002

19 119 dSPM 10 31.852 0.000 2166

19 119 dSPM 20 27.915 0.000 912

19 119 dSPM 30 24.329 0.000 448

19 119 dSPM 40 21.587 0.000 240

19 119 dSPM 50 18.946 0.000 127

19 119 dSPM 60 17.633 0.000 61

19 119 dSPM 70 15.507 0.000 34

19 119 dSPM 80 14.225 0.000 21

19 119 dSPM 90 14.469 0.000 10

19 119 dSPM 100 11.425 11.425 1

19 119 MNE 0 32.879 0.000 8002

19 119 MNE 10 26.987 0.000 1539

19 119 MNE 20 21.261 0.000 588

19 119 MNE 30 17.171 0.000 250

19 119 MNE 40 14.321 0.000 118

19 119 MNE 50 11.806 0.000 66

19 119 MNE 60 11.062 0.000 34

19 119 MNE 70 8.799 0.000 17

19 119 MNE 80 8.562 0.000 6

19 119 MNE 90 4.097 0.000 2

19 119 MNE 100 5.646 5.646 1

19 119 sLORETA 0 36.193 0.000 8002

19 119 sLORETA 10 32.809 0.000 2826

19 119 sLORETA 20 27.342 0.000 1311

19 119 sLORETA 30 22.816 0.000 632

19 119 sLORETA 40 17.893 0.000 300

19 119 sLORETA 50 12.995 0.000 137

19 119 sLORETA 60 10.303 0.000 75

19 119 sLORETA 70 9.606 0.000 42

19 119 sLORETA 80 8.984 0.000 15

19 119 sLORETA 90 7.323 0.000 9

19 119 sLORETA 100 11.425 11.425 1

19 120 Ave 0 32.199 0.000 8002

19 120 Ave 10 26.781 0.000 2031

19 120 Ave 20 21.041 0.000 790

19 120 Ave 30 15.872 0.000 340

19 120 Ave 40 12.162 0.000 169

19 120 Ave 50 10.774 0.000 93

19 120 Ave 60 8.984 0.000 52

19 120 Ave 70 7.884 0.000 27

19 120 Ave 80 7.255 0.000 13

19 120 Ave 90 4.241 2.968 3

19 120 Ave 100 3.530 3.530 1

19 120 cMEM 0 18.190 0.000 8002

19 120 cMEM 10 16.509 0.000 514

19 120 cMEM 20 14.641 0.000 242

19 120 cMEM 30 13.922 0.000 155

19 120 cMEM 40 13.022 0.000 92

19 120 cMEM 50 12.053 0.000 66

19 120 cMEM 60 9.841 0.000 42

19 120 cMEM 70 9.183 0.000 24

19 120 cMEM 80 9.680 3.530 9

19 120 cMEM 90 9.069 6.790 2

19 120 cMEM 100 10.812 10.812 1

19 120 dSPM 0 34.964 0.000 8002

19 120 dSPM 10 31.555 0.000 2174

19 120 dSPM 20 27.090 0.000 898

19 120 dSPM 30 23.561 0.000 442

19 120 dSPM 40 20.393 0.000 219

19 120 dSPM 50 17.843 0.000 117

19 120 dSPM 60 16.848 0.000 63

19 120 dSPM 70 15.228 0.000 34

19 120 dSPM 80 14.846 0.000 20

19 120 dSPM 90 13.907 0.000 10

19 120 dSPM 100 11.425 11.425 1

19 120 MNE 0 32.120 0.000 8002

19 120 MNE 10 25.498 0.000 1456

19 120 MNE 20 20.412 0.000 559

19 120 MNE 30 16.546 0.000 231

19 120 MNE 40 12.369 0.000 112

19 120 MNE 50 10.796 0.000 66

19 120 MNE 60 8.833 0.000 29

19 120 MNE 70 6.006 0.000 14

19 120 MNE 80 4.536 0.000 5

19 120 MNE 90 4.058 0.000 2

19 120 MNE 100 5.646 5.646 1

19 120 sLORETA 0 36.197 0.000 8002

19 120 sLORETA 10 32.938 0.000 2907

19 120 sLORETA 20 27.404 0.000 1327

19 120 sLORETA 30 22.955 0.000 648

19 120 sLORETA 40 18.468 0.000 312

19 120 sLORETA 50 12.505 0.000 143

19 120 sLORETA 60 9.747 0.000 74

19 120 sLORETA 70 9.134 0.000 43

19 120 sLORETA 80 8.141 0.000 15

19 120 sLORETA 90 6.286 0.000 9

19 120 sLORETA 100 11.425 11.425 1

19 121 Ave 0 32.570 0.000 8002

19 121 Ave 10 27.793 0.000 2196

19 121 Ave 20 22.345 0.000 907

19 121 Ave 30 17.674 0.000 398

19 121 Ave 40 14.190 0.000 201

19 121 Ave 50 12.891 0.000 117

19 121 Ave 60 12.624 0.000 78

19 121 Ave 70 12.246 0.000 37

19 121 Ave 80 10.407 0.000 14

19 121 Ave 90 6.517 2.968 4

19 121 Ave 100 3.530 3.530 1

19 121 cMEM 0 22.945 0.000 8002

19 121 cMEM 10 21.663 0.000 455

19 121 cMEM 20 20.953 0.000 201

19 121 cMEM 30 20.751 0.000 101

19 121 cMEM 40 21.438 2.601 65

19 121 cMEM 50 22.124 3.530 45

19 121 cMEM 60 23.252 6.790 27

19 121 cMEM 70 24.555 11.291 17

19 121 cMEM 80 24.316 19.401 13

19 121 cMEM 90 25.122 19.558 7

19 121 cMEM 100 28.830 28.830 1

19 121 dSPM 0 35.229 0.000 8002

19 121 dSPM 10 32.037 0.000 2191

19 121 dSPM 20 27.877 0.000 903

19 121 dSPM 30 24.197 0.000 474

19 121 dSPM 40 21.058 0.000 245

19 121 dSPM 50 18.128 0.000 121

19 121 dSPM 60 16.772 0.000 69

19 121 dSPM 70 15.074 0.000 35

19 121 dSPM 80 14.009 0.000 22

19 121 dSPM 90 13.205 0.000 8

19 121 dSPM 100 2.578 2.578 1

19 121 MNE 0 32.134 0.000 8002

19 121 MNE 10 26.200 0.000 1536

19 121 MNE 20 21.274 0.000 598

19 121 MNE 30 17.160 0.000 259

19 121 MNE 40 14.703 0.000 131

19 121 MNE 50 12.868 0.000 71

19 121 MNE 60 11.418 0.000 45

19 121 MNE 70 9.163 0.000 19

19 121 MNE 80 8.822 0.000 7

19 121 MNE 90 4.251 0.000 3

19 121 MNE 100 0.000 0.000 1

19 121 sLORETA 0 35.686 0.000 8002

19 121 sLORETA 10 32.283 0.000 2866

19 121 sLORETA 20 26.650 0.000 1290

19 121 sLORETA 30 21.699 0.000 597

19 121 sLORETA 40 16.409 0.000 290

19 121 sLORETA 50 12.616 0.000 143

19 121 sLORETA 60 9.888 0.000 80

19 121 sLORETA 70 9.649 0.000 42

19 121 sLORETA 80 8.755 0.000 16

19 121 sLORETA 90 7.474 0.000 8

19 121 sLORETA 100 11.425 11.425 1

19 122 Ave 0 33.077 0.000 8002

19 122 Ave 10 28.764 0.000 2259

19 122 Ave 20 24.287 0.000 974

19 122 Ave 30 20.019 0.000 453

19 122 Ave 40 15.402 0.000 225

19 122 Ave 50 12.255 0.000 127

19 122 Ave 60 11.288 0.000 67

19 122 Ave 70 10.036 0.000 36

19 122 Ave 80 6.194 0.000 10

19 122 Ave 90 4.180 2.968 3

19 122 Ave 100 3.530 3.530 1

19 122 cMEM 0 44.289 0.000 8002

19 122 cMEM 10 43.721 0.000 549

19 122 cMEM 20 43.261 0.000 245

19 122 cMEM 30 43.262 0.000 122

19 122 cMEM 40 45.529 10.928 62

19 122 cMEM 50 44.996 19.401 36

19 122 cMEM 60 45.157 19.401 23

19 122 cMEM 70 46.635 19.558 14

19 122 cMEM 80 56.990 28.830 5

19 122 cMEM 90 62.343 59.515 2

19 122 cMEM 100 64.764 64.764 1

19 122 dSPM 0 34.026 0.000 8002

19 122 dSPM 10 30.676 0.000 2126

19 122 dSPM 20 26.584 0.000 871

19 122 dSPM 30 23.379 0.000 442

19 122 dSPM 40 20.290 0.000 234

19 122 dSPM 50 17.091 0.000 121

19 122 dSPM 60 15.542 0.000 62

19 122 dSPM 70 14.362 0.000 36

19 122 dSPM 80 13.794 0.000 20

19 122 dSPM 90 8.864 0.000 8

19 122 dSPM 100 11.425 11.425 1

19 122 MNE 0 31.155 0.000 8002

19 122 MNE 10 25.069 0.000 1508

19 122 MNE 20 20.120 0.000 560

19 122 MNE 30 15.952 0.000 241

19 122 MNE 40 13.706 0.000 129

19 122 MNE 50 11.450 0.000 72

19 122 MNE 60 10.812 0.000 41

19 122 MNE 70 7.767 0.000 16

19 122 MNE 80 7.601 0.000 8

19 122 MNE 90 4.033 0.000 2

19 122 MNE 100 5.646 5.646 1

19 122 sLORETA 0 34.698 0.000 8002

19 122 sLORETA 10 31.191 0.000 2824

19 122 sLORETA 20 26.167 0.000 1304

19 122 sLORETA 30 21.328 0.000 608

19 122 sLORETA 40 16.391 0.000 302

19 122 sLORETA 50 11.969 0.000 142

19 122 sLORETA 60 9.421 0.000 78

19 122 sLORETA 70 8.816 0.000 46

19 122 sLORETA 80 7.766 0.000 17

19 122 sLORETA 90 7.283 0.000 9

19 122 sLORETA 100 3.530 3.530 1

19 123 Ave 0 34.972 0.000 8002

19 123 Ave 10 31.041 0.000 2638

19 123 Ave 20 25.459 0.000 1168

19 123 Ave 30 20.043 0.000 536

19 123 Ave 40 15.308 0.000 270

19 123 Ave 50 11.839 0.000 137

19 123 Ave 60 9.483 0.000 78

19 123 Ave 70 7.984 0.000 41

19 123 Ave 80 6.790 0.000 16

19 123 Ave 90 5.273 0.000 6

19 123 Ave 100 3.530 3.530 1

19 123 cMEM 0 66.797 0.000 8002

19 123 cMEM 10 67.836 18.944 168

19 123 cMEM 20 68.340 58.178 76

19 123 cMEM 30 68.351 58.413 57

19 123 cMEM 40 68.105 59.881 39

19 123 cMEM 50 67.900 59.881 29

19 123 cMEM 60 67.684 62.525 23

19 123 cMEM 70 68.069 62.865 14

19 123 cMEM 80 66.701 62.865 6

19 123 cMEM 90 68.221 68.221 1

19 123 cMEM 100 68.221 68.221 1

19 123 dSPM 0 35.535 0.000 8002

19 123 dSPM 10 32.396 0.000 2172

19 123 dSPM 20 28.469 0.000 920

19 123 dSPM 30 24.270 0.000 465

19 123 dSPM 40 21.156 0.000 238

19 123 dSPM 50 18.161 0.000 125

19 123 dSPM 60 16.763 0.000 62

19 123 dSPM 70 15.613 0.000 40

19 123 dSPM 80 15.386 0.000 21

19 123 dSPM 90 13.363 0.000 9

19 123 dSPM 100 11.425 11.425 1

19 123 MNE 0 32.601 0.000 8002

19 123 MNE 10 25.628 0.000 1482

19 123 MNE 20 20.345 0.000 544

19 123 MNE 30 15.992 0.000 228

19 123 MNE 40 12.746 0.000 122

19 123 MNE 50 11.270 0.000 67

19 123 MNE 60 9.617 0.000 36

19 123 MNE 70 6.144 0.000 14

19 123 MNE 80 4.980 0.000 4

19 123 MNE 90 4.066 0.000 2

19 123 MNE 100 5.646 5.646 1

19 123 sLORETA 0 35.830 0.000 8002

19 123 sLORETA 10 32.362 0.000 2936

19 123 sLORETA 20 26.471 0.000 1288

19 123 sLORETA 30 22.080 0.000 639

19 123 sLORETA 40 17.556 0.000 318

19 123 sLORETA 50 12.189 0.000 148

19 123 sLORETA 60 9.111 0.000 74

19 123 sLORETA 70 8.924 0.000 46

19 123 sLORETA 80 9.288 0.000 18

19 123 sLORETA 90 6.260 0.000 9

19 123 sLORETA 100 11.425 11.425 1

19 124 Ave 0 35.058 0.000 8002

19 124 Ave 10 31.016 0.000 2429

19 124 Ave 20 26.844 0.000 1139

19 124 Ave 30 22.766 0.000 565

19 124 Ave 40 18.442 0.000 284

19 124 Ave 50 15.477 0.000 151

19 124 Ave 60 14.317 0.000 85

19 124 Ave 70 10.561 0.000 42

19 124 Ave 80 8.056 0.000 15

19 124 Ave 90 4.222 2.968 3

19 124 Ave 100 3.530 3.530 1

19 124 cMEM 0 26.277 0.000 8002

19 124 cMEM 10 24.777 0.000 483

19 124 cMEM 20 24.225 0.000 239

19 124 cMEM 30 24.676 0.000 139

19 124 cMEM 40 25.163 0.000 84

19 124 cMEM 50 26.222 5.646 57

19 124 cMEM 60 26.971 10.812 29

19 124 cMEM 70 26.066 14.176 14

19 124 cMEM 80 25.144 19.558 8

19 124 cMEM 90 28.830 28.830 1

19 124 cMEM 100 28.830 28.830 1

19 124 dSPM 0 36.576 0.000 8002

19 124 dSPM 10 33.717 0.000 2419

19 124 dSPM 20 30.267 0.000 1114

19 124 dSPM 30 27.342 0.000 581

19 124 dSPM 40 24.000 0.000 291

19 124 dSPM 50 22.426 0.000 167

19 124 dSPM 60 19.546 0.000 88

19 124 dSPM 70 17.507 0.000 46

19 124 dSPM 80 16.997 0.000 26

19 124 dSPM 90 17.565 0.000 12

19 124 dSPM 100 26.834 26.834 1

19 124 MNE 0 36.146 0.000 8002

19 124 MNE 10 31.217 0.000 1800

19 124 MNE 20 26.742 0.000 729

19 124 MNE 30 23.169 0.000 342

19 124 MNE 40 17.549 0.000 153

19 124 MNE 50 13.862 0.000 82

19 124 MNE 60 10.569 0.000 43

19 124 MNE 70 8.364 0.000 17

19 124 MNE 80 4.438 0.000 5

19 124 MNE 90 4.253 0.000 3

19 124 MNE 100 5.646 5.646 1

19 124 sLORETA 0 37.564 0.000 8002

19 124 sLORETA 10 34.819 0.000 3174

19 124 sLORETA 20 30.047 0.000 1462

19 124 sLORETA 30 26.813 0.000 808

19 124 sLORETA 40 23.000 0.000 423

19 124 sLORETA 50 17.851 0.000 198

19 124 sLORETA 60 11.029 0.000 86

19 124 sLORETA 70 9.164 0.000 46

19 124 sLORETA 80 9.696 0.000 20

19 124 sLORETA 90 6.813 0.000 9

19 124 sLORETA 100 11.425 11.425 1

19 125 Ave 0 36.456 0.000 8002

19 125 Ave 10 32.627 0.000 2508

19 125 Ave 20 26.016 0.000 1073

19 125 Ave 30 21.678 0.000 518

19 125 Ave 40 17.789 0.000 244

19 125 Ave 50 15.820 0.000 142

19 125 Ave 60 13.711 0.000 75

19 125 Ave 70 11.257 0.000 33

19 125 Ave 80 7.357 0.000 12

19 125 Ave 90 6.437 2.968 4

19 125 Ave 100 3.530 3.530 1

19 125 cMEM 0 30.333 0.000 8002

19 125 cMEM 10 28.796 0.000 542

19 125 cMEM 20 27.785 0.000 251

19 125 cMEM 30 26.667 0.000 127

19 125 cMEM 40 27.210 2.968 80

19 125 cMEM 50 26.750 3.530 47

19 125 cMEM 60 26.561 10.812 26

19 125 cMEM 70 27.179 14.176 17

19 125 cMEM 80 28.899 19.558 10

19 125 cMEM 90 28.830 28.830 1

19 125 cMEM 100 28.830 28.830 1

19 125 dSPM 0 38.619 0.000 8002

19 125 dSPM 10 35.977 0.000 2460

19 125 dSPM 20 31.557 0.000 1133

19 125 dSPM 30 26.759 0.000 549

19 125 dSPM 40 23.810 0.000 298

19 125 dSPM 50 21.160 0.000 155

19 125 dSPM 60 19.274 0.000 79

19 125 dSPM 70 17.909 0.000 44

19 125 dSPM 80 17.082 0.000 24

19 125 dSPM 90 17.629 0.000 11

19 125 dSPM 100 26.834 26.834 1

19 125 MNE 0 36.736 0.000 8002

19 125 MNE 10 30.547 0.000 1679

19 125 MNE 20 24.609 0.000 650

19 125 MNE 30 20.780 0.000 297

19 125 MNE 40 14.984 0.000 128

19 125 MNE 50 12.871 0.000 70

19 125 MNE 60 10.530 0.000 38

19 125 MNE 70 9.875 0.000 14

19 125 MNE 80 5.000 0.000 4

19 125 MNE 90 4.111 0.000 2

19 125 MNE 100 5.646 5.646 1

19 125 sLORETA 0 39.029 0.000 8002

19 125 sLORETA 10 36.411 0.000 3210

19 125 sLORETA 20 29.466 0.000 1363

19 125 sLORETA 30 25.152 0.000 731

19 125 sLORETA 40 20.969 0.000 373

19 125 sLORETA 50 15.422 0.000 173

19 125 sLORETA 60 10.414 0.000 80

19 125 sLORETA 70 9.565 0.000 42

19 125 sLORETA 80 9.895 0.000 16

19 125 sLORETA 90 6.414 0.000 8

19 125 sLORETA 100 11.425 11.425 1

20 126 Ave 0 41.310 0.000 8002

20 126 Ave 10 39.905 0.000 3835

20 126 Ave 20 33.534 0.000 1524

20 126 Ave 30 24.591 0.000 603

20 126 Ave 40 17.122 0.000 244

20 126 Ave 50 12.177 0.000 104

20 126 Ave 60 8.618 0.000 54

20 126 Ave 70 4.631 0.000 18

20 126 Ave 80 2.763 0.000 8

20 126 Ave 90 0.000 0.000 3

20 126 Ave 100 0.000 0.000 1

20 126 cMEM 0 14.090 0.000 8002

20 126 cMEM 10 9.029 0.000 301

20 126 cMEM 20 5.630 0.000 130

20 126 cMEM 30 4.574 0.000 66

20 126 cMEM 40 4.029 0.000 45

20 126 cMEM 50 3.869 0.000 24

20 126 cMEM 60 3.511 0.000 13

20 126 cMEM 70 2.034 0.000 8

20 126 cMEM 80 0.000 0.000 4

20 126 cMEM 90 0.000 0.000 1

20 126 cMEM 100 0.000 0.000 1

20 126 dSPM 0 40.292 0.000 8002

20 126 dSPM 10 39.341 0.000 4405

20 126 dSPM 20 34.845 0.000 2088

20 126 dSPM 30 27.908 0.000 966

20 126 dSPM 40 21.957 0.000 505

20 126 dSPM 50 17.575 0.000 232

20 126 dSPM 60 13.771 0.000 101

20 126 dSPM 70 12.561 0.000 38

20 126 dSPM 80 12.234 0.000 15

20 126 dSPM 90 12.030 7.875 5

20 126 dSPM 100 14.396 14.396 1

20 126 MNE 0 47.648 0.000 8002

20 126 MNE 10 45.738 0.000 1708

20 126 MNE 20 40.482 0.000 455

20 126 MNE 30 34.717 0.000 124

20 126 MNE 40 25.036 0.000 49

20 126 MNE 50 16.743 0.000 20

20 126 MNE 60 0.000 0.000 6

20 126 MNE 70 0.000 0.000 5

20 126 MNE 80 0.000 0.000 2

20 126 MNE 90 0.000 0.000 2

20 126 MNE 100 0.000 0.000 1

20 126 sLORETA 0 40.322 0.000 8002

20 126 sLORETA 10 39.205 0.000 4083

20 126 sLORETA 20 33.960 0.000 1774

20 126 sLORETA 30 26.745 0.000 796

20 126 sLORETA 40 21.102 0.000 411

20 126 sLORETA 50 17.532 0.000 219

20 126 sLORETA 60 14.103 0.000 109

20 126 sLORETA 70 7.920 0.000 45

20 126 sLORETA 80 3.124 0.000 16

20 126 sLORETA 90 2.748 0.000 8

20 126 sLORETA 100 0.000 0.000 1

20 127 Ave 0 31.124 0.000 8002

20 127 Ave 10 27.870 0.000 2622

20 127 Ave 20 23.655 0.000 1128

20 127 Ave 30 20.938 0.000 648

20 127 Ave 40 18.330 0.000 339

20 127 Ave 50 14.620 0.000 170

20 127 Ave 60 10.154 0.000 76

20 127 Ave 70 5.295 0.000 33

20 127 Ave 80 5.225 0.000 11

20 127 Ave 90 0.000 0.000 3

20 127 Ave 100 0.000 0.000 1

20 127 cMEM 0 7.163 0.000 8002

20 127 cMEM 10 4.451 0.000 320

20 127 cMEM 20 3.944 0.000 189

20 127 cMEM 30 2.954 0.000 111

20 127 cMEM 40 1.345 0.000 59

20 127 cMEM 50 1.099 0.000 34

20 127 cMEM 60 1.233 0.000 10

20 127 cMEM 70 0.000 0.000 6

20 127 cMEM 80 0.000 0.000 3

20 127 cMEM 90 0.000 0.000 1

20 127 cMEM 100 0.000 0.000 1

20 127 dSPM 0 32.316 0.000 8002

20 127 dSPM 10 30.019 0.000 2926

20 127 dSPM 20 26.396 0.000 1354

20 127 dSPM 30 23.203 0.000 739

20 127 dSPM 40 21.746 0.000 439

20 127 dSPM 50 19.440 0.000 231

20 127 dSPM 60 15.338 0.000 94

20 127 dSPM 70 10.952 0.000 36

20 127 dSPM 80 9.438 0.000 15

20 127 dSPM 90 10.330 4.371 4

20 127 dSPM 100 14.396 14.396 1

20 127 MNE 0 33.686 0.000 8002

20 127 MNE 10 27.612 0.000 1050

20 127 MNE 20 22.371 0.000 343

20 127 MNE 30 18.946 0.000 137

20 127 MNE 40 13.890 0.000 50

20 127 MNE 50 2.184 0.000 19

20 127 MNE 60 0.000 0.000 5

20 127 MNE 70 0.000 0.000 4

20 127 MNE 80 0.000 0.000 2

20 127 MNE 90 0.000 0.000 1

20 127 MNE 100 0.000 0.000 1

20 127 sLORETA 0 31.212 0.000 8002

20 127 sLORETA 10 28.822 0.000 2939

20 127 sLORETA 20 25.456 0.000 1362

20 127 sLORETA 30 23.582 0.000 835

20 127 sLORETA 40 21.575 0.000 503

20 127 sLORETA 50 20.037 0.000 309

20 127 sLORETA 60 17.470 0.000 171

20 127 sLORETA 70 14.467 0.000 80

20 127 sLORETA 80 12.136 0.000 28

20 127 sLORETA 90 3.500 0.000 11

20 127 sLORETA 100 0.000 0.000 1

20 128 Ave 0 33.406 0.000 8002

20 128 Ave 10 29.793 0.000 2347

20 128 Ave 20 24.727 0.000 944

20 128 Ave 30 20.678 0.000 487

20 128 Ave 40 17.394 0.000 237

20 128 Ave 50 13.160 0.000 99

20 128 Ave 60 6.185 0.000 38

20 128 Ave 70 4.026 0.000 16

20 128 Ave 80 0.000 0.000 5

20 128 Ave 90 0.000 0.000 1

20 128 Ave 100 0.000 0.000 1

20 128 cMEM 0 11.773 0.000 8002

20 128 cMEM 10 10.068 0.000 311

20 128 cMEM 20 9.738 0.000 119

20 128 cMEM 30 9.857 0.000 70

20 128 cMEM 40 9.730 0.000 41

20 128 cMEM 50 9.769 0.000 21

20 128 cMEM 60 8.342 0.000 11

20 128 cMEM 70 0.000 0.000 7

20 128 cMEM 80 0.000 0.000 4

20 128 cMEM 90 0.000 0.000 1

20 128 cMEM 100 0.000 0.000 1

20 128 dSPM 0 34.493 0.000 8002

20 128 dSPM 10 32.480 0.000 3069

20 128 dSPM 20 28.579 0.000 1445

20 128 dSPM 30 25.230 0.000 794

20 128 dSPM 40 22.411 0.000 443

20 128 dSPM 50 20.594 0.000 239

20 128 dSPM 60 17.057 0.000 105

20 128 dSPM 70 11.063 0.000 28

20 128 dSPM 80 9.758 0.000 14

20 128 dSPM 90 12.812 7.875 4

20 128 dSPM 100 14.396 14.396 1

20 128 MNE 0 36.961 0.000 8002

20 128 MNE 10 31.619 0.000 1107

20 128 MNE 20 25.404 0.000 358

20 128 MNE 30 21.820 0.000 140

20 128 MNE 40 17.799 0.000 54

20 128 MNE 50 2.225 0.000 17

20 128 MNE 60 0.000 0.000 6

20 128 MNE 70 0.000 0.000 5

20 128 MNE 80 0.000 0.000 2

20 128 MNE 90 0.000 0.000 1

20 128 MNE 100 0.000 0.000 1

20 128 sLORETA 0 33.383 0.000 8002

20 128 sLORETA 10 31.132 0.000 2925

20 128 sLORETA 20 27.043 0.000 1307

20 128 sLORETA 30 24.872 0.000 759

20 128 sLORETA 40 22.541 0.000 469

20 128 sLORETA 50 20.309 0.000 276

20 128 sLORETA 60 18.680 0.000 153

20 128 sLORETA 70 15.678 0.000 59

20 128 sLORETA 80 12.955 0.000 22

20 128 sLORETA 90 0.000 0.000 5

20 128 sLORETA 100 0.000 0.000 1

20 129 Ave 0 31.439 0.000 8002

20 129 Ave 10 28.543 0.000 2814

20 129 Ave 20 23.394 0.000 1231

20 129 Ave 30 20.013 0.000 685

20 129 Ave 40 16.822 0.000 356

20 129 Ave 50 12.435 0.000 167

20 129 Ave 60 7.389 0.000 75

20 129 Ave 70 6.471 0.000 38

20 129 Ave 80 6.436 0.000 15

20 129 Ave 90 4.503 0.000 3

20 129 Ave 100 0.000 0.000 1

20 129 cMEM 0 11.893 0.000 8002

20 129 cMEM 10 5.785 0.000 319

20 129 cMEM 20 4.972 0.000 168

20 129 cMEM 30 4.293 0.000 96

20 129 cMEM 40 3.852 0.000 59

20 129 cMEM 50 3.648 0.000 30

20 129 cMEM 60 2.514 0.000 14

20 129 cMEM 70 3.486 0.000 5

20 129 cMEM 80 4.186 3.017 2

20 129 cMEM 90 4.893 4.893 1

20 129 cMEM 100 4.893 4.893 1

20 129 dSPM 0 32.857 0.000 8002

20 129 dSPM 10 30.669 0.000 2943

20 129 dSPM 20 26.864 0.000 1350

20 129 dSPM 30 24.275 0.000 757

20 129 dSPM 40 22.330 0.000 432

20 129 dSPM 50 20.977 0.000 236

20 129 dSPM 60 17.815 0.000 90

20 129 dSPM 70 12.925 0.000 34

20 129 dSPM 80 10.110 4.371 15

20 129 dSPM 90 11.397 4.371 6

20 129 dSPM 100 14.396 14.396 1

20 129 MNE 0 35.560 0.000 8002

20 129 MNE 10 31.283 0.000 1555

20 129 MNE 20 24.115 0.000 490

20 129 MNE 30 18.345 0.000 196

20 129 MNE 40 13.849 0.000 89

20 129 MNE 50 7.857 0.000 34

20 129 MNE 60 1.994 0.000 13

20 129 MNE 70 0.000 0.000 5

20 129 MNE 80 0.000 0.000 1

20 129 MNE 90 0.000 0.000 1

20 129 MNE 100 0.000 0.000 1

20 129 sLORETA 0 31.416 0.000 8002

20 129 sLORETA 10 29.396 0.000 3182

20 129 sLORETA 20 25.275 0.000 1500

20 129 sLORETA 30 22.915 0.000 882

20 129 sLORETA 40 21.241 0.000 560

20 129 sLORETA 50 19.029 0.000 339

20 129 sLORETA 60 15.440 0.000 173

20 129 sLORETA 70 13.490 0.000 77

20 129 sLORETA 80 6.808 0.000 29

20 129 sLORETA 90 6.988 0.000 8

20 129 sLORETA 100 0.000 0.000 1

20 130 Ave 0 28.726 0.000 8002

20 130 Ave 10 25.070 0.000 2169

20 130 Ave 20 21.214 0.000 996

20 130 Ave 30 19.149 0.000 570

20 130 Ave 40 16.205 0.000 296

20 130 Ave 50 12.776 0.000 146

20 130 Ave 60 7.800 0.000 64

20 130 Ave 70 5.879 0.000 30

20 130 Ave 80 4.761 0.000 11

20 130 Ave 90 0.000 0.000 3

20 130 Ave 100 0.000 0.000 1

20 130 cMEM 0 16.442 0.000 8002

20 130 cMEM 10 12.356 0.000 527

20 130 cMEM 20 8.980 0.000 249

20 130 cMEM 30 7.666 0.000 154

20 130 cMEM 40 7.125 0.000 92

20 130 cMEM 50 4.748 0.000 49

20 130 cMEM 60 3.903 0.000 36

20 130 cMEM 70 4.471 0.000 18

20 130 cMEM 80 3.816 0.000 10

20 130 cMEM 90 0.000 0.000 3

20 130 cMEM 100 0.000 0.000 1

20 130 dSPM 0 30.795 0.000 8002

20 130 dSPM 10 28.124 0.000 2383

20 130 dSPM 20 25.546 0.000 1173

20 130 dSPM 30 23.563 0.000 695

20 130 dSPM 40 22.036 0.000 403

20 130 dSPM 50 21.172 0.000 212

20 130 dSPM 60 20.321 0.000 110

20 130 dSPM 70 17.572 0.000 45

20 130 dSPM 80 11.103 4.371 9

20 130 dSPM 90 10.089 4.371 4

20 130 dSPM 100 4.371 4.371 1

20 130 MNE 0 31.359 0.000 8002

20 130 MNE 10 24.882 0.000 1084

20 130 MNE 20 18.681 0.000 362

20 130 MNE 30 13.238 0.000 144

20 130 MNE 40 7.209 0.000 62

20 130 MNE 50 2.599 0.000 22

20 130 MNE 60 0.000 0.000 9

20 130 MNE 70 0.000 0.000 3

20 130 MNE 80 0.000 0.000 2

20 130 MNE 90 0.000 0.000 2

20 130 MNE 100 0.000 0.000 1

20 130 sLORETA 0 29.445 0.000 8002

20 130 sLORETA 10 27.135 0.000 2706

20 130 sLORETA 20 24.394 0.000 1396

20 130 sLORETA 30 22.664 0.000 843

20 130 sLORETA 40 21.354 0.000 589

20 130 sLORETA 50 19.588 0.000 369

20 130 sLORETA 60 16.581 0.000 214

20 130 sLORETA 70 11.996 0.000 110

20 130 sLORETA 80 8.359 0.000 38

20 130 sLORETA 90 6.111 0.000 13

20 130 sLORETA 100 0.000 0.000 1

20 131 Ave 0 30.133 0.000 8002

20 131 Ave 10 27.546 0.000 2765

20 131 Ave 20 23.483 0.000 1281

20 131 Ave 30 21.305 0.000 752

20 131 Ave 40 19.336 0.000 470

20 131 Ave 50 15.935 0.000 250

20 131 Ave 60 11.830 0.000 138

20 131 Ave 70 9.098 0.000 72

20 131 Ave 80 6.673 0.000 36

20 131 Ave 90 5.590 0.000 9

20 131 Ave 100 0.000 0.000 1

20 131 cMEM 0 11.571 0.000 8002

20 131 cMEM 10 7.835 0.000 453

20 131 cMEM 20 6.958 0.000 253

20 131 cMEM 30 6.360 0.000 139

20 131 cMEM 40 6.033 0.000 75

20 131 cMEM 50 2.564 0.000 41

20 131 cMEM 60 1.503 0.000 19

20 131 cMEM 70 0.000 0.000 11

20 131 cMEM 80 0.000 0.000 8

20 131 cMEM 90 0.000 0.000 3

20 131 cMEM 100 0.000 0.000 1

20 131 dSPM 0 32.211 0.000 8002

20 131 dSPM 10 29.747 0.000 2623

20 131 dSPM 20 26.712 0.000 1210

20 131 dSPM 30 24.571 0.000 693

20 131 dSPM 40 23.192 0.000 402

20 131 dSPM 50 22.561 0.000 225

20 131 dSPM 60 20.405 0.000 98

20 131 dSPM 70 15.240 0.000 33

20 131 dSPM 80 10.374 4.371 13

20 131 dSPM 90 10.826 4.371 3

20 131 dSPM 100 14.396 14.396 1

20 131 MNE 0 33.234 0.000 8002

20 131 MNE 10 27.475 0.000 1188

20 131 MNE 20 20.153 0.000 381

20 131 MNE 30 16.255 0.000 162

20 131 MNE 40 13.472 0.000 66

20 131 MNE 50 2.026 0.000 26

20 131 MNE 60 0.000 0.000 7

20 131 MNE 70 0.000 0.000 4

20 131 MNE 80 0.000 0.000 2

20 131 MNE 90 0.000 0.000 1

20 131 MNE 100 0.000 0.000 1

20 131 sLORETA 0 30.602 0.000 8002

20 131 sLORETA 10 28.478 0.000 2860

20 131 sLORETA 20 25.175 0.000 1396

20 131 sLORETA 30 23.560 0.000 856

20 131 sLORETA 40 22.644 0.000 598

20 131 sLORETA 50 20.551 0.000 354

20 131 sLORETA 60 15.770 0.000 199

20 131 sLORETA 70 13.158 0.000 106

20 131 sLORETA 80 7.322 0.000 33

20 131 sLORETA 90 5.302 0.000 14

20 131 sLORETA 100 0.000 0.000 1

21 132 Ave 0 46.634 0.000 8002

21 132 Ave 10 44.110 0.000 3725

21 132 Ave 20 37.184 0.000 1713

21 132 Ave 30 32.230 0.000 915

21 132 Ave 40 26.640 0.000 484

21 132 Ave 50 24.411 0.000 270

21 132 Ave 60 23.845 0.000 141

21 132 Ave 70 23.365 0.000 65

21 132 Ave 80 18.097 0.000 22

21 132 Ave 90 1.738 0.000 6

21 132 Ave 100 0.000 0.000 1

21 132 cMEM 0 23.791 0.000 8002

21 132 cMEM 10 18.488 0.000 459

21 132 cMEM 20 11.482 0.000 169

21 132 cMEM 30 6.753 0.000 68

21 132 cMEM 40 5.418 0.000 36

21 132 cMEM 50 0.000 0.000 18

21 132 cMEM 60 0.000 0.000 16

21 132 cMEM 70 0.000 0.000 12

21 132 cMEM 80 0.000 0.000 7

21 132 cMEM 90 0.000 0.000 4

21 132 cMEM 100 0.000 0.000 1

21 132 dSPM 0 47.423 0.000 8002

21 132 dSPM 10 44.587 0.000 3391

21 132 dSPM 20 37.540 0.000 1506

21 132 dSPM 30 30.225 0.000 742

21 132 dSPM 40 24.516 0.000 398

21 132 dSPM 50 18.837 0.000 184

21 132 dSPM 60 13.927 0.000 75

21 132 dSPM 70 10.798 0.000 33

21 132 dSPM 80 11.223 0.000 15

21 132 dSPM 90 11.229 0.000 6

21 132 dSPM 100 19.816 19.816 1

21 132 MNE 0 48.632 0.000 8002

21 132 MNE 10 44.800 0.000 2373

21 132 MNE 20 37.695 0.000 923

21 132 MNE 30 31.741 0.000 397

21 132 MNE 40 28.958 0.000 201

21 132 MNE 50 27.505 0.000 91

21 132 MNE 60 28.380 0.000 45

21 132 MNE 70 28.958 0.000 20

21 132 MNE 80 34.136 0.000 7

21 132 MNE 90 36.010 36.010 1

21 132 MNE 100 36.010 36.010 1

21 132 sLORETA 0 49.716 0.000 8002

21 132 sLORETA 10 47.560 0.000 3715

21 132 sLORETA 20 42.474 0.000 1736

21 132 sLORETA 30 38.480 0.000 943

21 132 sLORETA 40 33.949 0.000 462

21 132 sLORETA 50 33.478 0.000 255

21 132 sLORETA 60 35.160 0.000 133

21 132 sLORETA 70 38.678 0.000 56

21 132 sLORETA 80 42.646 4.457 23

21 132 sLORETA 90 38.837 32.648 5

21 132 sLORETA 100 47.171 47.171 1

21 133 Ave 0 43.762 0.000 8002

21 133 Ave 10 39.642 0.000 3068

21 133 Ave 20 26.670 0.000 1182

21 133 Ave 30 19.029 0.000 579

21 133 Ave 40 15.640 0.000 286

21 133 Ave 50 13.734 0.000 131

21 133 Ave 60 14.202 0.000 55

21 133 Ave 70 2.267 0.000 14

21 133 Ave 80 1.845 0.000 5

21 133 Ave 90 0.000 0.000 1

21 133 Ave 100 0.000 0.000 1

21 133 cMEM 0 23.306 0.000 8002

21 133 cMEM 10 13.866 0.000 236

21 133 cMEM 20 5.635 0.000 76

21 133 cMEM 30 1.950 0.000 41

21 133 cMEM 40 0.000 0.000 18

21 133 cMEM 50 0.000 0.000 14

21 133 cMEM 60 0.000 0.000 10

21 133 cMEM 70 0.000 0.000 6

21 133 cMEM 80 0.000 0.000 3

21 133 cMEM 90 0.000 0.000 2

21 133 cMEM 100 0.000 0.000 1

21 133 dSPM 0 43.773 0.000 8002

21 133 dSPM 10 40.049 0.000 2898

21 133 dSPM 20 29.578 0.000 1192

21 133 dSPM 30 23.977 0.000 610

21 133 dSPM 40 19.229 0.000 296

21 133 dSPM 50 15.153 0.000 152

21 133 dSPM 60 10.435 0.000 63

21 133 dSPM 70 9.370 0.000 36

21 133 dSPM 80 9.058 0.000 18

21 133 dSPM 90 8.199 0.000 7

21 133 dSPM 100 4.457 4.457 1

21 133 MNE 0 47.522 0.000 8002

21 133 MNE 10 43.561 0.000 2176

21 133 MNE 20 35.468 0.000 870

21 133 MNE 30 28.145 0.000 403

21 133 MNE 40 22.015 0.000 208

21 133 MNE 50 20.899 0.000 107

21 133 MNE 60 23.327 0.000 45

21 133 MNE 70 28.227 0.000 19

21 133 MNE 80 36.340 0.000 6

21 133 MNE 90 35.505 32.344 3

21 133 MNE 100 36.010 36.010 1

21 133 sLORETA 0 45.461 0.000 8002

21 133 sLORETA 10 43.395 0.000 3932

21 133 sLORETA 20 35.098 0.000 1743

21 133 sLORETA 30 26.917 0.000 926

21 133 sLORETA 40 23.007 0.000 526

21 133 sLORETA 50 21.916 0.000 290

21 133 sLORETA 60 21.055 0.000 148

21 133 sLORETA 70 18.210 0.000 79

21 133 sLORETA 80 16.186 0.000 31

21 133 sLORETA 90 15.497 0.000 12

21 133 sLORETA 100 4.457 4.457 1

21 134 Ave 0 47.676 0.000 8002

21 134 Ave 10 45.267 0.000 3536

21 134 Ave 20 38.840 0.000 1634

21 134 Ave 30 32.796 0.000 871

21 134 Ave 40 27.631 0.000 453

21 134 Ave 50 25.872 0.000 252

21 134 Ave 60 22.738 0.000 119

21 134 Ave 70 19.524 0.000 51

21 134 Ave 80 20.673 0.000 21

21 134 Ave 90 18.643 0.000 3

21 134 Ave 100 0.000 0.000 1

21 134 cMEM 0 13.515 0.000 8002

21 134 cMEM 10 9.008 0.000 380

21 134 cMEM 20 7.899 0.000 191

21 134 cMEM 30 6.476 0.000 98

21 134 cMEM 40 4.552 0.000 49

21 134 cMEM 50 2.024 0.000 25

21 134 cMEM 60 2.145 0.000 16

21 134 cMEM 70 0.000 0.000 9

21 134 cMEM 80 0.000 0.000 3

21 134 cMEM 90 0.000 0.000 2

21 134 cMEM 100 0.000 0.000 1

21 134 dSPM 0 48.360 0.000 8002

21 134 dSPM 10 44.420 0.000 2804

21 134 dSPM 20 37.574 0.000 1174

21 134 dSPM 30 28.799 0.000 503

21 134 dSPM 40 22.317 0.000 211

21 134 dSPM 50 17.840 0.000 78

21 134 dSPM 60 14.970 0.000 25

21 134 dSPM 70 16.061 0.000 9

21 134 dSPM 80 17.959 14.547 3

21 134 dSPM 90 19.816 19.816 1

21 134 dSPM 100 19.816 19.816 1

21 134 MNE 0 50.484 0.000 8002

21 134 MNE 10 47.381 0.000 2389

21 134 MNE 20 40.224 0.000 895

21 134 MNE 30 34.313 0.000 406

21 134 MNE 40 30.942 0.000 198

21 134 MNE 50 28.553 0.000 97

21 134 MNE 60 26.613 0.000 42

21 134 MNE 70 27.238 0.000 22

21 134 MNE 80 30.468 0.000 13

21 134 MNE 90 30.127 0.000 7

21 134 MNE 100 0.000 0.000 1

21 134 sLORETA 0 51.142 0.000 8002

21 134 sLORETA 10 49.358 0.000 3680

21 134 sLORETA 20 44.634 0.000 1768

21 134 sLORETA 30 41.122 0.000 959

21 134 sLORETA 40 37.538 0.000 517

21 134 sLORETA 50 35.471 0.000 278

21 134 sLORETA 60 36.486 0.000 135

21 134 sLORETA 70 39.029 0.000 55

21 134 sLORETA 80 39.910 27.593 19

21 134 sLORETA 90 36.749 30.287 5

21 134 sLORETA 100 30.287 30.287 1

21 135 Ave 0 52.914 0.000 8002

21 135 Ave 10 51.224 0.000 4232

21 135 Ave 20 45.485 0.000 2108

21 135 Ave 30 34.595 0.000 899

21 135 Ave 40 19.495 0.000 353

21 135 Ave 50 12.010 0.000 155

21 135 Ave 60 9.606 0.000 68

21 135 Ave 70 4.968 0.000 22

21 135 Ave 80 1.521 0.000 8

21 135 Ave 90 0.000 0.000 2

21 135 Ave 100 0.000 0.000 1

21 135 cMEM 0 32.957 0.000 8002

21 135 cMEM 10 30.297 0.000 694

21 135 cMEM 20 24.448 0.000 207

21 135 cMEM 30 19.231 0.000 109

21 135 cMEM 40 14.599 0.000 68

21 135 cMEM 50 12.004 0.000 45

21 135 cMEM 60 5.521 0.000 27

21 135 cMEM 70 1.390 0.000 17

21 135 cMEM 80 0.000 0.000 7

21 135 cMEM 90 0.000 0.000 4

21 135 cMEM 100 0.000 0.000 1

21 135 dSPM 0 50.711 0.000 8002

21 135 dSPM 10 48.598 0.000 3768

21 135 dSPM 20 40.901 0.000 1615

21 135 dSPM 30 33.003 0.000 747

21 135 dSPM 40 29.232 0.000 388

21 135 dSPM 50 23.491 0.000 179

21 135 dSPM 60 15.518 0.000 81

21 135 dSPM 70 12.228 0.000 34

21 135 dSPM 80 7.440 0.000 16

21 135 dSPM 90 7.194 0.000 7

21 135 dSPM 100 0.000 0.000 1

21 135 MNE 0 58.717 0.000 8002

21 135 MNE 10 57.378 0.000 3399

21 135 MNE 20 53.718 0.000 1454

21 135 MNE 30 47.320 0.000 647

21 135 MNE 40 43.035 0.000 286

21 135 MNE 50 34.043 0.000 136

21 135 MNE 60 29.682 0.000 52

21 135 MNE 70 16.876 0.000 23

21 135 MNE 80 17.743 0.000 12

21 135 MNE 90 22.233 0.000 4

21 135 MNE 100 19.690 19.690 1

21 135 sLORETA 0 54.835 0.000 8002

21 135 sLORETA 10 53.945 0.000 4885

21 135 sLORETA 20 50.427 0.000 2787

21 135 sLORETA 30 45.770 0.000 1586

21 135 sLORETA 40 38.827 0.000 759

21 135 sLORETA 50 31.093 0.000 361

21 135 sLORETA 60 19.567 0.000 159

21 135 sLORETA 70 18.259 0.000 83

21 135 sLORETA 80 13.715 0.000 32

21 135 sLORETA 90 11.469 0.000 9

21 135 sLORETA 100 4.457 4.457 1

21 136 Ave 0 39.586 0.000 8002

21 136 Ave 10 32.413 0.000 2478

21 136 Ave 20 17.556 0.000 870

21 136 Ave 30 9.351 0.000 379

21 136 Ave 40 6.269 0.000 191

21 136 Ave 50 5.441 0.000 106

21 136 Ave 60 3.780 0.000 45

21 136 Ave 70 2.538 0.000 21

21 136 Ave 80 0.000 0.000 4

21 136 Ave 90 0.000 0.000 1

21 136 Ave 100 0.000 0.000 1

21 136 cMEM 0 13.718 0.000 8002

21 136 cMEM 10 4.207 0.000 257

21 136 cMEM 20 2.052 0.000 83

21 136 cMEM 30 1.837 0.000 58

21 136 cMEM 40 1.763 0.000 42

21 136 cMEM 50 0.887 0.000 26

21 136 cMEM 60 0.000 0.000 19

21 136 cMEM 70 0.000 0.000 13

21 136 cMEM 80 0.000 0.000 9

21 136 cMEM 90 0.000 0.000 3

21 136 cMEM 100 0.000 0.000 1

21 136 dSPM 0 40.698 0.000 8002

21 136 dSPM 10 34.650 0.000 2354

21 136 dSPM 20 26.084 0.000 959

21 136 dSPM 30 20.825 0.000 478

21 136 dSPM 40 13.107 0.000 228

21 136 dSPM 50 10.710 0.000 128

21 136 dSPM 60 9.004 0.000 65

21 136 dSPM 70 7.530 0.000 29

21 136 dSPM 80 6.024 0.000 11

21 136 dSPM 90 6.627 0.000 5

21 136 dSPM 100 4.457 4.457 1

21 136 MNE 0 44.265 0.000 8002

21 136 MNE 10 39.849 0.000 2401

21 136 MNE 20 30.456 0.000 908

21 136 MNE 30 22.850 0.000 440

21 136 MNE 40 16.187 0.000 249

21 136 MNE 50 9.426 0.000 148

21 136 MNE 60 4.486 0.000 78

21 136 MNE 70 1.910 0.000 39

21 136 MNE 80 0.000 0.000 15

21 136 MNE 90 0.000 0.000 8

21 136 MNE 100 0.000 0.000 1

21 136 sLORETA 0 41.341 0.000 8002

21 136 sLORETA 10 37.207 0.000 3238

21 136 sLORETA 20 25.815 0.000 1324

21 136 sLORETA 30 17.084 0.000 641

21 136 sLORETA 40 12.633 0.000 306

21 136 sLORETA 50 9.176 0.000 164

21 136 sLORETA 60 7.830 0.000 98

21 136 sLORETA 70 7.222 0.000 49

21 136 sLORETA 80 4.481 0.000 22

21 136 sLORETA 90 3.787 0.000 8

21 136 sLORETA 100 0.000 0.000 1

21 137 Ave 0 46.135 0.000 8002

21 137 Ave 10 42.755 0.000 3382

21 137 Ave 20 31.558 0.000 1334

21 137 Ave 30 24.168 0.000 634

21 137 Ave 40 21.437 0.000 332

21 137 Ave 50 18.625 0.000 152

21 137 Ave 60 17.809 0.000 55

21 137 Ave 70 14.281 0.000 15

21 137 Ave 80 0.000 0.000 4

21 137 Ave 90 0.000 0.000 1

21 137 Ave 100 0.000 0.000 1

21 137 cMEM 0 22.586 0.000 8002

21 137 cMEM 10 17.089 0.000 560

21 137 cMEM 20 13.686 0.000 224

21 137 cMEM 30 9.164 0.000 92

21 137 cMEM 40 7.614 0.000 60

21 137 cMEM 50 6.068 0.000 37

21 137 cMEM 60 4.815 0.000 26

21 137 cMEM 70 0.000 0.000 14

21 137 cMEM 80 0.000 0.000 6

21 137 cMEM 90 0.000 0.000 2

21 137 cMEM 100 0.000 0.000 1

21 137 dSPM 0 46.229 0.000 8002

21 137 dSPM 10 43.272 0.000 3293

21 137 dSPM 20 34.436 0.000 1409

21 137 dSPM 30 25.771 0.000 684

21 137 dSPM 40 18.817 0.000 354

21 137 dSPM 50 15.558 0.000 167

21 137 dSPM 60 13.479 0.000 88

21 137 dSPM 70 12.472 0.000 46

21 137 dSPM 80 10.901 0.000 21

21 137 dSPM 90 9.414 0.000 7

21 137 dSPM 100 0.000 0.000 1

21 137 MNE 0 50.273 0.000 8002

21 137 MNE 10 47.067 0.000 2537

21 137 MNE 20 39.040 0.000 959

21 137 MNE 30 31.575 0.000 448

21 137 MNE 40 26.513 0.000 222

21 137 MNE 50 25.773 0.000 108

21 137 MNE 60 26.042 0.000 51

21 137 MNE 70 28.672 0.000 24

21 137 MNE 80 33.626 0.000 11

21 137 MNE 90 36.438 30.287 4

21 137 MNE 100 36.010 36.010 1

21 137 sLORETA 0 48.989 0.000 8002

21 137 sLORETA 10 47.286 0.000 4134

21 137 sLORETA 20 40.948 0.000 1989

21 137 sLORETA 30 34.780 0.000 1068

21 137 sLORETA 40 31.129 0.000 594

21 137 sLORETA 50 30.220 0.000 331

21 137 sLORETA 60 30.767 0.000 174

21 137 sLORETA 70 31.712 0.000 75

21 137 sLORETA 80 33.018 0.000 30

21 137 sLORETA 90 33.311 0.000 9

21 137 sLORETA 100 30.287 30.287 1

21 138 Ave 0 37.748 0.000 8002

21 138 Ave 10 31.891 0.000 2499

21 138 Ave 20 20.190 0.000 987

21 138 Ave 30 13.172 0.000 502

21 138 Ave 40 10.473 0.000 272

21 138 Ave 50 7.684 0.000 135

21 138 Ave 60 5.174 0.000 67

21 138 Ave 70 5.018 0.000 28

21 138 Ave 80 6.014 0.000 12

21 138 Ave 90 0.000 0.000 1

21 138 Ave 100 0.000 0.000 1

21 138 cMEM 0 15.435 0.000 8002

21 138 cMEM 10 11.250 0.000 440

21 138 cMEM 20 7.852 0.000 174

21 138 cMEM 30 6.001 0.000 93

21 138 cMEM 40 4.359 0.000 61

21 138 cMEM 50 4.513 0.000 45

21 138 cMEM 60 4.745 0.000 31

21 138 cMEM 70 5.256 0.000 19

21 138 cMEM 80 6.058 0.000 9

21 138 cMEM 90 5.377 0.000 4

21 138 cMEM 100 7.980 7.980 1

21 138 dSPM 0 38.800 0.000 8002

21 138 dSPM 10 32.757 0.000 2187

21 138 dSPM 20 24.982 0.000 927

21 138 dSPM 30 20.882 0.000 454

21 138 dSPM 40 15.439 0.000 236

21 138 dSPM 50 11.125 0.000 120

21 138 dSPM 60 10.469 0.000 53

21 138 dSPM 70 9.382 0.000 20

21 138 dSPM 80 9.497 0.000 9

21 138 dSPM 90 9.261 0.000 3

21 138 dSPM 100 14.547 14.547 1

21 138 MNE 0 42.614 0.000 8002

21 138 MNE 10 36.982 0.000 1764

21 138 MNE 20 26.483 0.000 646

21 138 MNE 30 20.071 0.000 326

21 138 MNE 40 17.062 0.000 184

21 138 MNE 50 14.364 0.000 86

21 138 MNE 60 16.302 0.000 29

21 138 MNE 70 16.525 0.000 11

21 138 MNE 80 2.428 0.000 5

21 138 MNE 90 1.534 0.000 2

21 138 MNE 100 2.100 2.100 1

21 138 sLORETA 0 40.623 0.000 8002

21 138 sLORETA 10 36.979 0.000 3193

21 138 sLORETA 20 27.237 0.000 1360

21 138 sLORETA 30 18.666 0.000 704

21 138 sLORETA 40 15.403 0.000 394

21 138 sLORETA 50 13.563 0.000 196

21 138 sLORETA 60 11.148 0.000 105

21 138 sLORETA 70 9.465 0.000 48

21 138 sLORETA 80 5.311 0.000 21

21 138 sLORETA 90 3.010 0.000 5

21 138 sLORETA 100 4.844 4.844 1

21 139 Ave 0 36.611 0.000 8002

21 139 Ave 10 28.785 0.000 2222

21 139 Ave 20 15.316 0.000 886

21 139 Ave 30 8.972 0.000 454

21 139 Ave 40 6.753 0.000 259

21 139 Ave 50 5.386 0.000 142

21 139 Ave 60 5.396 0.000 63

21 139 Ave 70 4.630 0.000 30

21 139 Ave 80 5.637 0.000 12

21 139 Ave 90 0.000 0.000 1

21 139 Ave 100 0.000 0.000 1

21 139 cMEM 0 12.951 0.000 8002

21 139 cMEM 10 8.707 0.000 348

21 139 cMEM 20 4.866 0.000 135

21 139 cMEM 30 4.816 0.000 91

21 139 cMEM 40 4.703 0.000 64

21 139 cMEM 50 4.733 0.000 49

21 139 cMEM 60 4.954 0.000 37

21 139 cMEM 70 4.743 0.000 25

21 139 cMEM 80 5.179 0.000 13

21 139 cMEM 90 4.474 0.000 7

21 139 cMEM 100 7.980 7.980 1

21 139 dSPM 0 36.963 0.000 8002

21 139 dSPM 10 29.122 0.000 2006

21 139 dSPM 20 20.820 0.000 857

21 139 dSPM 30 17.593 0.000 477

21 139 dSPM 40 15.467 0.000 256

21 139 dSPM 50 12.077 0.000 144

21 139 dSPM 60 10.365 0.000 67

21 139 dSPM 70 8.618 0.000 26

21 139 dSPM 80 6.407 0.000 12

21 139 dSPM 90 6.302 0.000 6

21 139 dSPM 100 0.000 0.000 1

21 139 MNE 0 43.037 0.000 8002

21 139 MNE 10 38.328 0.000 2090

21 139 MNE 20 27.782 0.000 779

21 139 MNE 30 17.695 0.000 405

21 139 MNE 40 10.293 0.000 234

21 139 MNE 50 4.306 0.000 136

21 139 MNE 60 3.679 0.000 83

21 139 MNE 70 2.068 0.000 40

21 139 MNE 80 1.536 0.000 15

21 139 MNE 90 0.000 0.000 4

21 139 MNE 100 0.000 0.000 1

21 139 sLORETA 0 38.719 0.000 8002

21 139 sLORETA 10 33.294 0.000 2754

21 139 sLORETA 20 21.387 0.000 1204

21 139 sLORETA 30 13.183 0.000 604

21 139 sLORETA 40 10.449 0.000 323

21 139 sLORETA 50 8.008 0.000 180

21 139 sLORETA 60 7.015 0.000 86

21 139 sLORETA 70 6.782 0.000 44

21 139 sLORETA 80 6.555 0.000 19

21 139 sLORETA 90 2.987 0.000 5

21 139 sLORETA 100 4.844 4.844 1

21 140 Ave 0 45.069 0.000 8002

21 140 Ave 10 41.666 0.000 3204

21 140 Ave 20 32.621 0.000 1305

21 140 Ave 30 26.878 0.000 661

21 140 Ave 40 23.085 0.000 333

21 140 Ave 50 22.458 0.000 166

21 140 Ave 60 19.945 0.000 86

21 140 Ave 70 19.597 0.000 39

21 140 Ave 80 16.961 0.000 17

21 140 Ave 90 0.000 0.000 5

21 140 Ave 100 0.000 0.000 1

21 140 cMEM 0 10.384 0.000 8002

21 140 cMEM 10 3.154 0.000 187

21 140 cMEM 20 0.000 0.000 54

21 140 cMEM 30 0.000 0.000 36

21 140 cMEM 40 0.000 0.000 30

21 140 cMEM 50 0.000 0.000 25

21 140 cMEM 60 0.000 0.000 16

21 140 cMEM 70 0.000 0.000 13

21 140 cMEM 80 0.000 0.000 8

21 140 cMEM 90 0.000 0.000 3

21 140 cMEM 100 0.000 0.000 1

21 140 dSPM 0 46.153 0.000 8002

21 140 dSPM 10 43.166 0.000 3283

21 140 dSPM 20 34.716 0.000 1412

21 140 dSPM 30 28.635 0.000 701

21 140 dSPM 40 22.425 0.000 385

21 140 dSPM 50 18.322 0.000 171

21 140 dSPM 60 14.623 0.000 72

21 140 dSPM 70 10.235 0.000 26

21 140 dSPM 80 10.573 0.000 12

21 140 dSPM 90 11.949 0.000 5

21 140 dSPM 100 0.000 0.000 1

21 140 MNE 0 46.327 0.000 8002

21 140 MNE 10 41.086 0.000 1942

21 140 MNE 20 31.703 0.000 678

21 140 MNE 30 25.508 0.000 289

21 140 MNE 40 22.785 0.000 135

21 140 MNE 50 21.841 0.000 69

21 140 MNE 60 24.042 0.000 40

21 140 MNE 70 29.763 0.000 15

21 140 MNE 80 33.827 0.000 6

21 140 MNE 90 34.371 32.344 2

21 140 MNE 100 36.010 36.010 1

21 140 sLORETA 0 47.505 0.000 8002

21 140 sLORETA 10 45.291 0.000 3493

21 140 sLORETA 20 38.935 0.000 1568

21 140 sLORETA 30 33.941 0.000 809

21 140 sLORETA 40 31.036 0.000 456

21 140 sLORETA 50 30.027 0.000 231

21 140 sLORETA 60 30.811 0.000 125

21 140 sLORETA 70 31.492 0.000 67

21 140 sLORETA 80 33.596 0.000 26

21 140 sLORETA 90 35.021 21.312 9

21 140 sLORETA 100 32.648 32.648 1

21 141 Ave 0 35.969 0.000 8002

21 141 Ave 10 29.720 0.000 2227

21 141 Ave 20 18.974 0.000 886

21 141 Ave 30 11.749 0.000 470

21 141 Ave 40 7.666 0.000 251

21 141 Ave 50 5.946 0.000 123

21 141 Ave 60 5.146 0.000 68

21 141 Ave 70 4.861 0.000 29

21 141 Ave 80 3.998 0.000 9

21 141 Ave 90 0.000 0.000 2

21 141 Ave 100 0.000 0.000 1

21 141 cMEM 0 8.270 0.000 8002

21 141 cMEM 10 6.600 0.000 347

21 141 cMEM 20 5.797 0.000 200

21 141 cMEM 30 5.462 0.000 145

21 141 cMEM 40 5.388 0.000 99

21 141 cMEM 50 5.207 0.000 69

21 141 cMEM 60 4.877 0.000 37

21 141 cMEM 70 3.361 0.000 16

21 141 cMEM 80 2.562 0.000 8

21 141 cMEM 90 0.000 0.000 3

21 141 cMEM 100 0.000 0.000 1

21 141 dSPM 0 36.606 0.000 8002

21 141 dSPM 10 29.715 0.000 1978

21 141 dSPM 20 22.026 0.000 844

21 141 dSPM 30 19.997 0.000 427

21 141 dSPM 40 17.686 0.000 236

21 141 dSPM 50 14.761 0.000 117

21 141 dSPM 60 12.060 0.000 51

21 141 dSPM 70 10.668 0.000 15

21 141 dSPM 80 9.067 0.000 10

21 141 dSPM 90 8.934 0.000 3

21 141 dSPM 100 14.547 14.547 1

21 141 MNE 0 43.362 0.000 8002

21 141 MNE 10 39.045 0.000 1857

21 141 MNE 20 30.361 0.000 690

21 141 MNE 30 23.140 0.000 356

21 141 MNE 40 16.116 0.000 208

21 141 MNE 50 11.149 0.000 102

21 141 MNE 60 2.250 0.000 47

21 141 MNE 70 1.701 0.000 15

21 141 MNE 80 0.847 0.000 6

21 141 MNE 90 0.000 0.000 2

21 141 MNE 100 0.000 0.000 1

21 141 sLORETA 0 38.541 0.000 8002

21 141 sLORETA 10 34.346 0.000 2911

21 141 sLORETA 20 25.700 0.000 1276

21 141 sLORETA 30 17.524 0.000 699

21 141 sLORETA 40 13.212 0.000 375

21 141 sLORETA 50 10.001 0.000 197

21 141 sLORETA 60 7.811 0.000 93

21 141 sLORETA 70 6.755 0.000 45

21 141 sLORETA 80 5.205 0.000 20

21 141 sLORETA 90 2.978 0.000 5

21 141 sLORETA 100 4.844 4.844 1

21 142 Ave 0 50.792 0.000 8002

21 142 Ave 10 48.392 0.000 3516

21 142 Ave 20 41.651 0.000 1573

21 142 Ave 30 30.455 0.000 744

21 142 Ave 40 22.983 0.000 346

21 142 Ave 50 10.650 0.000 138

21 142 Ave 60 6.241 0.000 55

21 142 Ave 70 1.776 0.000 25

21 142 Ave 80 1.326 0.000 10

21 142 Ave 90 0.000 0.000 1

21 142 Ave 100 0.000 0.000 1

21 142 cMEM 0 11.586 0.000 8002

21 142 cMEM 10 5.817 0.000 256

21 142 cMEM 20 3.993 0.000 75

21 142 cMEM 30 1.931 0.000 43

21 142 cMEM 40 1.352 0.000 32

21 142 cMEM 50 0.000 0.000 23

21 142 cMEM 60 0.000 0.000 15

21 142 cMEM 70 0.000 0.000 9

21 142 cMEM 80 0.000 0.000 4

21 142 cMEM 90 0.000 0.000 2

21 142 cMEM 100 0.000 0.000 1

21 142 dSPM 0 50.264 0.000 8002

21 142 dSPM 10 48.085 0.000 3685

21 142 dSPM 20 41.090 0.000 1644

21 142 dSPM 30 31.910 0.000 821

21 142 dSPM 40 23.928 0.000 398

21 142 dSPM 50 16.537 0.000 184

21 142 dSPM 60 14.915 0.000 92

21 142 dSPM 70 14.014 0.000 45

21 142 dSPM 80 10.642 0.000 16

21 142 dSPM 90 10.512 0.000 6

21 142 dSPM 100 4.457 4.457 1

21 142 MNE 0 55.335 0.000 8002

21 142 MNE 10 52.920 0.000 2408

21 142 MNE 20 46.940 0.000 964

21 142 MNE 30 39.245 0.000 405

21 142 MNE 40 28.886 0.000 179

21 142 MNE 50 23.290 0.000 81

21 142 MNE 60 18.885 0.000 33

21 142 MNE 70 11.506 0.000 14

21 142 MNE 80 2.447 0.000 5

21 142 MNE 90 2.100 2.100 1

21 142 MNE 100 2.100 2.100 1

21 142 sLORETA 0 52.897 0.000 8002

21 142 sLORETA 10 51.469 0.000 4121

21 142 sLORETA 20 47.658 0.000 2132

21 142 sLORETA 30 41.896 0.000 1176

21 142 sLORETA 40 34.322 0.000 617

21 142 sLORETA 50 30.267 0.000 317

21 142 sLORETA 60 25.804 0.000 155

21 142 sLORETA 70 20.744 0.000 54

21 142 sLORETA 80 2.257 0.000 14

21 142 sLORETA 90 2.522 0.000 7

21 142 sLORETA 100 0.000 0.000 1

21 143 Ave 0 40.126 0.000 8002

21 143 Ave 10 34.111 0.000 2845

21 143 Ave 20 23.161 0.000 1093

21 143 Ave 30 13.684 0.000 491

21 143 Ave 40 6.906 0.000 238

21 143 Ave 50 5.994 0.000 122

21 143 Ave 60 5.242 0.000 60

21 143 Ave 70 4.608 0.000 20

21 143 Ave 80 5.275 0.000 5

21 143 Ave 90 0.000 0.000 1

21 143 Ave 100 0.000 0.000 1

21 143 cMEM 0 7.967 0.000 8002

21 143 cMEM 10 4.494 0.000 195

21 143 cMEM 20 4.251 0.000 87

21 143 cMEM 30 4.119 0.000 74

21 143 cMEM 40 3.678 0.000 51

21 143 cMEM 50 3.182 0.000 35

21 143 cMEM 60 1.494 0.000 16

21 143 cMEM 70 0.000 0.000 11

21 143 cMEM 80 0.000 0.000 4

21 143 cMEM 90 0.000 0.000 2

21 143 cMEM 100 0.000 0.000 1

21 143 dSPM 0 42.280 0.000 8002

21 143 dSPM 10 37.427 0.000 2751

21 143 dSPM 20 29.556 0.000 1082

21 143 dSPM 30 24.901 0.000 550

21 143 dSPM 40 18.627 0.000 273

21 143 dSPM 50 14.548 0.000 143

21 143 dSPM 60 11.665 0.000 55

21 143 dSPM 70 10.942 0.000 24

21 143 dSPM 80 8.076 0.000 7

21 143 dSPM 90 14.547 14.547 1

21 143 dSPM 100 14.547 14.547 1

21 143 MNE 0 43.217 0.000 8002

21 143 MNE 10 37.271 0.000 2000

21 143 MNE 20 27.531 0.000 719

21 143 MNE 30 20.453 0.000 326

21 143 MNE 40 18.354 0.000 166

21 143 MNE 50 7.824 0.000 78

21 143 MNE 60 1.866 0.000 32

21 143 MNE 70 2.126 0.000 12

21 143 MNE 80 2.286 0.000 6

21 143 MNE 90 1.554 0.000 2

21 143 MNE 100 2.100 2.100 1

21 143 sLORETA 0 41.883 0.000 8002

21 143 sLORETA 10 38.549 0.000 3684

21 143 sLORETA 20 29.074 0.000 1582

21 143 sLORETA 30 21.134 0.000 783

21 143 sLORETA 40 14.400 0.000 411

21 143 sLORETA 50 10.066 0.000 208

21 143 sLORETA 60 7.714 0.000 109

21 143 sLORETA 70 6.629 0.000 58

21 143 sLORETA 80 7.297 0.000 19

21 143 sLORETA 90 5.583 0.000 8

21 143 sLORETA 100 4.844 4.844 1

21 144 Ave 0 43.062 0.000 8002

21 144 Ave 10 39.083 0.000 3302

21 144 Ave 20 27.716 0.000 1284

21 144 Ave 30 18.423 0.000 615

21 144 Ave 40 12.260 0.000 331

21 144 Ave 50 7.142 0.000 155

21 144 Ave 60 4.406 0.000 73

21 144 Ave 70 3.005 0.000 26

21 144 Ave 80 2.901 0.000 8

21 144 Ave 90 2.491 0.000 3

21 144 Ave 100 0.000 0.000 1

21 144 cMEM 0 10.078 0.000 8002

21 144 cMEM 10 3.731 0.000 186

21 144 cMEM 20 1.098 0.000 82

21 144 cMEM 30 0.000 0.000 46

21 144 cMEM 40 0.000 0.000 37

21 144 cMEM 50 0.000 0.000 26

21 144 cMEM 60 0.000 0.000 13

21 144 cMEM 70 0.000 0.000 7

21 144 cMEM 80 0.000 0.000 4

21 144 cMEM 90 0.000 0.000 2

21 144 cMEM 100 0.000 0.000 1

21 144 dSPM 0 43.118 0.000 8002

21 144 dSPM 10 38.880 0.000 2857

21 144 dSPM 20 28.040 0.000 1064

21 144 dSPM 30 21.552 0.000 495

21 144 dSPM 40 17.542 0.000 258

21 144 dSPM 50 11.763 0.000 130

21 144 dSPM 60 10.575 0.000 59

21 144 dSPM 70 9.026 0.000 26

21 144 dSPM 80 7.013 0.000 11

21 144 dSPM 90 8.180 0.000 5

21 144 dSPM 100 4.457 4.457 1

21 144 MNE 0 45.315 0.000 8002

21 144 MNE 10 41.983 0.000 2708

21 144 MNE 20 34.545 0.000 1119

21 144 MNE 30 28.078 0.000 580

21 144 MNE 40 24.943 0.000 326

21 144 MNE 50 20.118 0.000 175

21 144 MNE 60 17.249 0.000 94

21 144 MNE 70 9.671 0.000 40

21 144 MNE 80 7.567 0.000 17

21 144 MNE 90 2.217 0.000 6

21 144 MNE 100 0.000 0.000 1

21 144 sLORETA 0 45.670 0.000 8002

21 144 sLORETA 10 43.086 0.000 3822

21 144 sLORETA 20 34.824 0.000 1710

21 144 sLORETA 30 24.067 0.000 811

21 144 sLORETA 40 18.505 0.000 418

21 144 sLORETA 50 14.139 0.000 221

21 144 sLORETA 60 9.407 0.000 94

21 144 sLORETA 70 6.924 0.000 53

21 144 sLORETA 80 3.876 0.000 20

21 144 sLORETA 90 3.728 0.000 4

21 144 sLORETA 100 4.457 4.457 1

21 145 Ave 0 38.361 0.000 8002

21 145 Ave 10 33.782 0.000 3079

21 145 Ave 20 21.816 0.000 1187

21 145 Ave 30 16.404 0.000 623

21 145 Ave 40 13.533 0.000 349

21 145 Ave 50 12.272 0.000 185

21 145 Ave 60 9.073 0.000 97

21 145 Ave 70 5.660 0.000 39

21 145 Ave 80 5.046 0.000 19

21 145 Ave 90 5.440 0.000 3

21 145 Ave 100 6.648 6.648 1

21 145 cMEM 0 15.133 0.000 8002

21 145 cMEM 10 10.469 0.000 459

21 145 cMEM 20 9.375 0.000 239

21 145 cMEM 30 8.615 0.000 129

21 145 cMEM 40 7.926 0.000 70

21 145 cMEM 50 6.177 0.000 44

21 145 cMEM 60 5.360 0.000 32

21 145 cMEM 70 5.171 0.000 15

21 145 cMEM 80 4.646 0.000 11

21 145 cMEM 90 5.107 0.000 6

21 145 cMEM 100 6.648 6.648 1

21 145 dSPM 0 39.244 0.000 8002

21 145 dSPM 10 34.268 0.000 2663

21 145 dSPM 20 24.968 0.000 1052

21 145 dSPM 30 20.889 0.000 535

21 145 dSPM 40 17.639 0.000 288

21 145 dSPM 50 15.469 0.000 153

21 145 dSPM 60 13.893 0.000 77

21 145 dSPM 70 12.424 0.000 32

21 145 dSPM 80 11.110 0.000 10

21 145 dSPM 90 11.397 0.000 5

21 145 dSPM 100 14.547 14.547 1

21 145 MNE 0 42.869 0.000 8002

21 145 MNE 10 37.686 0.000 2054

21 145 MNE 20 28.240 0.000 748

21 145 MNE 30 19.432 0.000 358

21 145 MNE 40 17.493 0.000 186

21 145 MNE 50 18.388 0.000 102

21 145 MNE 60 18.533 0.000 33

21 145 MNE 70 20.603 0.000 13

21 145 MNE 80 18.595 0.000 7

21 145 MNE 90 3.103 0.000 3

21 145 MNE 100 2.100 2.100 1

21 145 sLORETA 0 42.136 0.000 8002

21 145 sLORETA 10 39.658 0.000 3841

21 145 sLORETA 20 31.922 0.000 1820

21 145 sLORETA 30 23.436 0.000 911

21 145 sLORETA 40 20.078 0.000 526

21 145 sLORETA 50 18.410 0.000 310

21 145 sLORETA 60 17.124 0.000 165

21 145 sLORETA 70 15.616 0.000 75

21 145 sLORETA 80 11.762 0.000 26

21 145 sLORETA 90 5.572 0.000 9

21 145 sLORETA 100 4.844 4.844 1

21 146 Ave 0 35.897 0.000 8002

21 146 Ave 10 27.335 0.000 2073

21 146 Ave 20 14.082 0.000 749

21 146 Ave 30 9.992 0.000 408

21 146 Ave 40 8.368 0.000 225

21 146 Ave 50 7.155 0.000 106

21 146 Ave 60 6.930 0.000 49

21 146 Ave 70 7.725 0.000 21

21 146 Ave 80 7.870 0.000 10

21 146 Ave 90 9.491 6.648 3

21 146 Ave 100 6.648 6.648 1

21 146 cMEM 0 7.568 0.000 8002

21 146 cMEM 10 5.875 0.000 248

21 146 cMEM 20 5.317 0.000 126

21 146 cMEM 30 5.565 0.000 66

21 146 cMEM 40 6.093 0.000 39

21 146 cMEM 50 6.974 0.000 19

21 146 cMEM 60 7.409 0.000 11

21 146 cMEM 70 7.717 0.000 8

21 146 cMEM 80 6.784 0.000 5

21 146 cMEM 90 7.290 6.648 2

21 146 cMEM 100 6.648 6.648 1

21 146 dSPM 0 36.600 0.000 8002

21 146 dSPM 10 26.207 0.000 1621

21 146 dSPM 20 18.885 0.000 613

21 146 dSPM 30 16.611 0.000 311

21 146 dSPM 40 13.791 0.000 154

21 146 dSPM 50 11.387 0.000 65

21 146 dSPM 60 11.468 0.000 22

21 146 dSPM 70 10.754 0.000 11

21 146 dSPM 80 15.946 12.667 3

21 146 dSPM 90 14.547 14.547 1

21 146 dSPM 100 14.547 14.547 1

21 146 MNE 0 41.252 0.000 8002

21 146 MNE 10 35.732 0.000 1809

21 146 MNE 20 25.145 0.000 671

21 146 MNE 30 15.180 0.000 344

21 146 MNE 40 9.038 0.000 183

21 146 MNE 50 8.286 0.000 104

21 146 MNE 60 6.719 0.000 45

21 146 MNE 70 2.868 0.000 13

21 146 MNE 80 2.637 0.000 7

21 146 MNE 90 2.470 0.000 5

21 146 MNE 100 2.100 2.100 1

21 146 sLORETA 0 37.843 0.000 8002

21 146 sLORETA 10 32.608 0.000 2865

21 146 sLORETA 20 19.772 0.000 1109

21 146 sLORETA 30 13.531 0.000 571

21 146 sLORETA 40 11.159 0.000 333

21 146 sLORETA 50 9.638 0.000 189

21 146 sLORETA 60 8.198 0.000 90

21 146 sLORETA 70 7.972 0.000 45

21 146 sLORETA 80 9.540 0.000 16

21 146 sLORETA 90 10.970 4.844 2

21 146 sLORETA 100 14.547 14.547 1

21 147 Ave 0 46.398 0.000 8002

21 147 Ave 10 43.797 0.000 3582

21 147 Ave 20 37.635 0.000 1683

21 147 Ave 30 32.192 0.000 894

21 147 Ave 40 27.260 0.000 437

21 147 Ave 50 26.806 0.000 229

21 147 Ave 60 26.192 0.000 107

21 147 Ave 70 26.367 0.000 42

21 147 Ave 80 18.455 0.000 17

21 147 Ave 90 0.000 0.000 7

21 147 Ave 100 0.000 0.000 1

21 147 cMEM 0 23.716 0.000 8002

21 147 cMEM 10 16.750 0.000 440

21 147 cMEM 20 10.242 0.000 122

21 147 cMEM 30 4.647 0.000 29

21 147 cMEM 40 0.000 0.000 16

21 147 cMEM 50 0.000 0.000 13

21 147 cMEM 60 0.000 0.000 11

21 147 cMEM 70 0.000 0.000 8

21 147 cMEM 80 0.000 0.000 5

21 147 cMEM 90 0.000 0.000 3

21 147 cMEM 100 0.000 0.000 1

21 147 dSPM 0 47.580 0.000 8002

21 147 dSPM 10 44.679 0.000 3229

21 147 dSPM 20 38.585 0.000 1495

21 147 dSPM 30 32.506 0.000 737

21 147 dSPM 40 24.344 0.000 309

21 147 dSPM 50 18.754 0.000 145

21 147 dSPM 60 13.384 0.000 53

21 147 dSPM 70 11.526 0.000 26

21 147 dSPM 80 10.777 0.000 9

21 147 dSPM 90 19.816 19.816 1

21 147 dSPM 100 19.816 19.816 1

21 147 MNE 0 48.166 0.000 8002

21 147 MNE 10 44.101 0.000 2135

21 147 MNE 20 37.001 0.000 807

21 147 MNE 30 32.654 0.000 345

21 147 MNE 40 31.202 0.000 154

21 147 MNE 50 30.900 0.000 74

21 147 MNE 60 30.581 0.000 32

21 147 MNE 70 34.708 0.000 16

21 147 MNE 80 33.700 0.000 9

21 147 MNE 90 36.010 36.010 1

21 147 MNE 100 36.010 36.010 1

21 147 sLORETA 0 48.816 0.000 8002

21 147 sLORETA 10 46.494 0.000 3528

21 147 sLORETA 20 41.654 0.000 1676

21 147 sLORETA 30 37.564 0.000 854

21 147 sLORETA 40 34.034 0.000 394

21 147 sLORETA 50 34.321 0.000 204

21 147 sLORETA 60 37.240 0.000 98

21 147 sLORETA 70 40.517 0.000 45

21 147 sLORETA 80 40.003 21.312 17

21 147 sLORETA 90 39.041 21.312 6

21 147 sLORETA 100 47.171 47.171 1

21 148 Ave 0 40.088 0.000 8002

21 148 Ave 10 35.606 0.000 3017

21 148 Ave 20 22.827 0.000 1180

21 148 Ave 30 14.538 0.000 639

21 148 Ave 40 11.450 0.000 355

21 148 Ave 50 9.420 0.000 208

21 148 Ave 60 6.462 0.000 104

21 148 Ave 70 5.169 0.000 42

21 148 Ave 80 5.082 0.000 18

21 148 Ave 90 4.073 0.000 8

21 148 Ave 100 6.648 6.648 1

21 148 cMEM 0 7.574 0.000 8002

21 148 cMEM 10 5.024 0.000 180

21 148 cMEM 20 4.637 0.000 87

21 148 cMEM 30 4.837 0.000 56

21 148 cMEM 40 5.009 0.000 45

21 148 cMEM 50 5.294 0.000 28

21 148 cMEM 60 5.384 0.000 21

21 148 cMEM 70 5.026 0.000 13

21 148 cMEM 80 5.103 0.000 7

21 148 cMEM 90 5.220 0.000 4

21 148 cMEM 100 0.000 0.000 1

21 148 dSPM 0 39.811 0.000 8002

21 148 dSPM 10 32.758 0.000 2254

21 148 dSPM 20 21.926 0.000 851

21 148 dSPM 30 18.559 0.000 422

21 148 dSPM 40 16.435 0.000 221

21 148 dSPM 50 14.104 0.000 103

21 148 dSPM 60 12.146 0.000 39

21 148 dSPM 70 10.260 0.000 14

21 148 dSPM 80 13.062 0.000 5

21 148 dSPM 90 17.176 14.547 2

21 148 dSPM 100 14.547 14.547 1

21 148 MNE 0 44.074 0.000 8002

21 148 MNE 10 39.989 0.000 2239

21 148 MNE 20 30.191 0.000 851

21 148 MNE 30 20.862 0.000 424

21 148 MNE 40 17.336 0.000 245

21 148 MNE 50 13.018 0.000 140

21 148 MNE 60 10.864 0.000 68

21 148 MNE 70 12.655 0.000 39

21 148 MNE 80 5.398 0.000 13

21 148 MNE 90 2.035 0.000 3

21 148 MNE 100 0.000 0.000 1

21 148 sLORETA 0 42.451 0.000 8002

21 148 sLORETA 10 39.598 0.000 3618

21 148 sLORETA 20 30.342 0.000 1618

21 148 sLORETA 30 19.992 0.000 861

21 148 sLORETA 40 16.368 0.000 496

21 148 sLORETA 50 14.370 0.000 276

21 148 sLORETA 60 11.394 0.000 148

21 148 sLORETA 70 9.643 0.000 66

21 148 sLORETA 80 6.967 0.000 27

21 148 sLORETA 90 6.743 0.000 6

21 148 sLORETA 100 4.844 4.844 1

21 149 Ave 0 39.130 0.000 8002

21 149 Ave 10 33.451 0.000 2494

21 149 Ave 20 20.929 0.000 971

21 149 Ave 30 12.660 0.000 514

21 149 Ave 40 9.968 0.000 286

21 149 Ave 50 8.912 0.000 161

21 149 Ave 60 7.497 0.000 68

21 149 Ave 70 5.482 0.000 26

21 149 Ave 80 3.983 0.000 7

21 149 Ave 90 0.000 0.000 1

21 149 Ave 100 0.000 0.000 1

21 149 cMEM 0 9.282 0.000 8002

21 149 cMEM 10 6.823 0.000 323

21 149 cMEM 20 5.497 0.000 130

21 149 cMEM 30 3.891 0.000 68

21 149 cMEM 40 3.191 0.000 47

21 149 cMEM 50 3.217 0.000 34

21 149 cMEM 60 3.275 0.000 23

21 149 cMEM 70 2.913 0.000 13

21 149 cMEM 80 2.219 0.000 9

21 149 cMEM 90 0.000 0.000 3

21 149 cMEM 100 0.000 0.000 1

21 149 dSPM 0 37.808 0.000 8002

21 149 dSPM 10 31.616 0.000 2234

21 149 dSPM 20 19.818 0.000 850

21 149 dSPM 30 18.021 0.000 483

21 149 dSPM 40 16.810 0.000 282

21 149 dSPM 50 14.921 0.000 160

21 149 dSPM 60 13.854 0.000 81

21 149 dSPM 70 13.583 0.000 36

21 149 dSPM 80 12.526 0.000 13

21 149 dSPM 90 8.706 0.000 3

21 149 dSPM 100 14.547 14.547 1

21 149 MNE 0 45.789 0.000 8002

21 149 MNE 10 42.158 0.000 2140

21 149 MNE 20 32.928 0.000 831

21 149 MNE 30 23.040 0.000 414

21 149 MNE 40 13.253 0.000 225

21 149 MNE 50 10.147 0.000 126

21 149 MNE 60 10.023 0.000 69

21 149 MNE 70 5.037 0.000 30

21 149 MNE 80 2.064 0.000 9

21 149 MNE 90 3.126 0.000 3

21 149 MNE 100 5.110 5.110 1

21 149 sLORETA 0 41.661 0.000 8002

21 149 sLORETA 10 38.111 0.000 3165

21 149 sLORETA 20 29.343 0.000 1382

21 149 sLORETA 30 20.709 0.000 731

21 149 sLORETA 40 14.264 0.000 407

21 149 sLORETA 50 11.224 0.000 228

21 149 sLORETA 60 10.169 0.000 125

21 149 sLORETA 70 7.837 0.000 50

21 149 sLORETA 80 7.404 0.000 20

21 149 sLORETA 90 2.550 0.000 4

21 149 sLORETA 100 4.844 4.844 1

22 150 Ave 0 16.300 0.000 8002

22 150 Ave 10 13.069 0.000 2285

22 150 Ave 20 10.183 0.000 1115

22 150 Ave 30 5.948 0.000 536

22 150 Ave 40 2.204 0.000 260

22 150 Ave 50 1.616 0.000 138

22 150 Ave 60 1.508 0.000 70

22 150 Ave 70 1.605 0.000 35

22 150 Ave 80 1.253 0.000 15

22 150 Ave 90 1.883 0.000 3

22 150 Ave 100 0.000 0.000 1

22 150 cMEM 0 18.331 0.000 8002

22 150 cMEM 10 18.024 0.000 1331

22 150 cMEM 20 18.122 0.000 615

22 150 cMEM 30 18.221 0.000 307

22 150 cMEM 40 18.258 0.000 152

22 150 cMEM 50 18.421 0.000 100

22 150 cMEM 60 16.921 0.000 57

22 150 cMEM 70 16.906 0.000 37

22 150 cMEM 80 13.391 0.000 12

22 150 cMEM 90 14.546 0.000 4

22 150 cMEM 100 14.230 14.230 1

22 150 dSPM 0 18.378 0.000 8002

22 150 dSPM 10 16.157 0.000 2417

22 150 dSPM 20 14.045 0.000 1277

22 150 dSPM 30 11.649 0.000 687

22 150 dSPM 40 9.344 0.000 368

22 150 dSPM 50 5.447 0.000 169

22 150 dSPM 60 0.582 0.000 70

22 150 dSPM 70 0.000 0.000 33

22 150 dSPM 80 0.000 0.000 11

22 150 dSPM 90 0.000 0.000 3

22 150 dSPM 100 0.000 0.000 1

22 150 MNE 0 16.208 0.000 8002

22 150 MNE 10 10.425 0.000 1380

22 150 MNE 20 6.952 0.000 555

22 150 MNE 30 4.644 0.000 232

22 150 MNE 40 2.612 0.000 102

22 150 MNE 50 2.831 0.000 57

22 150 MNE 60 2.414 0.000 30

22 150 MNE 70 2.869 0.000 18

22 150 MNE 80 2.627 0.000 12

22 150 MNE 90 3.022 0.000 3

22 150 MNE 100 0.000 0.000 1

22 150 sLORETA 0 16.368 0.000 8002

22 150 sLORETA 10 13.949 0.000 2499

22 150 sLORETA 20 11.621 0.000 1305

22 150 sLORETA 30 8.745 0.000 738

22 150 sLORETA 40 5.495 0.000 430

22 150 sLORETA 50 2.437 0.000 238

22 150 sLORETA 60 1.030 0.000 134

22 150 sLORETA 70 0.403 0.000 67

22 150 sLORETA 80 0.593 0.000 26

22 150 sLORETA 90 0.000 0.000 9

22 150 sLORETA 100 0.000 0.000 1

22 151 Ave 0 17.901 0.000 8002

22 151 Ave 10 14.866 0.000 2802

22 151 Ave 20 10.696 0.000 1352

22 151 Ave 30 7.041 0.000 735

22 151 Ave 40 3.372 0.000 386

22 151 Ave 50 2.094 0.000 208

22 151 Ave 60 1.599 0.000 114

22 151 Ave 70 1.683 0.000 61

22 151 Ave 80 1.943 0.000 32

22 151 Ave 90 1.317 0.000 13

22 151 Ave 100 0.000 0.000 1

22 151 cMEM 0 17.275 0.000 8002

22 151 cMEM 10 16.828 0.000 907

22 151 cMEM 20 15.999 0.000 344

22 151 cMEM 30 15.707 0.000 145

22 151 cMEM 40 14.851 0.000 73

22 151 cMEM 50 11.936 0.000 33

22 151 cMEM 60 10.109 0.000 14

22 151 cMEM 70 9.792 0.000 3

22 151 cMEM 80 14.230 14.230 1

22 151 cMEM 90 14.230 14.230 1

22 151 cMEM 100 14.230 14.230 1

22 151 dSPM 0 19.528 0.000 8002

22 151 dSPM 10 16.414 0.000 2651

22 151 dSPM 20 12.746 0.000 1288

22 151 dSPM 30 10.278 0.000 688

22 151 dSPM 40 7.841 0.000 339

22 151 dSPM 50 4.988 0.000 156

22 151 dSPM 60 0.655 0.000 67

22 151 dSPM 70 0.000 0.000 32

22 151 dSPM 80 0.000 0.000 11

22 151 dSPM 90 0.000 0.000 3

22 151 dSPM 100 0.000 0.000 1

22 151 MNE 0 19.855 0.000 8002

22 151 MNE 10 14.895 0.000 1565

22 151 MNE 20 9.524 0.000 578

22 151 MNE 30 6.331 0.000 253

22 151 MNE 40 3.615 0.000 123

22 151 MNE 50 3.119 0.000 56

22 151 MNE 60 2.445 0.000 35

22 151 MNE 70 3.057 0.000 18

22 151 MNE 80 3.501 0.000 10

22 151 MNE 90 3.298 0.000 4

22 151 MNE 100 5.387 5.387 1

22 151 sLORETA 0 16.918 0.000 8002

22 151 sLORETA 10 14.121 0.000 2689

22 151 sLORETA 20 11.041 0.000 1324

22 151 sLORETA 30 8.007 0.000 744

22 151 sLORETA 40 4.115 0.000 424

22 151 sLORETA 50 1.623 0.000 237

22 151 sLORETA 60 1.434 0.000 135

22 151 sLORETA 70 1.040 0.000 73

22 151 sLORETA 80 0.625 0.000 26

22 151 sLORETA 90 0.000 0.000 6

22 151 sLORETA 100 0.000 0.000 1

22 152 Ave 0 17.588 0.000 8002

22 152 Ave 10 15.166 0.000 2896

22 152 Ave 20 12.236 0.000 1485

22 152 Ave 30 8.078 0.000 732

22 152 Ave 40 4.335 0.000 384

22 152 Ave 50 2.287 0.000 200

22 152 Ave 60 1.033 0.000 118

22 152 Ave 70 1.090 0.000 51

22 152 Ave 80 1.076 0.000 29

22 152 Ave 90 0.000 0.000 10

22 152 Ave 100 0.000 0.000 1

22 152 cMEM 0 18.148 0.000 8002

22 152 cMEM 10 17.084 0.000 657

22 152 cMEM 20 15.134 0.000 156

22 152 cMEM 30 13.457 0.000 52

22 152 cMEM 40 10.346 0.000 9

22 152 cMEM 50 12.539 0.000 2

22 152 cMEM 60 14.230 14.230 1

22 152 cMEM 70 14.230 14.230 1

22 152 cMEM 80 14.230 14.230 1

22 152 cMEM 90 14.230 14.230 1

22 152 cMEM 100 14.230 14.230 1

22 152 dSPM 0 19.164 0.000 8002

22 152 dSPM 10 16.979 0.000 2619

22 152 dSPM 20 14.609 0.000 1281

22 152 dSPM 30 12.279 0.000 645

22 152 dSPM 40 9.860 0.000 346

22 152 dSPM 50 7.133 0.000 158

22 152 dSPM 60 3.228 0.000 58

22 152 dSPM 70 0.000 0.000 24

22 152 dSPM 80 0.000 0.000 8

22 152 dSPM 90 0.000 0.000 2

22 152 dSPM 100 0.000 0.000 1

22 152 MNE 0 18.080 0.000 8002

22 152 MNE 10 13.795 0.000 1640

22 152 MNE 20 9.361 0.000 601

22 152 MNE 30 6.486 0.000 262

22 152 MNE 40 3.975 0.000 107

22 152 MNE 50 2.005 0.000 50

22 152 MNE 60 2.325 0.000 25

22 152 MNE 70 2.551 0.000 19

22 152 MNE 80 2.123 0.000 7

22 152 MNE 90 3.612 0.000 2

22 152 MNE 100 0.000 0.000 1

22 152 sLORETA 0 17.201 0.000 8002

22 152 sLORETA 10 15.076 0.000 2964

22 152 sLORETA 20 12.344 0.000 1566

22 152 sLORETA 30 7.709 0.000 773

22 152 sLORETA 40 4.403 0.000 445

22 152 sLORETA 50 2.274 0.000 246

22 152 sLORETA 60 0.856 0.000 155

22 152 sLORETA 70 0.344 0.000 81

22 152 sLORETA 80 0.000 0.000 38

22 152 sLORETA 90 0.000 0.000 15

22 152 sLORETA 100 0.000 0.000 1

22 153 Ave 0 16.346 0.000 8002

22 153 Ave 10 13.327 0.000 2528

22 153 Ave 20 9.828 0.000 1266

22 153 Ave 30 5.757 0.000 656

22 153 Ave 40 2.607 0.000 341

22 153 Ave 50 2.010 0.000 180

22 153 Ave 60 1.572 0.000 90

22 153 Ave 70 1.745 0.000 47

22 153 Ave 80 1.068 0.000 22

22 153 Ave 90 1.658 0.000 8

22 153 Ave 100 0.000 0.000 1

22 153 cMEM 0 20.575 0.000 8002

22 153 cMEM 10 20.200 0.000 1004

22 153 cMEM 20 19.421 0.000 366

22 153 cMEM 30 19.177 0.000 141

22 153 cMEM 40 19.302 0.000 74

22 153 cMEM 50 17.949 0.000 34

22 153 cMEM 60 21.401 0.000 13

22 153 cMEM 70 29.029 14.230 4

22 153 cMEM 80 30.277 14.230 3

22 153 cMEM 90 41.543 41.543 1

22 153 cMEM 100 41.543 41.543 1

22 153 dSPM 0 17.947 0.000 8002

22 153 dSPM 10 15.472 0.000 2519

22 153 dSPM 20 12.995 0.000 1309

22 153 dSPM 30 10.400 0.000 729

22 153 dSPM 40 7.821 0.000 372

22 153 dSPM 50 3.617 0.000 174

22 153 dSPM 60 1.217 0.000 81

22 153 dSPM 70 0.648 0.000 38

22 153 dSPM 80 0.000 0.000 10

22 153 dSPM 90 0.000 0.000 4

22 153 dSPM 100 0.000 0.000 1

22 153 MNE 0 15.861 0.000 8002

22 153 MNE 10 10.563 0.000 1415

22 153 MNE 20 6.129 0.000 550

22 153 MNE 30 4.560 0.000 235

22 153 MNE 40 2.400 0.000 115

22 153 MNE 50 2.134 0.000 59

22 153 MNE 60 2.289 0.000 37

22 153 MNE 70 2.702 0.000 23

22 153 MNE 80 2.802 0.000 11

22 153 MNE 90 3.199 0.000 3

22 153 MNE 100 5.387 5.387 1

22 153 sLORETA 0 16.027 0.000 8002

22 153 sLORETA 10 13.298 0.000 2513

22 153 sLORETA 20 10.282 0.000 1276

22 153 sLORETA 30 6.805 0.000 684

22 153 sLORETA 40 3.118 0.000 379

22 153 sLORETA 50 1.568 0.000 208

22 153 sLORETA 60 1.138 0.000 110

22 153 sLORETA 70 0.871 0.000 53

22 153 sLORETA 80 0.675 0.000 21

22 153 sLORETA 90 0.000 0.000 5

22 153 sLORETA 100 0.000 0.000 1

23 154 Ave 0 27.384 0.000 8002

23 154 Ave 10 21.786 0.000 1844

23 154 Ave 20 14.367 0.000 786

23 154 Ave 30 8.585 0.000 400

23 154 Ave 40 6.963 0.000 242

23 154 Ave 50 5.367 0.000 136

23 154 Ave 60 3.635 0.000 67

23 154 Ave 70 1.634 0.000 24

23 154 Ave 80 0.000 0.000 9

23 154 Ave 90 0.000 0.000 4

23 154 Ave 100 0.000 0.000 1

23 154 cMEM 0 10.765 0.000 8002

23 154 cMEM 10 10.249 0.000 380

23 154 cMEM 20 10.064 0.000 189

23 154 cMEM 30 9.562 0.000 121

23 154 cMEM 40 8.900 0.000 84

23 154 cMEM 50 7.114 0.000 46

23 154 cMEM 60 2.959 0.000 22

23 154 cMEM 70 0.000 0.000 14

23 154 cMEM 80 0.000 0.000 4

23 154 cMEM 90 0.000 0.000 2

23 154 cMEM 100 0.000 0.000 1

23 154 dSPM 0 30.523 0.000 8002

23 154 dSPM 10 27.333 0.000 2575

23 154 dSPM 20 21.389 0.000 1142

23 154 dSPM 30 15.902 0.000 607

23 154 dSPM 40 11.076 0.000 339

23 154 dSPM 50 9.276 0.000 191

23 154 dSPM 60 8.895 0.000 120

23 154 dSPM 70 8.271 0.000 75

23 154 dSPM 80 9.150 0.000 32

23 154 dSPM 90 9.249 0.000 14

23 154 dSPM 100 13.134 13.134 1

23 154 MNE 0 29.060 0.000 8002

23 154 MNE 10 20.520 0.000 848

23 154 MNE 20 12.458 0.000 312

23 154 MNE 30 6.689 0.000 131

23 154 MNE 40 5.118 0.000 67

23 154 MNE 50 1.868 0.000 32

23 154 MNE 60 1.597 0.000 19

23 154 MNE 70 1.842 0.000 7

23 154 MNE 80 2.249 0.000 4

23 154 MNE 90 0.000 0.000 1

23 154 MNE 100 0.000 0.000 1

23 154 sLORETA 0 27.350 0.000 8002

23 154 sLORETA 10 22.694 0.000 1939

23 154 sLORETA 20 17.286 0.000 887

23 154 sLORETA 30 11.463 0.000 471

23 154 sLORETA 40 8.235 0.000 268

23 154 sLORETA 50 7.118 0.000 169

23 154 sLORETA 60 5.820 0.000 110

23 154 sLORETA 70 2.155 0.000 54

23 154 sLORETA 80 0.000 0.000 17

23 154 sLORETA 90 0.000 0.000 5

23 154 sLORETA 100 0.000 0.000 1

23 155 Ave 0 32.229 0.000 8002

23 155 Ave 10 27.827 0.000 2287

23 155 Ave 20 18.557 0.000 813

23 155 Ave 30 10.185 0.000 392

23 155 Ave 40 7.806 0.000 253

23 155 Ave 50 6.472 0.000 153

23 155 Ave 60 3.836 0.000 66

23 155 Ave 70 1.552 0.000 23

23 155 Ave 80 0.000 0.000 8

23 155 Ave 90 0.000 0.000 2

23 155 Ave 100 0.000 0.000 1

23 155 cMEM 0 11.677 0.000 8002

23 155 cMEM 10 7.773 0.000 458

23 155 cMEM 20 3.800 0.000 194

23 155 cMEM 30 3.718 0.000 137

23 155 cMEM 40 3.241 0.000 88

23 155 cMEM 50 2.643 0.000 51

23 155 cMEM 60 2.915 0.000 26

23 155 cMEM 70 3.299 0.000 11

23 155 cMEM 80 0.000 0.000 6

23 155 cMEM 90 0.000 0.000 3

23 155 cMEM 100 0.000 0.000 1

23 155 dSPM 0 36.714 0.000 8002

23 155 dSPM 10 34.729 0.000 2992

23 155 dSPM 20 30.691 0.000 1386

23 155 dSPM 30 24.904 0.000 698

23 155 dSPM 40 17.777 0.000 365

23 155 dSPM 50 11.750 0.000 198

23 155 dSPM 60 10.244 0.000 112

23 155 dSPM 70 10.683 0.000 57

23 155 dSPM 80 10.484 0.000 30

23 155 dSPM 90 9.938 5.138 9

23 155 dSPM 100 5.496 5.496 1

23 155 MNE 0 31.243 0.000 8002

23 155 MNE 10 19.122 0.000 678

23 155 MNE 20 11.448 0.000 229

23 155 MNE 30 4.999 0.000 91

23 155 MNE 40 2.713 0.000 33

23 155 MNE 50 1.932 0.000 19

23 155 MNE 60 1.938 0.000 10

23 155 MNE 70 2.143 0.000 4

23 155 MNE 80 0.000 0.000 1

23 155 MNE 90 0.000 0.000 1

23 155 MNE 100 0.000 0.000 1

23 155 sLORETA 0 32.088 0.000 8002

23 155 sLORETA 10 28.247 0.000 2447

23 155 sLORETA 20 20.851 0.000 940

23 155 sLORETA 30 13.544 0.000 462

23 155 sLORETA 40 10.624 0.000 286

23 155 sLORETA 50 8.587 0.000 181

23 155 sLORETA 60 7.402 0.000 110

23 155 sLORETA 70 6.067 0.000 53

23 155 sLORETA 80 0.000 0.000 13

23 155 sLORETA 90 0.000 0.000 5

23 155 sLORETA 100 0.000 0.000 1

23 156 Ave 0 33.571 0.000 8002

23 156 Ave 10 29.668 0.000 2685

23 156 Ave 20 18.481 0.000 915

23 156 Ave 30 10.703 0.000 450

23 156 Ave 40 7.831 0.000 257

23 156 Ave 50 6.547 0.000 145

23 156 Ave 60 4.672 0.000 89

23 156 Ave 70 1.554 0.000 43

23 156 Ave 80 1.171 0.000 15

23 156 Ave 90 0.000 0.000 3

23 156 Ave 100 0.000 0.000 1

23 156 cMEM 0 13.665 0.000 8002

23 156 cMEM 10 11.360 0.000 412

23 156 cMEM 20 10.496 0.000 210

23 156 cMEM 30 9.958 0.000 120

23 156 cMEM 40 9.177 0.000 52

23 156 cMEM 50 4.858 0.000 17

23 156 cMEM 60 0.000 0.000 10

23 156 cMEM 70 0.000 0.000 5

23 156 cMEM 80 0.000 0.000 3

23 156 cMEM 90 0.000 0.000 2

23 156 cMEM 100 0.000 0.000 1

23 156 dSPM 0 37.366 0.000 8002

23 156 dSPM 10 35.413 0.000 3411

23 156 dSPM 20 29.648 0.000 1506

23 156 dSPM 30 22.167 0.000 706

23 156 dSPM 40 15.036 0.000 381

23 156 dSPM 50 10.689 0.000 211

23 156 dSPM 60 10.024 0.000 130

23 156 dSPM 70 10.017 0.000 72

23 156 dSPM 80 9.002 0.000 27

23 156 dSPM 90 10.318 0.000 5

23 156 dSPM 100 11.843 11.843 1

23 156 MNE 0 36.121 0.000 8002

23 156 MNE 10 29.223 0.000 1128

23 156 MNE 20 14.941 0.000 311

23 156 MNE 30 5.449 0.000 138

23 156 MNE 40 2.871 0.000 65

23 156 MNE 50 2.556 0.000 38

23 156 MNE 60 2.313 0.000 21

23 156 MNE 70 1.940 0.000 14

23 156 MNE 80 2.366 0.000 7

23 156 MNE 90 0.000 0.000 3

23 156 MNE 100 0.000 0.000 1

23 156 sLORETA 0 32.350 0.000 8002

23 156 sLORETA 10 28.639 0.000 2623

23 156 sLORETA 20 20.371 0.000 1006

23 156 sLORETA 30 14.525 0.000 522

23 156 sLORETA 40 11.083 0.000 317

23 156 sLORETA 50 8.125 0.000 193

23 156 sLORETA 60 7.403 0.000 120

23 156 sLORETA 70 5.677 0.000 72

23 156 sLORETA 80 0.000 0.000 27

23 156 sLORETA 90 0.000 0.000 5

23 156 sLORETA 100 0.000 0.000 1

23 157 Ave 0 29.351 0.000 8002

23 157 Ave 10 23.916 0.000 2059

23 157 Ave 20 15.248 0.000 824

23 157 Ave 30 9.965 0.000 428

23 157 Ave 40 6.517 0.000 236

23 157 Ave 50 4.240 0.000 127

23 157 Ave 60 2.878 0.000 65

23 157 Ave 70 0.754 0.000 33

23 157 Ave 80 0.000 0.000 14

23 157 Ave 90 0.000 0.000 6

23 157 Ave 100 0.000 0.000 1

23 157 cMEM 0 10.522 0.000 8002

23 157 cMEM 10 9.404 0.000 386

23 157 cMEM 20 8.871 0.000 228

23 157 cMEM 30 7.832 0.000 135

23 157 cMEM 40 7.285 0.000 68

23 157 cMEM 50 6.807 0.000 30

23 157 cMEM 60 7.287 0.000 12

23 157 cMEM 70 5.506 0.000 8

23 157 cMEM 80 0.000 0.000 4

23 157 cMEM 90 0.000 0.000 2

23 157 cMEM 100 0.000 0.000 1

23 157 dSPM 0 33.223 0.000 8002

23 157 dSPM 10 30.755 0.000 2979

23 157 dSPM 20 25.545 0.000 1399

23 157 dSPM 30 19.136 0.000 757

23 157 dSPM 40 14.830 0.000 446

23 157 dSPM 50 11.452 0.000 251

23 157 dSPM 60 8.592 0.000 139

23 157 dSPM 70 8.476 0.000 79

23 157 dSPM 80 8.260 0.000 34

23 157 dSPM 90 7.770 0.000 9

23 157 dSPM 100 0.000 0.000 1

23 157 MNE 0 31.340 0.000 8002

23 157 MNE 10 22.145 0.000 872

23 157 MNE 20 12.855 0.000 304

23 157 MNE 30 7.150 0.000 122

23 157 MNE 40 3.392 0.000 56

23 157 MNE 50 1.569 0.000 31

23 157 MNE 60 1.486 0.000 15

23 157 MNE 70 0.000 0.000 6

23 157 MNE 80 0.000 0.000 4

23 157 MNE 90 0.000 0.000 3

23 157 MNE 100 0.000 0.000 1

23 157 sLORETA 0 28.686 0.000 8002

23 157 sLORETA 10 24.005 0.000 2142

23 157 sLORETA 20 17.467 0.000 923

23 157 sLORETA 30 13.572 0.000 543

23 157 sLORETA 40 10.274 0.000 308

23 157 sLORETA 50 7.401 0.000 179

23 157 sLORETA 60 5.032 0.000 97

23 157 sLORETA 70 0.962 0.000 49

23 157 sLORETA 80 0.000 0.000 20

23 157 sLORETA 90 0.000 0.000 6

23 157 sLORETA 100 0.000 0.000 1

23 158 Ave 0 31.818 0.000 8002

23 158 Ave 10 27.695 0.000 2660

23 158 Ave 20 17.642 0.000 982

23 158 Ave 30 11.398 0.000 510

23 158 Ave 40 6.577 0.000 275

23 158 Ave 50 4.683 0.000 173

23 158 Ave 60 4.050 0.000 99

23 158 Ave 70 2.097 0.000 43

23 158 Ave 80 1.081 0.000 19

23 158 Ave 90 0.000 0.000 5

23 158 Ave 100 0.000 0.000 1

23 158 cMEM 0 11.800 0.000 8002

23 158 cMEM 10 10.846 0.000 380

23 158 cMEM 20 10.456 0.000 234

23 158 cMEM 30 9.863 0.000 137

23 158 cMEM 40 8.162 0.000 71

23 158 cMEM 50 8.108 0.000 43

23 158 cMEM 60 8.654 0.000 24

23 158 cMEM 70 8.927 0.000 14

23 158 cMEM 80 8.395 0.000 8

23 158 cMEM 90 6.061 4.861 2

23 158 cMEM 100 6.949 6.949 1

23 158 dSPM 0 35.365 0.000 8002

23 158 dSPM 10 33.113 0.000 3293

23 158 dSPM 20 27.006 0.000 1421

23 158 dSPM 30 21.641 0.000 751

23 158 dSPM 40 15.893 0.000 400

23 158 dSPM 50 11.709 0.000 211

23 158 dSPM 60 9.102 0.000 129

23 158 dSPM 70 9.189 0.000 62

23 158 dSPM 80 8.414 0.000 26

23 158 dSPM 90 9.306 0.000 7

23 158 dSPM 100 11.843 11.843 1

23 158 MNE 0 35.672 0.000 8002

23 158 MNE 10 28.434 0.000 1175

23 158 MNE 20 16.358 0.000 361

23 158 MNE 30 10.038 0.000 160

23 158 MNE 40 6.106 0.000 78

23 158 MNE 50 2.298 0.000 35

23 158 MNE 60 1.541 0.000 15

23 158 MNE 70 0.000 0.000 6

23 158 MNE 80 0.000 0.000 4

23 158 MNE 90 0.000 0.000 1

23 158 MNE 100 0.000 0.000 1

23 158 sLORETA 0 30.327 0.000 8002

23 158 sLORETA 10 26.375 0.000 2602

23 158 sLORETA 20 17.298 0.000 1001

23 158 sLORETA 30 12.946 0.000 582

23 158 sLORETA 40 8.560 0.000 318

23 158 sLORETA 50 5.912 0.000 198

23 158 sLORETA 60 4.585 0.000 119

23 158 sLORETA 70 3.033 0.000 63

23 158 sLORETA 80 1.336 0.000 24

23 158 sLORETA 90 0.000 0.000 8

23 158 sLORETA 100 0.000 0.000 1

24 159 Ave 0 23.143 0.000 8009

24 159 Ave 10 22.759 0.000 5085

24 159 Ave 20 21.453 0.000 3126

24 159 Ave 30 19.843 0.000 1897

24 159 Ave 40 17.805 0.000 1056

24 159 Ave 50 15.631 0.000 519

24 159 Ave 60 14.027 0.000 220

24 159 Ave 70 14.517 0.000 114

24 159 Ave 80 17.089 0.000 39

24 159 Ave 90 14.213 7.763 7

24 159 Ave 100 18.215 18.215 1

24 159 cMEM 0 23.190 0.000 8009

24 159 cMEM 10 23.387 0.000 704

24 159 cMEM 20 24.175 0.000 327

24 159 cMEM 30 25.960 0.000 152

24 159 cMEM 40 27.688 0.000 73

24 159 cMEM 50 27.986 3.280 36

24 159 cMEM 60 30.172 4.926 21

24 159 cMEM 70 33.371 21.826 9

24 159 cMEM 80 31.211 24.980 3

24 159 cMEM 90 31.211 24.980 3

24 159 cMEM 100 37.964 37.964 1

24 159 dSPM 0 20.785 0.000 8009

24 159 dSPM 10 20.148 0.000 4401

24 159 dSPM 20 18.512 0.000 2493

24 159 dSPM 30 16.782 0.000 1434

24 159 dSPM 40 15.181 0.000 809

24 159 dSPM 50 12.238 0.000 394

24 159 dSPM 60 9.323 0.000 198

24 159 dSPM 70 9.404 0.000 91

24 159 dSPM 80 8.780 0.000 29

24 159 dSPM 90 10.663 0.000 9

24 159 dSPM 100 15.291 15.291 1

24 159 MNE 0 27.440 0.000 8009

24 159 MNE 10 26.893 0.000 3092

24 159 MNE 20 25.888 0.000 1300

24 159 MNE 30 25.220 0.000 565

24 159 MNE 40 24.290 0.000 224

24 159 MNE 50 24.333 0.000 90

24 159 MNE 60 24.117 0.000 44

24 159 MNE 70 24.157 0.000 24

24 159 MNE 80 27.970 7.763 9

24 159 MNE 90 10.612 7.763 2

24 159 MNE 100 12.572 12.572 1

24 159 sLORETA 0 22.633 0.000 8009

24 159 sLORETA 10 22.277 0.000 5136

24 159 sLORETA 20 21.057 0.000 3282

24 159 sLORETA 30 19.356 0.000 1996

24 159 sLORETA 40 17.355 0.000 1181

24 159 sLORETA 50 15.064 0.000 659

24 159 sLORETA 60 13.287 0.000 327

24 159 sLORETA 70 12.011 0.000 150

24 159 sLORETA 80 8.874 0.000 55

24 159 sLORETA 90 7.811 0.000 16

24 159 sLORETA 100 9.958 9.958 1

24 160 Ave 0 18.025 0.000 8009

24 160 Ave 10 17.334 0.000 4331

24 160 Ave 20 14.835 0.000 2413

24 160 Ave 30 12.343 0.000 1485

24 160 Ave 40 10.087 0.000 995

24 160 Ave 50 8.604 0.000 654

24 160 Ave 60 7.737 0.000 383

24 160 Ave 70 7.170 0.000 207

24 160 Ave 80 6.163 0.000 79

24 160 Ave 90 5.166 0.000 11

24 160 Ave 100 0.000 0.000 1

24 160 cMEM 0 8.009 0.000 8009

24 160 cMEM 10 7.303 0.000 439

24 160 cMEM 20 5.674 0.000 169

24 160 cMEM 30 2.745 0.000 81

24 160 cMEM 40 1.304 0.000 44

24 160 cMEM 50 0.000 0.000 36

24 160 cMEM 60 0.000 0.000 21

24 160 cMEM 70 0.000 0.000 12

24 160 cMEM 80 0.000 0.000 9

24 160 cMEM 90 0.000 0.000 5

24 160 cMEM 100 0.000 0.000 1

24 160 dSPM 0 15.926 0.000 8009

24 160 dSPM 10 14.090 0.000 3144

24 160 dSPM 20 10.652 0.000 1475

24 160 dSPM 30 9.007 0.000 787

24 160 dSPM 40 7.992 0.000 439

24 160 dSPM 50 7.260 0.000 229

24 160 dSPM 60 7.342 0.000 102

24 160 dSPM 70 6.271 0.000 45

24 160 dSPM 80 6.494 0.000 20

24 160 dSPM 90 3.454 0.000 5

24 160 dSPM 100 0.000 0.000 1

24 160 MNE 0 22.031 0.000 8009

24 160 MNE 10 21.022 0.000 2988

24 160 MNE 20 18.444 0.000 1430

24 160 MNE 30 16.542 0.000 736

24 160 MNE 40 14.424 0.000 379

24 160 MNE 50 12.109 0.000 176

24 160 MNE 60 9.161 0.000 67

24 160 MNE 70 8.616 0.000 26

24 160 MNE 80 4.689 0.000 5

24 160 MNE 90 0.000 0.000 1

24 160 MNE 100 0.000 0.000 1

24 160 sLORETA 0 16.852 0.000 8009

24 160 sLORETA 10 16.023 0.000 4084

24 160 sLORETA 20 13.327 0.000 2229

24 160 sLORETA 30 10.541 0.000 1389

24 160 sLORETA 40 9.072 0.000 930

24 160 sLORETA 50 8.084 0.000 604

24 160 sLORETA 60 7.401 0.000 345

24 160 sLORETA 70 6.237 0.000 192

24 160 sLORETA 80 5.018 0.000 73

24 160 sLORETA 90 2.134 0.000 25

24 160 sLORETA 100 0.000 0.000 1

24 161 Ave 0 16.034 0.000 8009

24 161 Ave 10 14.696 0.000 3026

24 161 Ave 20 12.404 0.000 1477

24 161 Ave 30 10.182 0.000 839

24 161 Ave 40 7.788 0.000 467

24 161 Ave 50 6.356 0.000 278

24 161 Ave 60 4.532 0.000 146

24 161 Ave 70 3.286 0.000 71

24 161 Ave 80 0.997 0.000 25

24 161 Ave 90 0.000 0.000 3

24 161 Ave 100 0.000 0.000 1

24 161 cMEM 0 6.859 0.000 8009

24 161 cMEM 10 6.156 0.000 197

24 161 cMEM 20 4.726 0.000 96

24 161 cMEM 30 3.486 0.000 39

24 161 cMEM 40 2.199 0.000 23

24 161 cMEM 50 0.818 0.000 12

24 161 cMEM 60 0.000 0.000 7

24 161 cMEM 70 0.000 0.000 4

24 161 cMEM 80 0.000 0.000 2

24 161 cMEM 90 0.000 0.000 1

24 161 cMEM 100 0.000 0.000 1

24 161 dSPM 0 15.821 0.000 8009

24 161 dSPM 10 14.906 0.000 3492

24 161 dSPM 20 12.944 0.000 1877

24 161 dSPM 30 10.806 0.000 1172

24 161 dSPM 40 8.702 0.000 730

24 161 dSPM 50 6.840 0.000 429

24 161 dSPM 60 5.719 0.000 267

24 161 dSPM 70 4.121 0.000 134

24 161 dSPM 80 2.901 0.000 59

24 161 dSPM 90 2.172 0.000 18

24 161 dSPM 100 0.000 0.000 1

24 161 MNE 0 18.932 0.000 8009

24 161 MNE 10 16.169 0.000 1146

24 161 MNE 20 12.599 0.000 327

24 161 MNE 30 10.197 0.000 120

24 161 MNE 40 10.190 0.000 46

24 161 MNE 50 0.000 0.000 13

24 161 MNE 60 0.000 0.000 6

24 161 MNE 70 0.000 0.000 3

24 161 MNE 80 0.000 0.000 1

24 161 MNE 90 0.000 0.000 1

24 161 MNE 100 0.000 0.000 1

24 161 sLORETA 0 15.374 0.000 8009

24 161 sLORETA 10 13.779 0.000 2532

24 161 sLORETA 20 11.774 0.000 1238

24 161 sLORETA 30 9.740 0.000 710

24 161 sLORETA 40 7.752 0.000 431

24 161 sLORETA 50 6.766 0.000 263

24 161 sLORETA 60 6.085 0.000 140

24 161 sLORETA 70 5.703 0.000 67

24 161 sLORETA 80 2.595 0.000 27

24 161 sLORETA 90 0.000 0.000 6

24 161 sLORETA 100 0.000 0.000 1

24 162 Ave 0 13.650 0.000 8009

24 162 Ave 10 11.233 0.000 2974

24 162 Ave 20 6.706 0.000 1371

24 162 Ave 30 3.725 0.000 780

24 162 Ave 40 2.131 0.000 473

24 162 Ave 50 0.908 0.000 296

24 162 Ave 60 0.428 0.000 163

24 162 Ave 70 0.466 0.000 62

24 162 Ave 80 0.000 0.000 26

24 162 Ave 90 0.000 0.000 10

24 162 Ave 100 0.000 0.000 1

24 162 cMEM 0 3.677 0.000 8009

24 162 cMEM 10 2.566 0.000 468

24 162 cMEM 20 2.096 0.000 206

24 162 cMEM 30 0.802 0.000 89

24 162 cMEM 40 0.329 0.000 62

24 162 cMEM 50 0.000 0.000 42

24 162 cMEM 60 0.000 0.000 24

24 162 cMEM 70 0.000 0.000 14

24 162 cMEM 80 0.000 0.000 7

24 162 cMEM 90 0.000 0.000 2

24 162 cMEM 100 0.000 0.000 1

24 162 dSPM 0 13.267 0.000 8009

24 162 dSPM 10 10.872 0.000 2826

24 162 dSPM 20 7.248 0.000 1305

24 162 dSPM 30 5.231 0.000 763

24 162 dSPM 40 3.935 0.000 441

24 162 dSPM 50 2.837 0.000 254

24 162 dSPM 60 1.496 0.000 138

24 162 dSPM 70 0.563 0.000 59

24 162 dSPM 80 0.430 0.000 22

24 162 dSPM 90 0.000 0.000 2

24 162 dSPM 100 0.000 0.000 1

24 162 MNE 0 16.375 0.000 8009

24 162 MNE 10 12.517 0.000 1664

24 162 MNE 20 7.559 0.000 618

24 162 MNE 30 2.926 0.000 266

24 162 MNE 40 2.207 0.000 132

24 162 MNE 50 0.360 0.000 59

24 162 MNE 60 0.000 0.000 23

24 162 MNE 70 0.000 0.000 13

24 162 MNE 80 0.000 0.000 4

24 162 MNE 90 0.000 0.000 3

24 162 MNE 100 0.000 0.000 1

24 162 sLORETA 0 12.932 0.000 8009

24 162 sLORETA 10 10.694 0.000 2999

24 162 sLORETA 20 6.901 0.000 1442

24 162 sLORETA 30 3.829 0.000 877

24 162 sLORETA 40 2.007 0.000 556

24 162 sLORETA 50 1.086 0.000 349

24 162 sLORETA 60 0.583 0.000 208

24 162 sLORETA 70 0.349 0.000 118

24 162 sLORETA 80 0.000 0.000 60

24 162 sLORETA 90 0.000 0.000 23

24 162 sLORETA 100 0.000 0.000 1

24 163 Ave 0 16.462 0.000 8009

24 163 Ave 10 15.359 0.000 3317

24 163 Ave 20 13.469 0.000 1839

24 163 Ave 30 11.607 0.000 1142

24 163 Ave 40 10.247 0.000 711

24 163 Ave 50 8.928 0.000 425

24 163 Ave 60 7.780 0.000 269

24 163 Ave 70 6.768 0.000 160

24 163 Ave 80 6.690 0.000 64

24 163 Ave 90 2.322 0.000 17

24 163 Ave 100 0.000 0.000 1

24 163 cMEM 0 5.231 0.000 8009

24 163 cMEM 10 4.380 0.000 355

24 163 cMEM 20 4.063 0.000 135

24 163 cMEM 30 4.255 0.000 73

24 163 cMEM 40 4.725 0.000 41

24 163 cMEM 50 5.136 0.000 24

24 163 cMEM 60 5.127 0.000 13

24 163 cMEM 70 4.784 0.000 10

24 163 cMEM 80 4.902 0.000 7

24 163 cMEM 90 4.605 4.605 1

24 163 cMEM 100 4.605 4.605 1

24 163 dSPM 0 15.734 0.000 8009

24 163 dSPM 10 14.592 0.000 3203

24 163 dSPM 20 12.541 0.000 1735

24 163 dSPM 30 10.422 0.000 1066

24 163 dSPM 40 8.825 0.000 640

24 163 dSPM 50 7.795 0.000 370

24 163 dSPM 60 6.410 0.000 216

24 163 dSPM 70 5.146 0.000 111

24 163 dSPM 80 2.199 0.000 30

24 163 dSPM 90 0.000 0.000 9

24 163 dSPM 100 0.000 0.000 1

24 163 MNE 0 19.324 0.000 8009

24 163 MNE 10 16.765 0.000 1559

24 163 MNE 20 14.286 0.000 563

24 163 MNE 30 12.268 0.000 221

24 163 MNE 40 11.336 0.000 87

24 163 MNE 50 11.077 0.000 26

24 163 MNE 60 0.000 0.000 6

24 163 MNE 70 0.000 0.000 1

24 163 MNE 80 0.000 0.000 1

24 163 MNE 90 0.000 0.000 1

24 163 MNE 100 0.000 0.000 1

24 163 sLORETA 0 16.397 0.000 8009

24 163 sLORETA 10 15.016 0.000 2753

24 163 sLORETA 20 13.176 0.000 1496

24 163 sLORETA 30 11.272 0.000 863

24 163 sLORETA 40 9.800 0.000 503

24 163 sLORETA 50 9.723 0.000 296

24 163 sLORETA 60 9.251 0.000 162

24 163 sLORETA 70 9.059 0.000 77

24 163 sLORETA 80 7.136 0.000 26

24 163 sLORETA 90 2.795 0.000 7

24 163 sLORETA 100 0.000 0.000 1

24 164 Ave 0 18.302 0.000 8009

24 164 Ave 10 17.421 0.000 4194

24 164 Ave 20 14.378 0.000 2118

24 164 Ave 30 12.026 0.000 1265

24 164 Ave 40 8.712 0.000 735

24 164 Ave 50 5.903 0.000 442

24 164 Ave 60 2.040 0.000 235

24 164 Ave 70 0.717 0.000 126

24 164 Ave 80 0.290 0.000 47

24 164 Ave 90 0.000 0.000 12

24 164 Ave 100 0.000 0.000 1

24 164 cMEM 0 4.560 0.000 8009

24 164 cMEM 10 1.886 0.000 235

24 164 cMEM 20 0.969 0.000 51

24 164 cMEM 30 0.403 0.000 22

24 164 cMEM 40 0.000 0.000 11

24 164 cMEM 50 0.000 0.000 10

24 164 cMEM 60 0.000 0.000 9

24 164 cMEM 70 0.000 0.000 7

24 164 cMEM 80 0.000 0.000 7

24 164 cMEM 90 0.000 0.000 3

24 164 cMEM 100 0.000 0.000 1

24 164 dSPM 0 17.084 0.000 8009

24 164 dSPM 10 15.344 0.000 3120

24 164 dSPM 20 12.332 0.000 1518

24 164 dSPM 30 9.123 0.000 734

24 164 dSPM 40 6.563 0.000 376

24 164 dSPM 50 4.436 0.000 176

24 164 dSPM 60 3.275 0.000 80

24 164 dSPM 70 2.131 0.000 37

24 164 dSPM 80 0.557 0.000 14

24 164 dSPM 90 0.000 0.000 3

24 164 dSPM 100 0.000 0.000 1

24 164 MNE 0 22.537 0.000 8009

24 164 MNE 10 21.146 0.000 2670

24 164 MNE 20 18.014 0.000 1157

24 164 MNE 30 16.172 0.000 494

24 164 MNE 40 14.029 0.000 214

24 164 MNE 50 14.222 0.000 97

24 164 MNE 60 14.245 0.000 42

24 164 MNE 70 0.000 0.000 21

24 164 MNE 80 0.000 0.000 9

24 164 MNE 90 0.000 0.000 3

24 164 MNE 100 0.000 0.000 1

24 164 sLORETA 0 17.182 0.000 8009

24 164 sLORETA 10 16.239 0.000 4094

24 164 sLORETA 20 13.553 0.000 2130

24 164 sLORETA 30 11.554 0.000 1324

24 164 sLORETA 40 8.116 0.000 846

24 164 sLORETA 50 5.327 0.000 515

24 164 sLORETA 60 3.841 0.000 301

24 164 sLORETA 70 0.885 0.000 170

24 164 sLORETA 80 0.000 0.000 71

24 164 sLORETA 90 0.000 0.000 19

24 164 sLORETA 100 0.000 0.000 1

24 165 Ave 0 14.300 0.000 8009

24 165 Ave 10 11.795 0.000 2223

24 165 Ave 20 9.777 0.000 1048

24 165 Ave 30 7.612 0.000 559

24 165 Ave 40 6.077 0.000 321

24 165 Ave 50 4.935 0.000 206

24 165 Ave 60 4.247 0.000 117

24 165 Ave 70 3.954 0.000 60

24 165 Ave 80 3.855 0.000 28

24 165 Ave 90 3.871 0.000 14

24 165 Ave 100 0.000 0.000 1

24 165 cMEM 0 12.387 0.000 8009

24 165 cMEM 10 11.018 0.000 196

24 165 cMEM 20 11.031 0.000 115

24 165 cMEM 30 10.669 0.000 69

24 165 cMEM 40 10.013 0.000 34

24 165 cMEM 50 8.387 4.493 12

24 165 cMEM 60 8.289 4.493 9

24 165 cMEM 70 8.059 4.493 6

24 165 cMEM 80 8.094 4.493 5

24 165 cMEM 90 9.178 7.537 3

24 165 cMEM 100 10.706 10.706 1

24 165 dSPM 0 14.692 0.000 8009

24 165 dSPM 10 12.585 0.000 2324

24 165 dSPM 20 10.926 0.000 1145

24 165 dSPM 30 9.269 0.000 612

24 165 dSPM 40 7.661 0.000 330

24 165 dSPM 50 6.472 0.000 173

24 165 dSPM 60 5.305 0.000 82

24 165 dSPM 70 5.103 0.000 30

24 165 dSPM 80 4.704 0.000 8

24 165 dSPM 90 5.102 0.000 6

24 165 dSPM 100 4.446 4.446 1

24 165 MNE 0 15.825 0.000 8009

24 165 MNE 10 11.708 0.000 994

24 165 MNE 20 9.285 0.000 361

24 165 MNE 30 6.782 0.000 171

24 165 MNE 40 5.392 0.000 90

24 165 MNE 50 4.312 0.000 52

24 165 MNE 60 2.545 0.000 33

24 165 MNE 70 2.376 0.000 12

24 165 MNE 80 2.575 0.000 6

24 165 MNE 90 1.973 0.000 3

24 165 MNE 100 3.319 3.319 1

24 165 sLORETA 0 13.366 0.000 8009

24 165 sLORETA 10 10.907 0.000 2077

24 165 sLORETA 20 8.975 0.000 980

24 165 sLORETA 30 7.043 0.000 563

24 165 sLORETA 40 5.900 0.000 335

24 165 sLORETA 50 4.613 0.000 225

24 165 sLORETA 60 4.019 0.000 138

24 165 sLORETA 70 3.902 0.000 73

24 165 sLORETA 80 3.758 0.000 28

24 165 sLORETA 90 4.034 0.000 10

24 165 sLORETA 100 8.815 8.815 1

24 166 Ave 0 17.873 0.000 8009

24 166 Ave 10 15.648 0.000 2772

24 166 Ave 20 12.200 0.000 1150

24 166 Ave 30 10.484 0.000 481

24 166 Ave 40 9.842 0.000 229

24 166 Ave 50 9.301 0.000 120

24 166 Ave 60 9.536 0.000 61

24 166 Ave 70 8.759 0.000 39

24 166 Ave 80 7.555 0.000 20

24 166 Ave 90 6.286 0.000 4

24 166 Ave 100 2.170 2.170 1

24 166 cMEM 0 13.983 0.000 8009

24 166 cMEM 10 12.674 0.000 293

24 166 cMEM 20 11.615 0.000 111

24 166 cMEM 30 10.658 0.000 70

24 166 cMEM 40 10.280 0.000 47

24 166 cMEM 50 8.484 0.000 28

24 166 cMEM 60 7.505 0.000 13

24 166 cMEM 70 8.065 0.000 6

24 166 cMEM 80 9.869 8.711 2

24 166 cMEM 90 10.706 10.706 1

24 166 cMEM 100 10.706 10.706 1

24 166 dSPM 0 17.754 0.000 8009

24 166 dSPM 10 15.965 0.000 2816

24 166 dSPM 20 13.400 0.000 1229

24 166 dSPM 30 11.745 0.000 538

24 166 dSPM 40 11.081 0.000 241

24 166 dSPM 50 10.669 0.000 127

24 166 dSPM 60 10.160 0.000 60

24 166 dSPM 70 10.026 0.000 32

24 166 dSPM 80 10.268 0.000 20

24 166 dSPM 90 12.834 5.474 6

24 166 dSPM 100 14.147 14.147 1

24 166 MNE 0 19.477 0.000 8009

24 166 MNE 10 15.950 0.000 1274

24 166 MNE 20 12.417 0.000 386

24 166 MNE 30 11.383 0.000 166

24 166 MNE 40 9.681 0.000 75

24 166 MNE 50 9.274 0.000 40

24 166 MNE 60 9.840 0.000 22

24 166 MNE 70 5.116 0.000 10

24 166 MNE 80 3.703 0.000 7

24 166 MNE 90 1.471 0.000 2

24 166 MNE 100 0.000 0.000 1

24 166 sLORETA 0 17.168 0.000 8009

24 166 sLORETA 10 14.969 0.000 2841

24 166 sLORETA 20 11.726 0.000 1239

24 166 sLORETA 30 9.733 0.000 575

24 166 sLORETA 40 8.621 0.000 284

24 166 sLORETA 50 8.352 0.000 153

24 166 sLORETA 60 8.191 0.000 76

24 166 sLORETA 70 7.750 0.000 43

24 166 sLORETA 80 6.544 0.000 25

24 166 sLORETA 90 5.395 0.000 10

24 166 sLORETA 100 8.815 8.815 1

24 167 Ave 0 17.349 0.000 8009

24 167 Ave 10 14.989 0.000 2494

24 167 Ave 20 11.185 0.000 989

24 167 Ave 30 9.854 0.000 469

24 167 Ave 40 8.884 0.000 224

24 167 Ave 50 8.456 0.000 129

24 167 Ave 60 8.857 0.000 83

24 167 Ave 70 9.296 0.000 44

24 167 Ave 80 7.739 0.000 20

24 167 Ave 90 5.935 0.000 5

24 167 Ave 100 2.170 2.170 1

24 167 cMEM 0 18.114 0.000 8009

24 167 cMEM 10 17.192 0.000 342

24 167 cMEM 20 15.709 0.000 122

24 167 cMEM 30 13.636 0.000 54

24 167 cMEM 40 12.855 0.000 27

24 167 cMEM 50 10.079 0.000 14

24 167 cMEM 60 8.596 4.493 8

24 167 cMEM 70 8.292 4.493 5

24 167 cMEM 80 9.250 7.537 3

24 167 cMEM 90 9.860 8.711 2

24 167 cMEM 100 10.706 10.706 1

24 167 dSPM 0 16.951 0.000 8009

24 167 dSPM 10 14.763 0.000 2588

24 167 dSPM 20 11.610 0.000 1060

24 167 dSPM 30 10.729 0.000 497

24 167 dSPM 40 10.140 0.000 238

24 167 dSPM 50 9.827 0.000 137

24 167 dSPM 60 9.469 0.000 72

24 167 dSPM 70 9.883 0.000 39

24 167 dSPM 80 10.659 0.000 19

24 167 dSPM 90 12.574 7.446 7

24 167 dSPM 100 10.274 10.274 1

24 167 MNE 0 19.356 0.000 8009

24 167 MNE 10 16.422 0.000 1191

24 167 MNE 20 11.858 0.000 363

24 167 MNE 30 9.410 0.000 178

24 167 MNE 40 8.560 0.000 85

24 167 MNE 50 7.954 0.000 50

24 167 MNE 60 8.567 0.000 25

24 167 MNE 70 5.433 0.000 13

24 167 MNE 80 4.447 0.000 6

24 167 MNE 90 4.541 0.000 3

24 167 MNE 100 0.000 0.000 1

24 167 sLORETA 0 16.478 0.000 8009

24 167 sLORETA 10 14.041 0.000 2482

24 167 sLORETA 20 10.291 0.000 1024

24 167 sLORETA 30 8.720 0.000 503

24 167 sLORETA 40 7.672 0.000 255

24 167 sLORETA 50 7.763 0.000 143

24 167 sLORETA 60 8.212 0.000 93

24 167 sLORETA 70 6.507 0.000 48

24 167 sLORETA 80 5.501 0.000 25

24 167 sLORETA 90 2.914 0.000 5

24 167 sLORETA 100 4.820 4.820 1

24 168 Ave 0 18.425 0.000 8009

24 168 Ave 10 16.199 0.000 2833

24 168 Ave 20 13.087 0.000 1206

24 168 Ave 30 11.810 0.000 556

24 168 Ave 40 10.780 0.000 253

24 168 Ave 50 11.416 0.000 115

24 168 Ave 60 11.943 0.000 71

24 168 Ave 70 12.319 0.000 44

24 168 Ave 80 14.032 0.000 18

24 168 Ave 90 15.676 2.170 7

24 168 Ave 100 2.170 2.170 1

24 168 cMEM 0 19.624 0.000 8009

24 168 cMEM 10 19.591 0.000 280

24 168 cMEM 20 18.170 5.311 108

24 168 cMEM 30 16.712 5.311 53

24 168 cMEM 40 15.326 5.311 33

24 168 cMEM 50 14.379 5.311 21

24 168 cMEM 60 14.610 6.424 15

24 168 cMEM 70 13.741 6.424 11

24 168 cMEM 80 14.447 13.605 4

24 168 cMEM 90 14.283 13.605 3

24 168 cMEM 100 13.847 13.847 1

24 168 dSPM 0 17.498 0.000 8009

24 168 dSPM 10 14.761 0.000 2565

24 168 dSPM 20 12.271 0.000 1022

24 168 dSPM 30 10.877 0.000 383

24 168 dSPM 40 11.024 0.000 158

24 168 dSPM 50 10.919 0.000 86

24 168 dSPM 60 11.353 0.000 50

24 168 dSPM 70 11.757 2.612 25

24 168 dSPM 80 11.713 2.612 13

24 168 dSPM 90 12.423 7.446 7

24 168 dSPM 100 11.292 11.292 1

24 168 MNE 0 20.888 0.000 8009

24 168 MNE 10 18.199 0.000 1569

24 168 MNE 20 15.207 0.000 497

24 168 MNE 30 13.655 0.000 210

24 168 MNE 40 13.087 0.000 106

24 168 MNE 50 12.216 0.000 52

24 168 MNE 60 12.940 0.000 21

24 168 MNE 70 13.016 0.000 16

24 168 MNE 80 12.117 0.000 8

24 168 MNE 90 10.067 0.000 6

24 168 MNE 100 7.537 7.537 1

24 168 sLORETA 0 17.602 0.000 8009

24 168 sLORETA 10 15.598 0.000 2941

24 168 sLORETA 20 12.408 0.000 1344

24 168 sLORETA 30 11.169 0.000 707

24 168 sLORETA 40 9.850 0.000 349

24 168 sLORETA 50 9.759 0.000 169

24 168 sLORETA 60 10.849 0.000 84

24 168 sLORETA 70 11.125 0.000 56

24 168 sLORETA 80 12.180 0.000 25

24 168 sLORETA 90 11.416 2.170 4

24 168 sLORETA 100 2.170 2.170 1

25 169 Ave 0 23.193 0.000 8003

25 169 Ave 10 15.510 0.000 1521

25 169 Ave 20 9.368 0.000 513

25 169 Ave 30 7.683 0.000 261

25 169 Ave 40 6.911 0.000 154

25 169 Ave 50 6.293 0.000 93

25 169 Ave 60 5.676 0.000 54

25 169 Ave 70 5.572 0.000 27

25 169 Ave 80 4.570 0.000 11

25 169 Ave 90 4.651 0.000 4

25 169 Ave 100 3.631 3.631 1

25 169 cMEM 0 8.097 0.000 8003

25 169 cMEM 10 7.705 0.000 218

25 169 cMEM 20 7.494 0.000 132

25 169 cMEM 30 7.296 0.000 100

25 169 cMEM 40 7.344 0.000 83

25 169 cMEM 50 7.314 0.000 61

25 169 cMEM 60 7.295 0.000 42

25 169 cMEM 70 6.517 0.000 20

25 169 cMEM 80 5.900 3.631 8

25 169 cMEM 90 5.658 4.930 4

25 169 cMEM 100 6.260 6.260 1

25 169 dSPM 0 24.973 0.000 8003

25 169 dSPM 10 19.695 0.000 2074

25 169 dSPM 20 14.674 0.000 814

25 169 dSPM 30 11.999 0.000 383

25 169 dSPM 40 11.021 0.000 221

25 169 dSPM 50 9.516 0.000 134

25 169 dSPM 60 9.082 0.000 82

25 169 dSPM 70 8.762 0.000 51

25 169 dSPM 80 8.807 0.000 28

25 169 dSPM 90 7.926 4.138 12

25 169 dSPM 100 4.553 4.553 1

25 169 MNE 0 26.519 0.000 8003

25 169 MNE 10 20.235 0.000 1251

25 169 MNE 20 11.464 0.000 428

25 169 MNE 30 7.559 0.000 209

25 169 MNE 40 6.156 0.000 104

25 169 MNE 50 5.574 0.000 59

25 169 MNE 60 5.092 0.000 29

25 169 MNE 70 4.464 0.000 15

25 169 MNE 80 2.405 0.000 6

25 169 MNE 90 2.129 0.000 3

25 169 MNE 100 0.000 0.000 1

25 169 sLORETA 0 24.799 0.000 8003

25 169 sLORETA 10 19.405 0.000 2116

25 169 sLORETA 20 12.564 0.000 809

25 169 sLORETA 30 8.696 0.000 375

25 169 sLORETA 40 6.838 0.000 215

25 169 sLORETA 50 6.036 0.000 135

25 169 sLORETA 60 5.610 0.000 85

25 169 sLORETA 70 4.007 0.000 42

25 169 sLORETA 80 3.375 0.000 21

25 169 sLORETA 90 1.333 0.000 7

25 169 sLORETA 100 0.000 0.000 1

25 170 Ave 0 22.229 0.000 8003

25 170 Ave 10 14.775 0.000 1347

25 170 Ave 20 9.161 0.000 494

25 170 Ave 30 7.839 0.000 268

25 170 Ave 40 7.014 0.000 157

25 170 Ave 50 5.793 0.000 91

25 170 Ave 60 5.107 0.000 63

25 170 Ave 70 4.510 0.000 40

25 170 Ave 80 3.914 0.000 21

25 170 Ave 90 3.762 0.000 7

25 170 Ave 100 3.631 3.631 1

25 170 cMEM 0 8.237 0.000 8003

25 170 cMEM 10 7.987 0.000 180

25 170 cMEM 20 7.892 0.000 146

25 170 cMEM 30 7.698 0.000 124

25 170 cMEM 40 7.328 0.000 96

25 170 cMEM 50 6.311 0.000 56

25 170 cMEM 60 5.955 0.000 40

25 170 cMEM 70 5.171 0.000 22

25 170 cMEM 80 5.630 0.000 9

25 170 cMEM 90 5.665 4.930 4

25 170 cMEM 100 6.260 6.260 1

25 170 dSPM 0 24.362 0.000 8003

25 170 dSPM 10 18.882 0.000 1895

25 170 dSPM 20 13.045 0.000 730

25 170 dSPM 30 11.494 0.000 370

25 170 dSPM 40 10.206 0.000 202

25 170 dSPM 50 8.896 0.000 125

25 170 dSPM 60 8.642 0.000 83

25 170 dSPM 70 8.511 0.000 44

25 170 dSPM 80 8.219 0.000 25

25 170 dSPM 90 6.599 2.775 8

25 170 dSPM 100 6.440 6.440 1

25 170 MNE 0 26.157 0.000 8003

25 170 MNE 10 21.020 0.000 1190

25 170 MNE 20 13.625 0.000 430

25 170 MNE 30 9.551 0.000 223

25 170 MNE 40 7.339 0.000 121

25 170 MNE 50 6.698 0.000 69

25 170 MNE 60 6.189 0.000 42

25 170 MNE 70 5.959 0.000 22

25 170 MNE 80 3.291 0.000 11

25 170 MNE 90 1.817 0.000 4

25 170 MNE 100 0.000 0.000 1

25 170 sLORETA 0 23.080 0.000 8003

25 170 sLORETA 10 16.868 0.000 1728

25 170 sLORETA 20 9.937 0.000 652

25 170 sLORETA 30 7.738 0.000 342

25 170 sLORETA 40 6.698 0.000 199

25 170 sLORETA 50 5.670 0.000 119

25 170 sLORETA 60 4.343 0.000 64

25 170 sLORETA 70 3.362 0.000 35

25 170 sLORETA 80 2.296 0.000 20

25 170 sLORETA 90 0.000 0.000 4

25 170 sLORETA 100 0.000 0.000 1

25 171 Ave 0 23.699 0.000 8003

25 171 Ave 10 15.155 0.000 1386

25 171 Ave 20 6.970 0.000 434

25 171 Ave 30 3.694 0.000 187

25 171 Ave 40 2.667 0.000 99

25 171 Ave 50 0.898 0.000 50

25 171 Ave 60 0.640 0.000 30

25 171 Ave 70 0.000 0.000 12

25 171 Ave 80 0.000 0.000 4

25 171 Ave 90 0.000 0.000 1

25 171 Ave 100 0.000 0.000 1

25 171 cMEM 0 5.799 0.000 8003

25 171 cMEM 10 3.841 0.000 234

25 171 cMEM 20 2.179 0.000 131

25 171 cMEM 30 1.417 0.000 71

25 171 cMEM 40 0.000 0.000 38

25 171 cMEM 50 0.000 0.000 23

25 171 cMEM 60 0.000 0.000 18

25 171 cMEM 70 0.000 0.000 9

25 171 cMEM 80 0.000 0.000 5

25 171 cMEM 90 0.000 0.000 2

25 171 cMEM 100 0.000 0.000 1

25 171 dSPM 0 25.531 0.000 8003

25 171 dSPM 10 20.700 0.000 2372

25 171 dSPM 20 13.841 0.000 877

25 171 dSPM 30 9.792 0.000 388

25 171 dSPM 40 7.316 0.000 193

25 171 dSPM 50 6.470 0.000 114

25 171 dSPM 60 5.203 0.000 72

25 171 dSPM 70 5.573 0.000 46

25 171 dSPM 80 6.177 0.000 18

25 171 dSPM 90 5.465 0.000 7

25 171 dSPM 100 0.000 0.000 1

25 171 MNE 0 25.976 0.000 8003

25 171 MNE 10 17.885 0.000 979

25 171 MNE 20 9.746 0.000 284

25 171 MNE 30 6.358 0.000 129

25 171 MNE 40 5.129 0.000 60

25 171 MNE 50 1.128 0.000 27

25 171 MNE 60 1.025 0.000 14

25 171 MNE 70 0.000 0.000 6

25 171 MNE 80 0.000 0.000 3

25 171 MNE 90 0.000 0.000 1

25 171 MNE 100 0.000 0.000 1

25 171 sLORETA 0 25.238 0.000 8003

25 171 sLORETA 10 20.001 0.000 2189

25 171 sLORETA 20 12.295 0.000 759

25 171 sLORETA 30 7.846 0.000 360

25 171 sLORETA 40 3.958 0.000 176

25 171 sLORETA 50 2.512 0.000 99

25 171 sLORETA 60 1.082 0.000 49

25 171 sLORETA 70 0.000 0.000 26

25 171 sLORETA 80 0.000 0.000 13

25 171 sLORETA 90 0.000 0.000 7

25 171 sLORETA 100 0.000 0.000 1

25 172 Ave 0 28.678 0.000 8003

25 172 Ave 10 24.268 0.000 2423

25 172 Ave 20 19.614 0.000 971

25 172 Ave 30 16.711 0.000 400

25 172 Ave 40 12.872 0.000 171

25 172 Ave 50 5.354 0.000 71

25 172 Ave 60 0.000 0.000 34

25 172 Ave 70 0.000 0.000 16

25 172 Ave 80 0.000 0.000 10

25 172 Ave 90 0.000 0.000 6

25 172 Ave 100 0.000 0.000 1

25 172 cMEM 0 25.724 0.000 8003

25 172 cMEM 10 25.019 0.000 515

25 172 cMEM 20 25.156 0.000 240

25 172 cMEM 30 25.157 0.000 121

25 172 cMEM 40 24.796 0.000 72

25 172 cMEM 50 25.355 0.000 47

25 172 cMEM 60 25.732 0.000 29

25 172 cMEM 70 26.817 0.000 17

25 172 cMEM 80 23.320 0.000 8

25 172 cMEM 90 26.664 0.000 2

25 172 cMEM 100 35.875 35.875 1

25 172 dSPM 0 30.133 0.000 8003

25 172 dSPM 10 27.597 0.000 3037

25 172 dSPM 20 23.584 0.000 1419

25 172 dSPM 30 21.115 0.000 667

25 172 dSPM 40 19.935 0.000 342

25 172 dSPM 50 18.057 0.000 183

25 172 dSPM 60 13.642 0.000 102

25 172 dSPM 70 5.198 0.000 44

25 172 dSPM 80 5.185 0.000 17

25 172 dSPM 90 5.465 0.000 8

25 172 dSPM 100 0.000 0.000 1

25 172 MNE 0 29.420 0.000 8003

25 172 MNE 10 23.979 0.000 1360

25 172 MNE 20 18.851 0.000 445

25 172 MNE 30 14.628 0.000 167

25 172 MNE 40 12.113 0.000 74

25 172 MNE 50 8.861 0.000 36

25 172 MNE 60 9.315 0.000 16

25 172 MNE 70 0.000 0.000 6

25 172 MNE 80 0.000 0.000 5

25 172 MNE 90 0.000 0.000 2

25 172 MNE 100 0.000 0.000 1

25 172 sLORETA 0 28.254 0.000 8003

25 172 sLORETA 10 24.304 0.000 2622

25 172 sLORETA 20 19.669 0.000 1174

25 172 sLORETA 30 16.332 0.000 513

25 172 sLORETA 40 11.292 0.000 215

25 172 sLORETA 50 3.115 0.000 94

25 172 sLORETA 60 1.159 0.000 44

25 172 sLORETA 70 0.000 0.000 21

25 172 sLORETA 80 0.000 0.000 13

25 172 sLORETA 90 0.000 0.000 8

25 172 sLORETA 100 0.000 0.000 1

25 173 Ave 0 22.171 0.000 8003

25 173 Ave 10 17.243 0.000 2053

25 173 Ave 20 11.211 0.000 905

25 173 Ave 30 9.045 0.000 543

25 173 Ave 40 7.442 0.000 334

25 173 Ave 50 5.985 0.000 194

25 173 Ave 60 4.801 0.000 106

25 173 Ave 70 3.915 0.000 59

25 173 Ave 80 3.159 0.000 37

25 173 Ave 90 3.326 0.000 13

25 173 Ave 100 3.631 3.631 1

25 173 cMEM 0 10.633 0.000 8003

25 173 cMEM 10 9.358 0.000 557

25 173 cMEM 20 8.253 0.000 282

25 173 cMEM 30 7.404 0.000 175

25 173 cMEM 40 5.521 0.000 113

25 173 cMEM 50 3.540 0.000 62

25 173 cMEM 60 2.463 0.000 42

25 173 cMEM 70 0.960 0.000 24

25 173 cMEM 80 0.000 0.000 11

25 173 cMEM 90 0.000 0.000 2

25 173 cMEM 100 0.000 0.000 1

25 173 dSPM 0 24.293 0.000 8003

25 173 dSPM 10 19.725 0.000 2244

25 173 dSPM 20 14.717 0.000 1035

25 173 dSPM 30 12.868 0.000 611

25 173 dSPM 40 11.190 0.000 339

25 173 dSPM 50 8.515 0.000 158

25 173 dSPM 60 7.521 0.000 89

25 173 dSPM 70 7.760 0.000 53

25 173 dSPM 80 7.120 0.000 25

25 173 dSPM 90 7.443 0.000 9

25 173 dSPM 100 9.875 9.875 1

25 173 MNE 0 25.996 0.000 8003

25 173 MNE 10 21.590 0.000 1473

25 173 MNE 20 14.123 0.000 581

25 173 MNE 30 10.434 0.000 321

25 173 MNE 40 9.217 0.000 189

25 173 MNE 50 7.601 0.000 94

25 173 MNE 60 7.538 0.000 45

25 173 MNE 70 7.793 0.000 24

25 173 MNE 80 3.067 0.000 7

25 173 MNE 90 2.866 0.000 3

25 173 MNE 100 3.370 3.370 1

25 173 sLORETA 0 23.539 0.000 8003

25 173 sLORETA 10 19.869 0.000 2450

25 173 sLORETA 20 12.877 0.000 1073

25 173 sLORETA 30 10.440 0.000 639

25 173 sLORETA 40 8.645 0.000 418

25 173 sLORETA 50 7.916 0.000 259

25 173 sLORETA 60 6.181 0.000 143

25 173 sLORETA 70 3.940 0.000 81

25 173 sLORETA 80 3.569 0.000 36

25 173 sLORETA 90 3.483 0.000 9

25 173 sLORETA 100 0.000 0.000 1

25 174 Ave 0 25.041 0.000 8003

25 174 Ave 10 20.556 0.000 2441

25 174 Ave 20 13.174 0.000 956

25 174 Ave 30 11.123 0.000 556

25 174 Ave 40 9.773 0.000 311

25 174 Ave 50 7.811 0.000 181

25 174 Ave 60 6.150 0.000 111

25 174 Ave 70 5.180 0.000 68

25 174 Ave 80 3.637 0.000 34

25 174 Ave 90 2.299 0.000 9

25 174 Ave 100 3.631 3.631 1

25 174 cMEM 0 8.712 0.000 8003

25 174 cMEM 10 7.665 0.000 301

25 174 cMEM 20 7.340 0.000 148

25 174 cMEM 30 5.943 0.000 57

25 174 cMEM 40 3.255 0.000 8

25 174 cMEM 50 0.000 0.000 4

25 174 cMEM 60 0.000 0.000 4

25 174 cMEM 70 0.000 0.000 3

25 174 cMEM 80 0.000 0.000 2

25 174 cMEM 90 0.000 0.000 1

25 174 cMEM 100 0.000 0.000 1

25 174 dSPM 0 26.599 0.000 8003

25 174 dSPM 10 22.424 0.000 2550

25 174 dSPM 20 16.439 0.000 1029

25 174 dSPM 30 15.156 0.000 549

25 174 dSPM 40 13.829 0.000 316

25 174 dSPM 50 11.762 0.000 176

25 174 dSPM 60 9.979 0.000 95

25 174 dSPM 70 8.988 0.000 47

25 174 dSPM 80 9.300 0.000 26

25 174 dSPM 90 10.633 4.138 6

25 174 dSPM 100 9.875 9.875 1

25 174 MNE 0 28.646 0.000 8003

25 174 MNE 10 24.819 0.000 1732

25 174 MNE 20 16.649 0.000 672

25 174 MNE 30 12.205 0.000 349

25 174 MNE 40 11.252 0.000 188

25 174 MNE 50 10.649 0.000 95

25 174 MNE 60 9.658 0.000 54

25 174 MNE 70 4.446 0.000 24

25 174 MNE 80 3.523 0.000 12

25 174 MNE 90 3.041 0.000 7

25 174 MNE 100 0.000 0.000 1

25 174 sLORETA 0 25.134 0.000 8003

25 174 sLORETA 10 21.333 0.000 2726

25 174 sLORETA 20 13.969 0.000 1092

25 174 sLORETA 30 11.758 0.000 650

25 174 sLORETA 40 10.279 0.000 382

25 174 sLORETA 50 8.608 0.000 216

25 174 sLORETA 60 6.069 0.000 133

25 174 sLORETA 70 4.920 0.000 79

25 174 sLORETA 80 2.948 0.000 40

25 174 sLORETA 90 2.550 0.000 10

25 174 sLORETA 100 0.000 0.000 1

25 175 Ave 0 28.577 0.000 8003

25 175 Ave 10 23.054 0.000 1972

25 175 Ave 20 12.545 0.000 638

25 175 Ave 30 7.049 0.000 325

25 175 Ave 40 5.908 0.000 188

25 175 Ave 50 5.216 0.000 102

25 175 Ave 60 4.507 0.000 61

25 175 Ave 70 4.255 0.000 23

25 175 Ave 80 4.625 0.000 6

25 175 Ave 90 3.631 3.631 1

25 175 Ave 100 3.631 3.631 1

25 175 cMEM 0 9.183 0.000 8003

25 175 cMEM 10 8.326 0.000 432

25 175 cMEM 20 7.213 0.000 240

25 175 cMEM 30 6.615 0.000 158

25 175 cMEM 40 6.084 0.000 110

25 175 cMEM 50 4.844 0.000 77

25 175 cMEM 60 4.543 0.000 49

25 175 cMEM 70 4.503 0.000 25

25 175 cMEM 80 5.189 0.000 11

25 175 cMEM 90 5.656 4.930 4

25 175 cMEM 100 6.260 6.260 1

25 175 dSPM 0 30.595 0.000 8003

25 175 dSPM 10 27.489 0.000 2740

25 175 dSPM 20 17.591 0.000 950

25 175 dSPM 30 13.355 0.000 481

25 175 dSPM 40 11.646 0.000 271

25 175 dSPM 50 9.931 0.000 151

25 175 dSPM 60 8.506 0.000 76

25 175 dSPM 70 8.621 0.000 45

25 175 dSPM 80 8.766 0.000 25

25 175 dSPM 90 7.366 2.775 8

25 175 dSPM 100 6.440 6.440 1

25 175 MNE 0 34.289 0.000 8003

25 175 MNE 10 31.381 0.000 1722

25 175 MNE 20 23.696 0.000 576

25 175 MNE 30 15.411 0.000 257

25 175 MNE 40 9.011 0.000 145

25 175 MNE 50 5.798 0.000 80

25 175 MNE 60 5.247 0.000 36

25 175 MNE 70 5.548 0.000 15

25 175 MNE 80 5.884 0.000 9

25 175 MNE 90 7.959 0.000 4

25 175 MNE 100 3.631 3.631 1

25 175 sLORETA 0 29.743 0.000 8003

25 175 sLORETA 10 26.809 0.000 2808

25 175 sLORETA 20 17.422 0.000 1016

25 175 sLORETA 30 10.653 0.000 472

25 175 sLORETA 40 6.527 0.000 299

25 175 sLORETA 50 5.792 0.000 190

25 175 sLORETA 60 5.191 0.000 117

25 175 sLORETA 70 4.039 0.000 52

25 175 sLORETA 80 3.216 0.000 30

25 175 sLORETA 90 2.134 0.000 11

25 175 sLORETA 100 0.000 0.000 1

25 176 Ave 0 24.375 0.000 8003

25 176 Ave 10 17.393 0.000 1604

25 176 Ave 20 10.318 0.000 575

25 176 Ave 30 8.358 0.000 277

25 176 Ave 40 7.265 0.000 152

25 176 Ave 50 6.455 0.000 90

25 176 Ave 60 5.667 0.000 45

25 176 Ave 70 5.120 0.000 25

25 176 Ave 80 2.708 0.000 9

25 176 Ave 90 2.953 0.000 4

25 176 Ave 100 3.631 3.631 1

25 176 cMEM 0 8.411 0.000 8003

25 176 cMEM 10 8.114 0.000 223

25 176 cMEM 20 7.965 0.000 158

25 176 cMEM 30 7.512 0.000 120

25 176 cMEM 40 7.381 0.000 95

25 176 cMEM 50 6.992 0.000 71

25 176 cMEM 60 7.218 0.000 47

25 176 cMEM 70 6.509 0.000 22

25 176 cMEM 80 5.917 3.631 8

25 176 cMEM 90 5.670 4.930 4

25 176 cMEM 100 6.260 6.260 1

25 176 dSPM 0 27.978 0.000 8003

25 176 dSPM 10 24.802 0.000 2529

25 176 dSPM 20 17.374 0.000 916

25 176 dSPM 30 13.233 0.000 447

25 176 dSPM 40 12.465 0.000 240

25 176 dSPM 50 10.844 0.000 140

25 176 dSPM 60 8.403 0.000 77

25 176 dSPM 70 8.481 0.000 46

25 176 dSPM 80 8.098 0.000 26

25 176 dSPM 90 8.171 0.000 15

25 176 dSPM 100 4.553 4.553 1

25 176 MNE 0 24.904 0.000 8003

25 176 MNE 10 19.265 0.000 1209

25 176 MNE 20 13.383 0.000 469

25 176 MNE 30 9.938 0.000 219

25 176 MNE 40 8.301 0.000 131

25 176 MNE 50 7.725 0.000 73

25 176 MNE 60 7.697 0.000 35

25 176 MNE 70 6.309 0.000 15

25 176 MNE 80 6.035 0.000 7

25 176 MNE 90 3.433 0.000 3

25 176 MNE 100 3.631 3.631 1

25 176 sLORETA 0 25.329 0.000 8003

25 176 sLORETA 10 20.593 0.000 2214

25 176 sLORETA 20 12.921 0.000 834

25 176 sLORETA 30 9.888 0.000 433

25 176 sLORETA 40 7.955 0.000 239

25 176 sLORETA 50 6.288 0.000 134

25 176 sLORETA 60 5.150 0.000 77

25 176 sLORETA 70 3.843 0.000 39

25 176 sLORETA 80 2.431 0.000 19

25 176 sLORETA 90 1.452 0.000 6

25 176 sLORETA 100 0.000 0.000 1

25 177 Ave 0 23.959 0.000 8003

25 177 Ave 10 14.824 0.000 1066

25 177 Ave 20 10.152 0.000 434

25 177 Ave 30 8.713 0.000 190

25 177 Ave 40 5.317 0.000 110

25 177 Ave 50 4.188 0.000 74

25 177 Ave 60 3.291 0.000 38

25 177 Ave 70 3.357 0.000 26

25 177 Ave 80 3.439 0.000 12

25 177 Ave 90 2.421 0.000 5

25 177 Ave 100 4.003 4.003 1

25 177 cMEM 0 6.680 0.000 8003

25 177 cMEM 10 6.508 0.000 234

25 177 cMEM 20 6.169 0.000 153

25 177 cMEM 30 5.399 0.000 96

25 177 cMEM 40 4.776 0.000 70

25 177 cMEM 50 4.102 0.000 42

25 177 cMEM 60 2.452 0.000 22

25 177 cMEM 70 2.267 0.000 15

25 177 cMEM 80 2.147 0.000 9

25 177 cMEM 90 2.232 0.000 3

25 177 cMEM 100 0.000 0.000 1

25 177 dSPM 0 27.061 0.000 8003

25 177 dSPM 10 21.867 0.000 2096

25 177 dSPM 20 14.316 0.000 779

25 177 dSPM 30 12.685 0.000 420

25 177 dSPM 40 11.248 0.000 219

25 177 dSPM 50 7.271 0.000 104

25 177 dSPM 60 4.583 0.000 70

25 177 dSPM 70 3.987 0.000 39

25 177 dSPM 80 3.106 0.000 20

25 177 dSPM 90 2.821 0.000 9

25 177 dSPM 100 0.000 0.000 1

25 177 MNE 0 27.844 0.000 8003

25 177 MNE 10 20.371 0.000 780

25 177 MNE 20 13.316 0.000 264

25 177 MNE 30 11.733 0.000 119

25 177 MNE 40 9.578 0.000 46

25 177 MNE 50 7.198 0.000 28

25 177 MNE 60 6.330 0.000 20

25 177 MNE 70 3.841 0.000 9

25 177 MNE 80 3.006 0.000 6

25 177 MNE 90 4.058 3.370 2

25 177 MNE 100 4.583 4.583 1

25 177 sLORETA 0 25.242 0.000 8003

25 177 sLORETA 10 18.373 0.000 1398

25 177 sLORETA 20 12.089 0.000 532

25 177 sLORETA 30 11.023 0.000 261

25 177 sLORETA 40 9.575 0.000 135

25 177 sLORETA 50 6.801 0.000 81

25 177 sLORETA 60 4.089 0.000 47

25 177 sLORETA 70 3.916 0.000 30

25 177 sLORETA 80 4.134 0.000 16

25 177 sLORETA 90 4.576 0.000 7

25 177 sLORETA 100 0.000 0.000 1

26 178 Ave 0 13.133 0.000 8002

26 178 Ave 10 8.295 0.000 512

26 178 Ave 20 6.481 0.000 204

26 178 Ave 30 5.030 0.000 106

26 178 Ave 40 4.327 0.000 64

26 178 Ave 50 3.795 0.000 48

26 178 Ave 60 3.381 0.000 35

26 178 Ave 70 2.821 0.000 18

26 178 Ave 80 2.898 0.000 13

26 178 Ave 90 2.084 0.000 5

26 178 Ave 100 0.000 0.000 1

26 178 cMEM 0 5.486 0.000 8002

26 178 cMEM 10 5.286 0.000 163

26 178 cMEM 20 5.098 0.000 128

26 178 cMEM 30 4.768 0.000 101

26 178 cMEM 40 4.447 0.000 80

26 178 cMEM 50 4.089 0.000 54

26 178 cMEM 60 3.453 0.000 35

26 178 cMEM 70 3.329 0.000 18

26 178 cMEM 80 3.482 0.000 12

26 178 cMEM 90 2.211 0.000 4

26 178 cMEM 100 0.000 0.000 1

26 178 dSPM 0 15.954 0.000 8002

26 178 dSPM 10 11.866 0.000 812

26 178 dSPM 20 9.593 0.000 384

26 178 dSPM 30 7.536 0.000 187

26 178 dSPM 40 5.001 0.000 90

26 178 dSPM 50 4.126 0.000 49

26 178 dSPM 60 3.189 0.000 33

26 178 dSPM 70 3.015 0.000 22

26 178 dSPM 80 3.309 0.000 11

26 178 dSPM 90 2.941 0.000 3

26 178 dSPM 100 4.872 4.872 1

26 178 MNE 0 13.709 0.000 8002

26 178 MNE 10 9.260 0.000 344

26 178 MNE 20 8.099 0.000 162

26 178 MNE 30 7.221 0.000 92

26 178 MNE 40 5.084 0.000 48

26 178 MNE 50 3.645 0.000 29

26 178 MNE 60 3.714 0.000 21

26 178 MNE 70 2.699 0.000 13

26 178 MNE 80 2.632 0.000 9

26 178 MNE 90 4.211 0.000 3

26 178 MNE 100 0.000 0.000 1

26 178 sLORETA 0 15.392 0.000 8002

26 178 sLORETA 10 9.591 0.000 597

26 178 sLORETA 20 8.126 0.000 274

26 178 sLORETA 30 6.636 0.000 132

26 178 sLORETA 40 5.217 0.000 78

26 178 sLORETA 50 3.682 0.000 48

26 178 sLORETA 60 3.588 0.000 31

26 178 sLORETA 70 3.191 0.000 22

26 178 sLORETA 80 2.466 0.000 11

26 178 sLORETA 90 2.291 0.000 6

26 178 sLORETA 100 0.000 0.000 1

26 179 Ave 0 13.552 0.000 8002

26 179 Ave 10 8.467 0.000 532

26 179 Ave 20 6.781 0.000 210

26 179 Ave 30 5.122 0.000 106

26 179 Ave 40 4.346 0.000 66

26 179 Ave 50 3.622 0.000 47

26 179 Ave 60 3.037 0.000 34

26 179 Ave 70 2.674 0.000 19

26 179 Ave 80 2.410 0.000 11

26 179 Ave 90 0.000 0.000 5

26 179 Ave 100 0.000 0.000 1

26 179 cMEM 0 5.387 0.000 8002

26 179 cMEM 10 5.093 0.000 194

26 179 cMEM 20 4.581 0.000 130

26 179 cMEM 30 4.059 0.000 97

26 179 cMEM 40 3.412 0.000 70

26 179 cMEM 50 2.633 0.000 50

26 179 cMEM 60 2.418 0.000 29

26 179 cMEM 70 1.729 0.000 12

26 179 cMEM 80 0.000 0.000 8

26 179 cMEM 90 0.000 0.000 3

26 179 cMEM 100 0.000 0.000 1

26 179 dSPM 0 16.043 0.000 8002

26 179 dSPM 10 11.323 0.000 800

26 179 dSPM 20 9.370 0.000 376

26 179 dSPM 30 7.521 0.000 187

26 179 dSPM 40 5.121 0.000 90

26 179 dSPM 50 4.006 0.000 52

26 179 dSPM 60 2.961 0.000 34

26 179 dSPM 70 2.975 0.000 22

26 179 dSPM 80 3.159 0.000 12

26 179 dSPM 90 2.575 0.000 4

26 179 dSPM 100 4.872 4.872 1

26 179 MNE 0 13.697 0.000 8002

26 179 MNE 10 9.265 0.000 350

26 179 MNE 20 8.023 0.000 159

26 179 MNE 30 6.880 0.000 94

26 179 MNE 40 4.840 0.000 48

26 179 MNE 50 3.564 0.000 32

26 179 MNE 60 3.870 0.000 22

26 179 MNE 70 2.680 0.000 13

26 179 MNE 80 2.622 0.000 9

26 179 MNE 90 3.694 0.000 4

26 179 MNE 100 0.000 0.000 1

26 179 sLORETA 0 15.809 0.000 8002

26 179 sLORETA 10 9.665 0.000 604

26 179 sLORETA 20 8.063 0.000 266

26 179 sLORETA 30 6.722 0.000 138

26 179 sLORETA 40 5.469 0.000 79

26 179 sLORETA 50 4.026 0.000 52

26 179 sLORETA 60 3.323 0.000 33

26 179 sLORETA 70 2.956 0.000 20

26 179 sLORETA 80 2.444 0.000 11

26 179 sLORETA 90 2.264 0.000 6

26 179 sLORETA 100 0.000 0.000 1

26 180 Ave 0 12.837 0.000 8002

26 180 Ave 10 8.181 0.000 515

26 180 Ave 20 6.408 0.000 199

26 180 Ave 30 5.065 0.000 104

26 180 Ave 40 4.316 0.000 66

26 180 Ave 50 3.623 0.000 47

26 180 Ave 60 3.323 0.000 35

26 180 Ave 70 2.665 0.000 19

26 180 Ave 80 2.549 0.000 13

26 180 Ave 90 0.000 0.000 4

26 180 Ave 100 0.000 0.000 1

26 180 cMEM 0 6.258 0.000 8002

26 180 cMEM 10 5.753 0.000 172

26 180 cMEM 20 5.516 0.000 132

26 180 cMEM 30 5.031 0.000 105

26 180 cMEM 40 3.723 0.000 70

26 180 cMEM 50 3.121 0.000 49

26 180 cMEM 60 2.744 0.000 32

26 180 cMEM 70 1.829 0.000 15

26 180 cMEM 80 1.769 0.000 6

26 180 cMEM 90 0.000 0.000 3

26 180 cMEM 100 0.000 0.000 1

26 180 dSPM 0 15.423 0.000 8002

26 180 dSPM 10 11.026 0.000 776

26 180 dSPM 20 9.281 0.000 375

26 180 dSPM 30 7.353 0.000 184

26 180 dSPM 40 4.836 0.000 95

26 180 dSPM 50 3.824 0.000 49

26 180 dSPM 60 2.876 0.000 34

26 180 dSPM 70 3.032 0.000 23

26 180 dSPM 80 3.188 0.000 11

26 180 dSPM 90 2.563 0.000 4

26 180 dSPM 100 4.872 4.872 1

26 180 MNE 0 13.281 0.000 8002

26 180 MNE 10 9.013 0.000 354

26 180 MNE 20 7.658 0.000 163

26 180 MNE 30 6.307 0.000 89

26 180 MNE 40 4.843 0.000 49

26 180 MNE 50 3.592 0.000 32

26 180 MNE 60 3.902 0.000 22

26 180 MNE 70 2.666 0.000 13

26 180 MNE 80 2.596 0.000 9

26 180 MNE 90 3.676 0.000 4

26 180 MNE 100 0.000 0.000 1

26 180 sLORETA 0 15.003 0.000 8002

26 180 sLORETA 10 9.325 0.000 586

26 180 sLORETA 20 7.773 0.000 255

26 180 sLORETA 30 6.502 0.000 135

26 180 sLORETA 40 5.492 0.000 79

26 180 sLORETA 50 4.228 0.000 53

26 180 sLORETA 60 3.330 0.000 31

26 180 sLORETA 70 3.083 0.000 21

26 180 sLORETA 80 2.351 0.000 12

26 180 sLORETA 90 2.269 0.000 6

26 180 sLORETA 100 0.000 0.000 1

26 181 Ave 0 16.751 0.000 8002

26 181 Ave 10 9.144 0.000 551

26 181 Ave 20 7.298 0.000 238

26 181 Ave 30 5.477 0.000 105

26 181 Ave 40 4.651 0.000 65

26 181 Ave 50 4.192 0.000 43

26 181 Ave 60 3.942 0.000 33

26 181 Ave 70 3.588 0.000 21

26 181 Ave 80 3.460 0.000 11

26 181 Ave 90 4.219 0.000 4

26 181 Ave 100 4.708 4.708 1

26 181 cMEM 0 5.522 0.000 8002

26 181 cMEM 10 5.169 0.000 125

26 181 cMEM 20 4.986 0.000 87

26 181 cMEM 30 4.934 0.000 64

26 181 cMEM 40 4.591 0.000 42

26 181 cMEM 50 4.886 0.000 21

26 181 cMEM 60 5.091 3.094 10

26 181 cMEM 70 5.116 3.538 8

26 181 cMEM 80 5.209 3.856 6

26 181 cMEM 90 4.442 3.856 4

26 181 cMEM 100 4.499 4.499 1

26 181 dSPM 0 18.747 0.000 8002

26 181 dSPM 10 12.983 0.000 840

26 181 dSPM 20 10.259 0.000 393

26 181 dSPM 30 8.148 0.000 193

26 181 dSPM 40 5.618 0.000 95

26 181 dSPM 50 4.095 0.000 49

26 181 dSPM 60 3.566 0.000 34

26 181 dSPM 70 3.224 0.000 19

26 181 dSPM 80 3.329 0.000 11

26 181 dSPM 90 2.970 0.000 3

26 181 dSPM 100 4.872 4.872 1

26 181 MNE 0 17.592 0.000 8002

26 181 MNE 10 9.745 0.000 362

26 181 MNE 20 8.462 0.000 168

26 181 MNE 30 7.361 0.000 93

26 181 MNE 40 5.148 0.000 52

26 181 MNE 50 4.299 0.000 33

26 181 MNE 60 3.850 0.000 23

26 181 MNE 70 2.731 0.000 13

26 181 MNE 80 2.657 0.000 9

26 181 MNE 90 4.231 0.000 3

26 181 MNE 100 0.000 0.000 1

26 181 sLORETA 0 19.309 0.000 8002

26 181 sLORETA 10 10.253 0.000 654

26 181 sLORETA 20 8.575 0.000 281

26 181 sLORETA 30 7.127 0.000 139

26 181 sLORETA 40 5.621 0.000 83

26 181 sLORETA 50 4.340 0.000 54

26 181 sLORETA 60 3.615 0.000 33

26 181 sLORETA 70 3.245 0.000 22

26 181 sLORETA 80 2.487 0.000 11

26 181 sLORETA 90 2.310 0.000 6

26 181 sLORETA 100 0.000 0.000 1

26 182 Ave 0 27.285 0.000 8002

26 182 Ave 10 16.357 0.000 900

26 182 Ave 20 10.678 0.000 366

26 182 Ave 30 8.633 0.000 173

26 182 Ave 40 6.534 0.000 84

26 182 Ave 50 3.180 0.000 51

26 182 Ave 60 2.278 0.000 35

26 182 Ave 70 2.342 0.000 25

26 182 Ave 80 2.114 0.000 15

26 182 Ave 90 0.000 0.000 7

26 182 Ave 100 0.000 0.000 1

26 182 cMEM 0 16.003 0.000 8002

26 182 cMEM 10 15.449 0.000 402

26 182 cMEM 20 14.095 0.000 211

26 182 cMEM 30 13.007 0.000 137

26 182 cMEM 40 11.124 0.000 81

26 182 cMEM 50 8.481 0.000 50

26 182 cMEM 60 7.445 0.000 29

26 182 cMEM 70 5.327 0.000 16

26 182 cMEM 80 3.678 0.000 13

26 182 cMEM 90 2.369 0.000 4

26 182 cMEM 100 4.560 4.560 1

26 182 dSPM 0 28.479 0.000 8002

26 182 dSPM 10 21.918 0.000 1336

26 182 dSPM 20 16.646 0.000 560

26 182 dSPM 30 12.532 0.000 303

26 182 dSPM 40 9.806 0.000 170

26 182 dSPM 50 6.920 0.000 83

26 182 dSPM 60 4.035 0.000 44

26 182 dSPM 70 2.946 0.000 27

26 182 dSPM 80 3.134 0.000 10

26 182 dSPM 90 3.357 0.000 4

26 182 dSPM 100 4.872 4.872 1

26 182 MNE 0 30.876 0.000 8002

26 182 MNE 10 20.488 0.000 487

26 182 MNE 20 11.178 0.000 180

26 182 MNE 30 10.593 0.000 91

26 182 MNE 40 7.232 0.000 52

26 182 MNE 50 5.689 0.000 34

26 182 MNE 60 3.257 0.000 22

26 182 MNE 70 3.759 0.000 12

26 182 MNE 80 2.424 0.000 10

26 182 MNE 90 2.643 0.000 3

26 182 MNE 100 0.000 0.000 1

26 182 sLORETA 0 32.753 0.000 8002

26 182 sLORETA 10 23.721 0.000 1235

26 182 sLORETA 20 10.221 0.000 342

26 182 sLORETA 30 8.748 0.000 173

26 182 sLORETA 40 6.631 0.000 88

26 182 sLORETA 50 3.784 0.000 55

26 182 sLORETA 60 3.217 0.000 31

26 182 sLORETA 70 2.195 0.000 18

26 182 sLORETA 80 2.241 0.000 10

26 182 sLORETA 90 0.000 0.000 5

26 182 sLORETA 100 0.000 0.000 1

26 183 Ave 0 15.292 0.000 8002

26 183 Ave 10 8.731 0.000 523

26 183 Ave 20 7.070 0.000 215

26 183 Ave 30 5.462 0.000 113

26 183 Ave 40 4.563 0.000 67

26 183 Ave 50 3.935 0.000 46

26 183 Ave 60 3.565 0.000 30

26 183 Ave 70 2.930 0.000 16

26 183 Ave 80 2.884 0.000 14

26 183 Ave 90 0.000 0.000 4

26 183 Ave 100 0.000 0.000 1

26 183 cMEM 0 5.969 0.000 8002

26 183 cMEM 10 5.680 0.000 178

26 183 cMEM 20 5.146 0.000 111

26 183 cMEM 30 4.145 0.000 68

26 183 cMEM 40 3.327 0.000 41

26 183 cMEM 50 2.771 0.000 27

26 183 cMEM 60 2.537 0.000 15

26 183 cMEM 70 1.741 0.000 6

26 183 cMEM 80 0.000 0.000 4

26 183 cMEM 90 0.000 0.000 3

26 183 cMEM 100 0.000 0.000 1

26 183 dSPM 0 17.012 0.000 8002

26 183 dSPM 10 11.833 0.000 782

26 183 dSPM 20 9.195 0.000 367

26 183 dSPM 30 7.191 0.000 183

26 183 dSPM 40 5.054 0.000 90

26 183 dSPM 50 4.145 0.000 52

26 183 dSPM 60 3.600 0.000 35

26 183 dSPM 70 3.248 0.000 21

26 183 dSPM 80 3.385 0.000 9

26 183 dSPM 90 3.922 0.000 4

26 183 dSPM 100 4.872 4.872 1

26 183 MNE 0 16.326 0.000 8002

26 183 MNE 10 9.552 0.000 352

26 183 MNE 20 8.487 0.000 170

26 183 MNE 30 7.165 0.000 93

26 183 MNE 40 5.717 0.000 47

26 183 MNE 50 3.848 0.000 30

26 183 MNE 60 3.859 0.000 22

26 183 MNE 70 2.874 0.000 12

26 183 MNE 80 3.186 0.000 9

26 183 MNE 90 3.707 0.000 4

26 183 MNE 100 0.000 0.000 1

26 183 sLORETA 0 17.189 0.000 8002

26 183 sLORETA 10 9.707 0.000 590

26 183 sLORETA 20 8.418 0.000 278

26 183 sLORETA 30 7.032 0.000 142

26 183 sLORETA 40 5.490 0.000 79

26 183 sLORETA 50 4.682 0.000 53

26 183 sLORETA 60 3.914 0.000 32

26 183 sLORETA 70 3.397 0.000 21

26 183 sLORETA 80 2.751 0.000 9

26 183 sLORETA 90 2.404 0.000 6

26 183 sLORETA 100 0.000 0.000 1

26 184 Ave 0 16.326 0.000 8002

26 184 Ave 10 8.363 0.000 523

26 184 Ave 20 6.350 0.000 195

26 184 Ave 30 5.055 0.000 100

26 184 Ave 40 4.400 0.000 63

26 184 Ave 50 3.789 0.000 49

26 184 Ave 60 3.241 0.000 27

26 184 Ave 70 2.895 0.000 18

26 184 Ave 80 2.550 0.000 13

26 184 Ave 90 0.000 0.000 4

26 184 Ave 100 0.000 0.000 1

26 184 cMEM 0 4.900 0.000 8002

26 184 cMEM 10 4.582 0.000 158

26 184 cMEM 20 4.010 0.000 102

26 184 cMEM 30 3.387 0.000 74

26 184 cMEM 40 2.987 0.000 45

26 184 cMEM 50 2.710 0.000 28

26 184 cMEM 60 2.125 0.000 17

26 184 cMEM 70 2.217 0.000 8

26 184 cMEM 80 1.844 0.000 6

26 184 cMEM 90 0.000 0.000 3

26 184 cMEM 100 0.000 0.000 1

26 184 dSPM 0 18.009 0.000 8002

26 184 dSPM 10 11.926 0.000 815

26 184 dSPM 20 9.134 0.000 358

26 184 dSPM 30 7.407 0.000 184

26 184 dSPM 40 4.988 0.000 91

26 184 dSPM 50 4.095 0.000 51

26 184 dSPM 60 3.243 0.000 34

26 184 dSPM 70 3.217 0.000 21

26 184 dSPM 80 3.362 0.000 9

26 184 dSPM 90 3.912 0.000 4

26 184 dSPM 100 4.872 4.872 1

26 184 MNE 0 17.562 0.000 8002

26 184 MNE 10 8.892 0.000 358

26 184 MNE 20 7.541 0.000 157

26 184 MNE 30 6.022 0.000 82

26 184 MNE 40 4.974 0.000 49

26 184 MNE 50 3.730 0.000 33

26 184 MNE 60 3.761 0.000 24

26 184 MNE 70 3.895 0.000 14

26 184 MNE 80 3.132 0.000 9

26 184 MNE 90 3.699 0.000 4

26 184 MNE 100 0.000 0.000 1

26 184 sLORETA 0 18.859 0.000 8002

26 184 sLORETA 10 9.427 0.000 597

26 184 sLORETA 20 7.727 0.000 260

26 184 sLORETA 30 6.314 0.000 136

26 184 sLORETA 40 5.451 0.000 80

26 184 sLORETA 50 4.713 0.000 56

26 184 sLORETA 60 3.774 0.000 32

26 184 sLORETA 70 3.198 0.000 23

26 184 sLORETA 80 2.587 0.000 10

26 184 sLORETA 90 2.347 0.000 6

26 184 sLORETA 100 0.000 0.000 1

26 185 Ave 0 21.445 0.000 8002

26 185 Ave 10 8.439 0.000 536

26 185 Ave 20 5.688 0.000 196

26 185 Ave 30 4.107 0.000 93

26 185 Ave 40 3.405 0.000 49

26 185 Ave 50 3.249 0.000 34

26 185 Ave 60 2.993 0.000 23

26 185 Ave 70 3.067 0.000 12

26 185 Ave 80 2.046 0.000 8

26 185 Ave 90 0.000 0.000 3

26 185 Ave 100 0.000 0.000 1

26 185 cMEM 0 3.881 0.000 8002

26 185 cMEM 10 3.113 0.000 48

26 185 cMEM 20 3.154 0.000 30

26 185 cMEM 30 3.115 0.000 22

26 185 cMEM 40 3.190 0.000 19

26 185 cMEM 50 2.585 0.000 11

26 185 cMEM 60 2.649 0.000 10

26 185 cMEM 70 1.841 0.000 5

26 185 cMEM 80 0.000 0.000 4

26 185 cMEM 90 0.000 0.000 3

26 185 cMEM 100 0.000 0.000 1

26 185 dSPM 0 21.570 0.000 8002

26 185 dSPM 10 11.689 0.000 841

26 185 dSPM 20 8.268 0.000 366

26 185 dSPM 30 5.580 0.000 161

26 185 dSPM 40 4.429 0.000 86

26 185 dSPM 50 3.997 0.000 55

26 185 dSPM 60 3.257 0.000 33

26 185 dSPM 70 3.210 0.000 21

26 185 dSPM 80 3.192 0.000 10

26 185 dSPM 90 3.440 0.000 2

26 185 dSPM 100 0.000 0.000 1

26 185 MNE 0 23.085 0.000 8002

26 185 MNE 10 11.686 0.000 417

26 185 MNE 20 7.139 0.000 161

26 185 MNE 30 5.580 0.000 86

26 185 MNE 40 4.078 0.000 45

26 185 MNE 50 3.465 0.000 30

26 185 MNE 60 2.501 0.000 19

26 185 MNE 70 2.752 0.000 12

26 185 MNE 80 3.382 0.000 7

26 185 MNE 90 0.000 0.000 2

26 185 MNE 100 0.000 0.000 1

26 185 sLORETA 0 24.271 0.000 8002

26 185 sLORETA 10 12.145 0.000 764

26 185 sLORETA 20 7.308 0.000 289

26 185 sLORETA 30 5.358 0.000 152

26 185 sLORETA 40 4.465 0.000 84

26 185 sLORETA 50 3.606 0.000 47

26 185 sLORETA 60 3.638 0.000 38

26 185 sLORETA 70 3.250 0.000 25

26 185 sLORETA 80 2.527 0.000 12

26 185 sLORETA 90 2.337 0.000 6

26 185 sLORETA 100 0.000 0.000 1

26 186 Ave 0 47.907 0.000 8002

26 186 Ave 10 46.925 0.000 3745

26 186 Ave 20 41.578 0.000 1329

26 186 Ave 30 33.143 0.000 438

26 186 Ave 40 24.567 0.000 181

26 186 Ave 50 19.870 4.613 90

26 186 Ave 60 19.209 7.779 49

26 186 Ave 70 18.849 7.955 25

26 186 Ave 80 19.788 10.337 11

26 186 Ave 90 19.305 15.883 4

26 186 Ave 100 18.387 18.387 1

26 186 cMEM 0 23.343 0.000 8002

26 186 cMEM 10 21.347 0.000 371

26 186 cMEM 20 20.537 0.000 196

26 186 cMEM 30 20.238 3.800 110

26 186 cMEM 40 20.044 3.800 78

26 186 cMEM 50 19.750 7.153 58

26 186 cMEM 60 19.530 7.153 43

26 186 cMEM 70 19.784 7.153 28

26 186 cMEM 80 19.198 12.003 13

26 186 cMEM 90 21.078 18.387 4

26 186 cMEM 100 22.179 22.179 1

26 186 dSPM 0 43.186 0.000 8002

26 186 dSPM 10 41.982 0.000 3915

26 186 dSPM 20 35.574 0.000 1582

26 186 dSPM 30 27.563 0.000 706

26 186 dSPM 40 23.079 0.000 346

26 186 dSPM 50 21.128 0.000 176

26 186 dSPM 60 19.967 0.000 95

26 186 dSPM 70 19.505 7.779 33

26 186 dSPM 80 17.360 7.779 9

26 186 dSPM 90 10.337 10.337 1

26 186 dSPM 100 10.337 10.337 1

26 186 MNE 0 56.965 0.000 8002

26 186 MNE 10 56.849 0.000 2497

26 186 MNE 20 55.215 0.000 834

26 186 MNE 30 51.763 0.000 333

26 186 MNE 40 48.428 0.000 144

26 186 MNE 50 45.504 0.000 64

26 186 MNE 60 40.000 15.433 28

26 186 MNE 70 33.211 15.588 13

26 186 MNE 80 20.148 16.443 5

26 186 MNE 90 19.763 16.443 2

26 186 MNE 100 22.250 22.250 1

26 186 sLORETA 0 51.527 0.000 8002

26 186 sLORETA 10 51.177 0.000 4616

26 186 sLORETA 20 49.206 0.000 2313

26 186 sLORETA 30 45.386 0.000 951

26 186 sLORETA 40 39.903 0.000 376

26 186 sLORETA 50 30.005 0.000 164

26 186 sLORETA 60 21.303 0.000 74

26 186 sLORETA 70 18.376 7.779 36

26 186 sLORETA 80 18.114 10.337 14

26 186 sLORETA 90 16.594 13.768 6

26 186 sLORETA 100 13.768 13.768 1

27 187 Ave 0 32.629 0.000 8002

27 187 Ave 10 30.449 0.000 3154

27 187 Ave 20 23.999 0.000 1292

27 187 Ave 30 17.817 0.000 629

27 187 Ave 40 14.141 0.000 384

27 187 Ave 50 13.245 0.000 235

27 187 Ave 60 12.093 0.000 129

27 187 Ave 70 10.463 0.000 56

27 187 Ave 80 9.619 0.000 23

27 187 Ave 90 10.148 0.000 7

27 187 Ave 100 9.915 9.915 1

27 187 cMEM 0 15.232 0.000 8002

27 187 cMEM 10 14.560 0.000 496

27 187 cMEM 20 13.931 0.000 255

27 187 cMEM 30 12.638 0.000 135

27 187 cMEM 40 11.072 0.000 64

27 187 cMEM 50 8.574 0.000 33

27 187 cMEM 60 2.408 0.000 14

27 187 cMEM 70 0.000 0.000 8

27 187 cMEM 80 0.000 0.000 5

27 187 cMEM 90 0.000 0.000 4

27 187 cMEM 100 0.000 0.000 1

27 187 dSPM 0 31.747 0.000 8002

27 187 dSPM 10 29.549 0.000 3285

27 187 dSPM 20 23.231 0.000 1312

27 187 dSPM 30 17.474 0.000 640

27 187 dSPM 40 15.681 0.000 424

27 187 dSPM 50 15.351 0.000 261

27 187 dSPM 60 15.045 0.000 157

27 187 dSPM 70 14.571 0.000 78

27 187 dSPM 80 14.130 0.000 30

27 187 dSPM 90 15.201 0.000 12

27 187 dSPM 100 13.440 13.440 1

27 187 MNE 0 36.377 0.000 8002

27 187 MNE 10 33.864 0.000 1989

27 187 MNE 20 27.813 0.000 742

27 187 MNE 30 22.094 0.000 322

27 187 MNE 40 16.384 0.000 163

27 187 MNE 50 13.036 0.000 95

27 187 MNE 60 12.373 0.000 50

27 187 MNE 70 10.927 0.000 25

27 187 MNE 80 7.926 0.000 10

27 187 MNE 90 6.776 0.000 5

27 187 MNE 100 9.915 9.915 1

27 187 sLORETA 0 31.958 0.000 8002

27 187 sLORETA 10 29.806 0.000 3153

27 187 sLORETA 20 23.957 0.000 1351

27 187 sLORETA 30 17.848 0.000 657

27 187 sLORETA 40 14.916 0.000 410

27 187 sLORETA 50 13.697 0.000 257

27 187 sLORETA 60 12.564 0.000 164

27 187 sLORETA 70 12.150 0.000 81

27 187 sLORETA 80 10.456 0.000 33

27 187 sLORETA 90 11.691 0.000 9

27 187 sLORETA 100 9.915 9.915 1

27 188 Ave 0 35.088 0.000 8002

27 188 Ave 10 32.979 0.000 2477

27 188 Ave 20 29.368 0.000 1036

27 188 Ave 30 26.923 0.000 537

27 188 Ave 40 26.143 0.000 300

27 188 Ave 50 23.203 0.000 131

27 188 Ave 60 21.885 0.000 65

27 188 Ave 70 23.604 0.000 29

27 188 Ave 80 24.493 9.456 11

27 188 Ave 90 28.961 15.945 4

27 188 Ave 100 15.945 15.945 1

27 188 cMEM 0 24.888 0.000 8002

27 188 cMEM 10 23.156 0.000 282

27 188 cMEM 20 21.563 0.000 118

27 188 cMEM 30 21.346 0.000 55

27 188 cMEM 40 20.933 5.152 27

27 188 cMEM 50 20.843 15.548 9

27 188 cMEM 60 20.421 15.548 7

27 188 cMEM 70 21.187 15.945 4

27 188 cMEM 80 20.749 20.749 1

27 188 cMEM 90 20.749 20.749 1

27 188 cMEM 100 20.749 20.749 1

27 188 dSPM 0 34.499 0.000 8002

27 188 dSPM 10 32.615 0.000 2795

27 188 dSPM 20 29.633 0.000 1270

27 188 dSPM 30 27.673 0.000 696

27 188 dSPM 40 26.589 0.000 408

27 188 dSPM 50 26.157 0.000 212

27 188 dSPM 60 23.992 0.000 108

27 188 dSPM 70 24.486 0.000 48

27 188 dSPM 80 20.846 14.954 14

27 188 dSPM 90 18.873 17.230 6

27 188 dSPM 100 17.611 17.611 1

27 188 MNE 0 36.836 0.000 8002

27 188 MNE 10 33.953 0.000 1445

27 188 MNE 20 30.176 0.000 536

27 188 MNE 30 27.643 0.000 237

27 188 MNE 40 25.474 0.000 101

27 188 MNE 50 23.088 0.000 47

27 188 MNE 60 24.592 0.000 22

27 188 MNE 70 29.109 0.000 7

27 188 MNE 80 33.151 30.066 4

27 188 MNE 90 33.543 32.936 2

27 188 MNE 100 34.119 34.119 1

27 188 sLORETA 0 36.531 0.000 8002

27 188 sLORETA 10 35.097 0.000 2926

27 188 sLORETA 20 31.802 0.000 1281

27 188 sLORETA 30 27.974 0.000 651

27 188 sLORETA 40 26.796 0.000 403

27 188 sLORETA 50 25.843 0.000 222

27 188 sLORETA 60 24.464 0.000 98

27 188 sLORETA 70 22.755 0.000 35

27 188 sLORETA 80 26.107 7.524 15

27 188 sLORETA 90 29.785 15.945 5

27 188 sLORETA 100 30.066 30.066 1

27 189 Ave 0 37.308 0.000 8002

27 189 Ave 10 35.261 0.000 3366

27 189 Ave 20 29.469 0.000 1326

27 189 Ave 30 25.283 0.000 602

27 189 Ave 40 21.533 0.000 275

27 189 Ave 50 16.382 0.000 130

27 189 Ave 60 13.397 0.000 76

27 189 Ave 70 9.605 0.000 28

27 189 Ave 80 2.568 0.000 13

27 189 Ave 90 0.000 0.000 9

27 189 Ave 100 0.000 0.000 1

27 189 cMEM 0 13.130 0.000 8002

27 189 cMEM 10 11.800 0.000 384

27 189 cMEM 20 11.111 0.000 210

27 189 cMEM 30 10.642 0.000 125

27 189 cMEM 40 10.896 0.000 81

27 189 cMEM 50 11.344 0.000 56

27 189 cMEM 60 11.779 0.000 43

27 189 cMEM 70 12.566 0.000 30

27 189 cMEM 80 11.899 0.000 14

27 189 cMEM 90 13.422 0.000 4

27 189 cMEM 100 14.963 14.963 1

27 189 dSPM 0 38.101 0.000 8002

27 189 dSPM 10 36.223 0.000 3614

27 189 dSPM 20 31.253 0.000 1564

27 189 dSPM 30 26.711 0.000 667

27 189 dSPM 40 23.121 0.000 288

27 189 dSPM 50 19.946 0.000 120

27 189 dSPM 60 12.163 0.000 45

27 189 dSPM 70 9.808 0.000 26

27 189 dSPM 80 6.511 0.000 13

27 189 dSPM 90 5.075 0.000 6

27 189 dSPM 100 0.000 0.000 1

27 189 MNE 0 41.184 0.000 8002

27 189 MNE 10 39.662 0.000 2555

27 189 MNE 20 36.041 0.000 1081

27 189 MNE 30 32.911 0.000 505

27 189 MNE 40 29.909 0.000 242

27 189 MNE 50 28.855 0.000 120

27 189 MNE 60 24.895 0.000 52

27 189 MNE 70 26.373 0.000 24

27 189 MNE 80 31.232 0.000 13

27 189 MNE 90 29.570 0.000 4

27 189 MNE 100 32.936 32.936 1

27 189 sLORETA 0 38.691 0.000 8002

27 189 sLORETA 10 37.581 0.000 4086

27 189 sLORETA 20 33.783 0.000 1974

27 189 sLORETA 30 30.294 0.000 1045

27 189 sLORETA 40 27.797 0.000 523

27 189 sLORETA 50 23.823 0.000 261

27 189 sLORETA 60 20.840 0.000 131

27 189 sLORETA 70 16.519 0.000 65

27 189 sLORETA 80 15.099 0.000 33

27 189 sLORETA 90 1.462 0.000 12

27 189 sLORETA 100 0.000 0.000 1

27 190 Ave 0 35.461 0.000 8002

27 190 Ave 10 33.120 0.000 2563

27 190 Ave 20 28.015 0.000 989

27 190 Ave 30 25.074 0.000 445

27 190 Ave 40 24.117 0.000 207

27 190 Ave 50 22.102 0.000 93

27 190 Ave 60 22.931 7.524 45

27 190 Ave 70 23.019 9.572 26

27 190 Ave 80 21.586 13.935 12

27 190 Ave 90 20.580 15.945 3

27 190 Ave 100 23.802 23.802 1

27 190 cMEM 0 20.419 0.000 8002

27 190 cMEM 10 19.773 0.000 144

27 190 cMEM 20 18.976 0.000 49

27 190 cMEM 30 19.104 5.152 33

27 190 cMEM 40 19.453 9.456 26

27 190 cMEM 50 19.711 13.935 18

27 190 cMEM 60 20.148 14.780 11

27 190 cMEM 70 19.263 15.548 5

27 190 cMEM 80 21.059 20.749 2

27 190 cMEM 90 20.749 20.749 1

27 190 cMEM 100 20.749 20.749 1

27 190 dSPM 0 34.282 0.000 8002

27 190 dSPM 10 32.186 0.000 2848

27 190 dSPM 20 27.575 0.000 1168

27 190 dSPM 30 25.563 0.000 638

27 190 dSPM 40 24.771 0.000 364

27 190 dSPM 50 23.815 0.000 169

27 190 dSPM 60 23.326 0.000 74

27 190 dSPM 70 22.810 11.459 35

27 190 dSPM 80 21.570 13.900 18

27 190 dSPM 90 21.896 18.935 8

27 190 dSPM 100 24.384 24.384 1

27 190 MNE 0 37.976 0.000 8002

27 190 MNE 10 35.302 0.000 1593

27 190 MNE 20 31.451 0.000 602

27 190 MNE 30 28.378 0.000 232

27 190 MNE 40 26.350 0.000 107

27 190 MNE 50 24.091 0.000 44

27 190 MNE 60 24.636 0.000 24

27 190 MNE 70 29.109 13.935 9

27 190 MNE 80 32.557 30.066 3

27 190 MNE 90 33.592 32.936 2

27 190 MNE 100 34.119 34.119 1

27 190 sLORETA 0 37.427 0.000 8002

27 190 sLORETA 10 36.141 0.000 3222

27 190 sLORETA 20 32.564 0.000 1452

27 190 sLORETA 30 27.516 0.000 696

27 190 sLORETA 40 25.206 0.000 390

27 190 sLORETA 50 24.787 0.000 193

27 190 sLORETA 60 24.279 0.000 88

27 190 sLORETA 70 24.402 0.000 44

27 190 sLORETA 80 26.605 14.954 18

27 190 sLORETA 90 28.126 15.945 6

27 190 sLORETA 100 23.802 23.802 1

27 191 Ave 0 36.820 0.000 8002

27 191 Ave 10 35.749 0.000 3878

27 191 Ave 20 32.429 0.000 1964

27 191 Ave 30 28.649 0.000 1134

27 191 Ave 40 24.568 0.000 602

27 191 Ave 50 21.596 0.000 318

27 191 Ave 60 19.707 0.000 168

27 191 Ave 70 18.853 0.000 84

27 191 Ave 80 18.110 0.000 46

27 191 Ave 90 15.987 0.000 7

27 191 Ave 100 12.684 12.684 1

27 191 cMEM 0 13.074 0.000 8002

27 191 cMEM 10 11.936 0.000 427

27 191 cMEM 20 11.077 0.000 228

27 191 cMEM 30 9.812 0.000 127

27 191 cMEM 40 7.102 0.000 60

27 191 cMEM 50 3.899 0.000 26

27 191 cMEM 60 3.015 0.000 12

27 191 cMEM 70 3.403 0.000 8

27 191 cMEM 80 0.000 0.000 5

27 191 cMEM 90 0.000 0.000 3

27 191 cMEM 100 0.000 0.000 1

27 191 dSPM 0 34.535 0.000 8002

27 191 dSPM 10 32.728 0.000 3247

27 191 dSPM 20 27.545 0.000 1422

27 191 dSPM 30 23.066 0.000 695

27 191 dSPM 40 20.352 0.000 355

27 191 dSPM 50 18.266 0.000 184

27 191 dSPM 60 18.596 0.000 82

27 191 dSPM 70 19.486 0.000 32

27 191 dSPM 80 19.651 4.234 13

27 191 dSPM 90 22.129 16.268 5

27 191 dSPM 100 22.683 22.683 1

27 191 MNE 0 38.735 0.000 8002

27 191 MNE 10 37.124 0.000 2466

27 191 MNE 20 33.586 0.000 1007

27 191 MNE 30 30.240 0.000 474

27 191 MNE 40 27.826 0.000 235

27 191 MNE 50 26.759 0.000 133

27 191 MNE 60 24.787 0.000 61

27 191 MNE 70 24.646 0.000 33

27 191 MNE 80 23.707 0.000 17

27 191 MNE 90 12.626 0.000 6

27 191 MNE 100 0.000 0.000 1

27 191 sLORETA 0 38.113 0.000 8002

27 191 sLORETA 10 37.247 0.000 3987

27 191 sLORETA 20 34.848 0.000 2205

27 191 sLORETA 30 31.765 0.000 1304

27 191 sLORETA 40 27.898 0.000 723

27 191 sLORETA 50 22.200 0.000 358

27 191 sLORETA 60 19.436 0.000 195

27 191 sLORETA 70 18.296 0.000 101

27 191 sLORETA 80 18.565 0.000 49

27 191 sLORETA 90 19.565 0.000 15

27 191 sLORETA 100 26.312 26.312 1

27 192 Ave 0 32.679 0.000 8002

27 192 Ave 10 30.480 0.000 2129

27 192 Ave 20 28.493 0.000 916

27 192 Ave 30 27.095 0.000 458

27 192 Ave 40 24.726 0.000 223

27 192 Ave 50 23.883 0.000 92

27 192 Ave 60 23.995 5.152 50

27 192 Ave 70 25.419 9.456 27

27 192 Ave 80 27.140 13.935 13

27 192 Ave 90 27.300 15.945 6

27 192 Ave 100 23.802 23.802 1

27 192 cMEM 0 20.903 0.000 8002

27 192 cMEM 10 19.940 0.000 202

27 192 cMEM 20 19.291 0.000 71

27 192 cMEM 30 18.487 0.000 44

27 192 cMEM 40 18.671 5.152 28

27 192 cMEM 50 18.927 5.152 19

27 192 cMEM 60 19.268 5.152 12

27 192 cMEM 70 19.243 15.548 5

27 192 cMEM 80 20.749 20.749 1

27 192 cMEM 90 20.749 20.749 1

27 192 cMEM 100 20.749 20.749 1

27 192 dSPM 0 32.490 0.000 8002

27 192 dSPM 10 30.858 0.000 2599

27 192 dSPM 20 28.884 0.000 1232

27 192 dSPM 30 27.847 0.000 720

27 192 dSPM 40 25.903 0.000 413

27 192 dSPM 50 25.457 0.000 209

27 192 dSPM 60 24.251 0.000 98

27 192 dSPM 70 25.380 0.000 47

27 192 dSPM 80 24.281 13.900 23

27 192 dSPM 90 23.874 17.230 7

27 192 dSPM 100 25.325 25.325 1

27 192 MNE 0 35.246 0.000 8002

27 192 MNE 10 32.704 0.000 1226

27 192 MNE 20 31.084 0.000 445

27 192 MNE 30 29.792 0.000 174

27 192 MNE 40 27.930 0.000 72

27 192 MNE 50 26.783 0.000 30

27 192 MNE 60 29.976 9.456 12

27 192 MNE 70 33.282 30.066 6

27 192 MNE 80 33.166 30.066 4

27 192 MNE 90 33.576 32.936 2

27 192 MNE 100 34.119 34.119 1

27 192 sLORETA 0 33.703 0.000 8002

27 192 sLORETA 10 31.869 0.000 2416

27 192 sLORETA 20 29.317 0.000 999

27 192 sLORETA 30 28.184 0.000 549

27 192 sLORETA 40 26.183 0.000 294

27 192 sLORETA 50 25.347 0.000 130

27 192 sLORETA 60 25.523 0.000 55

27 192 sLORETA 70 28.723 13.935 21

27 192 sLORETA 80 30.295 23.802 10

27 192 sLORETA 90 31.445 23.802 6

27 192 sLORETA 100 30.066 30.066 1

27 193 Ave 0 34.484 0.000 8002

27 193 Ave 10 31.874 0.000 3030

27 193 Ave 20 23.499 0.000 1026

27 193 Ave 30 16.654 0.000 498

27 193 Ave 40 13.276 0.000 270

27 193 Ave 50 10.176 0.000 141

27 193 Ave 60 6.425 0.000 78

27 193 Ave 70 5.474 0.000 47

27 193 Ave 80 4.551 0.000 22

27 193 Ave 90 5.625 0.000 11

27 193 Ave 100 0.000 0.000 1

27 193 cMEM 0 10.804 0.000 8002

27 193 cMEM 10 9.344 0.000 412

27 193 cMEM 20 7.388 0.000 205

27 193 cMEM 30 5.231 0.000 117

27 193 cMEM 40 5.007 0.000 78

27 193 cMEM 50 4.990 0.000 55

27 193 cMEM 60 5.062 0.000 33

27 193 cMEM 70 4.423 0.000 16

27 193 cMEM 80 3.535 0.000 5

27 193 cMEM 90 0.000 0.000 2

27 193 cMEM 100 0.000 0.000 1

27 193 dSPM 0 33.863 0.000 8002

27 193 dSPM 10 30.172 0.000 2535

27 193 dSPM 20 20.989 0.000 814

27 193 dSPM 30 18.026 0.000 395

27 193 dSPM 40 16.819 0.000 204

27 193 dSPM 50 15.765 0.000 100

27 193 dSPM 60 16.129 0.000 51

27 193 dSPM 70 15.584 0.000 26

27 193 dSPM 80 15.240 6.833 10

27 193 dSPM 90 11.760 7.561 4

27 193 dSPM 100 7.561 7.561 1

27 193 MNE 0 40.063 0.000 8002

27 193 MNE 10 36.229 0.000 1492

27 193 MNE 20 27.654 0.000 427

27 193 MNE 30 21.211 0.000 167

27 193 MNE 40 17.961 0.000 64

27 193 MNE 50 14.884 0.000 25

27 193 MNE 60 8.136 0.000 9

27 193 MNE 70 0.000 0.000 3

27 193 MNE 80 0.000 0.000 1

27 193 MNE 90 0.000 0.000 1

27 193 MNE 100 0.000 0.000 1

27 193 sLORETA 0 37.149 0.000 8002

27 193 sLORETA 10 35.474 0.000 3468

27 193 sLORETA 20 29.715 0.000 1374

27 193 sLORETA 30 21.912 0.000 634

27 193 sLORETA 40 16.830 0.000 353

27 193 sLORETA 50 13.755 0.000 178

27 193 sLORETA 60 9.153 0.000 94

27 193 sLORETA 70 5.453 0.000 46

27 193 sLORETA 80 4.391 0.000 20

27 193 sLORETA 90 2.885 0.000 6

27 193 sLORETA 100 2.961 2.961 1

27 194 Ave 0 31.518 0.000 8002

27 194 Ave 10 28.780 0.000 2133

27 194 Ave 20 24.294 0.000 882

27 194 Ave 30 22.952 0.000 484

27 194 Ave 40 20.241 0.000 254

27 194 Ave 50 16.518 0.000 114

27 194 Ave 60 18.099 0.000 43

27 194 Ave 70 18.301 0.000 20

27 194 Ave 80 14.033 5.359 8

27 194 Ave 90 15.945 15.945 1

27 194 Ave 100 15.945 15.945 1

27 194 cMEM 0 21.097 0.000 8002

27 194 cMEM 10 21.094 0.000 264

27 194 cMEM 20 22.335 0.000 100

27 194 cMEM 30 23.519 0.000 41

27 194 cMEM 40 24.652 5.152 23

27 194 cMEM 50 24.924 15.945 16

27 194 cMEM 60 25.588 15.945 9

27 194 cMEM 70 22.003 15.945 4

27 194 cMEM 80 22.193 20.749 2

27 194 cMEM 90 20.749 20.749 1

27 194 cMEM 100 20.749 20.749 1

27 194 dSPM 0 31.274 0.000 8002

27 194 dSPM 10 29.219 0.000 2596

27 194 dSPM 20 25.727 0.000 1187

27 194 dSPM 30 24.148 0.000 695

27 194 dSPM 40 22.858 0.000 423

27 194 dSPM 50 21.444 0.000 230

27 194 dSPM 60 19.377 0.000 114

27 194 dSPM 70 19.524 0.000 49

27 194 dSPM 80 18.303 0.000 15

27 194 dSPM 90 18.682 14.954 8

27 194 dSPM 100 17.611 17.611 1

27 194 MNE 0 33.349 0.000 8002

27 194 MNE 10 31.040 0.000 1629

27 194 MNE 20 27.465 0.000 715

27 194 MNE 30 24.874 0.000 347

27 194 MNE 40 23.424 0.000 189

27 194 MNE 50 21.077 0.000 95

27 194 MNE 60 19.177 0.000 56

27 194 MNE 70 18.534 0.000 33

27 194 MNE 80 20.749 0.000 12

27 194 MNE 90 24.714 5.359 4

27 194 MNE 100 32.936 32.936 1

27 194 sLORETA 0 33.118 0.000 8002

27 194 sLORETA 10 31.424 0.000 2746

27 194 sLORETA 20 27.308 0.000 1182

27 194 sLORETA 30 24.092 0.000 659

27 194 sLORETA 40 23.279 0.000 414

27 194 sLORETA 50 20.762 0.000 236

27 194 sLORETA 60 17.278 0.000 128

27 194 sLORETA 70 17.348 0.000 46

27 194 sLORETA 80 18.314 0.000 17

27 194 sLORETA 90 12.667 7.524 4

27 194 sLORETA 100 15.945 15.945 1

27 195 Ave 0 32.093 0.000 8002

27 195 Ave 10 30.152 0.000 3001

27 195 Ave 20 25.718 0.000 1349

27 195 Ave 30 22.026 0.000 827

27 195 Ave 40 20.686 0.000 580

27 195 Ave 50 19.374 0.000 373

27 195 Ave 60 17.281 0.000 226

27 195 Ave 70 12.326 0.000 101

27 195 Ave 80 8.251 0.000 35

27 195 Ave 90 8.825 0.000 14

27 195 Ave 100 8.017 8.017 1

27 195 cMEM 0 17.746 0.000 8002

27 195 cMEM 10 16.919 0.000 500

27 195 cMEM 20 15.476 0.000 247

27 195 cMEM 30 14.052 0.000 163

27 195 cMEM 40 12.568 0.000 103

27 195 cMEM 50 12.349 0.000 66

27 195 cMEM 60 11.977 0.000 36

27 195 cMEM 70 11.458 0.000 21

27 195 cMEM 80 10.974 5.442 10

27 195 cMEM 90 10.832 8.472 3

27 195 cMEM 100 8.472 8.472 1

27 195 dSPM 0 29.997 0.000 8002

27 195 dSPM 10 26.463 0.000 1973

27 195 dSPM 20 22.473 0.000 884

27 195 dSPM 30 21.412 0.000 533

27 195 dSPM 40 20.842 0.000 336

27 195 dSPM 50 20.438 0.000 203

27 195 dSPM 60 20.360 0.000 116

27 195 dSPM 70 19.872 5.631 58

27 195 dSPM 80 18.323 6.833 26

27 195 dSPM 90 14.660 6.833 6

27 195 dSPM 100 7.561 7.561 1

27 195 MNE 0 35.910 0.000 8002

27 195 MNE 10 33.349 0.000 2050

27 195 MNE 20 27.843 0.000 758

27 195 MNE 30 23.666 0.000 404

27 195 MNE 40 22.390 0.000 246

27 195 MNE 50 21.133 0.000 144

27 195 MNE 60 20.060 0.000 70

27 195 MNE 70 17.168 0.000 20

27 195 MNE 80 18.401 0.000 7

27 195 MNE 90 8.902 0.000 3

27 195 MNE 100 0.000 0.000 1

27 195 sLORETA 0 32.978 0.000 8002

27 195 sLORETA 10 31.636 0.000 3495

27 195 sLORETA 20 27.902 0.000 1668

27 195 sLORETA 30 24.675 0.000 1062

27 195 sLORETA 40 21.590 0.000 692

27 195 sLORETA 50 20.496 0.000 494

27 195 sLORETA 60 19.096 0.000 333

27 195 sLORETA 70 16.884 0.000 183

27 195 sLORETA 80 11.536 0.000 82

27 195 sLORETA 90 8.230 0.000 36

27 195 sLORETA 100 6.615 6.615 1

27 196 Ave 0 32.376 0.000 8002

27 196 Ave 10 29.646 0.000 2039

27 196 Ave 20 26.667 0.000 895

27 196 Ave 30 25.613 0.000 480

27 196 Ave 40 23.500 0.000 248

27 196 Ave 50 21.273 0.000 112

27 196 Ave 60 22.749 0.000 52

27 196 Ave 70 22.841 7.524 30

27 196 Ave 80 22.522 14.954 14

27 196 Ave 90 19.489 15.945 4

27 196 Ave 100 15.945 15.945 1

27 196 cMEM 0 23.529 0.000 8002

27 196 cMEM 10 23.338 0.000 228

27 196 cMEM 20 23.189 0.000 109

27 196 cMEM 30 22.717 5.152 51

27 196 cMEM 40 22.609 14.408 35

27 196 cMEM 50 23.046 14.408 20

27 196 cMEM 60 21.814 15.945 9

27 196 cMEM 70 19.835 15.945 4

27 196 cMEM 80 19.290 16.916 2

27 196 cMEM 90 20.749 20.749 1

27 196 cMEM 100 20.749 20.749 1

27 196 dSPM 0 31.764 0.000 8002

27 196 dSPM 10 29.341 0.000 2257

27 196 dSPM 20 26.950 0.000 1109

27 196 dSPM 30 25.968 0.000 630

27 196 dSPM 40 24.991 0.000 349

27 196 dSPM 50 23.313 0.000 175

27 196 dSPM 60 21.633 0.000 81

27 196 dSPM 70 21.248 0.000 34

27 196 dSPM 80 21.204 14.954 17

27 196 dSPM 90 20.176 17.230 9

27 196 dSPM 100 18.935 18.935 1

27 196 MNE 0 34.645 0.000 8002

27 196 MNE 10 31.837 0.000 1428

27 196 MNE 20 29.234 0.000 583

27 196 MNE 30 27.328 0.000 283

27 196 MNE 40 26.558 0.000 144

27 196 MNE 50 23.694 0.000 63

27 196 MNE 60 23.010 0.000 37

27 196 MNE 70 25.390 9.456 15

27 196 MNE 80 33.126 30.066 4

27 196 MNE 90 33.558 32.936 2

27 196 MNE 100 34.119 34.119 1

27 196 sLORETA 0 33.758 0.000 8002

27 196 sLORETA 10 31.899 0.000 2671

27 196 sLORETA 20 27.963 0.000 1160

27 196 sLORETA 30 26.395 0.000 647

27 196 sLORETA 40 25.704 0.000 381

27 196 sLORETA 50 24.257 0.000 214

27 196 sLORETA 60 21.004 0.000 104

27 196 sLORETA 70 22.785 0.000 47

27 196 sLORETA 80 24.361 7.524 24

27 196 sLORETA 90 23.788 15.945 3

27 196 sLORETA 100 15.945 15.945 1

27 197 Ave 0 30.136 0.000 8002

27 197 Ave 10 27.681 0.000 2932

27 197 Ave 20 22.464 0.000 1256

27 197 Ave 30 18.780 0.000 726

27 197 Ave 40 17.279 0.000 503

27 197 Ave 50 14.862 0.000 293

27 197 Ave 60 11.683 0.000 154

27 197 Ave 70 7.729 0.000 80

27 197 Ave 80 6.829 0.000 46

27 197 Ave 90 4.227 0.000 16

27 197 Ave 100 4.620 4.620 1

27 197 cMEM 0 12.258 0.000 8002

27 197 cMEM 10 11.152 0.000 510

27 197 cMEM 20 10.357 0.000 281

27 197 cMEM 30 8.801 0.000 180

27 197 cMEM 40 6.214 0.000 100

27 197 cMEM 50 3.413 0.000 50

27 197 cMEM 60 2.913 0.000 32

27 197 cMEM 70 3.077 0.000 17

27 197 cMEM 80 2.566 0.000 10

27 197 cMEM 90 4.167 0.000 2

27 197 cMEM 100 5.777 5.777 1

27 197 dSPM 0 29.237 0.000 8002

27 197 dSPM 10 24.959 0.000 1991

27 197 dSPM 20 20.675 0.000 814

27 197 dSPM 30 19.524 0.000 453

27 197 dSPM 40 19.089 0.000 267

27 197 dSPM 50 18.810 0.000 153

27 197 dSPM 60 18.792 0.000 74

27 197 dSPM 70 18.156 6.833 33

27 197 dSPM 80 17.257 6.833 18

27 197 dSPM 90 11.567 7.561 3

27 197 dSPM 100 7.561 7.561 1

27 197 MNE 0 36.049 0.000 8002

27 197 MNE 10 32.588 0.000 1726

27 197 MNE 20 26.465 0.000 594

27 197 MNE 30 20.739 0.000 269

27 197 MNE 40 17.360 0.000 129

27 197 MNE 50 13.929 0.000 49

27 197 MNE 60 5.992 0.000 17

27 197 MNE 70 7.585 0.000 5

27 197 MNE 80 0.000 0.000 2

27 197 MNE 90 0.000 0.000 1

27 197 MNE 100 0.000 0.000 1

27 197 sLORETA 0 31.690 0.000 8002

27 197 sLORETA 10 29.875 0.000 3354

27 197 sLORETA 20 25.502 0.000 1553

27 197 sLORETA 30 21.331 0.000 880

27 197 sLORETA 40 19.189 0.000 585

27 197 sLORETA 50 17.554 0.000 415

27 197 sLORETA 60 14.952 0.000 245

27 197 sLORETA 70 11.156 0.000 119

27 197 sLORETA 80 9.127 0.000 48

27 197 sLORETA 90 4.833 0.000 17

27 197 sLORETA 100 0.000 0.000 1

27 198 Ave 0 32.886 0.000 8002

27 198 Ave 10 30.583 0.000 2660

27 198 Ave 20 26.634 0.000 1096

27 198 Ave 30 25.260 0.000 617

27 198 Ave 40 24.538 0.000 376

27 198 Ave 50 22.306 0.000 198

27 198 Ave 60 20.007 0.000 111

27 198 Ave 70 19.899 0.000 54

27 198 Ave 80 21.193 0.000 25

27 198 Ave 90 25.602 8.124 10

27 198 Ave 100 34.119 34.119 1

27 198 cMEM 0 19.745 0.000 8002

27 198 cMEM 10 19.241 0.000 392

27 198 cMEM 20 19.130 0.000 253

27 198 cMEM 30 18.701 0.000 173

27 198 cMEM 40 18.713 0.000 102

27 198 cMEM 50 18.834 0.000 55

27 198 cMEM 60 21.602 0.000 25

27 198 cMEM 70 22.962 5.152 9

27 198 cMEM 80 21.593 5.152 8

27 198 cMEM 90 7.668 5.152 2

27 198 cMEM 100 9.456 9.456 1

27 198 dSPM 0 32.253 0.000 8002

27 198 dSPM 10 29.836 0.000 2733

27 198 dSPM 20 26.519 0.000 1194

27 198 dSPM 30 25.076 0.000 690

27 198 dSPM 40 24.395 0.000 424

27 198 dSPM 50 22.249 0.000 231

27 198 dSPM 60 20.547 0.000 107

27 198 dSPM 70 18.204 0.000 48

27 198 dSPM 80 20.608 0.000 19

27 198 dSPM 90 24.488 18.935 4

27 198 dSPM 100 25.325 25.325 1

27 198 MNE 0 36.044 0.000 8002

27 198 MNE 10 33.269 0.000 1604

27 198 MNE 20 29.980 0.000 633

27 198 MNE 30 28.540 0.000 301

27 198 MNE 40 27.870 0.000 154

27 198 MNE 50 25.257 0.000 76

27 198 MNE 60 24.472 0.000 38

27 198 MNE 70 24.642 0.000 20

27 198 MNE 80 25.664 0.000 10

27 198 MNE 90 29.269 13.935 4

27 198 MNE 100 34.119 34.119 1

27 198 sLORETA 0 34.077 0.000 8002

27 198 sLORETA 10 32.326 0.000 3001

27 198 sLORETA 20 28.209 0.000 1266

27 198 sLORETA 30 26.259 0.000 708

27 198 sLORETA 40 25.350 0.000 440

27 198 sLORETA 50 24.246 0.000 264

27 198 sLORETA 60 21.555 0.000 136

27 198 sLORETA 70 19.159 0.000 74

27 198 sLORETA 80 21.096 0.000 32

27 198 sLORETA 90 25.841 13.562 12

27 198 sLORETA 100 23.802 23.802 1

27 199 Ave 0 34.138 0.000 8002

27 199 Ave 10 31.867 0.000 2948

27 199 Ave 20 25.344 0.000 1094

27 199 Ave 30 21.144 0.000 613

27 199 Ave 40 18.148 0.000 381

27 199 Ave 50 16.711 0.000 253

27 199 Ave 60 14.990 0.000 128

27 199 Ave 70 12.464 0.000 39

27 199 Ave 80 9.999 0.000 7

27 199 Ave 90 0.000 0.000 1

27 199 Ave 100 0.000 0.000 1

27 199 cMEM 0 13.135 0.000 8002

27 199 cMEM 10 12.558 0.000 445

27 199 cMEM 20 12.095 0.000 295

27 199 cMEM 30 11.452 0.000 194

27 199 cMEM 40 10.190 0.000 112

27 199 cMEM 50 6.952 0.000 40

27 199 cMEM 60 2.611 0.000 19

27 199 cMEM 70 0.000 0.000 10

27 199 cMEM 80 0.000 0.000 6

27 199 cMEM 90 0.000 0.000 4

27 199 cMEM 100 0.000 0.000 1

27 199 dSPM 0 32.085 0.000 8002

27 199 dSPM 10 29.251 0.000 2768

27 199 dSPM 20 22.635 0.000 950

27 199 dSPM 30 19.572 0.000 557

27 199 dSPM 40 18.532 0.000 374

27 199 dSPM 50 18.104 0.000 239

27 199 dSPM 60 17.821 0.000 140

27 199 dSPM 70 18.467 0.000 72

27 199 dSPM 80 18.990 6.833 30

27 199 dSPM 90 18.620 7.561 7

27 199 dSPM 100 7.561 7.561 1

27 199 MNE 0 38.221 0.000 8002

27 199 MNE 10 35.151 0.000 1539

27 199 MNE 20 28.284 0.000 530

27 199 MNE 30 23.179 0.000 236

27 199 MNE 40 18.760 0.000 104

27 199 MNE 50 16.142 0.000 51

27 199 MNE 60 14.043 0.000 14

27 199 MNE 70 13.262 0.000 4

27 199 MNE 80 5.111 0.000 2

27 199 MNE 90 0.000 0.000 1

27 199 MNE 100 0.000 0.000 1

27 199 sLORETA 0 33.841 0.000 8002

27 199 sLORETA 10 31.800 0.000 3033

27 199 sLORETA 20 26.312 0.000 1228

27 199 sLORETA 30 21.640 0.000 656

27 199 sLORETA 40 19.366 0.000 421

27 199 sLORETA 50 18.580 0.000 274

27 199 sLORETA 60 16.689 0.000 171

27 199 sLORETA 70 13.226 0.000 82

27 199 sLORETA 80 12.300 0.000 28

27 199 sLORETA 90 9.667 0.000 6

27 199 sLORETA 100 0.000 0.000 1

28 200 Ave 0 37.802 0.000 8002

28 200 Ave 10 37.255 0.000 4965

28 200 Ave 20 34.607 0.000 2702

28 200 Ave 30 30.924 0.000 1394

28 200 Ave 40 26.579 0.000 693

28 200 Ave 50 22.600 0.000 379

28 200 Ave 60 18.648 0.000 177

28 200 Ave 70 14.691 0.000 80

28 200 Ave 80 13.523 0.000 29

28 200 Ave 90 9.065 0.000 6

28 200 Ave 100 0.000 0.000 1

28 200 cMEM 0 26.950 0.000 8002

28 200 cMEM 10 26.373 0.000 691

28 200 cMEM 20 24.288 3.070 232

28 200 cMEM 30 21.453 3.070 110

28 200 cMEM 40 18.750 4.389 42

28 200 cMEM 50 15.106 4.389 18

28 200 cMEM 60 13.852 4.389 12

28 200 cMEM 70 14.097 4.389 9

28 200 cMEM 80 17.828 16.929 2

28 200 cMEM 90 16.929 16.929 1

28 200 cMEM 100 16.929 16.929 1

28 200 dSPM 0 33.322 0.000 8002

28 200 dSPM 10 31.170 0.000 3322

28 200 dSPM 20 24.640 0.000 1239

28 200 dSPM 30 18.365 0.000 495

28 200 dSPM 40 12.374 0.000 190

28 200 dSPM 50 10.621 0.000 98

28 200 dSPM 60 8.631 0.000 63

28 200 dSPM 70 7.221 0.000 32

28 200 dSPM 80 7.266 0.000 15

28 200 dSPM 90 4.389 0.000 2

28 200 dSPM 100 0.000 0.000 1

28 200 MNE 0 42.965 0.000 8002

28 200 MNE 10 42.054 0.000 2915

28 200 MNE 20 40.134 0.000 1203

28 200 MNE 30 38.670 0.000 498

28 200 MNE 40 36.230 0.000 174

28 200 MNE 50 33.916 0.000 75

28 200 MNE 60 28.790 5.217 30

28 200 MNE 70 28.386 5.217 13

28 200 MNE 80 28.405 9.578 6

28 200 MNE 90 36.153 9.578 3

28 200 MNE 100 9.578 9.578 1

28 200 sLORETA 0 38.937 0.000 8002

28 200 sLORETA 10 38.584 0.000 5337

28 200 sLORETA 20 36.623 0.000 3182

28 200 sLORETA 30 33.678 0.000 1753

28 200 sLORETA 40 30.437 0.000 970

28 200 sLORETA 50 26.264 0.000 513

28 200 sLORETA 60 24.673 0.000 271

28 200 sLORETA 70 23.274 0.000 145

28 200 sLORETA 80 17.968 0.000 44

28 200 sLORETA 90 15.257 0.000 9

28 200 sLORETA 100 6.378 6.378 1

28 201 Ave 0 41.772 0.000 8002

28 201 Ave 10 41.322 0.000 4796

28 201 Ave 20 39.790 0.000 2576

28 201 Ave 30 37.826 0.000 1322

28 201 Ave 40 37.074 0.000 638

28 201 Ave 50 36.405 0.000 303

28 201 Ave 60 35.767 4.463 167

28 201 Ave 70 34.757 7.792 85

28 201 Ave 80 32.394 7.792 35

28 201 Ave 90 32.204 12.319 9

28 201 Ave 100 29.132 29.132 1

28 201 cMEM 0 39.867 0.000 8002

28 201 cMEM 10 39.832 0.000 724

28 201 cMEM 20 39.833 0.000 329

28 201 cMEM 30 40.060 1.674 189

28 201 cMEM 40 40.038 19.822 111

28 201 cMEM 50 39.728 20.831 62

28 201 cMEM 60 38.185 22.894 31

28 201 cMEM 70 36.679 30.588 16

28 201 cMEM 80 35.683 30.588 10

28 201 cMEM 90 36.460 32.852 4

28 201 cMEM 100 33.061 33.061 1

28 201 dSPM 0 39.068 0.000 8002

28 201 dSPM 10 38.409 0.000 4375

28 201 dSPM 20 36.443 0.000 2250

28 201 dSPM 30 34.351 0.000 1198

28 201 dSPM 40 31.607 0.000 581

28 201 dSPM 50 27.710 0.000 285

28 201 dSPM 60 24.589 0.000 141

28 201 dSPM 70 23.604 0.000 63

28 201 dSPM 80 20.385 2.047 16

28 201 dSPM 90 20.781 4.463 6

28 201 dSPM 100 7.065 7.065 1

28 201 MNE 0 46.419 0.000 8002

28 201 MNE 10 45.649 0.000 2130

28 201 MNE 20 45.305 0.000 589

28 201 MNE 30 45.904 5.217 203

28 201 MNE 40 49.056 9.955 81

28 201 MNE 50 54.357 18.986 25

28 201 MNE 60 58.740 51.667 7

28 201 MNE 70 59.766 58.158 3

28 201 MNE 80 58.158 58.158 1

28 201 MNE 90 58.158 58.158 1

28 201 MNE 100 58.158 58.158 1

28 201 sLORETA 0 42.531 0.000 8002

28 201 sLORETA 10 41.953 0.000 4355

28 201 sLORETA 20 40.398 0.000 2045

28 201 sLORETA 30 38.676 0.000 932

28 201 sLORETA 40 37.888 2.047 413

28 201 sLORETA 50 35.545 7.792 206

28 201 sLORETA 60 31.833 7.792 102

28 201 sLORETA 70 29.035 7.792 64

28 201 sLORETA 80 26.069 7.792 30

28 201 sLORETA 90 26.793 12.319 10

28 201 sLORETA 100 28.542 28.542 1

28 202 Ave 0 48.988 0.000 8002

28 202 Ave 10 48.930 0.000 5232

28 202 Ave 20 48.536 0.000 3149

28 202 Ave 30 47.738 0.000 1660

28 202 Ave 40 46.683 0.000 836

28 202 Ave 50 44.742 3.210 436

28 202 Ave 60 41.631 4.463 230

28 202 Ave 70 38.879 7.792 114

28 202 Ave 80 39.598 7.792 51

28 202 Ave 90 46.528 29.132 12

28 202 Ave 100 36.273 36.273 1

28 202 cMEM 0 43.290 0.000 8002

28 202 cMEM 10 42.653 1.674 545

28 202 cMEM 20 40.619 1.674 217

28 202 cMEM 30 39.686 8.813 125

28 202 cMEM 40 38.870 9.797 78

28 202 cMEM 50 39.040 11.364 49

28 202 cMEM 60 39.287 14.270 30

28 202 cMEM 70 40.826 30.699 17

28 202 cMEM 80 41.344 34.397 8

28 202 cMEM 90 42.183 42.174 2

28 202 cMEM 100 42.190 42.190 1

28 202 dSPM 0 46.206 0.000 8002

28 202 dSPM 10 45.930 0.000 4331

28 202 dSPM 20 44.979 0.000 2223

28 202 dSPM 30 43.456 0.000 1062

28 202 dSPM 40 40.787 0.000 501

28 202 dSPM 50 38.525 0.000 222

28 202 dSPM 60 38.956 0.000 84

28 202 dSPM 70 36.671 4.463 29

28 202 dSPM 80 32.294 4.463 11

28 202 dSPM 90 27.686 25.979 2

28 202 dSPM 100 25.979 25.979 1

28 202 MNE 0 52.923 0.000 8002

28 202 MNE 10 53.017 0.000 3461

28 202 MNE 20 53.012 0.000 1421

28 202 MNE 30 52.909 3.070 646

28 202 MNE 40 53.337 6.408 291

28 202 MNE 50 54.448 8.917 132

28 202 MNE 60 53.622 9.578 56

28 202 MNE 70 53.552 9.578 25

28 202 MNE 80 55.459 29.148 12

28 202 MNE 90 64.754 57.334 2

28 202 MNE 100 57.334 57.334 1

28 202 sLORETA 0 49.040 0.000 8002

28 202 sLORETA 10 48.996 0.000 5037

28 202 sLORETA 20 48.505 0.000 2864

28 202 sLORETA 30 47.346 0.000 1379

28 202 sLORETA 40 45.349 0.000 641

28 202 sLORETA 50 41.354 3.210 300

28 202 sLORETA 60 35.098 7.792 146

28 202 sLORETA 70 33.584 7.792 83

28 202 sLORETA 80 28.171 7.792 37

28 202 sLORETA 90 28.164 11.654 12

28 202 sLORETA 100 24.804 24.804 1

28 203 Ave 0 46.835 0.000 8002

28 203 Ave 10 46.683 0.000 4979

28 203 Ave 20 45.816 0.000 2736

28 203 Ave 30 43.507 0.000 1356

28 203 Ave 40 40.482 0.000 639

28 203 Ave 50 36.789 1.956 284

28 203 Ave 60 32.455 7.792 113

28 203 Ave 70 26.978 7.792 51

28 203 Ave 80 27.111 11.140 20

28 203 Ave 90 29.950 28.542 5

28 203 Ave 100 29.132 29.132 1

28 203 cMEM 0 51.675 0.000 8002

28 203 cMEM 10 51.668 5.217 407

28 203 cMEM 20 52.373 9.705 162

28 203 cMEM 30 54.087 33.061 90

28 203 cMEM 40 53.554 38.037 56

28 203 cMEM 50 51.624 38.037 27

28 203 cMEM 60 47.074 43.538 9

28 203 cMEM 70 44.716 44.430 2

28 203 cMEM 80 44.716 44.430 2

28 203 cMEM 90 44.716 44.430 2

28 203 cMEM 100 44.954 44.954 1

28 203 dSPM 0 44.593 0.000 8002

28 203 dSPM 10 44.430 0.000 5095

28 203 dSPM 20 43.506 0.000 3001

28 203 dSPM 30 42.418 0.000 1772

28 203 dSPM 40 40.783 0.000 1030

28 203 dSPM 50 38.688 0.000 603

28 203 dSPM 60 37.084 0.000 342

28 203 dSPM 70 35.197 0.000 196

28 203 dSPM 80 32.702 3.203 82

28 203 dSPM 90 32.677 11.140 23

28 203 dSPM 100 32.559 32.559 1

28 203 MNE 0 50.583 0.000 8002

28 203 MNE 10 50.509 0.000 3697

28 203 MNE 20 49.825 1.956 1711

28 203 MNE 30 48.517 7.792 770

28 203 MNE 40 47.448 7.792 330

28 203 MNE 50 47.110 8.791 163

28 203 MNE 60 47.252 13.765 64

28 203 MNE 70 50.548 23.085 25

28 203 MNE 80 53.075 23.085 10

28 203 MNE 90 56.429 51.667 4

28 203 MNE 100 51.667 51.667 1

28 203 sLORETA 0 46.179 0.000 8002

28 203 sLORETA 10 45.995 0.000 4533

28 203 sLORETA 20 44.324 0.000 2195

28 203 sLORETA 30 41.429 0.000 980

28 203 sLORETA 40 38.000 1.956 464

28 203 sLORETA 50 33.436 7.792 194

28 203 sLORETA 60 28.390 7.792 94

28 203 sLORETA 70 27.319 7.792 53

28 203 sLORETA 80 27.818 11.140 29

28 203 sLORETA 90 28.006 12.319 6

28 203 sLORETA 100 30.338 30.338 1

28 204 Ave 0 43.404 0.000 8002

28 204 Ave 10 43.213 0.000 5511

28 204 Ave 20 42.231 0.000 3523

28 204 Ave 30 40.532 0.000 2084

28 204 Ave 40 38.847 0.000 1153

28 204 Ave 50 37.408 0.000 646

28 204 Ave 60 34.971 0.000 321

28 204 Ave 70 33.187 0.000 150

28 204 Ave 80 35.147 8.848 35

28 204 Ave 90 27.156 15.217 5

28 204 Ave 100 18.543 18.543 1

28 204 cMEM 0 38.508 0.000 8002

28 204 cMEM 10 37.856 2.382 885

28 204 cMEM 20 36.642 3.780 335

28 204 cMEM 30 36.568 3.780 141

28 204 cMEM 40 37.311 3.780 88

28 204 cMEM 50 38.471 3.780 55

28 204 cMEM 60 38.489 10.037 29

28 204 cMEM 70 44.162 11.151 13

28 204 cMEM 80 49.234 13.302 5

28 204 cMEM 90 54.316 54.316 1

28 204 cMEM 100 54.316 54.316 1

28 204 dSPM 0 40.092 0.000 8002

28 204 dSPM 10 39.776 0.000 5332

28 204 dSPM 20 38.137 0.000 3214

28 204 dSPM 30 35.807 0.000 1828

28 204 dSPM 40 33.601 0.000 1015

28 204 dSPM 50 30.423 0.000 546

28 204 dSPM 60 26.695 0.000 301

28 204 dSPM 70 25.227 0.000 143

28 204 dSPM 80 26.221 0.000 45

28 204 dSPM 90 30.860 0.000 15

28 204 dSPM 100 49.023 49.023 1

28 204 MNE 0 48.614 0.000 8002

28 204 MNE 10 48.453 0.000 3187

28 204 MNE 20 47.892 0.000 1330

28 204 MNE 30 47.409 4.908 546

28 204 MNE 40 46.396 9.141 234

28 204 MNE 50 45.555 9.526 86

28 204 MNE 60 40.493 12.679 35

28 204 MNE 70 32.108 14.676 11

28 204 MNE 80 25.185 15.217 7

28 204 MNE 90 20.105 18.543 3

28 204 MNE 100 21.754 21.754 1

28 204 sLORETA 0 44.032 0.000 8002

28 204 sLORETA 10 43.815 0.000 5211

28 204 sLORETA 20 42.632 0.000 3044

28 204 sLORETA 30 40.895 0.000 1627

28 204 sLORETA 40 39.333 0.000 879

28 204 sLORETA 50 37.837 0.000 448

28 204 sLORETA 60 35.816 0.000 206

28 204 sLORETA 70 37.079 6.975 62

28 204 sLORETA 80 44.277 21.745 9

28 204 sLORETA 90 49.704 47.941 2

28 204 sLORETA 100 47.941 47.941 1

28 205 Ave 0 47.815 0.000 8002

28 205 Ave 10 47.717 0.000 5785

28 205 Ave 20 47.313 0.000 3712

28 205 Ave 30 46.584 0.000 2178

28 205 Ave 40 45.783 0.000 1166

28 205 Ave 50 46.218 0.000 588

28 205 Ave 60 46.281 6.204 225

28 205 Ave 70 43.054 7.792 86

28 205 Ave 80 41.164 12.319 35

28 205 Ave 90 29.954 28.542 5

28 205 Ave 100 29.132 29.132 1

28 205 cMEM 0 45.510 0.000 8002

28 205 cMEM 10 44.547 4.730 940

28 205 cMEM 20 42.815 6.541 355

28 205 cMEM 30 40.425 6.541 119

28 205 cMEM 40 36.926 6.541 48

28 205 cMEM 50 34.632 6.541 29

28 205 cMEM 60 34.774 6.541 19

28 205 cMEM 70 36.670 6.541 10

28 205 cMEM 80 37.727 6.541 6

28 205 cMEM 90 44.287 42.012 2

28 205 cMEM 100 46.072 46.072 1

28 205 dSPM 0 44.096 0.000 8002

28 205 dSPM 10 43.865 0.000 5246

28 205 dSPM 20 42.927 0.000 3096

28 205 dSPM 30 41.016 0.000 1723

28 205 dSPM 40 39.629 0.000 970

28 205 dSPM 50 38.873 0.000 516

28 205 dSPM 60 38.687 0.000 216

28 205 dSPM 70 43.004 0.000 71

28 205 dSPM 80 48.607 6.204 20

28 205 dSPM 90 55.937 50.623 7

28 205 dSPM 100 51.217 51.217 1

28 205 MNE 0 52.825 0.000 8002

28 205 MNE 10 52.914 0.000 3901

28 205 MNE 20 53.667 0.000 1705

28 205 MNE 30 54.657 0.000 750

28 205 MNE 40 55.582 6.730 321

28 205 MNE 50 57.486 9.392 141

28 205 MNE 60 60.621 17.567 57

28 205 MNE 70 64.226 51.667 15

28 205 MNE 80 60.655 51.667 6

28 205 MNE 90 57.041 51.667 2

28 205 MNE 100 51.667 51.667 1

28 205 sLORETA 0 47.853 0.000 8002

28 205 sLORETA 10 47.719 0.000 5356

28 205 sLORETA 20 47.305 0.000 3067

28 205 sLORETA 30 46.421 0.000 1553

28 205 sLORETA 40 45.194 0.000 757

28 205 sLORETA 50 42.848 4.934 333

28 205 sLORETA 60 40.164 7.792 132

28 205 sLORETA 70 36.798 7.792 64

28 205 sLORETA 80 33.534 12.319 32

28 205 sLORETA 90 29.694 23.119 7

28 205 sLORETA 100 29.132 29.132 1

28 206 Ave 0 40.942 0.000 8002

28 206 Ave 10 40.557 0.000 5158

28 206 Ave 20 38.851 0.000 3001

28 206 Ave 30 36.970 0.000 1666

28 206 Ave 40 35.413 0.000 863

28 206 Ave 50 33.228 0.000 416

28 206 Ave 60 31.045 0.000 175

28 206 Ave 70 32.364 0.000 73

28 206 Ave 80 24.269 6.244 14

28 206 Ave 90 12.642 12.642 1

28 206 Ave 100 12.642 12.642 1

28 206 cMEM 0 40.342 0.000 8002

28 206 cMEM 10 40.492 0.000 535

28 206 cMEM 20 40.878 4.497 219

28 206 cMEM 30 41.190 6.541 101

28 206 cMEM 40 41.150 25.979 60

28 206 cMEM 50 40.835 26.956 42

28 206 cMEM 60 40.937 30.629 28

28 206 cMEM 70 41.890 34.397 17

28 206 cMEM 80 41.318 34.397 11

28 206 cMEM 90 42.506 37.639 6

28 206 cMEM 100 42.190 42.190 1

28 206 dSPM 0 36.086 0.000 8002

28 206 dSPM 10 34.753 0.000 3835

28 206 dSPM 20 31.089 0.000 1647

28 206 dSPM 30 26.455 0.000 724

28 206 dSPM 40 21.711 0.000 276

28 206 dSPM 50 13.902 0.000 106

28 206 dSPM 60 12.164 0.000 54

28 206 dSPM 70 11.260 0.000 24

28 206 dSPM 80 7.398 0.000 7

28 206 dSPM 90 6.075 4.463 3

28 206 dSPM 100 7.065 7.065 1

28 206 MNE 0 46.056 0.000 8002

28 206 MNE 10 45.581 0.000 3207

28 206 MNE 20 45.134 0.000 1244

28 206 MNE 30 45.457 0.000 514

28 206 MNE 40 45.713 6.408 197

28 206 MNE 50 46.879 6.408 80

28 206 MNE 60 46.727 9.578 30

28 206 MNE 70 47.127 9.578 17

28 206 MNE 80 38.218 9.578 6

28 206 MNE 90 19.844 12.642 2

28 206 MNE 100 12.642 12.642 1

28 206 sLORETA 0 41.231 0.000 8002

28 206 sLORETA 10 40.839 0.000 5121

28 206 sLORETA 20 39.087 0.000 2877

28 206 sLORETA 30 37.109 0.000 1570

28 206 sLORETA 40 35.699 0.000 791

28 206 sLORETA 50 33.599 0.000 388

28 206 sLORETA 60 31.081 0.000 165

28 206 sLORETA 70 30.604 0.000 65

28 206 sLORETA 80 31.665 11.654 28

28 206 sLORETA 90 30.691 23.119 11

28 206 sLORETA 100 23.119 23.119 1
