# Supplementary material for: Accuracy and spatial properties of distributed magnetic source imaging techniques in the investigation of focal epilepsy patients
Source: Hum Brain Mapp. 2020 May 9;41(11):3019–33. doi: 10.1002/hbm.24994 (PMC7336148; doi:10.1002/hbm.24994)
Supplement: Supplementary file 4 — Appendix S4. Supporting Information. [file HBM-41-3019-s004.docx]

Post-hoc Curves

|  |  |  | **SD** | | **MpSize** | | **Dmin** | |
| --- | --- | --- | --- | --- | --- | --- | --- | --- |
| **Threshold** | **Inverse (I)** | **Inverse**  **(J)** | **Men Difference (I-J)** | **Sig** | **Men Difference (I-J)** | **Sig** | **Men Difference (I-J)** | **Sig** |
| **30%** | Ave | cMEM | 7.249 | 0.000 | 554.850 | 0.000 | -1.382 | 1.000 |
|  | Ave | dSPM | -3.277 | 0.000 | -81.400 | 0.003 | 0.000 |  |
|  | Ave | MNE | -1.609 | 0.000 | 366.100 | 0.000 | -0.128 | 1.000 |
|  | Ave | sLORETA | -2.910 | 0.000 | -132.310 | 0.000 | 0.000 | 1.000 |
|  | cMEM | dSPM | -10.526 | 0.000 | -636.250 | 0.000 | 1.382 | 1.000 |
|  | cMEM | MNE | -8.858 | 0.000 | -188.750 | 0.000 | 1.254 | 1.000 |
|  | cMEM | sLORETA | -10.159 | 0.000 | -687.160 | 0.000 | 1.382 | 1.000 |
|  | dSPM | MNE | 1.667 | 0.464 | 447.500 | 0.000 | -0.128 | 1.000 |
|  | dSPM | sLORETA | 0.367 | 1.000 | -50.908 | 1.000 | 0.000 |  |
|  | MNE | sLORETA | -1.301 | 0.058 | -498.410 | 0.000 | 0.128 | 1.000 |
| **60%** | Ave | cMEM | 0.682 | 1.000 | 66.990 | 0.000 | -3.113 | 0.002 |
|  | Ave | dSPM | -3.411 | 0.000 | -8.286 | 1.000 | 0.277 | 1.000 |
|  | Ave | MNE | -3.379 | 0.000 | 55.280 | 0.000 | -0.910 | 0.353 |
|  | Ave | sLORETA | -2.276 | 0.000 | -25.310 | 0.000 | 0.243 | 1.000 |
|  | cMEM | dSPM | -4.092 | 0.011 | -75.280 | 0.000 | 3.390 | 0.000 |
|  | cMEM | MNE | -4.060 | 0.038 | -11.710 | 0.000 | 2.203 | 0.070 |
|  | cMEM | sLORETA | -2.957 | 0.670 | -92.300 | 0.000 | 3.356 | 0.000 |
|  | dSPM | MNE | 0.032 | 1.000 | 63.570 | 0.000 | -1.187 | 0.236 |
|  | dSPM | sLORETA | 1.135 | 1.000 | -17.024 | 0.065 | -0.034 | 1.000 |
|  | MNE | sLORETA | 1.103 | 1.000 | -80.590 | 0.000 | 1.153 | 0.075 |
| **90%** | Ave | cMEM | -2.941 | 0.308 | 2.540 | 0.000 | -4.510 | 0.000 |
|  | Ave | dSPM | -3.262 | 0.000 | -1.199 | 0.111 | 0.058 | 1.000 |
|  | Ave | MNE | -4.297 | 0.000 | 2.490 | 0.000 | -2.781 | 0.003 |
|  | Ave | sLORETA | -1.659 | 0.100 | -2.090 | 0.000 | -0.230 | 1.000 |
|  | cMEM | dSPM | -0.320 | 1.000 | -3.740 | 0.000 | 4.567 | 0.002 |
|  | cMEM | MNE | -1.356 | 1.000 | -0.058 | 1.000 | 1.729 | 1.000 |
|  | cMEM | sLORETA | 1.282 | 1.000 | -4.640 | 0.000 | 4.279 | 0.002 |
|  | dSPM | MNE | -1.035 | 1.000 | 3.680 | 0.000 | -2.838 | 0.077 |
|  | dSPM | sLORETA | 1.603 | 1.000 | -0.893 | 1.000 | -0.288 | 1.000 |
|  | MNE | sLORETA | 2.638 | 0.008 | -4.580 | 0.000 | 2.551 | 0.032 |
